# Supplementary material for: Antioxidant Lipid Supplement on Cardiovascular Risk Factors: A Systematic Review and Meta-Analysis
Source: Nutrients. 2024 Jul 10;16(14):2213. doi: 10.3390/nu16142213 (PMC11279989; doi:10.3390/nu16142213)
Supplement: Supplementary file 1 [file nutrients-16-02213-s001.zip › nutrients-3089954-supplementary.pdf]

Contents of Supplemental Materials

SUPPLEMENTAL 1 CHARACTERISTICS OF THE PUBLICATIONS INCLUDED IN THE META-ANALYSIS 1

SUPPLEMENTAL 2 FOREST PLOT 16

SUPPLEMENTAL 3 RISK OF BIAS 60

SUPPLEMENTAL 4 FUNNEL PLOT 66

SUPPLEMENTAL 5 GRADE 110

SUPPLEMENTAL 6 RESULTS OF SENSITIVITY ANALYSIS OF THE SYSTEMATIC REMOVAL OF EACH STUDY 147

Reference 152

Supplemental 1 - Characteristics of the publications included in the meta-analysis

Table S1-1. Characteristics of the publications included in the meta-analysis (N3 fatty acids).

Article(first author, year)	Location	Study design	Health status	Gender	Intervention duration	Intervention substance	Comparison	Age, year	n (Ctrl, Inter)	Extracted outcome(s)
Abbott 2020(1)	Australia	R, DB	Healthy	M/F	12W	1g EPA	placebo	50.9±12.7	73(35,38)	HDL-C, LDL-C, TG, TC, FBG, Insulin
Agh 2017(2)	Iran	R, DB	Hypertension	M/F	8W	720mg EPA+480mg DHA	placebo	55±1.29	45(21,24)	SBP, DBP, HDL-C, LDL-C, TG, TC
Alkhedhairi 2022(3)	UK	R, DB	Healthy	M/F	6M	4g N3	placebo	70.9=5.2	90(45,45)	SBP, DBP, HDL-C, LDL-C, TG, TC, FBG, Insulin
Ansari 2017(4)	Iran	R	Diabetes	M/F	10W	3750mg N3	placebo	51.34±1.36	44(22,22)	SBP, DBP, FBG, Insulin, HbA1c
Bakker 2023(5)	Netherlands	R, DB	Obesity	F	4W	0.92g EPA+ 0.76g DHA	placebo	44=18	60(30,30)	FBG, Insulin
Barbosa 2017(6)	USA	R, DB	Hypertension	M/F	2M	N3	placebo	47.6±11	80(40,40)	SBP, DBP, HDL-C, LDL-C, TG, TC
Bischoff-Ferrari 2020(7)	Switzerland	R, DB	Healthy	M/F	5Y	1g N3	placebo	70±1	2157(1077,1080)	SBP, DBP
Cazzola(1) 2007(8)	Italy	R, DB	Healthy	M	12W	2.7g EPA	placebo	18-42	93(46,47)	HDL-C, LDL-C, TG, TC
Cazzola(2) 2007(8)	Italy	R, DB	Healthy	M	13W	4.05g EPA	placebo	53-70	62(31,31)	HDL-C, LDL-C, TG, TC
Cussons(1) 2009(9)	Australia	R, C	PCOS	F	8W	4g N3	placebo	32.7±7.7	25(13,12)	SBP, DBP, HDL-C, LDL-C, TG, TC
Cussons(2) 2009(9)	Australia	R, C	PCOS	F	8W	4g N3	placebo	32.7±7.7	25(13,12)	SBP, DBP, HDL-C, LDL-C, TG, TC

Dangardt 2010(10)	Sweden	R, DB, C	Obesity	M/F	3M	1.2g N3	placebo	15.7±10	25(13,12)	HDL-C, LDL-C, TG, TC
De Giuseppe 2023(11)	Italy	R, DB	Healthy	M/F	12W	N3	placebo	70.6=4.5	66(33,33)	HDL-C, LDL-C, TG, TC
de Lorgeril 1994(12)	France	R, SB	Hypertension	M/F	104W	N3	placebo	53.5±10	250(128,122)	SBP,HDL-C, TG, TC
Derosa 2012(13)	Italy	R, DB	Dyslipidemia	M/F	6M	3g N3	placebo	18-75	157(78,79)	SBP, DBP, HDL-C, TG, TC
Dogay Us 2022(14)	UK	R, DB	Hyperlipidemia	M/F	8W	4g EPA	placebo	60.91=8.56	41(21,20)	HDL-C, LDL-C, TG, TC
Du Plooy 1992(15)	South Africa	R, SB	Hypertension	M/F	4W	1000mg EPA	placebo	21-65	30(15,15)	DBP, HDL-C, LDL-C, TG, TC
Ebrahimi 2009(16)	Iran	R	Metabolic Syndrome	M/F	6M	180mg EPA+120mg DHA	placebo	52.9±11.9	89(42,47)	SBP, DBP, HDL-C, LDL-C, TG, TC
Elajami(1) 2017(17)	USA	R	Diabeties	M/F	1Y	1.86g EPA +1.5g DHA	control	64.5±6.9	79(40,39)	SBP, DBP, HDL-C, LDL-C, TG, TC, FBG, HbA1c
Elajami(2) 2017(17)	USA	R	Nondiabetes	M/F	1Y	1.86g EPA +1.5g DHA	control	63.6±7.6	183(88.95)	SBP, DBP, HDL-C, LDL-C, TG, TC, FBG, HbA1c
Fayh 2018(18)	Brazil	R	Diabetes	M/F	8W	300mg N3	placebo	50.47±6.06	30(15,15)	HDL-C, LDL-C, TG, TC, FBG, HbA1c
Félix-Soriano 2021(19)	Spain	R, DB	Post-Menopausal Women	F	16W	1950mg N3	control	55-70	35(20,15)	SBP, DBP, HDL-C, LDL-C, TG, TC
Finnegan(1) 2003(20)	UK	R, DB	Hyperlipidemia	M/F	6M	0.8g N3	control	53±2	60(30,30)	SBP, DBP, HDL-C, LDL-C, TG, TC
Finnegan(2) 2003(20)	UK	R, DB	Hyperlipidemia	M/F	6M	4.5g ALA	control	53±2	60(30,30)	SBP, DBP, HDL-C, LDL-C, TG, TC
Finnegan(3) 2003(20)	UK	R, DB	Hyperlipidemia	M/F	6M	1.7g N3	control	53±2	60(30,30)	SBP, DBP, HDL-C, LDL-C, TG, TC

Finnegan(4) 2003(20)	UK	R, DB	Hyperlipidemia	M/F	6M	9.5g ALA	control	53±2	60(30,30)	SBP, DBP, HDL-C, TG
Ginty 2012(21)	USA	R, DB	Healthy	M/F	21D	1.4g N3	control	21±0.99	34(17,17)	SBP, DBP, LDL-C
Golpour 2020(22)	Iran	R, DB	Diabetes	M/F	10W	2700mg N3	placebo	51.15±7.45	61(30,31)	FBG, Insulin, HbA1c
Golzari 2017(23)	Iran	R, DB	Diabetes	M/F	8W	2g EPA	placebo	44.72±4.69	36(18,18)	SBP, DBP, HDL-C, LDL-C, TG, TC. FBG, HbA1c
Haghiac 2015(24)	USA	R, DB	Obesity	F	25W	800mg DHA+1200mg EPA	placebo	27±5	49(24,25)	FBG, Insulin
Harris(1) 2008(25)	USA	R, DB	Healthy	M/F	16W	1g EPA	placebo	42.3±10.1	22(11,11)	SBP, DBP, HDL-C, LDL-C, TG, TC
Harris(2) 2008(25)	USA	R, DB	Healthy	M/F	16W	1g EPA	placebo	42.3±10.1	22(11,11)	SBP, DBP, HDL-C, LDL-C, TG, TC
Holman 2009(26)	UK	R	Diabetes	M/F	28W	2000mg N3	placebo	63.5±11.7	400(200,200)	SBP, DBP, HDL-C, LDL-C, TC, HbA1c
Howe(1) 2018(26)	Australia	R, DB	Healthy	M/F	20W	1600mg DHA+400mg EPA	placebo	63.8±1.9	38(19,19)	SBP, DBP
Howe(2) 2018(26)	Australia	R, DB	Healthy	M/F	20W	1600mg DHA+400mg EPA	placebo	63.8±1.9	38(19,19)	SBP, DBP
Howe(3) 2018(26)	Australia	R, DB	Healthy	M/F	20W	1600mg DHA+400mg EPA	placebo	63.8±1.9	38(19,19)	SBP, DBP
Jamilian 2017(27)	Iran	R, DB	Diabetes	F	6W	240mg DHA +360mg EPA	placebo	30.7±4.1	70(35,35)	HDL-C, LDL-C, TG, TC, FBG, Insulin
Jamilian 2018(28)	Iran	R, DB	Diabetes	F	6W	240mg DHA +360mg EPA	placebo	30.8±2.4	40(20,20)	HDL-C, LDL-C, TG, TC, FBG, Insulin
Khani 2017(29)	Iran	R, DB	PCOS	F	24W	2000mg N3	placebo	31.04±5.04	88(44,44)	HDL-C, LDL-C, TG, TC, FBG

Kobayashi 2023(30)	Japan	R, DB	Dyslipidemia	M/F	3M	1.86g EPA +1.5g DHA	placebo	73.6=12.7	38(19,19)	HDL-C, LDL-C, TG
Lee 2015(31)	Korea	R, DB	Diabetes	M/F	12W	1.38g EPA +1.14g DHA	placebo	60.4±1.2	19(11,8)	SBP, DBP, HDL-C, LDL-C, TG, TC, FBG, HbA1c
Lee(1) 2019(32)	Canada	R, DB	Healthy	M/F	12W	3g EPA	olive oil	21±2	58(30,28)	SBP, DBP
Lee(2) 2019(32)	Canada	R, DB	Healthy	M/F	12W	3g DHA	olive oil	22±2	58(30,28)	SBP, DBP
Liu 2022(33)	China	R, DB	Diabetes	M/F	6m	3g N3	placebo	62.45=8.58	100(50,50)	SBP, DBP, HDL-C, LDL-C, TG, TC, FBG, Insulin, HbA1c
Logan 2015(34)	Japan	R	Healthy	F	12W	3g N3	placebo	66±1	24(12,12)	SBP, DBP, TG
Lungershausen 1997(35)	Australia	R, DB	Diabetes	M/F	12W	2g EPA +1.4g DHA	placebo	55±1.29	32(16,16)	SBP, DBP, FBG, HbA1c
Manor 2013(36)	Israel	R, DB	Attention-Deficit Hyperactivity Disorder	M/F	15W	300mg N3	placebo	6 月 13 日	162(81,81)	SBP, DBP, HDL-C, LDL-C, TG, TC
Mejia-Montilla 2018(37)	Spain	R, DB	PCOS	F	12W	1000mg N3	placebo	23.6±6.4	200(100,100)	HDL-C, LDL-C, TG, TC, Insulin
Mengelberg 2022(38)	New Zealand	R, DB	Memory Complaints	M/F	12M	1.5g DHA+0.35g EPA	placebo	72.33=6.16	60(30,30)	SBP, DBP
Miller 2013(39)	USA	R, C	Diabetes	M/F	8W	1.38g EPA +1.14g DHA	placebo	60.4±1.2	31(17,14)	SBP, DBP, HDL-C, LDL-C, TG, TC, FBG
Miyajima 2001(40)	Japan	R, DB, C	Hypertension	M	8W	2.7g EPA	placebo	44.8±6.4	77(39,38)	SBP, DBP, HDL-C, TG
Moosavi 2022(41)	USA	R	Healthy	M/F		3.9g N3	placebo	71.4±4.40	44(22,22)	SBP, DBP, HDL-C, LDL-C, TG, TC
Mori 2009(42)	Australia	R, DB	Healthy	M/F	3W	4g N3	placebo	56.5±1.4	35(15,20)	SBP, DBP, HDL-C, LDL-C, TG, TC

Mori(1) 1999(43)	Australia	R, DB	Hypercholesterolemic	M	6W	4g EPA	placebo	48.8±1.1	39(20,19)	HDL-C, LDL-C, TG, TC, FBG, Insulin
Mori(2) 1999(43)	Australia	R, DB	Hypercholesterolemic	M	6W	4g DHA	placebo	48.8±1.1	37(20,17)	HDL-C, LDL-C, TG, TC, FBG, Insulin
Mozaffarian 2022(44)	USA	R, DB	Hypertriglyceridemia	M/F	26W	4g N3	placebo	54.9=11.2	520(148,372)	HDL-C, LDL-C, TG
Murphy 2007(45)	Australia	R	Overweight	M/F	6M	1g N3	placebo	50.2±9.4	70(32,38)	SBP, DBP, HDL-C, LDL-C, TG, TC
Neff 2011(46)	USA	R, DB	Overweight	M/F	4.5M	2g DHA	placebo	44±10	36(18,18)	HDL-C, LDL-C, TG, TC
O'Mahoney 2020(47)	UK	R, DB	Diabetes	M/F	9M	3.3g N3	placebo	34±15	20(10,10)	SBP, DBP, TG, TC
Peña-de-la-Sancha 2023(48)	Mexico	R, SB	Hypertriglyceridemia	M/F	5W	0.46g EPA+0.38g DHA	placebo	42.5=6.2	18(9,9)	SBP, DBP, HDL-C, TG, TC, FBG
Pooya 2010(49)	Iran	R, DB	Diabetes	M/F	8W	2714mg N3	placebo	56.38±9.24	80(40,40)	LDL-C, TC, FBG, HbA1c
Rafraf 2012(50)	Iran	R, DB	PCOS	F	8W	4000mg N3	placebo	27.3±64.2	60(30,30)	FBG, Insulin
Root 2013(51)	USA	R, DB	Healthy	M/F	4W	1.7g N3	placebo	18-30	57(27,30)	SBP, HDL-C, LDL-C
Rossing 1996(52)	Denmark	R, DB	Diabetes	M/F	52W	2g EPA +2.6g DHA	placebo	33±1.3	29(15,14)	SBP, DBP, HDL-C, LDL-C, TG, TC, HbA1c
Samimi 2015(53)	Iran	R, DB	Diabetes	F	6W	120mg DHA +180mg EPA	placebo	30.3±5.5	56(28,28)	HDL-C, LDL-C, TG, TC, FBG, Insulin
Sanders 2006(54)	UK	R	Healthy	M/F	4W	1.5g DHA+0.6g EPA	placebo	31.6±12.9	79(39,40)	SBP, DBP, HDL-C, LDL-C, TG, TC
Sanders 2011(55)	UK	R, C	Healthy	M/F	12M	1.8g N3	placebo	55±2.1	178(88,90)	SBP, DBP, HDL-C, LDL-C, TG

Sarbolouki 2013(56)	Iran	R, DB	Diabetes	M/F	12W	2000mg N3	placebo	45.3±3.93	67(32,35)	FBG, Insulin, HbA1c
Satoh 2009(57)	Japan	R	Obesity	M/F	3M	1.8g EPA	placebo	51.7±1.5	92(46,46)	SBP, DBP, HDL-C, LDL-C, TG, TC, FBG, Insulin, HbA1c
Shimizu 1995(58)	Japan	R	Diabetes	M/F	12M	900mg EPA	placebo	66.3±2.5	25(16,29)	SBP, DBP, HDL-C, TG, TC, FBG, HbA1c
Singhai 2013(59)	UK	R, DB	Healthy	M/F	4M	4g DHA	placebo	27.6±4.7	274(138,136)	SBP, DBP, HDL-C, LDL-C, TG, TC, FBG, Insulin
Stark 2003(60)	Canada	R, DB, C	Healthy	F	4W	6g DHA	placebo	56.7±6.2	64(32,32)	SBP, DBP, HDL-C, LDL-C, TG, TC, FBG, Insulin
Stupin 2018(61)	Croatia	R, DB	Healthy	M/F	3W	277mg N3	placebo	21±1	36(18,18)	SBP, DBP, HDL-C, LDL-C, TG, TC
Takaki 2011(62)	Japan	R	Hypertension	M/F	48W	1800mg EPA	placebo	61.6±5.6	50(25,25)	SBP, DBP, HDL-C, LDL-C
Takeshita 2022(63)	Japan	R, DB	Diabetes	M/F	12W	1.8g EPA	placebo	18-30	20(10,10)	SBP, HDL-C, TG, TC, FBG, HbA1c
Theobald 2007(64)	UK	R, DB, C	Hypertension	M/F	3M	0.7g DHA	placebo	51.1±7.4	38(19,19)	SBP, DBP
Thota 2020(65)	Australia	R	Obesity	M/F	12W	2g DHA	placebo	51.1±12.9	58(27,31)	FBG, Insulin
Tomiyama 2005(66)	Japan	R	Dyslipidemia	M/F	13M	1.8g EPA	placebo	65±2	84(40,44)	SBP, DBP, HDL-C, TG, TC, FBG
Tsunoda(1) 2015(67)	USA	R, DB	Healthy	M/F	6W	1.8g EPA	placebo	53.6±8.6	32(16,16)	HDL-C, LDL-C, TC, FBG, Insulin
Tsunoda(2) 2015(67)	USA	R, DB	Healthy	M/F	6W	1.8g DHA	placebo	51.6±9.7	34(16,18)	HDL-C, LDL-C, TC, FBG, Insulin
Udupa 2013(68)	India	R, DB	Diabetes	M/F	13W	300mg N3	placebo	53.5±1.4	50(25,25)	FBG, HbA1c

Vakhapova 2011(69)	Israel	R, DB	Memory Complaints	M/F	15W	300mg DHA	placebo	72.36±7.93	121(60,61)	SBP, DBP, HDL-C, LDL-C, TG, TC
Veleba 2015(70)	Czech Republic	R	Diabetes	M/F	24W	5000mg EPA/DHA	placebo	62±1.4	60(30,30)	SBP, DBP, HDL-C, LDL-C, TG, TC, FBG, HbA1c
West 2010(71)	Canada	R, C	Hypercholesterolemic	M/F	18W	ALA	placebo	49.3±1.7	12(6,6)	SBP
Wong 2013(72)	Australia	R, SB	Obesity	M/F	12W	4g N3	placebo	60±4	25(12,13)	SBP, DBP, HDL-C, LDL-C, TG, TC
Woodman(1) 2002(73)	Australia	R, DB	Diabetes	M/F	6W	4000mg EPA	placebo	61.2±9.6	26(12,14)	SBP, DBP, HDL-C, LDL-C, TG, TC, FBG, Insulin, HbA1c
Woodman(2) 2002(73)	Australia	R, DB	Diabetes	M/F	6W	4000mg DHA	placebo	60.9±8.2	25(13,12)	SBP, DBP, HDL-C, LDL-C, TG, TC, FBG, Insulin, HbA1c
Yamamoto 2005(74)	Japan	R	Hypercholesterolemic	M/F	6M	0.9g EPA	placebo	71.6±4.8	60(29,31)	HDL-C, LDL-C, TG, FBG, Insulin, HbA1c
Zeman 2005(75)	Czech Republic	R, SB	Diabetes	M/F	52W	2.07g EPA +1.53g DHA	placebo	48.8±1.1	24(12,12)	TG, TC, FBG, HbA1c

Data are presented as mean ± SD or as a range.

R, randomized; SB, single-blinded; DB, double-blinded;C, crossover design; F, female; M, male; Ctrl, control group; Inter, Intervention group; W, week; M, month; D, day; PCOS, polycystic ovary syndrome; SBP, systolic blood pressure; DBP, diastolic blood pressure; TG, triglyceride; TC, total cholesterol; HDL-C, high density lipoprotein cholesterol; LDL-C, low density lipoprotein-cholesterol; FBG, fasting blood glucose; EPA, Eicosapentaenoic Acid; DHA, Docosahexaenoic Acid; ALA, alpha-linolenic acid.

Table S1-2. Characteristics of the publications included in the meta-analysis (N6 fatty acids).

Article(first author, year)	Location	Study design	Health status	Gender	Intervention duration	Intervention substance	Comparison	Age, year	n (Ctrl, Inter)	Extracted outcome(s)
Aryaeian 2008(76)	Iran	R, DB	Rheumatoid Arthritis	M/F	12W	2.5g CLA	placebo	46.23±2.79	44(22,22)	HDL-C, LDL-C, TG, TC
Carvalho 2012(77)	Brazil	R	Metabolic Syndrome	F	90D	3g CLA	placebo	41±3.97	14(7,7)	SBP, DBP, HDL-C, LDL-C, TG, TC
Damsgaard(1) 2008(78)	Brazil	R, DB	Healthy	M	8W	7.3g linoleic acid	low linoleic acid	25.6±4.6	64(23,23)	HDL-C, LDL-C, TG, TC
Damsgaard(2) 2008(78)	Brazil	R, DB	Healthy	M	8W	7.3g linoleic acid	low linoleic acid	25.6±4.6	64(23,23)	HDL-C, LDL-C, TG, TC
Diaz 2008(79)	USA	R, DB	Overweight	F	12W	1.8g CLA	placebo	36±1	35(17,18)	SBP, DBP, HDL-C, LDL-C, TG, TC
Dullaart 1992(80)	Netherlands	R	Diabetes	M/F	2Y	high linoleic acid diet	usual diet	44±12	36(20,16)	HbA1c
Engberink(1) 2012(81)	Netherlands	R, C	Healthy	M/F	9W	CLA	Oleic acid diet	30.9±13.7	61(30,31)	SBP, DBP
Engberink(2) 2012(81)	Netherlands	R, C	Healthy	M/F	9W	CLA	Industrial trans diet	30.9±13.7	61(30,31)	SBP, DBP
Ferrara 2000(82)	Italy	R	Hypertension	M/F	6M	20g linoleic acid	olive oil	25-70	46(23,23)	SBP, DBP, HDL-C, TG, TC, FBG
Iwata(1) 2007(83)	Japan	R, DB	Overweight	M/F	12W	3.4g CLA	placebo	43.4±9.8	60(20,20/20)	SBP, DBP, HDL-C, LDL-C, TG, TC
Iwata(2) 2017(83)	Japan	R, DB	Overweight	M/F	12W	6.8g CLA	placebo	43.4±9.8	60(20,20/20)	SBP, HDL-C, LDL-C, TG, TC
Kaźmierska 2022(84)	Poland	R, DB	Healthy	M/F	9M	N6	placebo	22.1.92	50(25,25)	HDL-C, LDL-C, TG

Kestin(1) 1990(85)	Australia	R	Hyperlipidemia	M/F	3W	14.3g linoleic acid	fish oil	28.6±2.6	62(31,31)	SBP, DBP, HDL-C, LDL-C, TG, TC
Kestin(2) 1990(85)	Australia	R	Hyperlipidemia	M/F	3W	14.3g linoleic acid	linoleic acid	28.6±2.6	62(31,31)	SBP, DBP, HDL-C, LDL-C, TG, TC
Kriketos 2001(86)	USA	R	Hypertension	M/F	10W	N6	placebo	45.67±1.94	36(19,17)	SBP, DBP, HDL-C, LDL-C, TG, TC
Laidlaw(1) 2003(87)	Canada	R	Healthy	F	28D	5 g EPA+DHA and 1 g GLA	4 g EPA +DHA	48±1.9	16(8,8)	HDL-C, LDL-C, TG, TC
Laidlaw(2) 2003(87)	Canada	R	Healthy	F	28D	5 g EPA+DHA and 2 g GLA	4 g EPA +DHA	48±1.9	15(8,7)	HDL-C, LDL-C, TG, TC
Laidlaw(3) 2003(87)	Canada	R	Healthy	F	28D	5 g EPA+DHA and 4 g GLA	4 g EPA +DHA	48±1.9	16(8,8)	HDL-C, LDL-C, TG, TC
Lee 2012(88)	Australia	R	Hypercholesterolemic	M/F	4W	N6	placebo	30-57	11(6,5)	SBP, DBP, HDL-C, LDL-C, TG, TC
Leng 1998(89)	UK	R	Stable Intermittent Claudication	M/F	24M	GLA	placebo	65±0.94	120(60,60)	HDL-C, LDL-C, TC
Miller 2016(90)	USA	R	Metabolic Syndrome	M/F	6M	linoleic acid	monounsaturated fat	60.9±8.5	88(44,44)	SBP, DBP, HDL-C, LDL-C, TG, TC, FBG, Insulin
Raff 2006(91)	Denmark	R, DB	Healthy	M	5W	4.7g CLA	control	25.8±3.9	38(20,18)	SBP, DBP

Data are presented as mean ± SD or as a range.

R, randomized; DB, double-blinded; C, crossover design; F, female; M, male; Ctrl, control group; Inter, Intervention group; W, week; M, month; D, day; PCOS, polycystic ovary syndrome; SBP, systolic blood pressure; DBP, diastolic blood pressure; TG, triglyceride; TC, total cholesterol; HDL-C, high density lipoprotein cholesterol; LDL-C, low density lipoprotein-cholesterol; FBG, fasting blood glucose; EPA, Eicosapentaenoic Acid; DHA, Docosahexaenoic Acid; ALA, alpha-linolenic acid; CLA, conjugated linoleic acid.

Table S1-3. Characteristics of the publications included in the meta-analysis (N9 fatty acids).

Article(first author, year)	Location	Study design	Health status	Gender	Intervention duration	Intervention substance	Comparison	Age, year	n (Ctrl, Inter)	Extracted outcome(s)
Binkoski 2005(92)	USA	R, DB	Hypercholesterolemia	M/F	6W	Mid-Oleic Sunflower Oil	olive oil	46.2±0.8	30(15,15)	HDL-C, LDL-C, TG, TC
Bowen 2019(93)	Canada	R, DB, C	Metabolic Syndrome	M/F	6W	High-Oleic Acid Canola Oil	conventional canola oil	44±13	80(40,40)	HDL-C, LDL-C, TG, TC
Gillingham 2011(94)	Canada	R, SB, C	Hypercholesterolemia	M/F	28D	High-Oleic Rapeseed Oil Diet	western diet	47.49±11.93	36(18,18)	HDL-C, LDL-C, TG, TC
Gilmore 2011(95)	USA	R	Healthy	M	5W	High-Oleic Acid Diet	low-oleic acid diet	35.8±1.3	40(20,20)	HDL-C, LDL-C, TG, TC
González-Rámila(1) 2022(96)	Spain	R, DB, C	Healthy	M/F	4W	Olive pomace oil	sunflower oil	30=2	30(15,15)	HDL-C, LDL-C, TG, TC, FBG, Insulin, HbA1c
González-Rámila(1) 2023(96)	Spain	R, DB, C	Healthy	M/F	4W	Olive pomace oil	sunflower oil	35=70	34(17,17)	SBP, DBP, HDL-C, LDL-C, TG, TC
González-Rámila(2) 2022(97)	Spain	R, DB, C	Hypercholesterolemia	M/F	4W	Olive pomace oil	sunflower oil	30=2	36(18,18)	HDL-C, LDL-C, TG, TC, FBG, Insulin, HbA1c
González-Rámila(2) 2023(97)	Spain	R, DB, C	Hypercholesterolemia	M/F	4W	Olive pomace oil	sunflower oil	44=52	30(15,15)	SBP, DBP, HDL-C, LDL-C, TG, TC
Jones 2014(98)	Canada	R, DB, C	Obesity	M/F	8W	High-Oleic Acid Canola Oil	conventional canola oil	43.87±15.81	52(26,26)	HDL-C, LDL-C, TG, TC
Jones 2015(99)	Canada	R, DB, C	Healthy	M/F	8W	High-Oleic Acid Canola Oil	conventional canola oil	45.8±12.9	30(15,15)	HDL-C, LDL-C, TG, TC

Liu 2018(100)	USA	R, C	Metabolic Syndrome	M/F	28D	High-Oleic Acid Canola Oil	conventional canola oil	49.5±12	40(20,20)	HDL-C, LDL-C, TG, TC
Loganathan 2022(101)	Malaysia	R, SB, C	Healthy	M/F	4W	palm olein	extra virgin olive oil	33.68=4.82	40(20,20)	HDL-C, LDL-C, TG, TC, FBG, Insulin
Pu 2016(102)	Canada	R, DB, C	Hypertension	M/F	30D	High-Oleic Acid Canola Oil	conventional canola oil	45.6±14.7	40(20,20)	HDL-C, LDL-C, TG, TC
Rodríguez-Pérez 2016(103)	Spain	R, DB	Metabolic Syndrome	M/F	8W	High-Oleic Acid Canola Oil	conventional canola oil	43.87±15.81	36(18,18)	HDL-C, LDL-C, TG
Sanders 2003(104)	UK	R, C	Healthy	M	2W	Oleate Diet	carbohydrate diet	24.2±5.9	30(15,15)	HDL-C, LDL-C, TG, TC
Tindall 2019(105)	USA	R, C	Hypertension	M/F	8W	Oleic Acid﹣Replaces﹣A-Linolenic Acid Diet	standard Western diet	43±10	22(11,11)	SBP, DBP, HDL-C, LDL-C, TG, TC

Data are presented as mean ± SD or as a range.

R, randomized; SB, single-blinded; DB, double-blinded; C, crossover design; F, female; M, male; Ctrl, control group; Inter, Intervention group; W, week; M, month; D, day; PCOS, polycystic ovary syndrome; SBP, systolic blood pressure; DBP, diastolic blood pressure; TG, triglyceride; TC, total cholesterol; HDL-C, high density lipoprotein cholesterol; LDL-C, low density lipoprotein-cholesterol; FBG, fasting blood glucose.

Table S1-4. Characteristics of the publications included in the meta-analysis (Lycopene).

Article(first author, year)	Location	Study design	Health status	Gender	Intervention duration	Intervention substance	Comparison	Age, year	n (Ctrl, Inter)	Extracted outcome(s)
Amstrup 2015	Denmark	R, DB	Osteopenia	F	1Y	1mg melatonin	placebo	62.9±4.7	81(40,41)	Insulin
Bazyar 2021	Iran	R, DB	Diabetes	M/F	8W	2.5mg melatonin	placebo	52.52±5.65	50(25,25)	SBP
Gajendragadkar(1) 2014(106)	UK	R, DB	Healthy	M/F	2M	7mg lycopene	placebo	67±7	36(12,24)	SBP, DBP, HDL-C, LDL-C
Gajendragadkar(2) 2014(106)	UK	R, DB	Healthy	M/F	2M	7mg lycopene	placebo	67±7	36(12,24)	SBP, DBP, HDL-C, LDL-C
Kim(1) 2011(107)	Korea	R	Healthy	M	8W	6mg lycopene	placebo	34.3±1.21	79(38,41)	SBP
Kim(2) 2011(107)	Korea	R	Healthy	M	8W	15mg lycopene	placebo	34.3±1.21	75(38,37)	SBP
Lane(1) 2018(108)	UK	R	PSA	M	6M	15mg lycopene	placebo	63.7±4.8	89(45,44)	SBP
Lane(2) 2018(108)	UK	R	PSA	M	6M	15mg lycopene	placebo	63.7±4.8	89(45,44)	SBP
Paran 2009(109)	Israel	R, DB, C	Hypertension	M/F	6W	15mg lycopene	placebo	61.4±8.9	54(27,27)	SBP, DBP
Park 2013(110)	USA	R	Healthy	M	3M	Lycopene	control	67.8±7.3	34(12,22)	SBP, DBP
Ried 2009(111)	Australia	R	Prehypertension	M/F	12W	15mg lycopene	placebo	53.8±12.7	25(10,15)	SBP, DBP
Thies(1) 2012(112)	UK	R, SB	Healthy	M/F	12W	10mg lycopene	control	51.1±0.8	157(76,81)	SBP, DBP, HDL-C, LDL-C, TG, TC

Thies(2) 2012(112)	UK	R, SB	Healthy	M/F	12W	10mg lycopene	control	51.1±0.8	144(76,68)	SBP, DBP, HDL-C, LDL-C, TG, TC
Wolak(1) 2019(113)	Israel	R, DB	Hypertension	M/F	8W	5mg lycopene	placebo	52.4±8.2	61(30,31)	SBP, DBP
Wolak(2) 2019(113)	Israel	R, DB	Hypertension	M/F	8W	15mg lycopene	placebo	52.4±8.2	61(30,31)	SBP, DBP
Wolak(3) 2019(113)	Israel	R, DB	Hypertension	M/F	8W	30mg lycopene	placebo	52.4±8.2	61(30,31)	SBP, DBP
Wolak(4) 2019(113)	Israel	R, DB	Hypertension	M/F	8W	15mg lycopene	placebo	52.4±8.2	61(30,31)	SBP, DBP

Data are presented as mean ± SD or as a range.

R, randomized; SB, single-blinded; DB, double-blinded; C, crossover design; F, female; M, male; Ctrl, control group; Inter, Intervention group; W, week; M, month; D, day; PSA, prostate cancer; SBP, systolic blood pressure; DBP, diastolic blood pressure; TG, triglyceride; TC, total cholesterol; HDL-C, high density lipoprotein cholesterol; LDL-C, low density lipoprotein-cholesterol.

Table S1-5. Characteristics of the publications included in the meta-analysis (Astaxanthin).

Article(first author, year)	Location	Study design	Health status	Gender	Intervention duration	Intervention substance	Comparison	Age, year	n (Ctrl, Inter)	Extracted outcome(s)
Choi 2011(114)	Korea	R, DB	Overweight	M/F	12W	20mg Astaxanthin	placebo	30.1=9.6	27(13,14)	HDL-C, LDL-C, TG, TC
Jabarpour 2024(115)	Iran	R, TB	PCOS	F	8W	12mg Astaxanthin	placebo	30.84=4.84	53(26,27)	SBP, DBP, HDL-C, LDL-C, TG, TC
Saeidi 2023 (1)(116)	Iran	R	Obesity	M	12W	20mg Astaxanthin	placebo	27.6=8.4	34(17,17)	HDL-C, LDL-C, TG, TC, FBG, Insulin
Saeidi 2023 (2)(116)	Iran	R	Obesity	M	12W	20mg Astaxanthin	placebo	27.6=8.4	34(17,17)	HDL-C, LDL-C, TG, TC, FBG, Insulin
Spiller 2003(117)	USA	R, DB	Healthy	M/F	8W	6mg Astaxanthin	placebo	58=9	35(16,19)	SBP, DBP, FBG
Urakaze 2021(118)	Japan	R, DB	Healthy	M/F	12W	12mg Astaxanthin	placebo	46.2=13.7	44(21,23)	HDL-C, LDL-C, TG, TC
Yoshida 2010 (1)(119)	Japan	R, DB	Hyperlipidemia	M/F	12W	6mg Astaxanthin	placebo	47.0=7.0	30(15,15)	SBP, DBP, HDL-C, LDL-C, TG, TC, FBG
Yoshida 2010 (2)(119)	Japan	R, DB	Hyperlipidemia	M/F	12W	12mg Astaxanthin	placebo	42.8=8.8	30(15,15)	SBP, DBP, HDL-C, LDL-C, TG, TC, FBG
Yoshida 2010 (3)(119)	Japan	R, DB	Hyperlipidemia	M/F	12W	18mg Astaxanthin	placebo	43.8=10.4	30(15,15)	SBP, DBP, HDL-C, LDL-C, TG, TC, FBG

Data are presented as mean ± SD or as a range.

R, randomized; DB, double-blinded; TB, triple-blinded; C, crossover design; F, female; M, male; Ctrl, control group; Inter, Intervention group; W, week; M, month; D, day; PCOS,

polycystic ovary syndrome; SBP, systolic blood pressure; DBP, diastolic blood pressure; TG, triglyceride; TC, total cholesterol; HDL-C, high density lipoprotein cholesterol; LDL-C, low density lipoprotein-cholesterol; FBG, fasting blood glucose.

Table S1-6. Characteristics of the publications included in the meta-analysis (Beta-carotene).

Article(first author, year)	Location	Study design	Health status	Gender	Intervention duration	Intervention substance	Comparison	Age, year	n (Ctrl, Inter)	Extracted outcome(s)
Asemi 2016(120)	Iran	R, DB, C	Diabetes	M/F	6W	50mg beta-carotene	placebo	52.9=8.1	102(51.51)	HDL-C, LDL-C, TG, TC
Nierenberg 1991(121)	USA	R	Healthy	M/F	12M	50mg beta-carotene	placebo	18-30	61(30,31)	HDL-C, TG, TC
Redlich 1999(122)	USA	R	Healthy	M/F	10M	30mg beta-carotene	placebo	54=6	52(23,29)	HDL-C, LDL-C, TG, TC
Ribaya-Mercado 1995(123)	USA	R	Healthy	F	3W	90mg beta-carotene	placebo	65.9=1.7	10(5,5)	HDL-C, LDL-C, TG, TC
Shaish 2006(124)	Israel	R, DB	Healthy	M/F	6W	60mg beta-carotene	placebo	43.9=10.2	22(11,11)	HDL-C, TG, TC
van Poppel 1994(125)	Netherlands	R, DB	Healthy	M	14W	20mg beta-carotene	placebo	40.0=10.4	50(25,25)	HDL-C, TC

Data are presented as mean ± SD or as a range.

R, randomized; DB, double-blinded; F, female; M, male; Ctrl, control group; Inter, Intervention group; W, week; M, month; TG, triglyceride; TC, total cholesterol; HDL-C, high density lipoprotein cholesterol; LDL-C, low density lipoprotein-cholesterol.

Supplemental 2-Forest plot

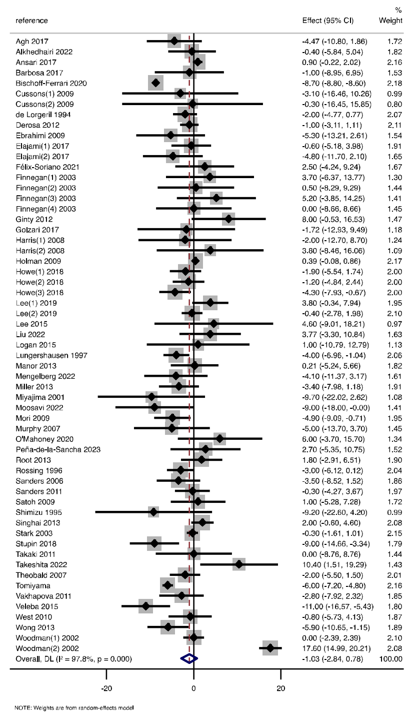

Figure S1-1-1 Forest plot of RCTs investigating the effect of n3 fatty acids supplementation on SBP.

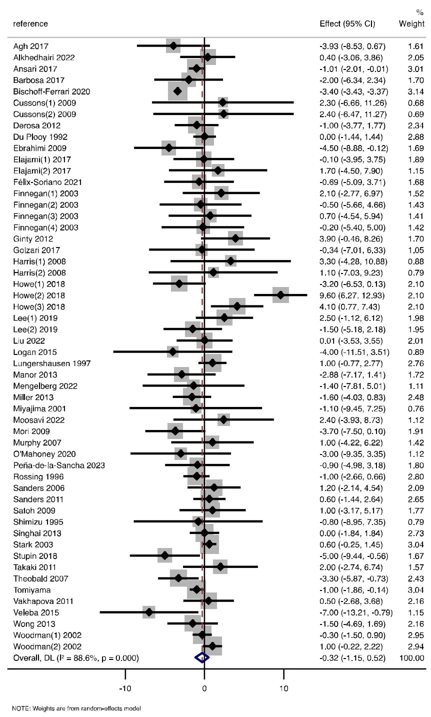

Figure S1-1-2 Forest plot of RCTs investigating the effect of n3 fatty acids supplementation on DBP.

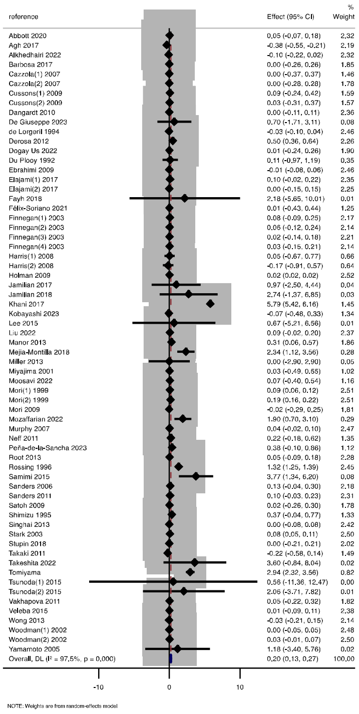

Figure S1-1-3 Forest plot of RCTs investigating the effect of n3 fatty acids supplementation on HDL-C.

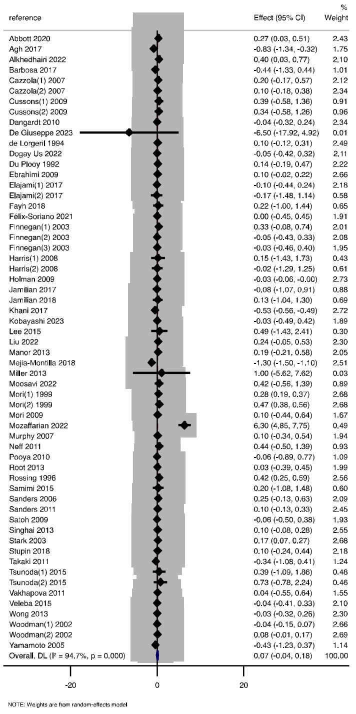

Figure S1-1-4 Forest plot of RCTs investigating the effect of n3 fatty acids supplementation on LDL-C.

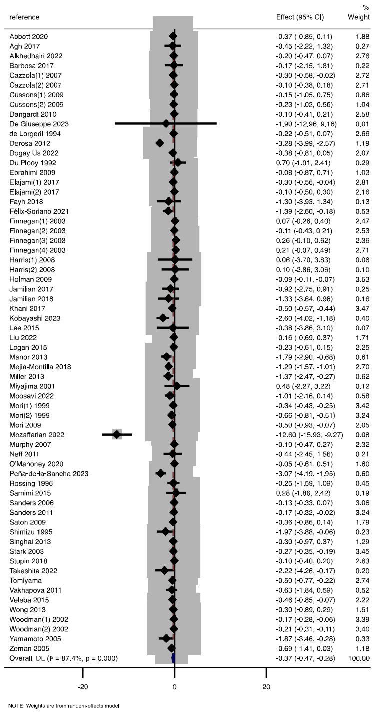

Figure S1-1-5 Forest plot of RCTs investigating the effect of n3 fatty acids supplementation on TG

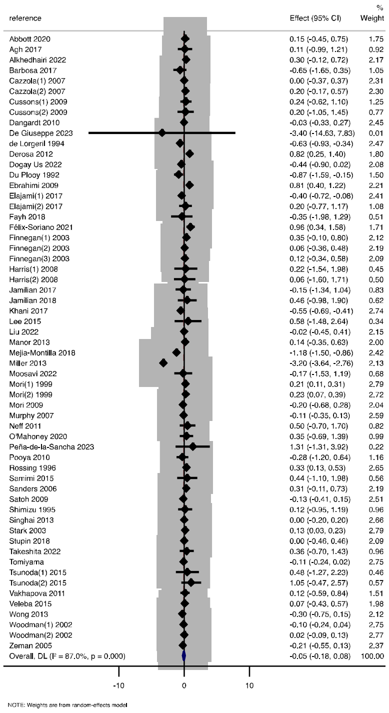

Figure S1-1-6 Forest plot of RCTs investigating the effect of n3 fatty acids supplementation on TC.

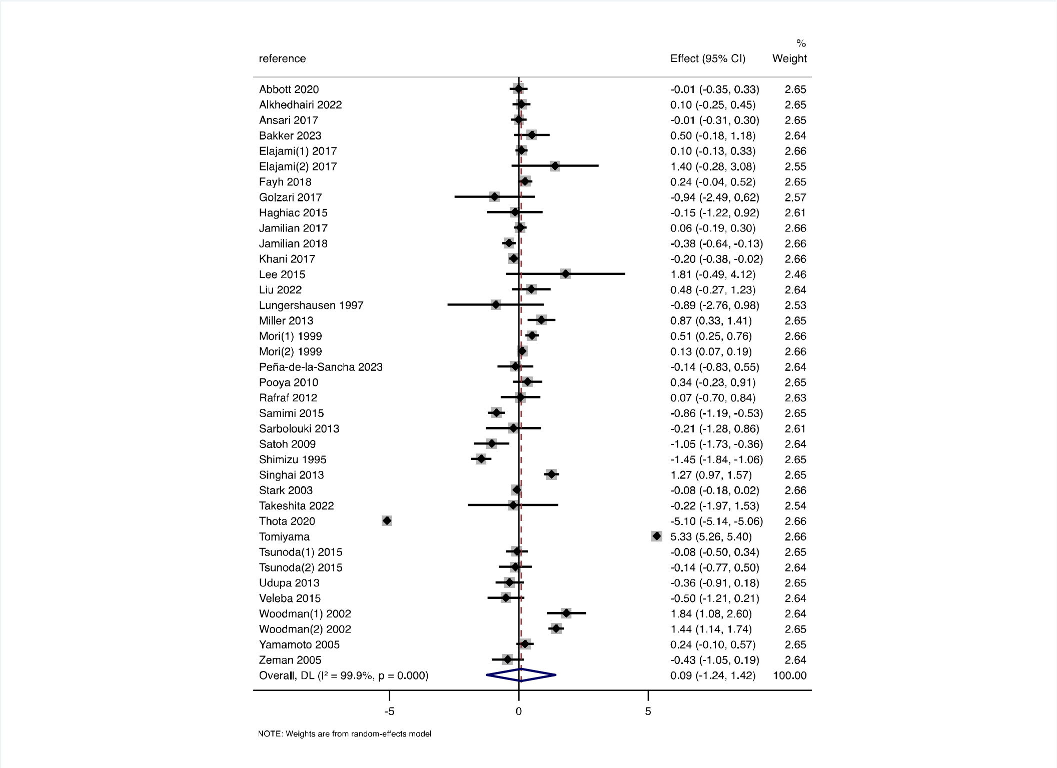

Figure S1-1-7 Forest plot of RCTs investigating the effect of n3 fatty acids supplementation on FBG.

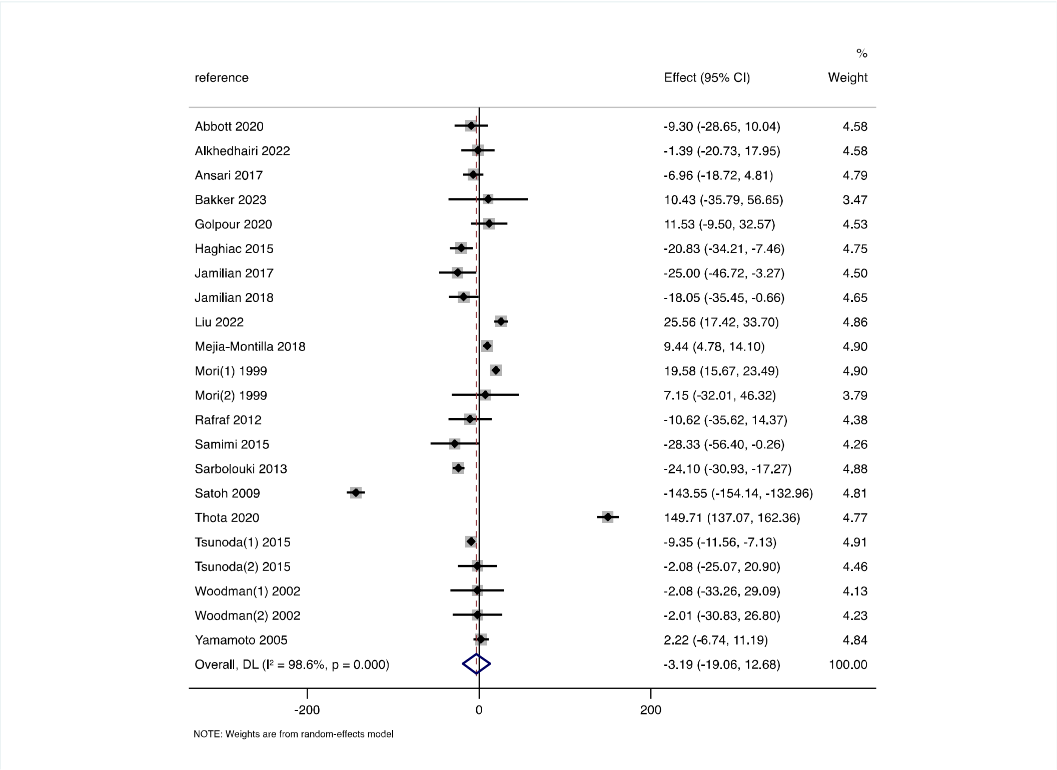

Figure S1-1-8 Forest plot of RCTs investigating the effect of n3 fatty acids supplementation on FBI.

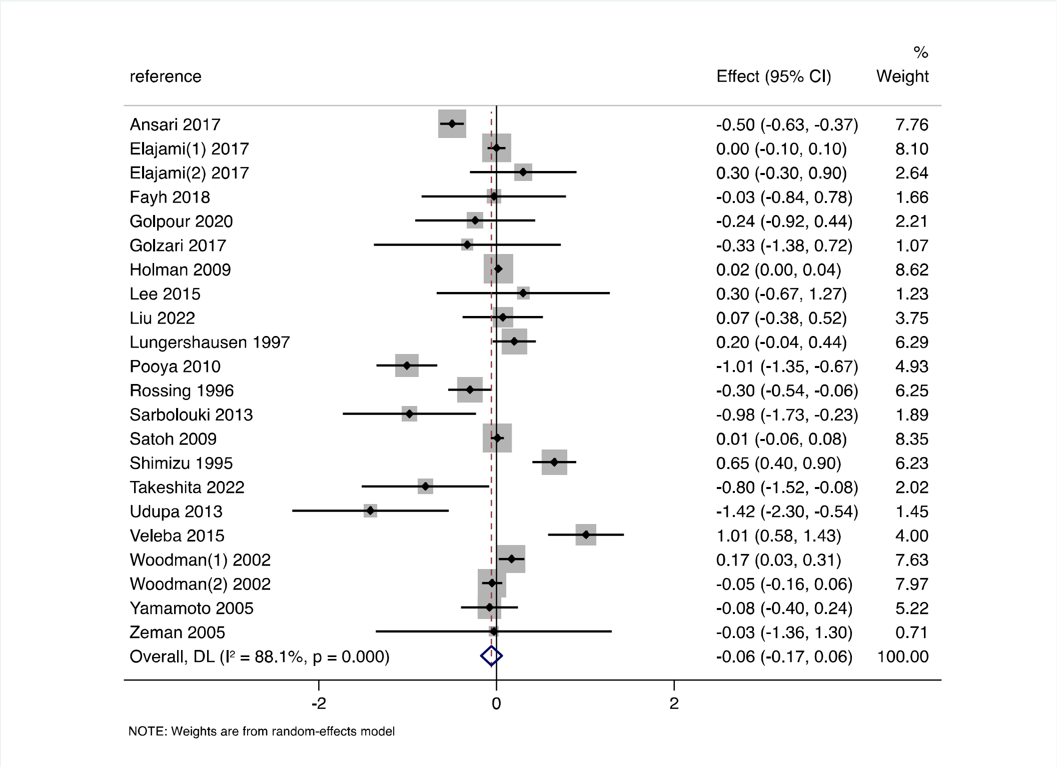

Figure S1-1-9 Forest plot of RCTs investigating the effect of n3 fatty acids supplementation on A1C.

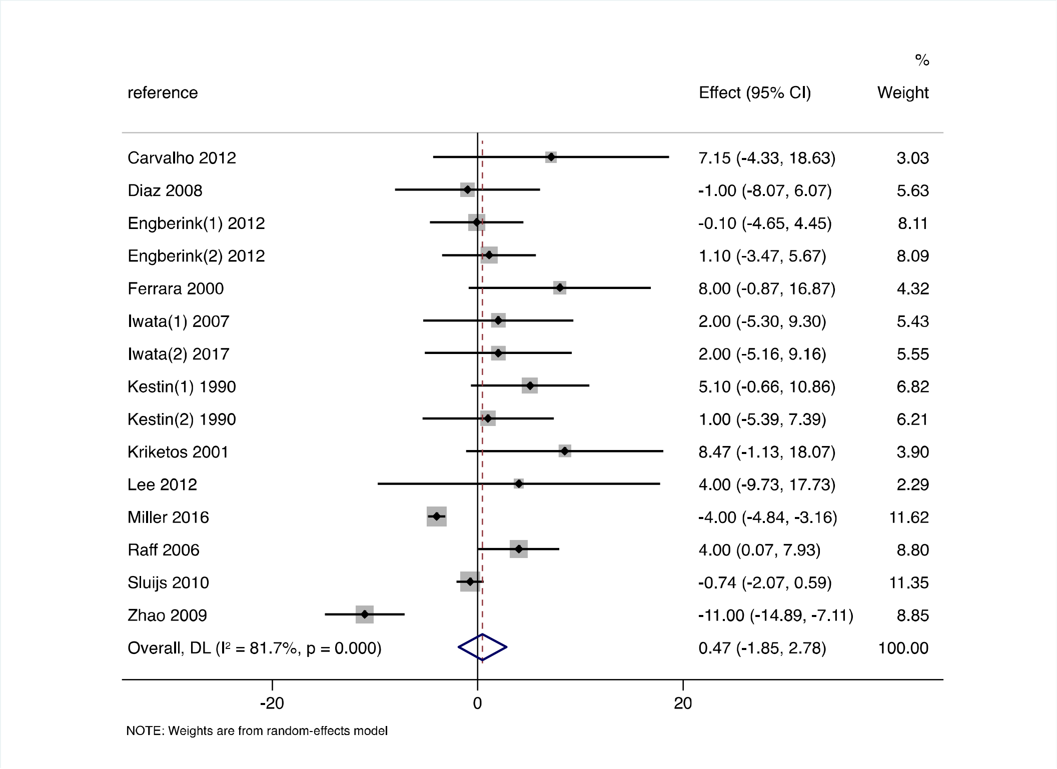

Figure S1-2-1 Forest plot of RCTs investigating the effect of n6 fatty acids supplementation on SBP.

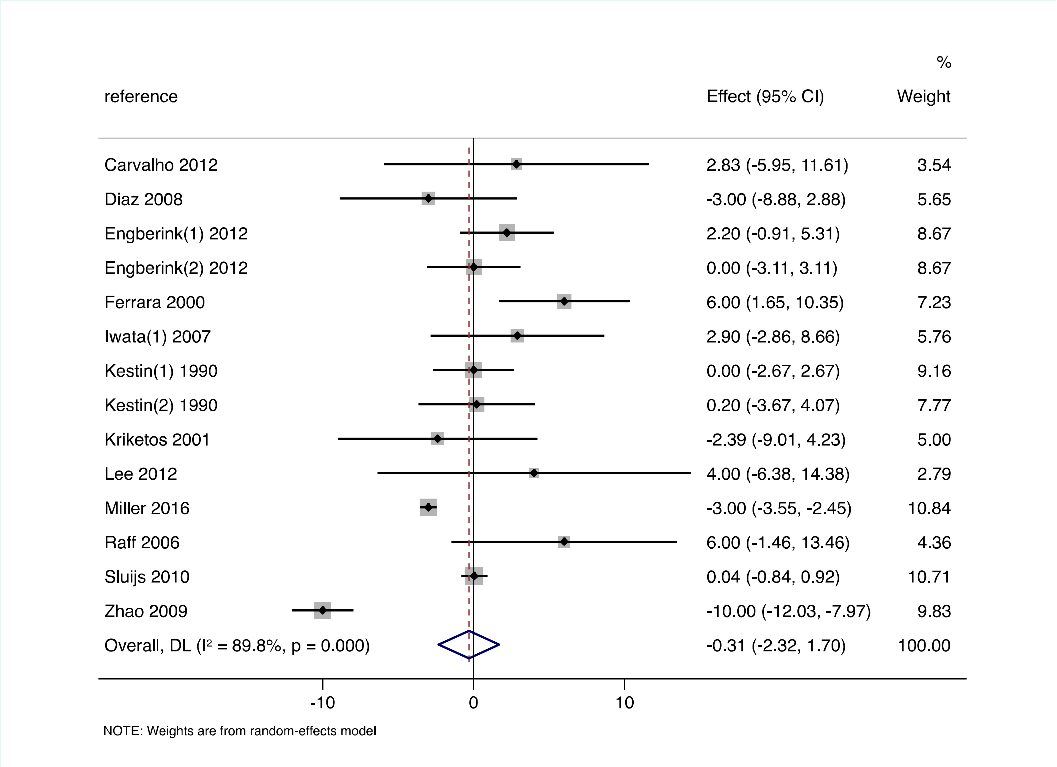

Figure S1-2-2 Forest plot of RCTs investigating the effect of n6 fatty acids supplementation on DBP.

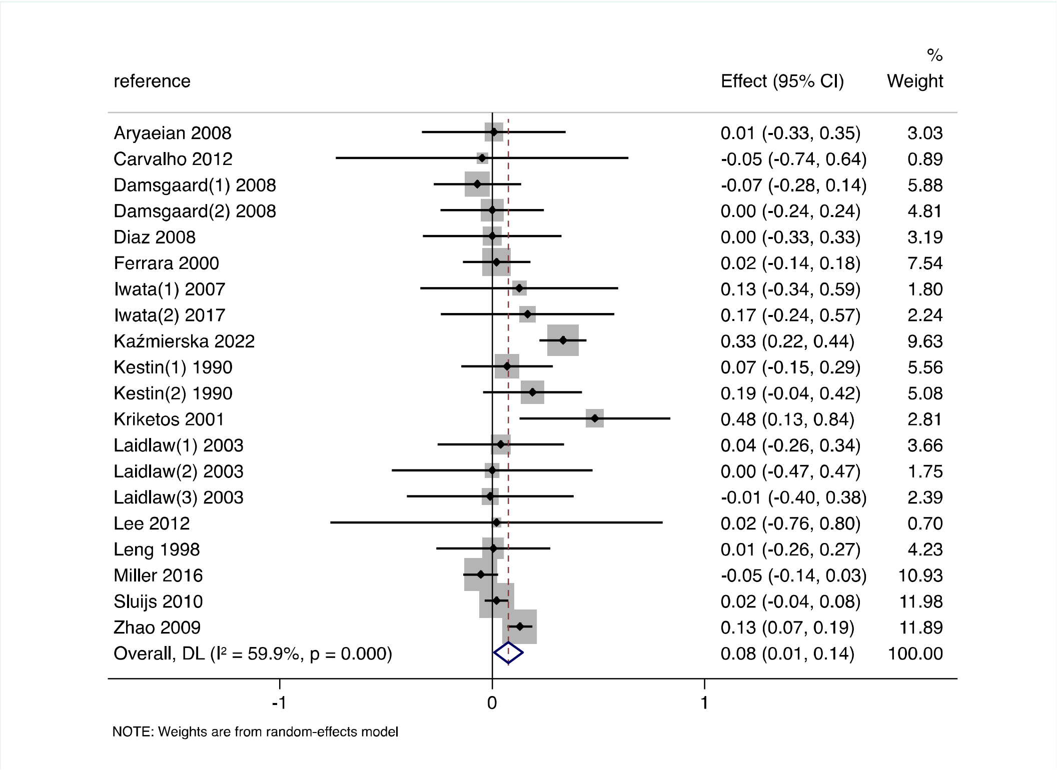

Figure S1-2-3 Forest plot of RCTs investigating the effect of n6 fatty acids supplementation on HDL-C.

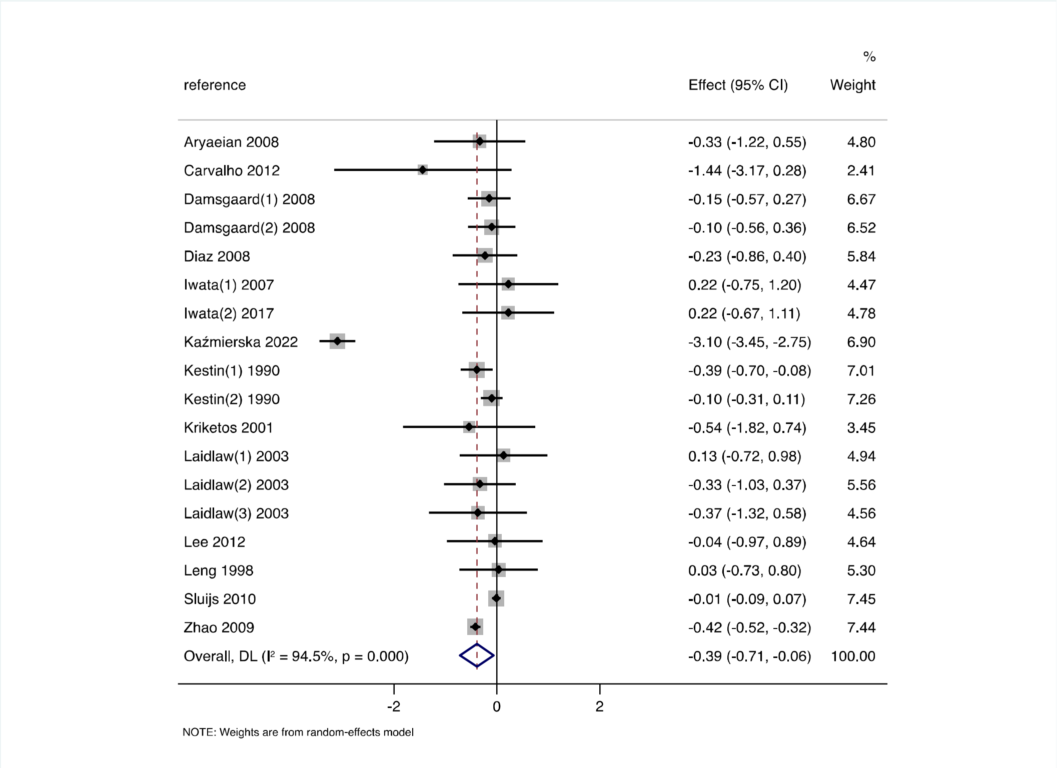

Figure S1-2-4 Forest plot of RCTs investigating the effect of n6 fatty acids supplementation on LDL-C.

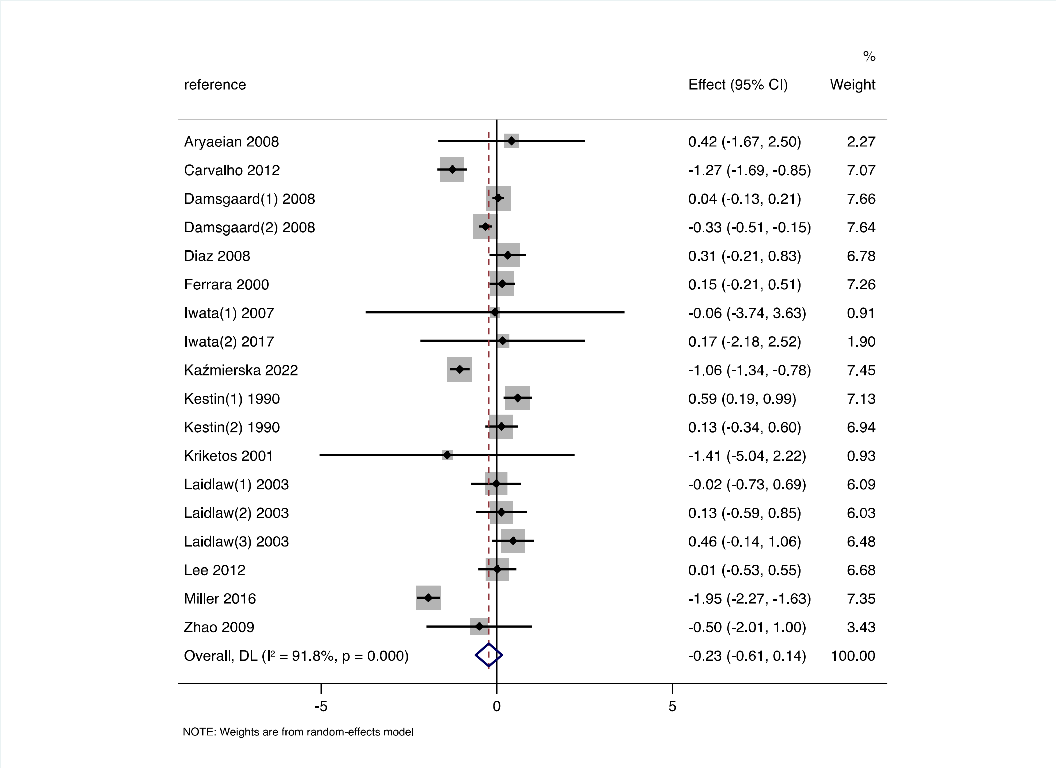

Figure S1-2-5 Forest plot of RCTs investigating the effect of n6 fatty acids supplementation on TG

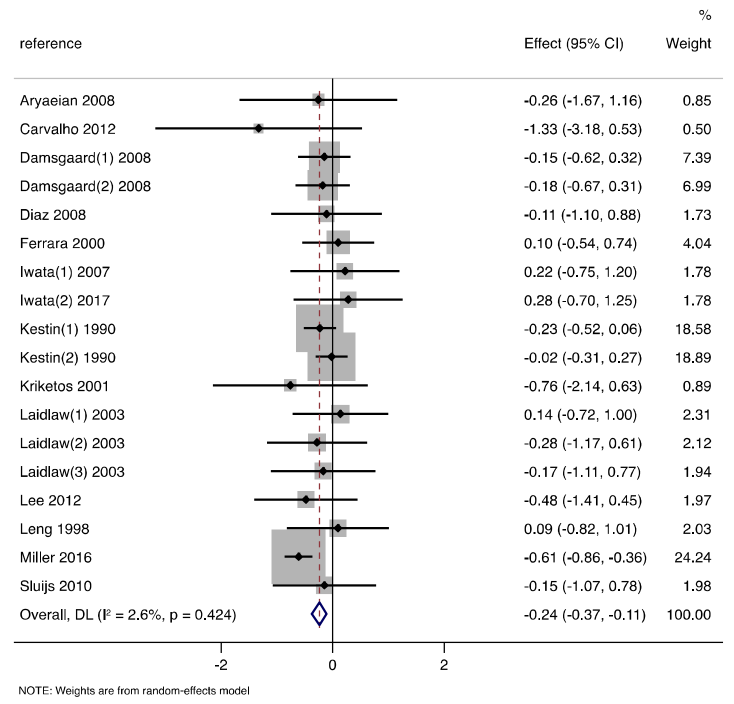

Figure S1-2-6 Forest plot of RCTs investigating the effect of n6 fatty acids supplementation on TC.

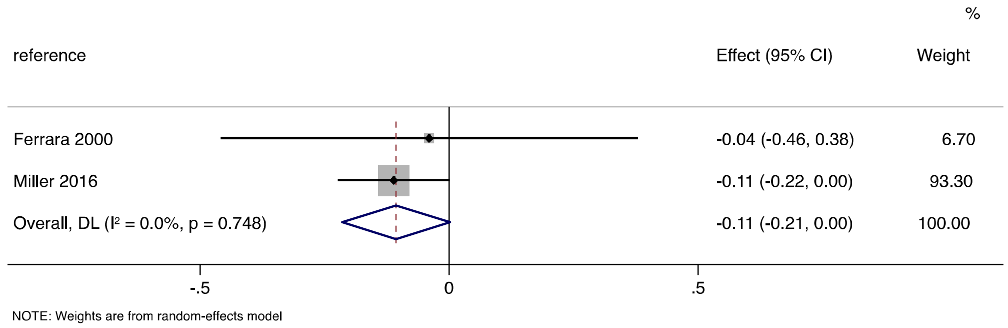

Figure S1-2-7 Forest plot of RCTs investigating the effect of n6 fatty acids supplementation on FBG.

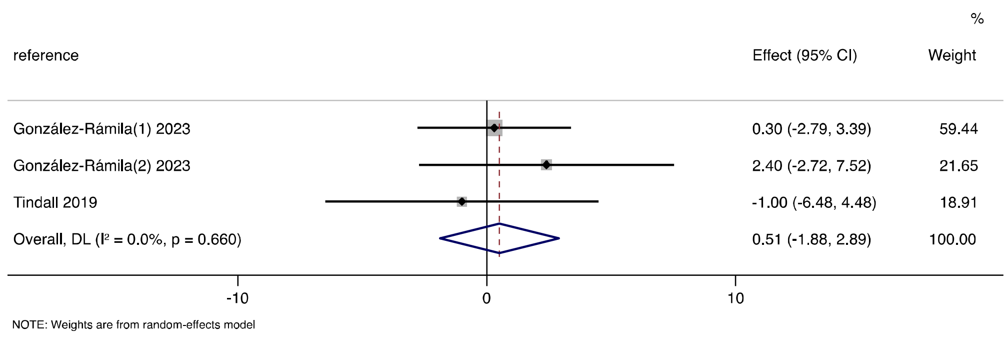

Figure S1-3-1 Forest plot of RCTs investigating the effect of n9 fatty acids supplementation on SBP.

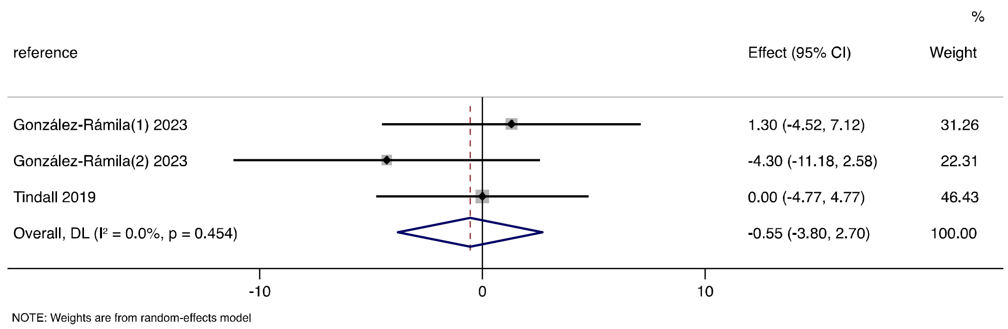

Figure S1-3-2 Forest plot of RCTs investigating the effect of n9 fatty acids supplementation on DBP.

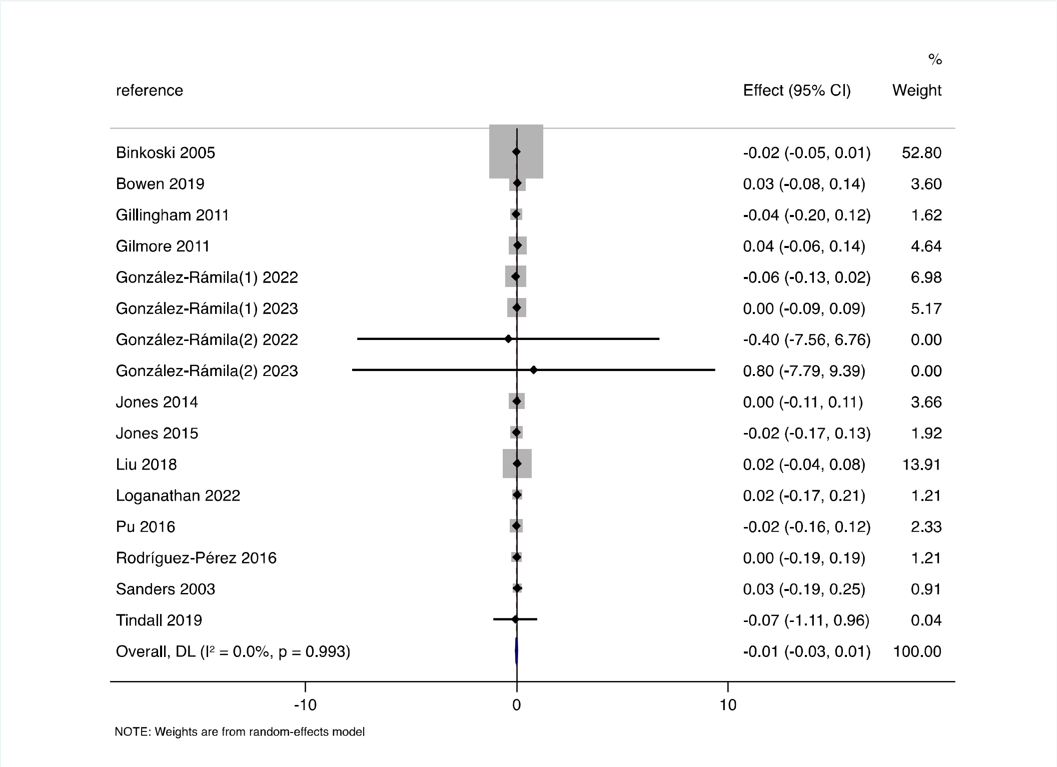

Figure S1-3-3 Forest plot of RCTs investigating the effect of n9 fatty acids supplementation on HDL-C.

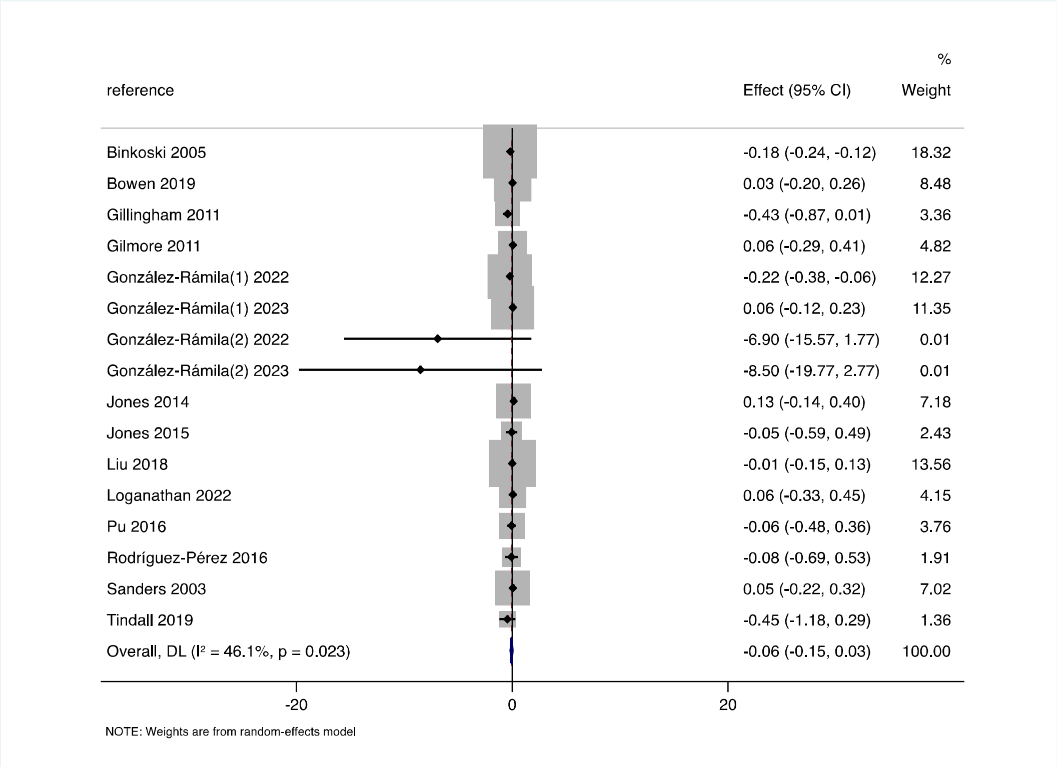

Figure S1-3-4 Forest plot of RCTs investigating the effect of n9 fatty acids supplementation on LDL-C.

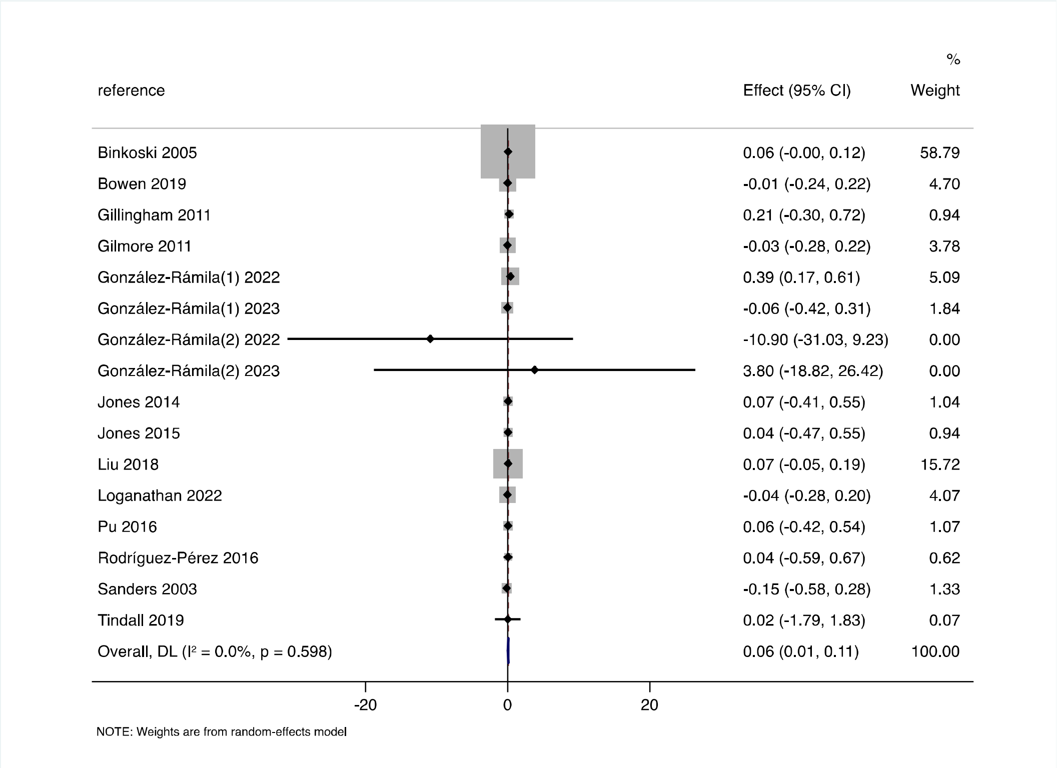

Figure S1-3-5 Forest plot of RCTs investigating the effect of n9 fatty acids supplementation on TG

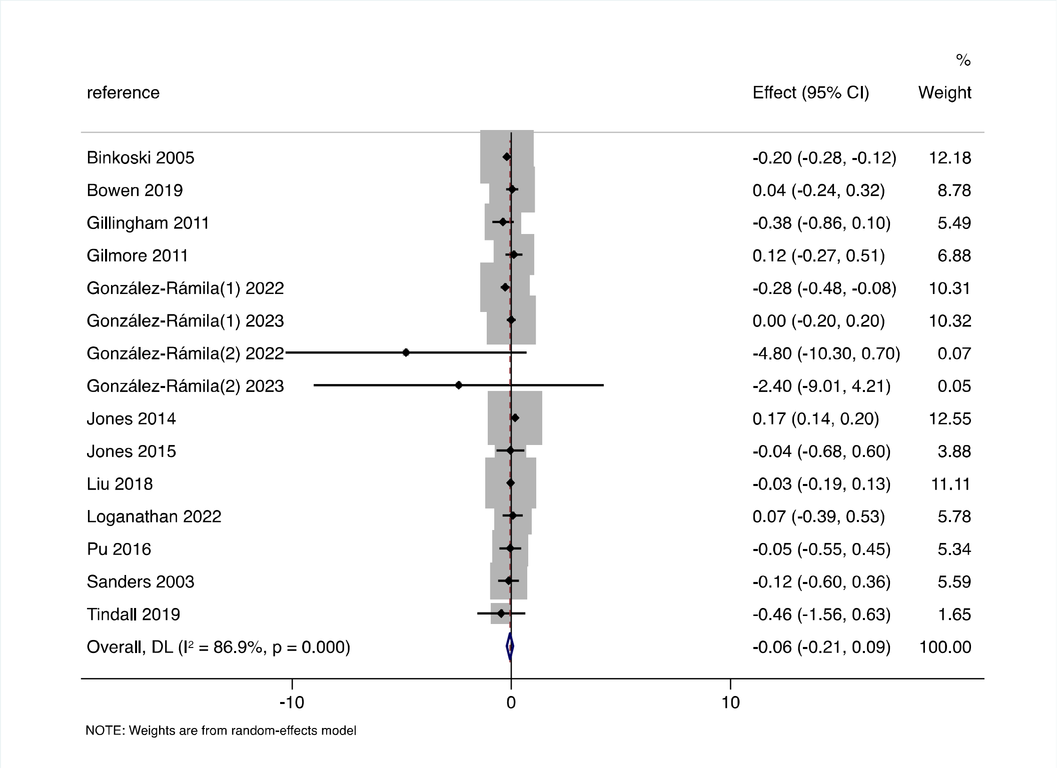

Figure S1-3-6 Forest plot of RCTs investigating the effect of n9 fatty acids supplementation on TC.

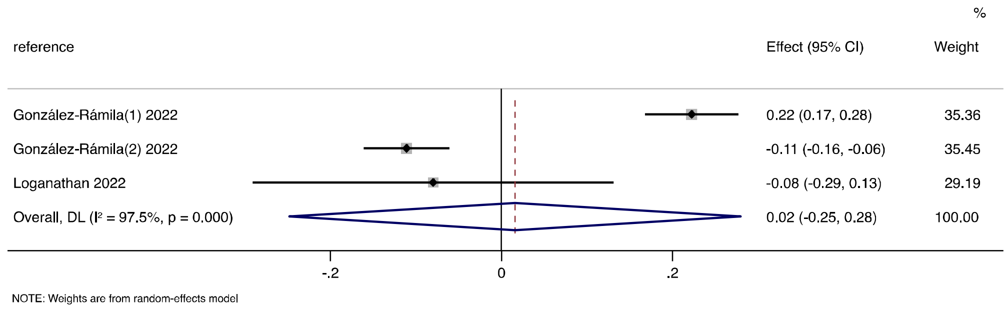

Figure S1-3-7 Forest plot of RCTs investigating the effect of n9 fatty acids supplementation on FBG.

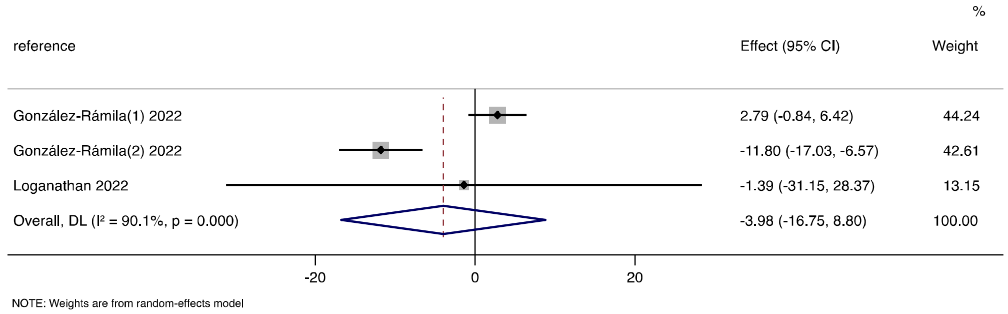

Figure S1-3-8 Forest plot of RCTs investigating the effect of n9 fatty acids supplementation on FBI.

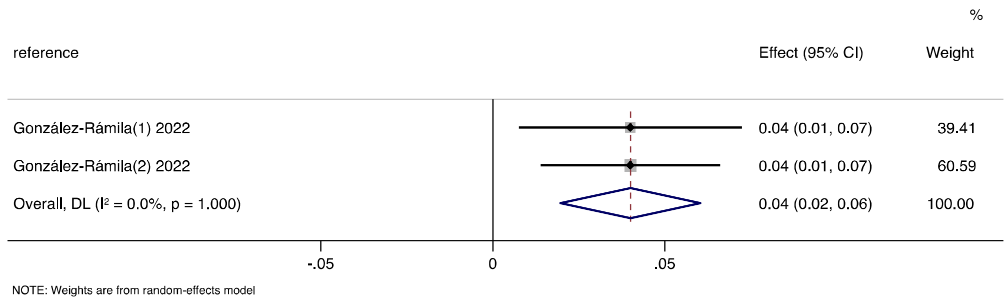

Figure S1-3-9 Forest plot of RCTs investigating the effect of n9 fatty acids supplementation on A1C.

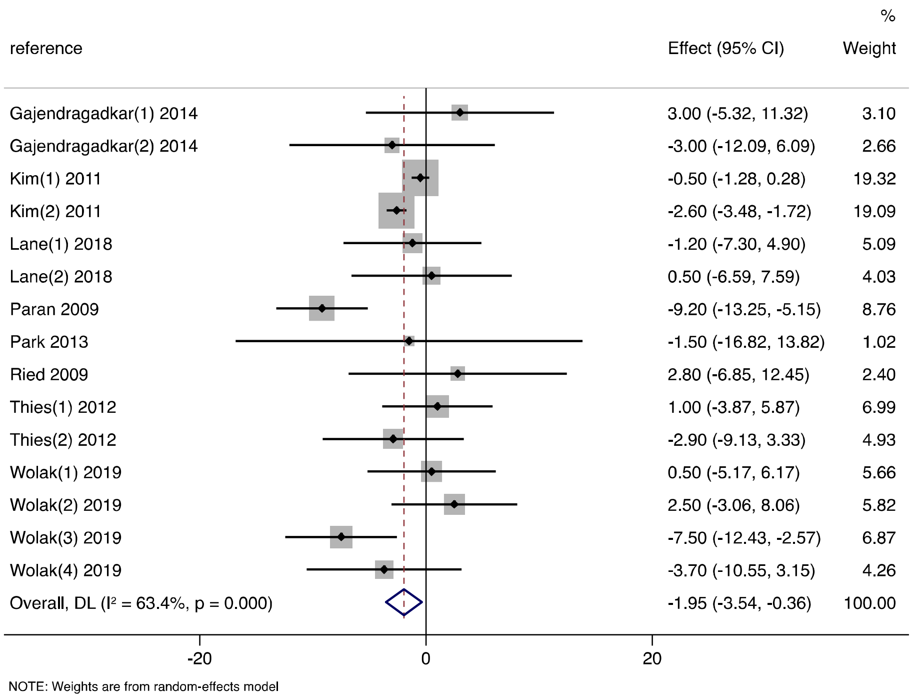

Figure S1-4-1 Forest plot of RCTs investigating the effect of lycopene supplementation on SBP.

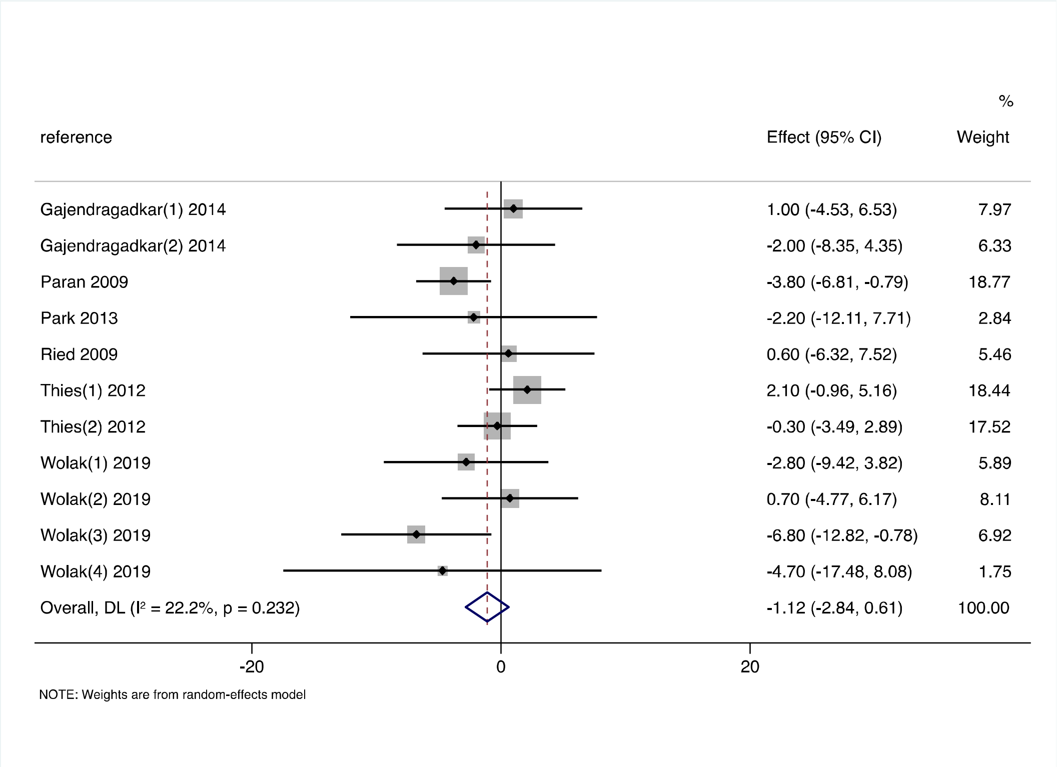

Figure S1-4-2 Forest plot of RCTs investigating the effect of lycopene supplementation on DBP.

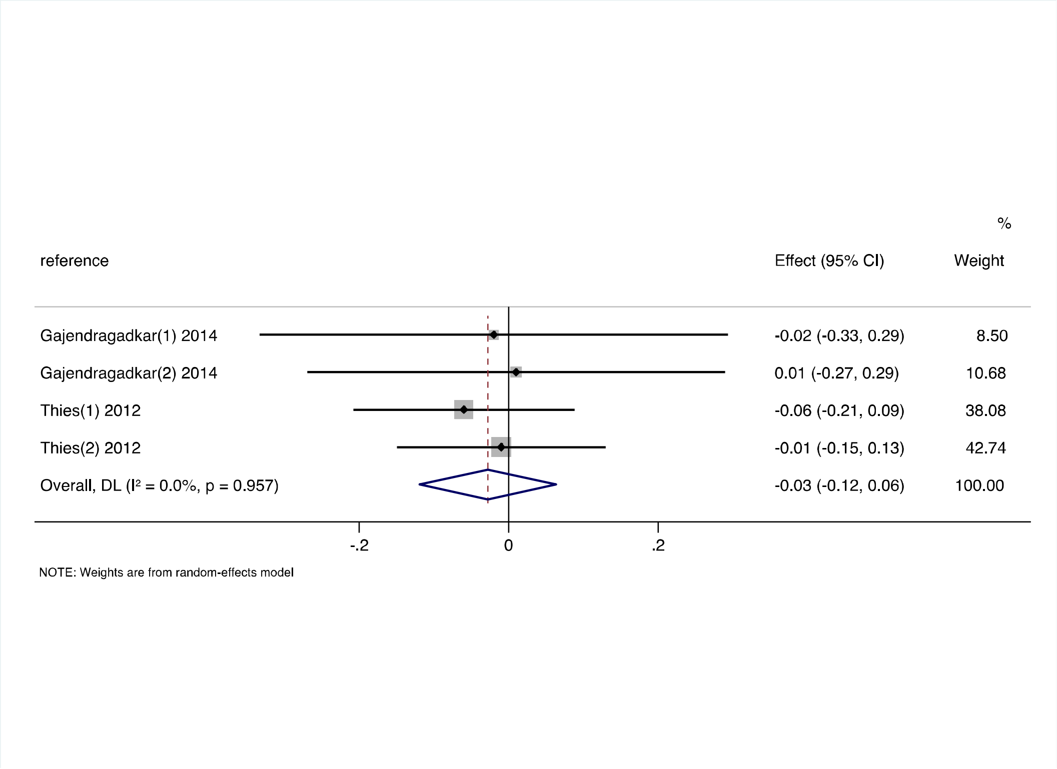

Figure S1-4-3 Forest plot of RCTs investigating the effect of lycopene supplementation on HDL-C.

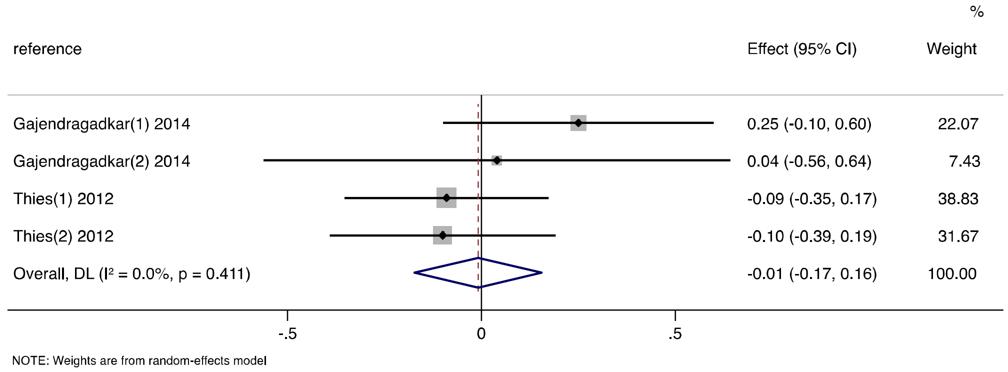

Figure S1-4-4 Forest plot of RCTs investigating the effect of lycopene supplementation on LDL-C.

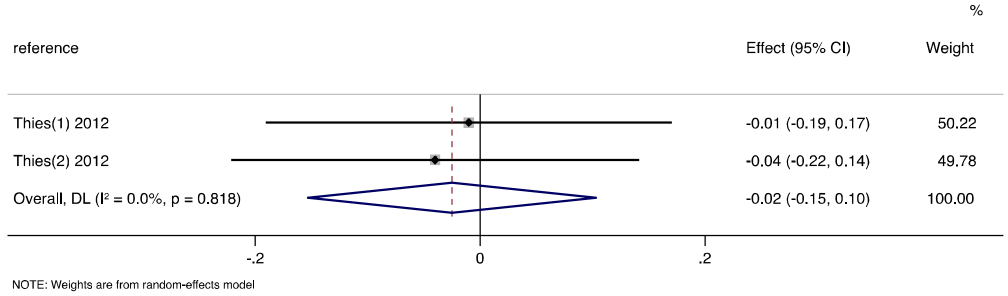

Figure S1-4-5 Forest plot of RCTs investigating the effect of lycopene supplementation on TG

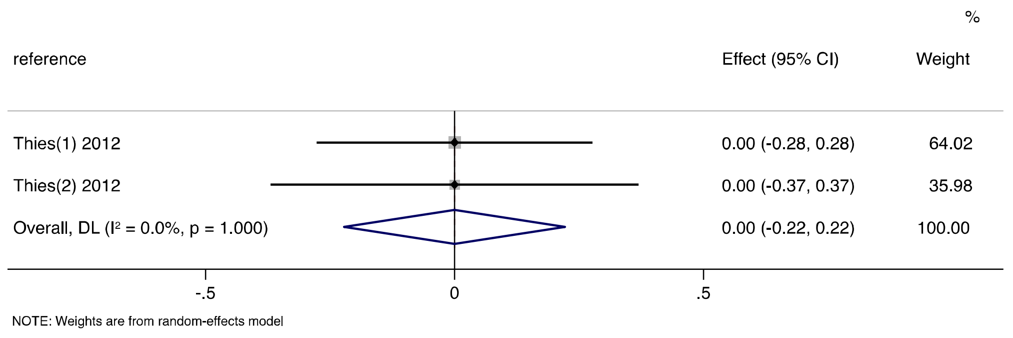

Figure S1-4-6 Forest plot of RCTs investigating the effect of lycopene supplementation on TC.

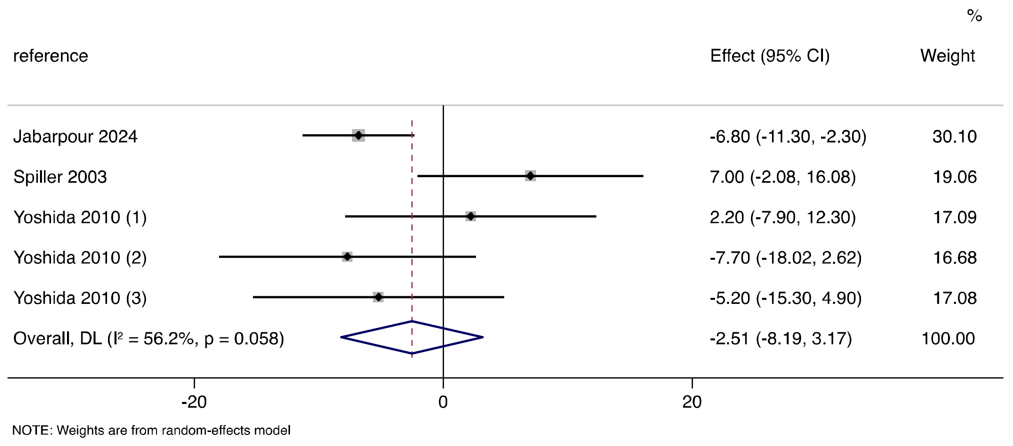

Figure S1-5-1 Forest plot of RCTs investigating the effect of astaxanthin supplementation on SBP.

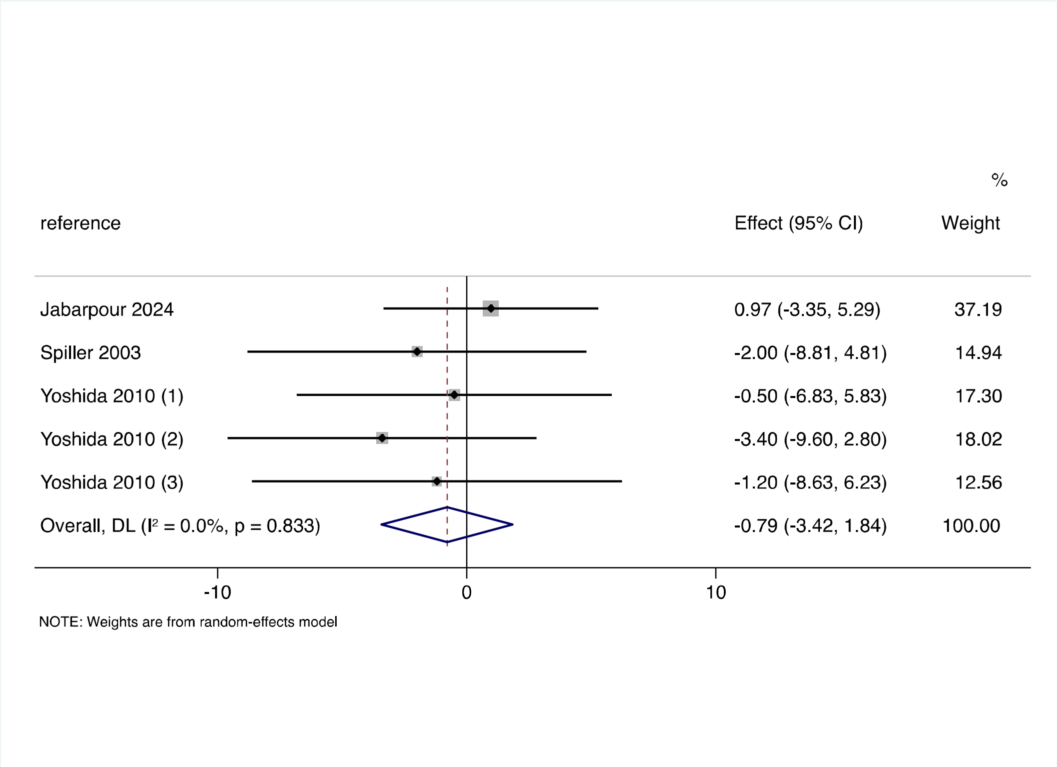

Figure S1-5-2 Forest plot of RCTs investigating the effect of astaxanthin supplementation on DBP.

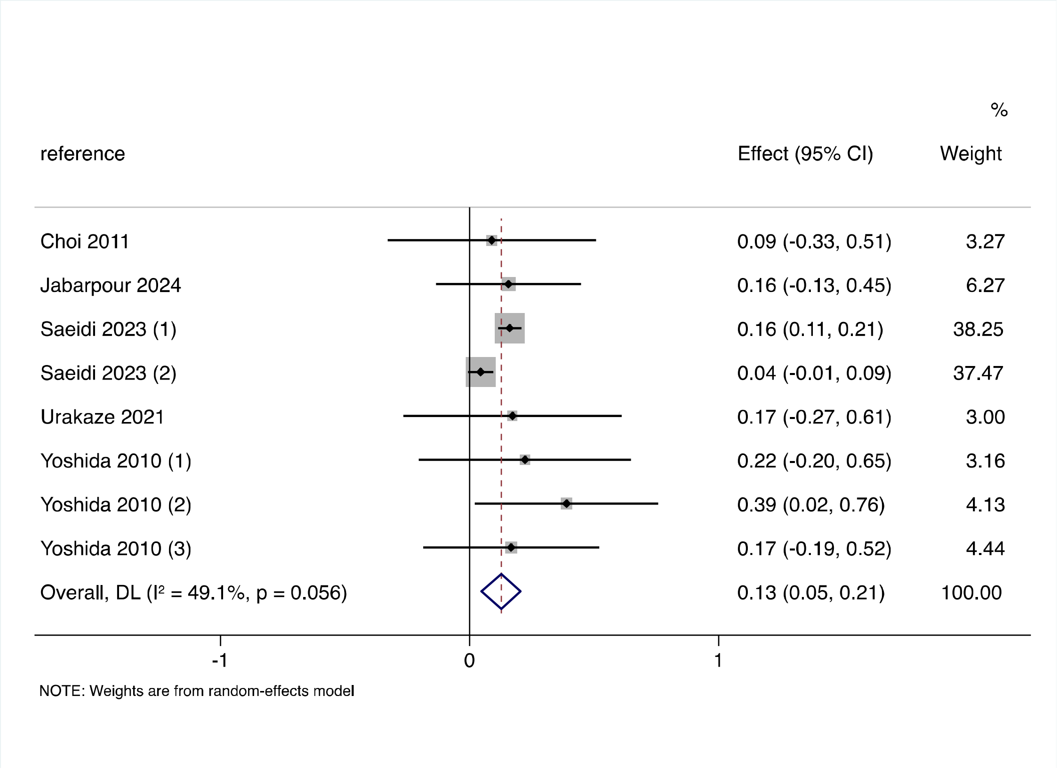

Figure S1-5-3 Forest plot of RCTs investigating the effect of astaxanthin supplementation on HDL-C.

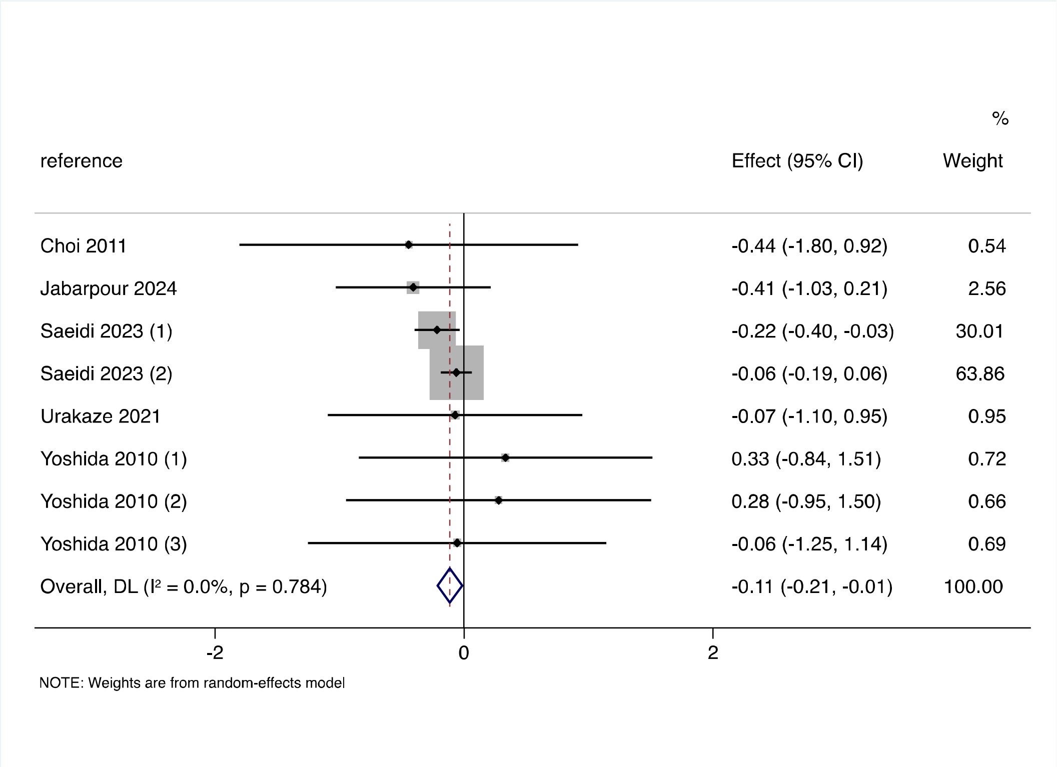

Figure S1-5-4 Forest plot of RCTs investigating the effect of astaxanthin supplementation on LDL-C.

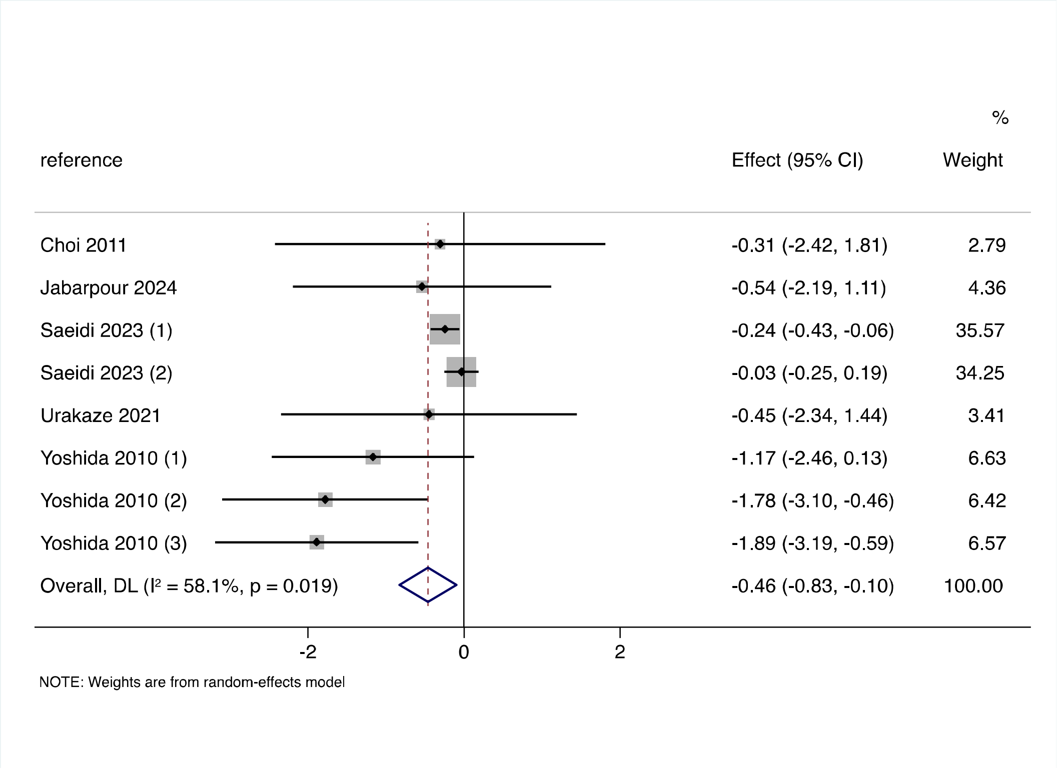

Figure S1-5-5 Forest plot of RCTs investigating the effect of astaxanthin supplementation on TG

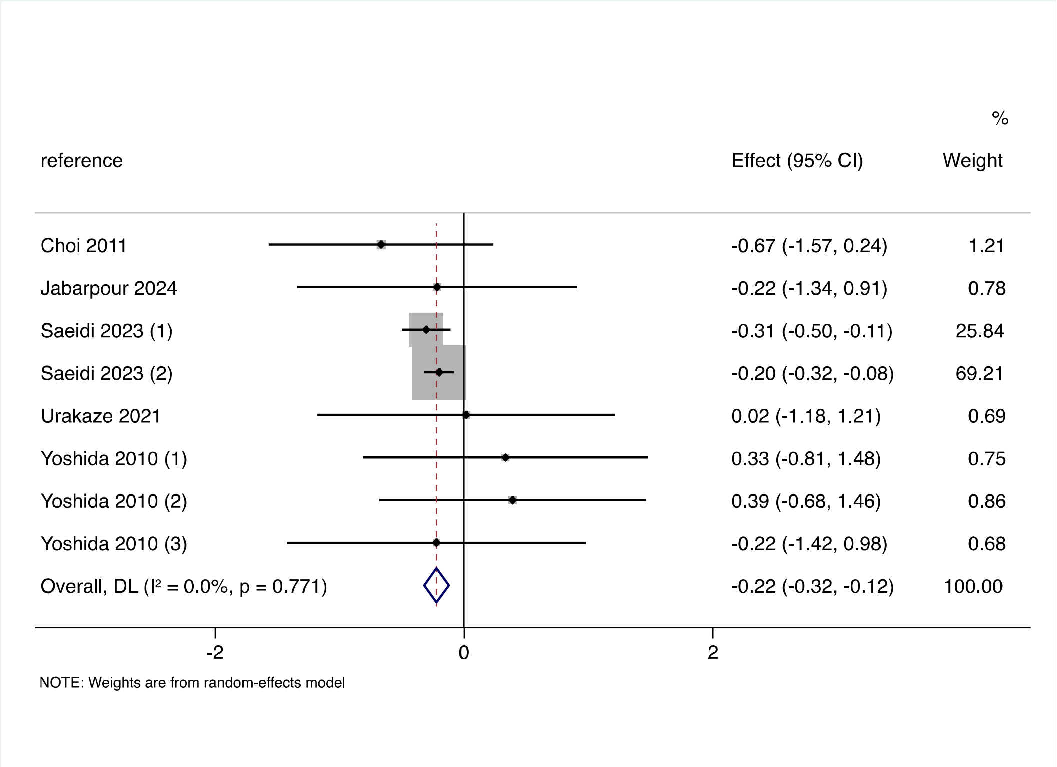

Figure S1-5-6 Forest plot of RCTs investigating the effect of astaxanthin supplementation on TC.

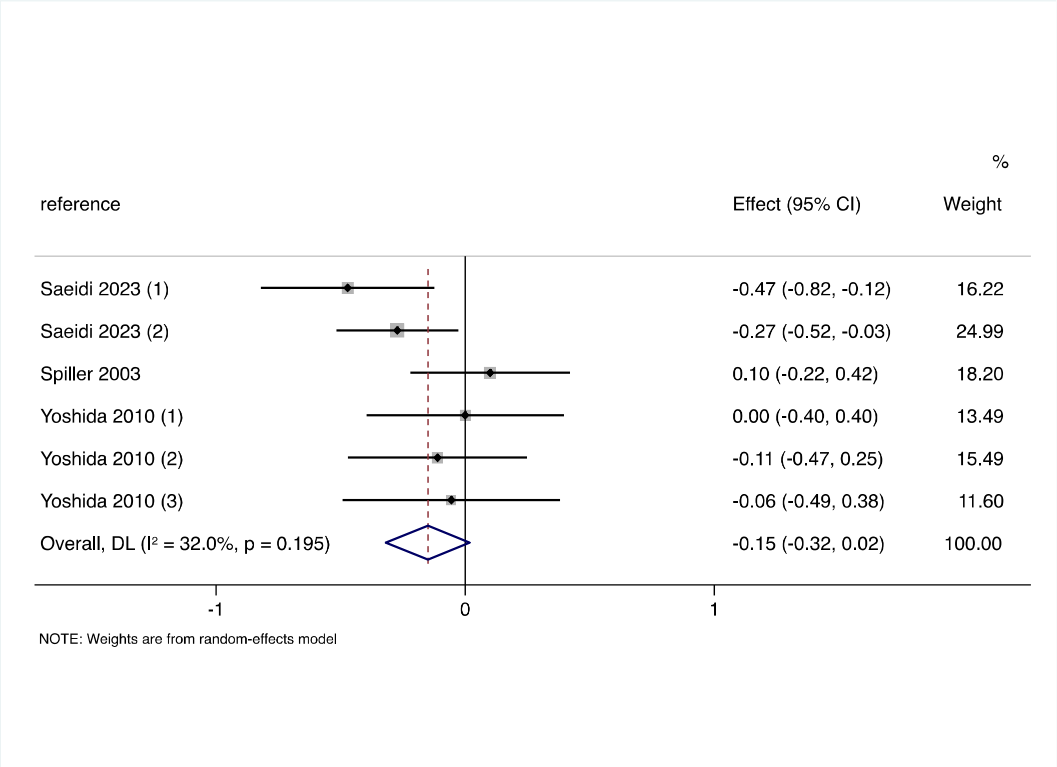

Figure S1-5-7 Forest plot of RCTs investigating the effect of astaxanthin supplementation on FBG.

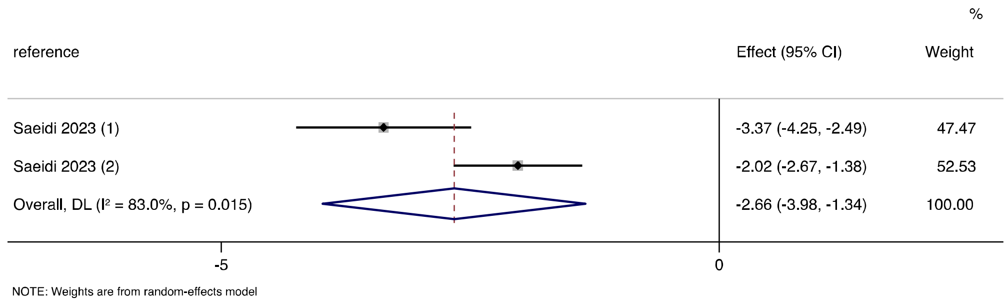

Figure S1-5-8 Forest plot of RCTs investigating the effect of astaxanthin supplementation on FBI.

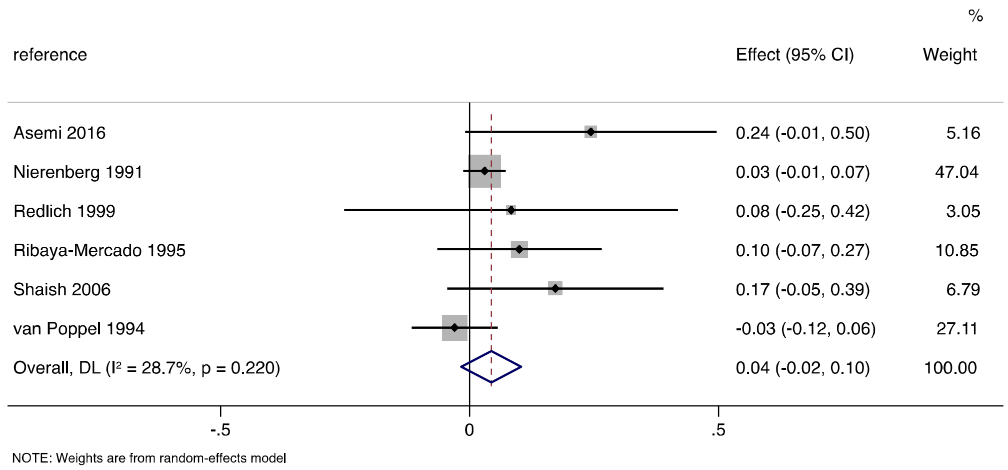

Figure S1-6-1 Forest plot of RCTs investigating the effect of beta-carotene supplementation on HDL-C.

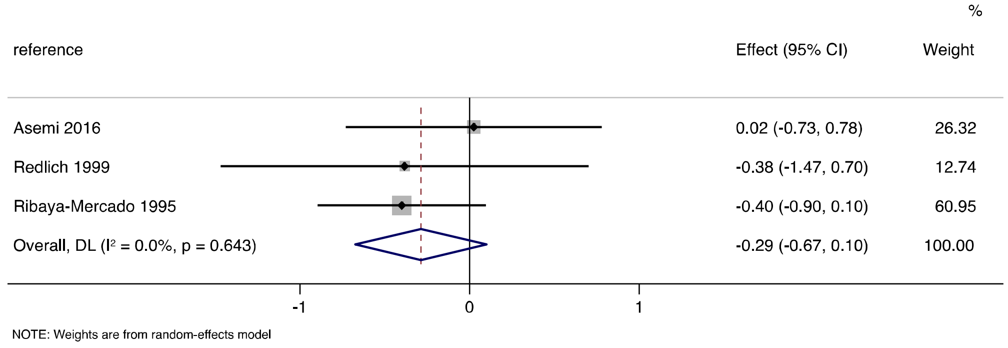

Figure S1-6-2 Forest plot of RCTs investigating the effect of beta-carotene supplementation on LDL-C.

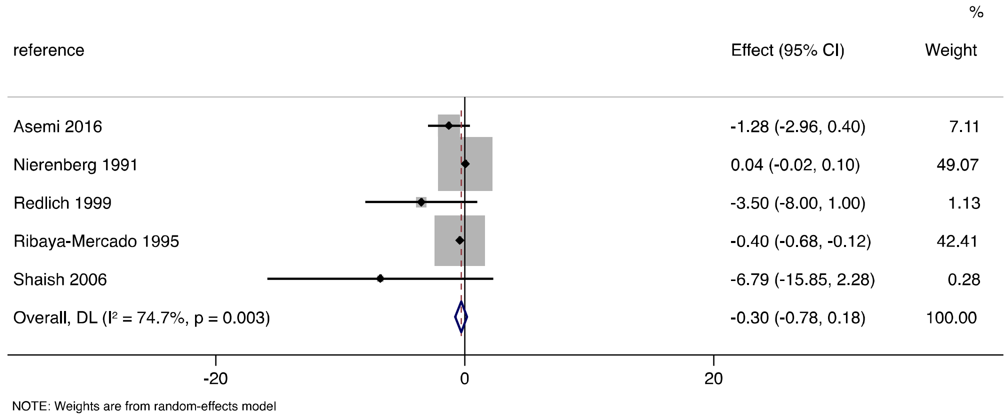

Figure S1-6-3 Forest plot of RCTs investigating the effect of beta-carotene supplementation on TG

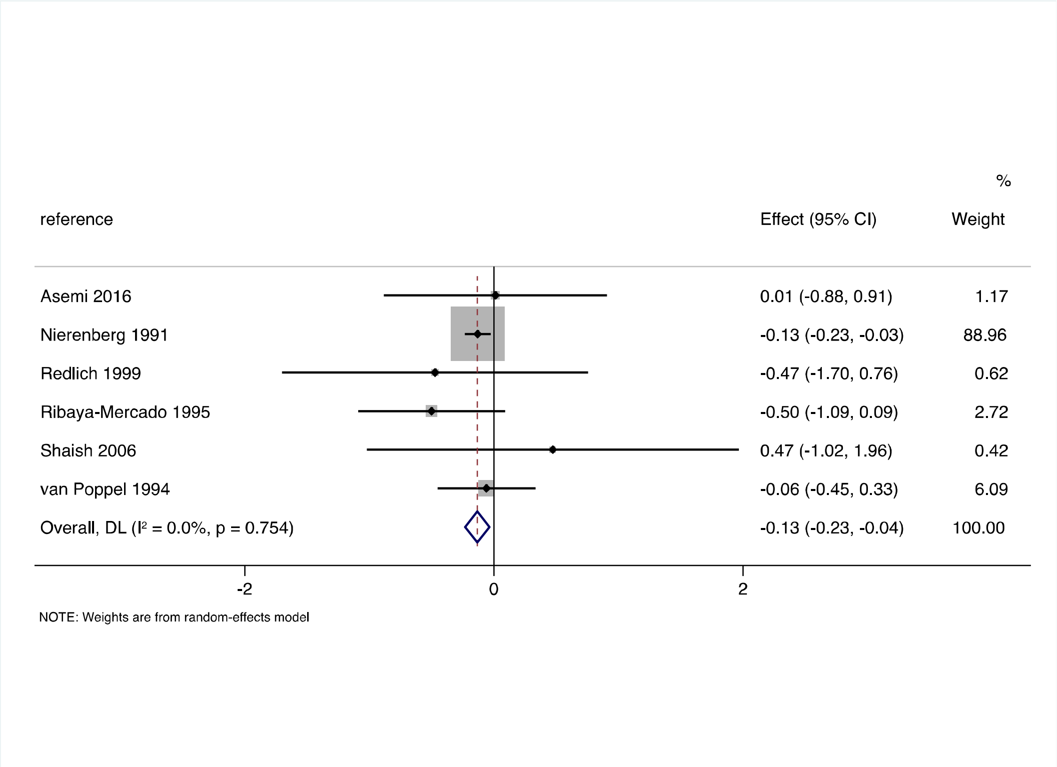

Figure S1-6-4 Forest plot of RCTs investigating the effect of beta-carotene supplementation on TC.

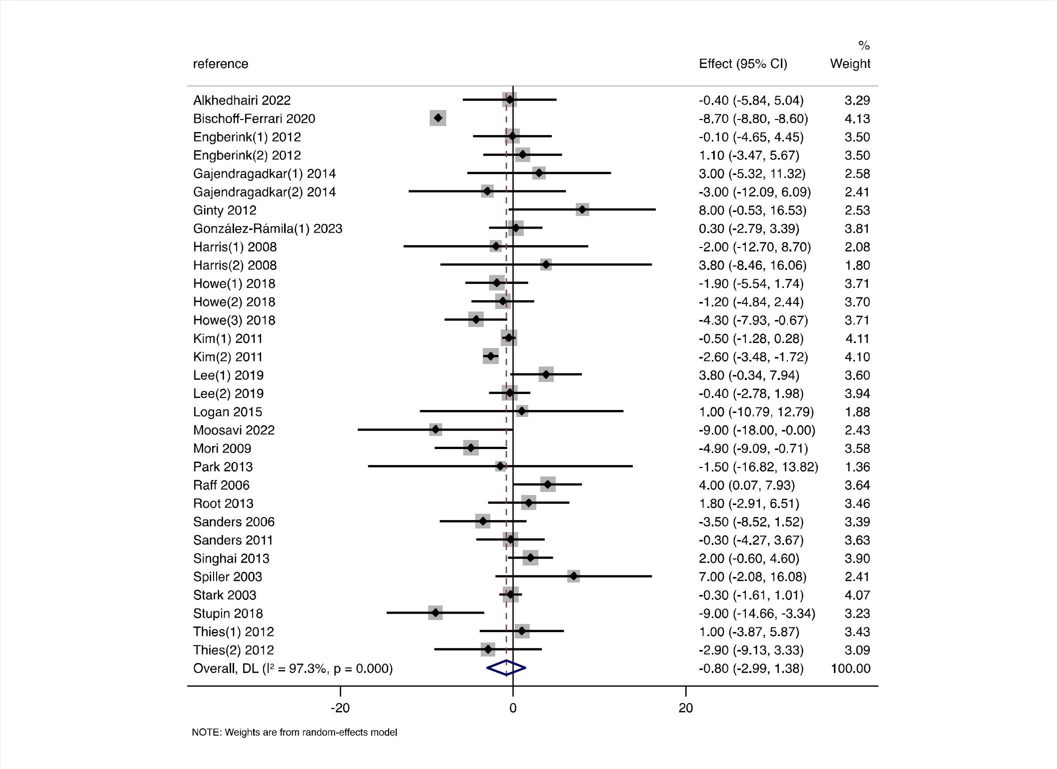

Figure S1-7-1 Forest plot of RCTs investigating the effect of antioxidant fatty acids supplementation on SBP in healthy population.

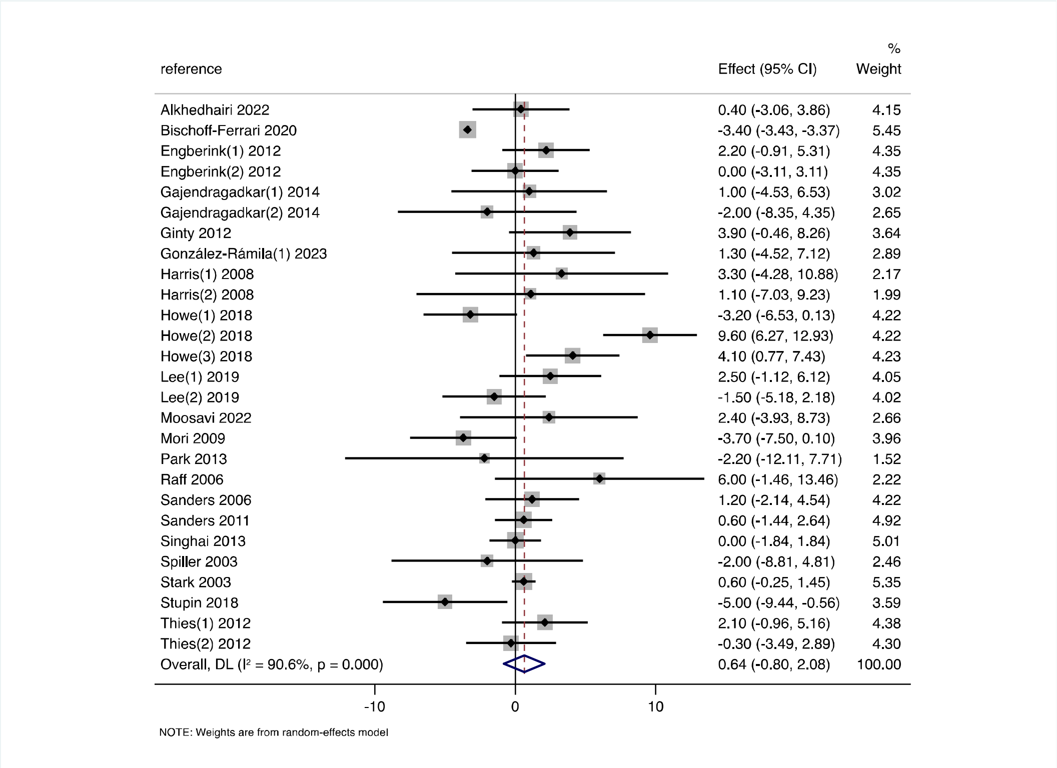

Figure S1-7-2 Forest plot of RCTs investigating the effect of antioxidant fatty acids supplementation on DBP in healthy population.

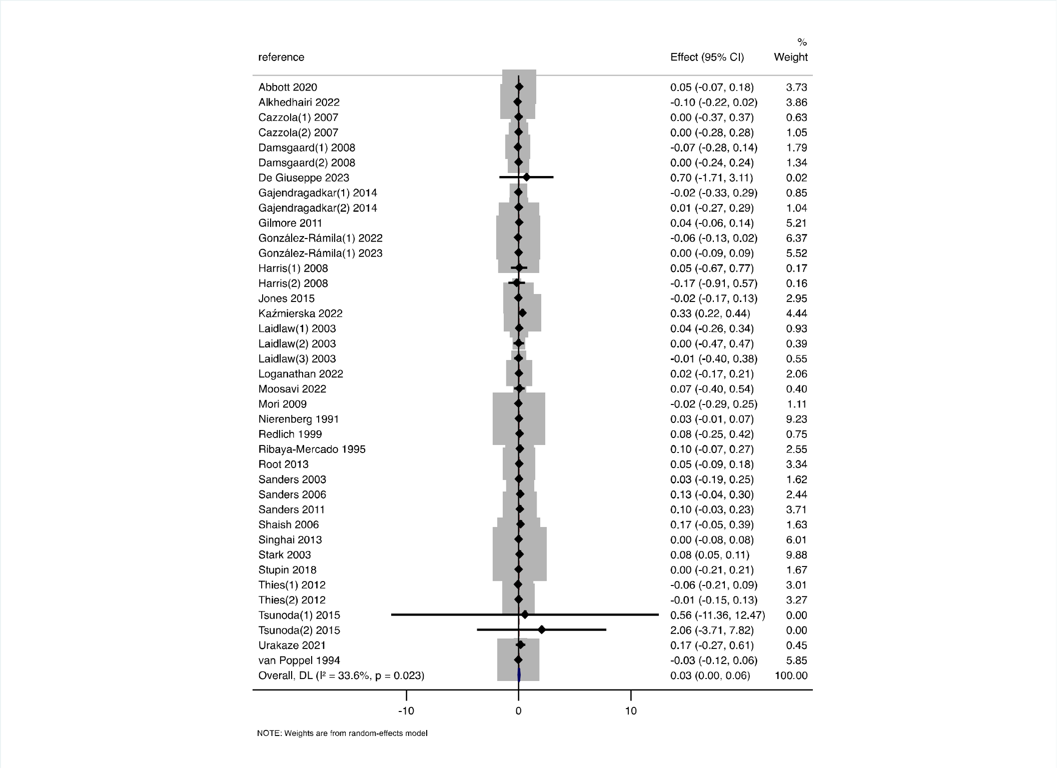

Figure S1-7-3 Forest plot of RCTs investigating the effect of antioxidant fatty acids supplementation on HDL in healthy population.

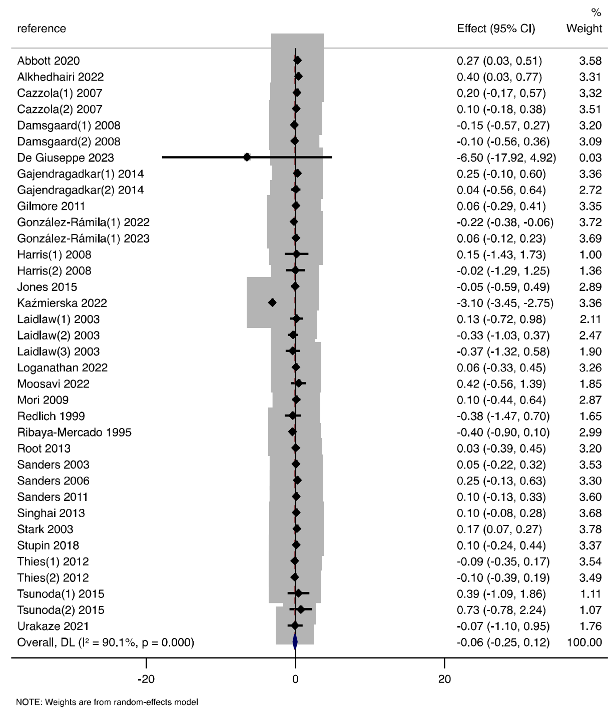

Figure S1-7-4 Forest plot of RCTs investigating the effect of antioxidant fatty acids supplementation on LDL in healthy population.

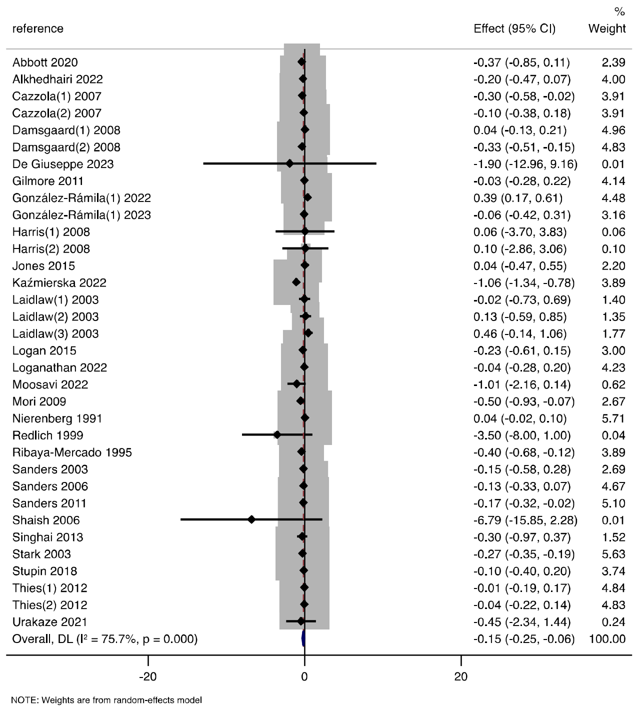

Figure S1-7-5 Forest plot of RCTs investigating the effect of antioxidant fatty acids supplementation on TG in healthy population.

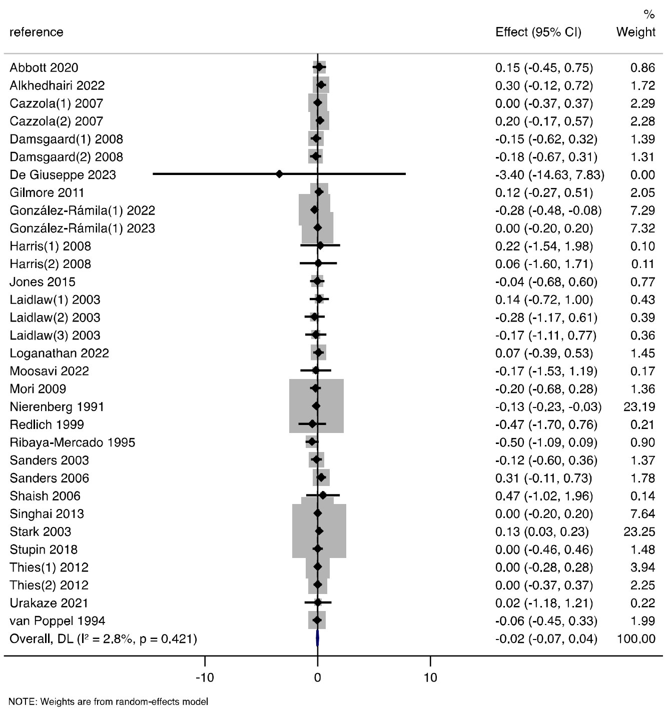

Figure S1-7-6 Forest plot of RCTs investigating the effect of antioxidant fatty acids supplementation on TC in healthy population.

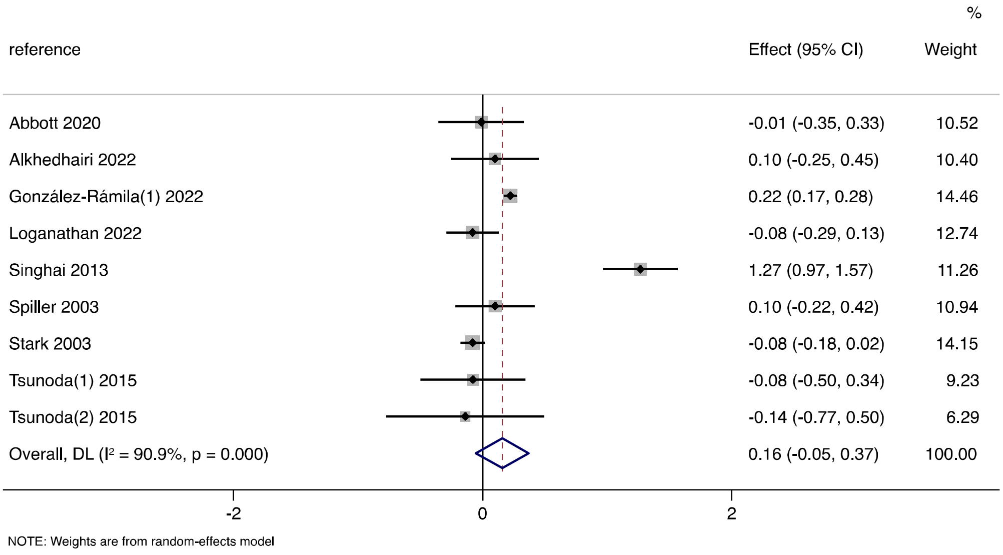

Figure S1-7-7 Forest plot of RCTs investigating the effect of antioxidant fatty acids supplementation on FBG in healthy population.

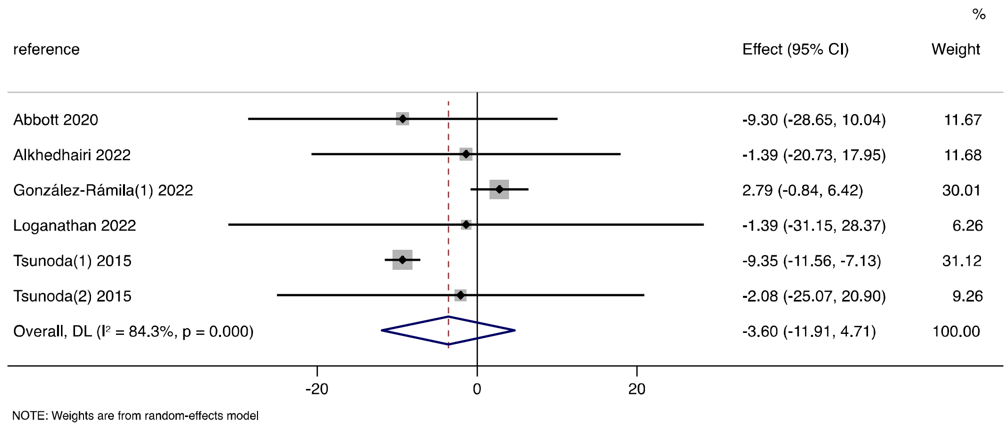

Figure S1-7-8 Forest plot of RCTs investigating the effect of antioxidant fatty acids supplementation on FBI in healthy population.

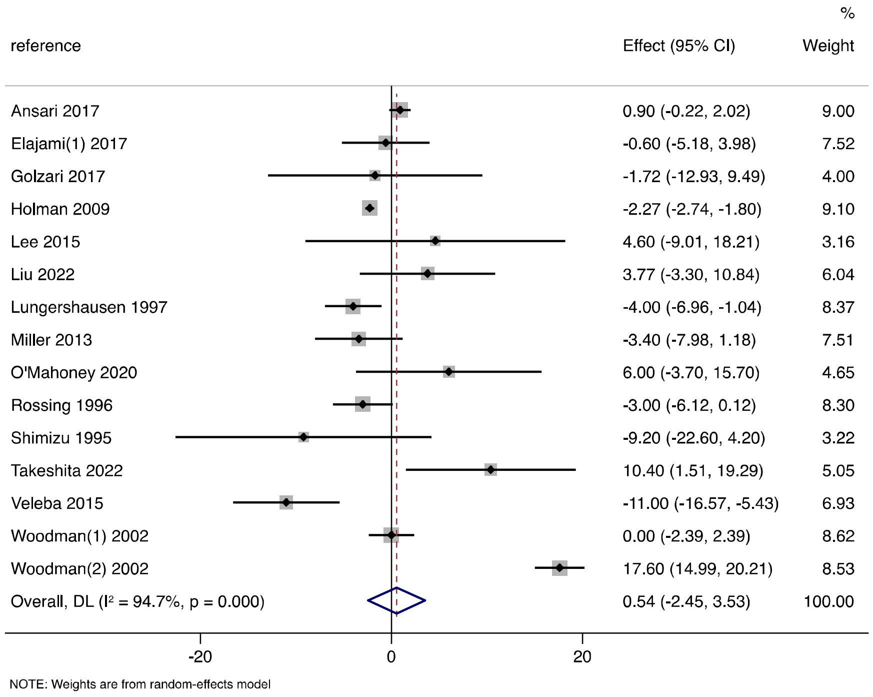

Figure S1-8-1 Forest plot of RCTs investigating the effect of antioxidant fatty acids supplementation on SBP in population with diabetes.

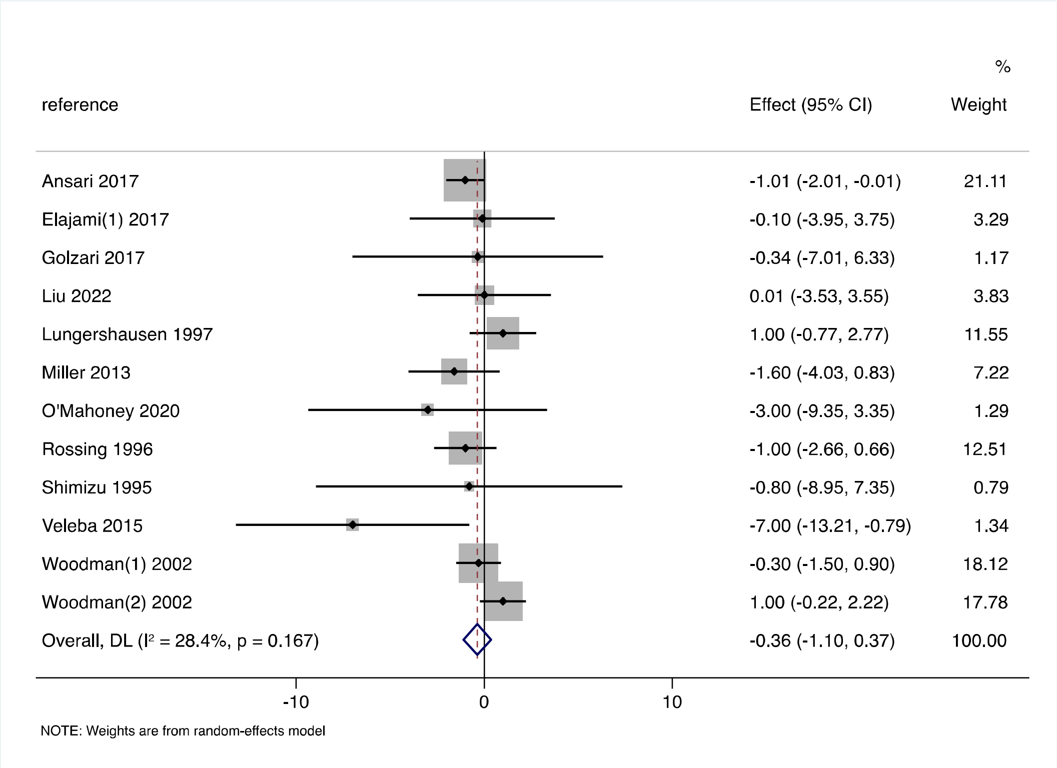

Figure S1-8-2 Forest plot of RCTs investigating the effect of antioxidant fatty acids supplementation on DBP in population with diabetes.

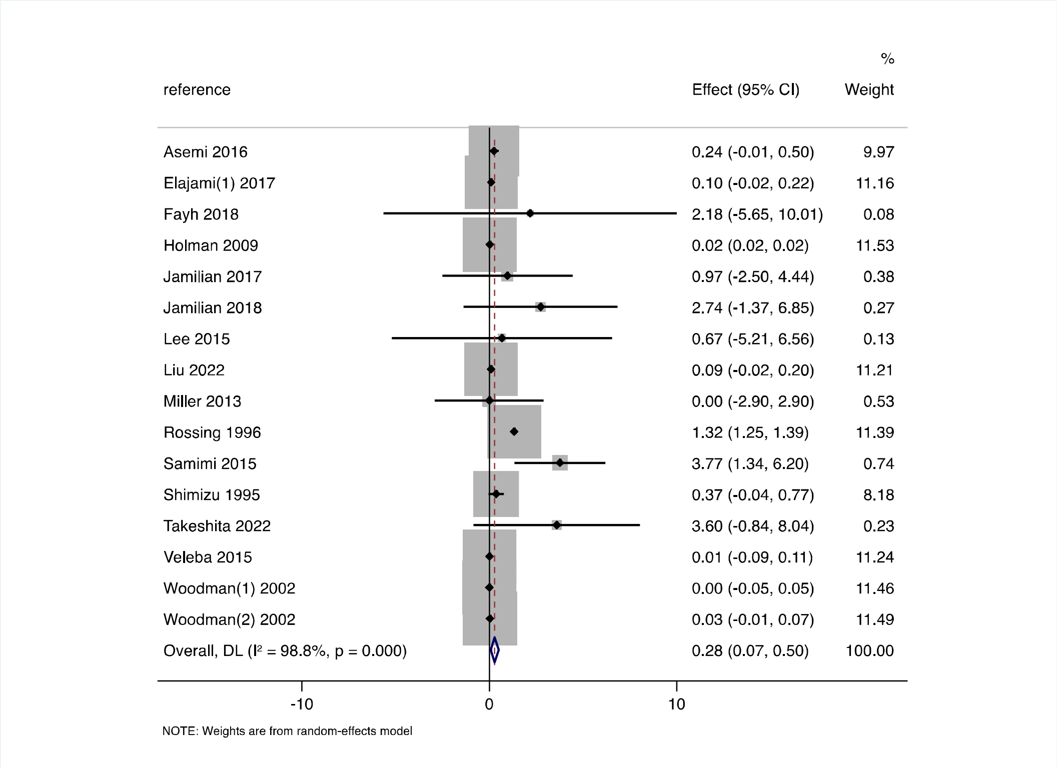

Figure S1-8-3 Forest plot of RCTs investigating the effect of antioxidant fatty acids supplementation on HDL in population with diabetes.

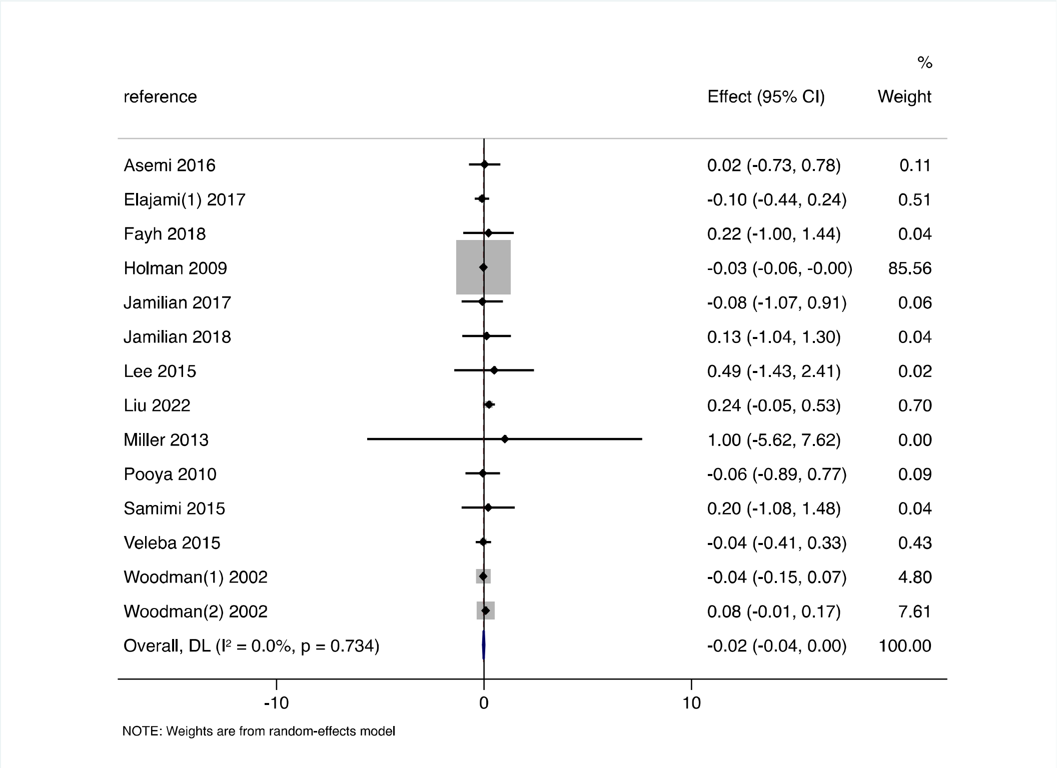

Figure S1-8-4 Forest plot of RCTs investigating the effect of antioxidant fatty acids supplementation on LDL in population with diabetes.

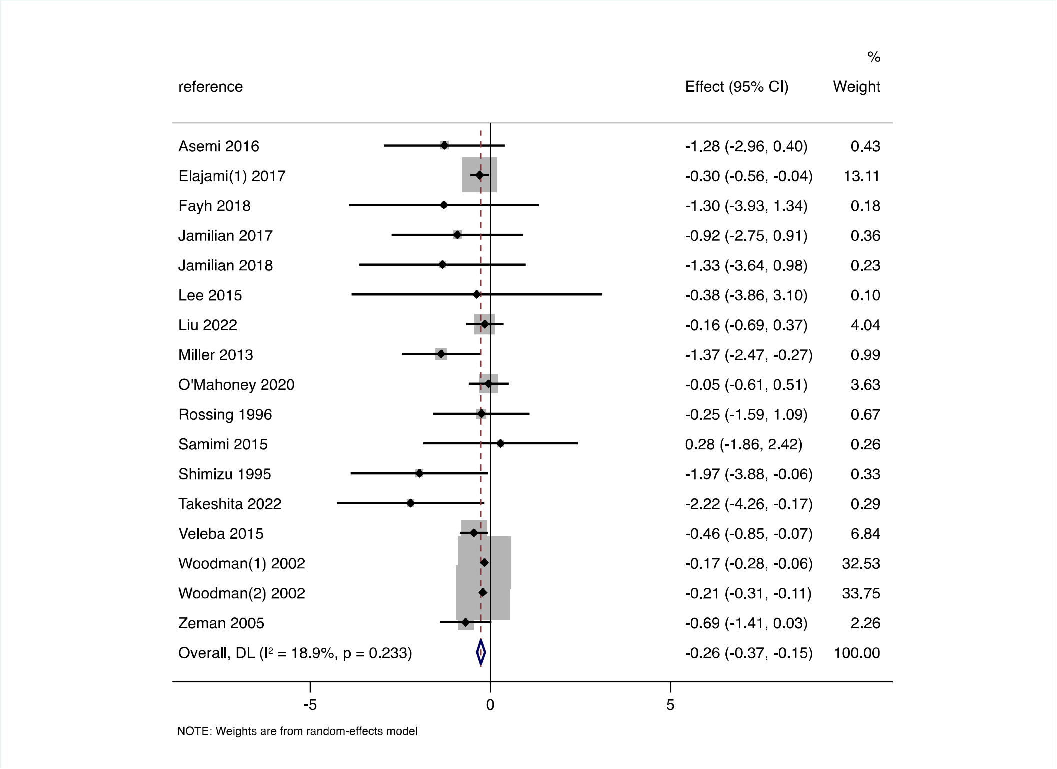

Figure S1-8-5 Forest plot of RCTs investigating the effect of antioxidant fatty acids supplementation on TG in population with diabetes.

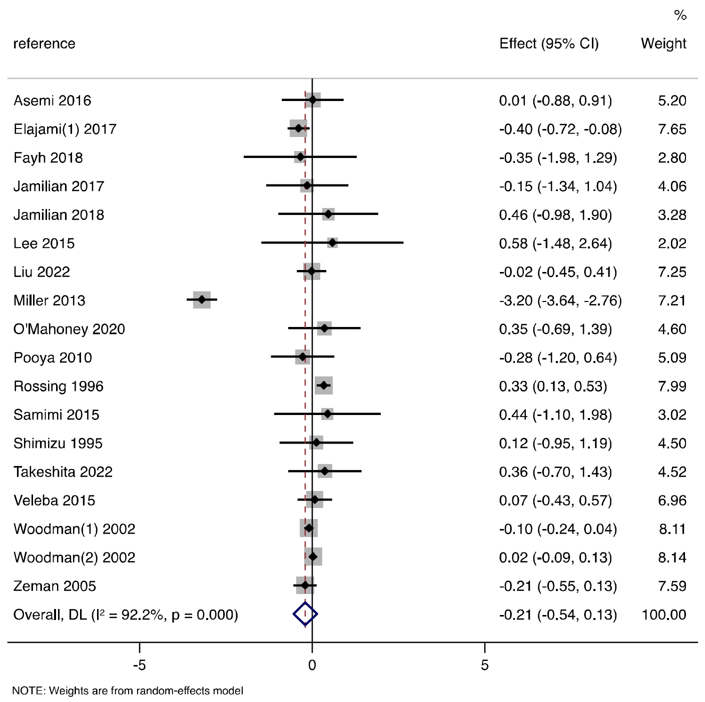

Figure S1-8-6 Forest plot of RCTs investigating the effect of antioxidant fatty acids supplementation on TC in population with diabetes.

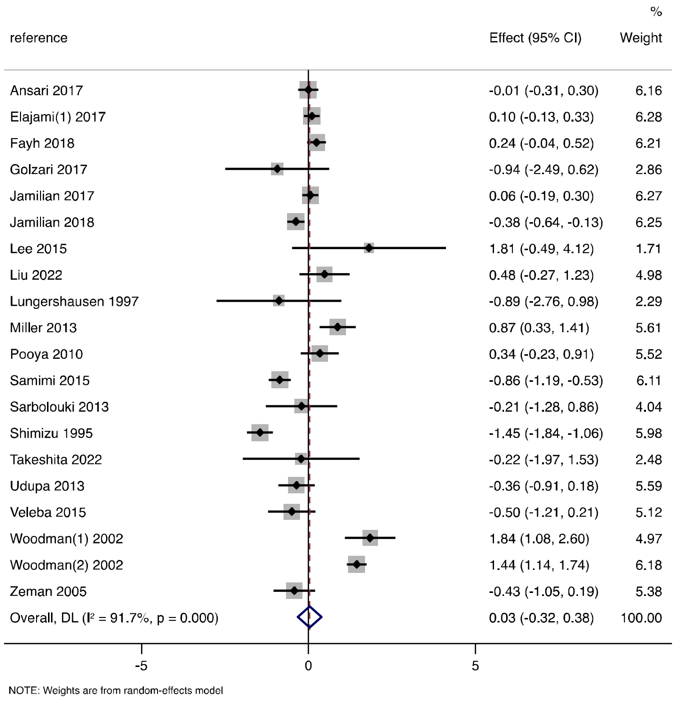

Figure S1-8-7 Forest plot of RCTs investigating the effect of antioxidant fatty acids supplementation on FBG in population with diabetes.

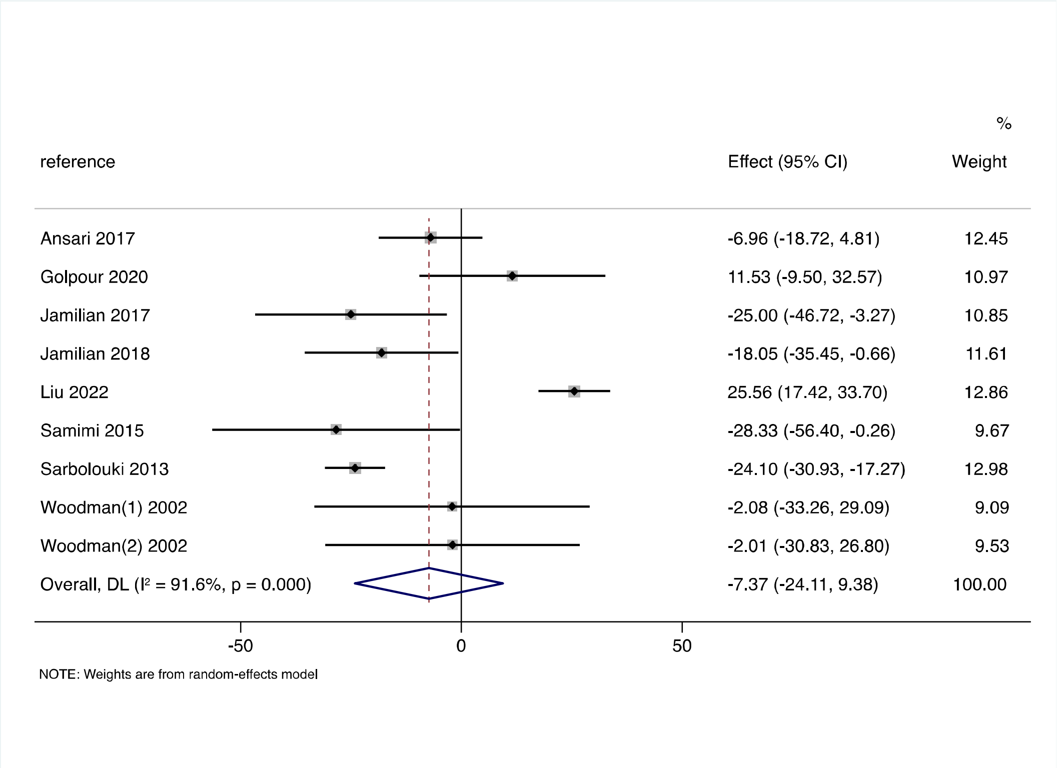

Figure S1-8-8 Forest plot of RCTs investigating the effect of antioxidant fatty acids supplementation on FBI in population with diabetes.

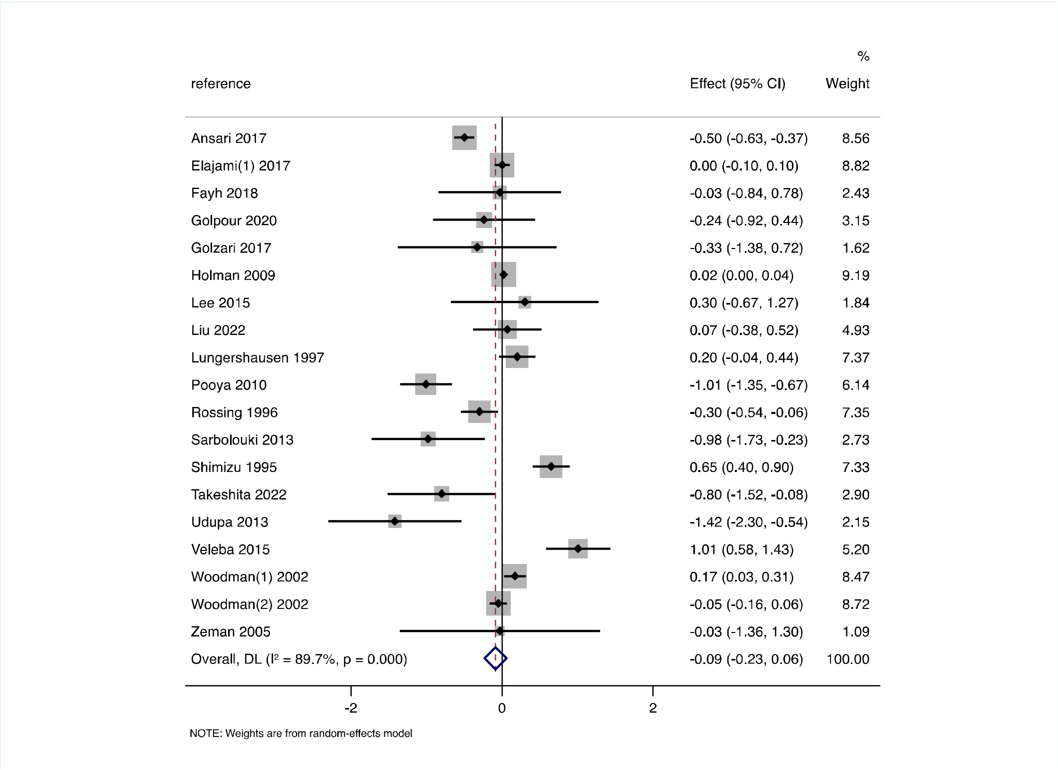

Figure S1-8-9 Forest plot of RCTs investigating the effect of antioxidant fatty acids supplementation on A1C in population with diabetes.

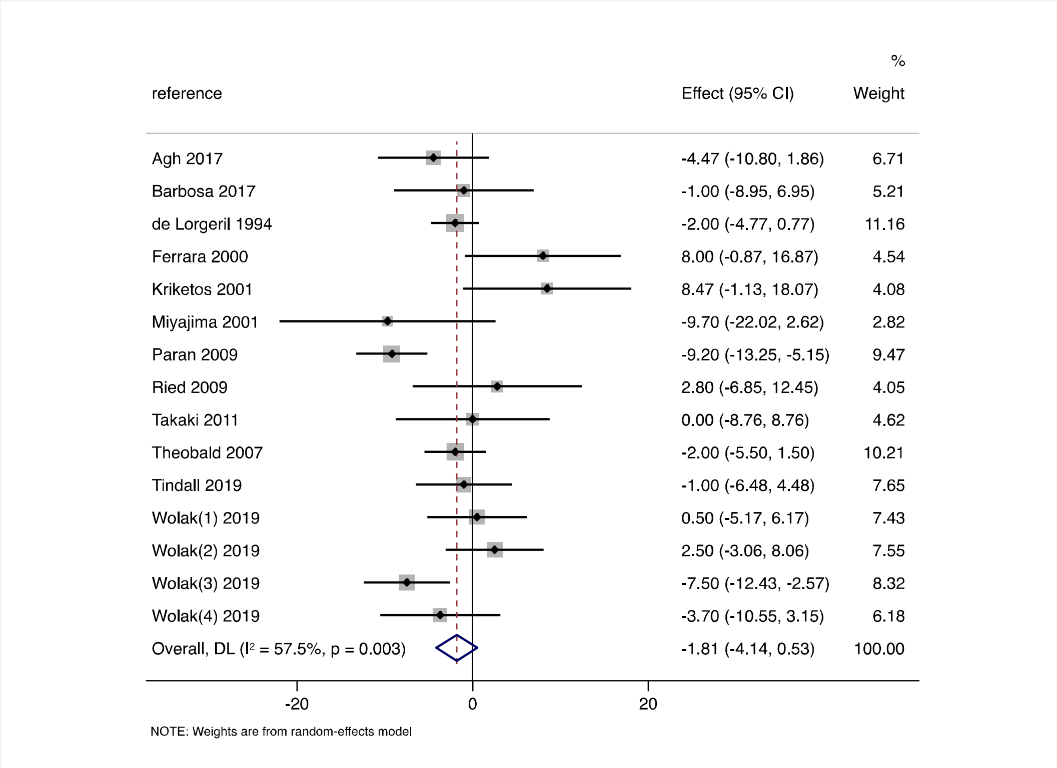

Figure S1-9-1 Forest plot of RCTs investigating the effect of antioxidant fatty acids supplementation on SBP in population with hypertension.

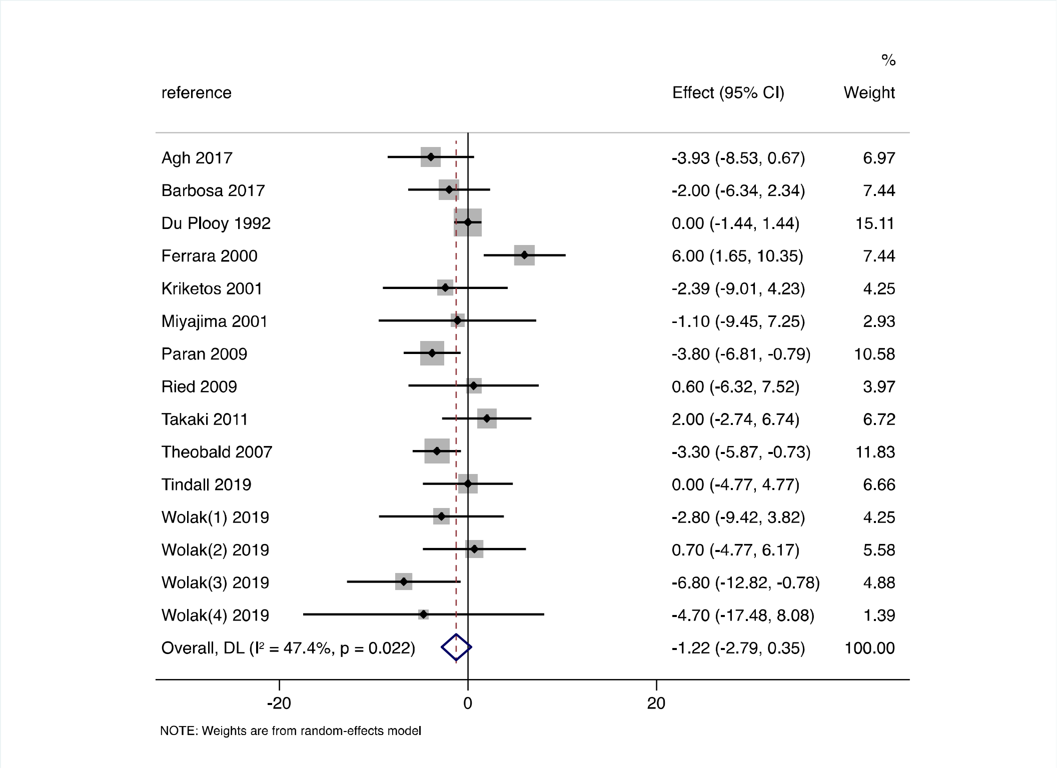

Figure S1-9-2 Forest plot of RCTs investigating the effect of antioxidant fatty acids supplementation on DBP in population with hypertension.

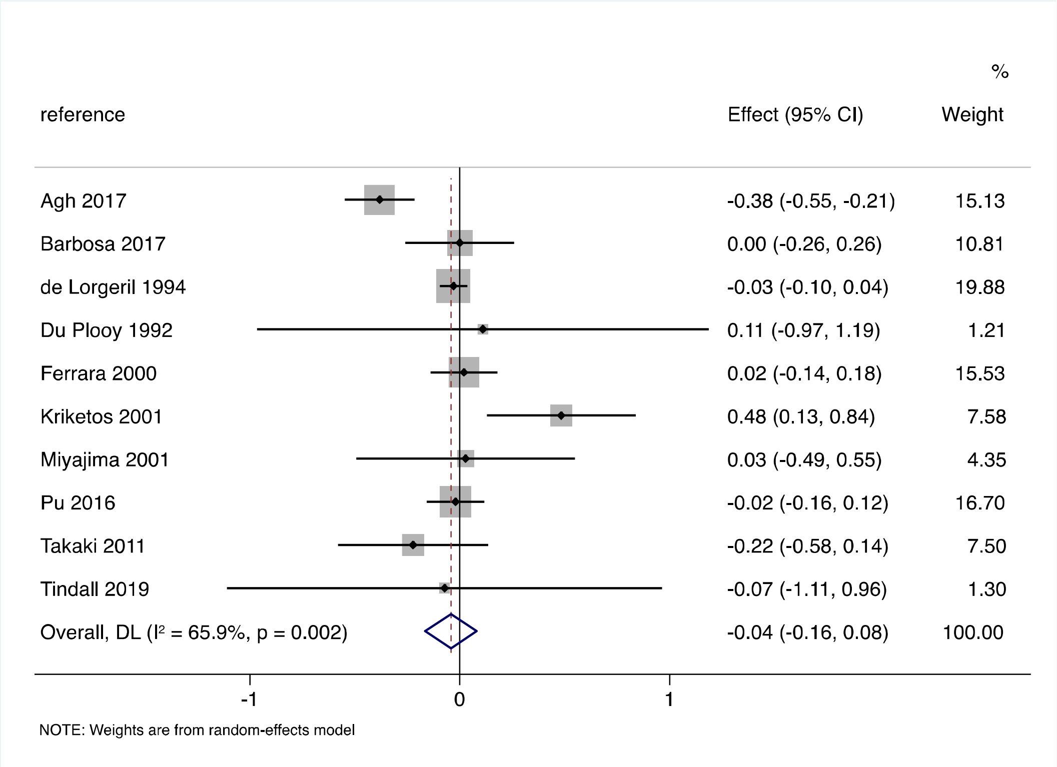

Figure S1-9-3 Forest plot of RCTs investigating the effect of antioxidant fatty acids supplementation on HDL in population with hypertension.

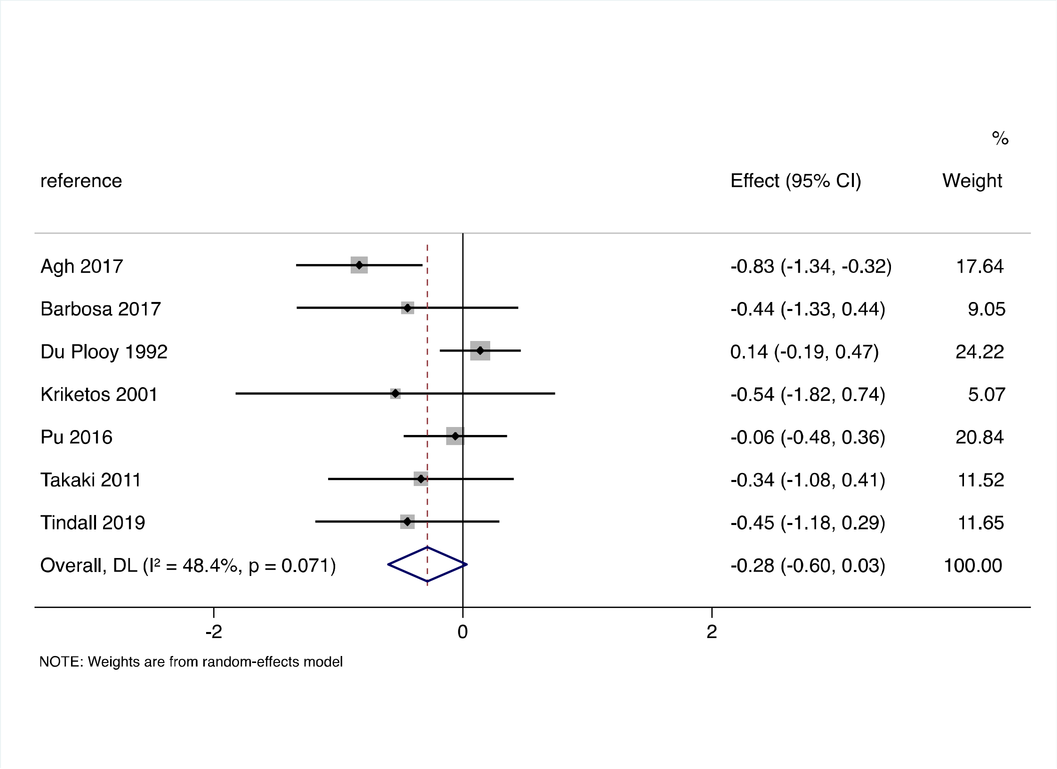

Figure S1-9-4 Forest plot of RCTs investigating the effect of antioxidant fatty acids supplementation on LDL in population with hypertension.

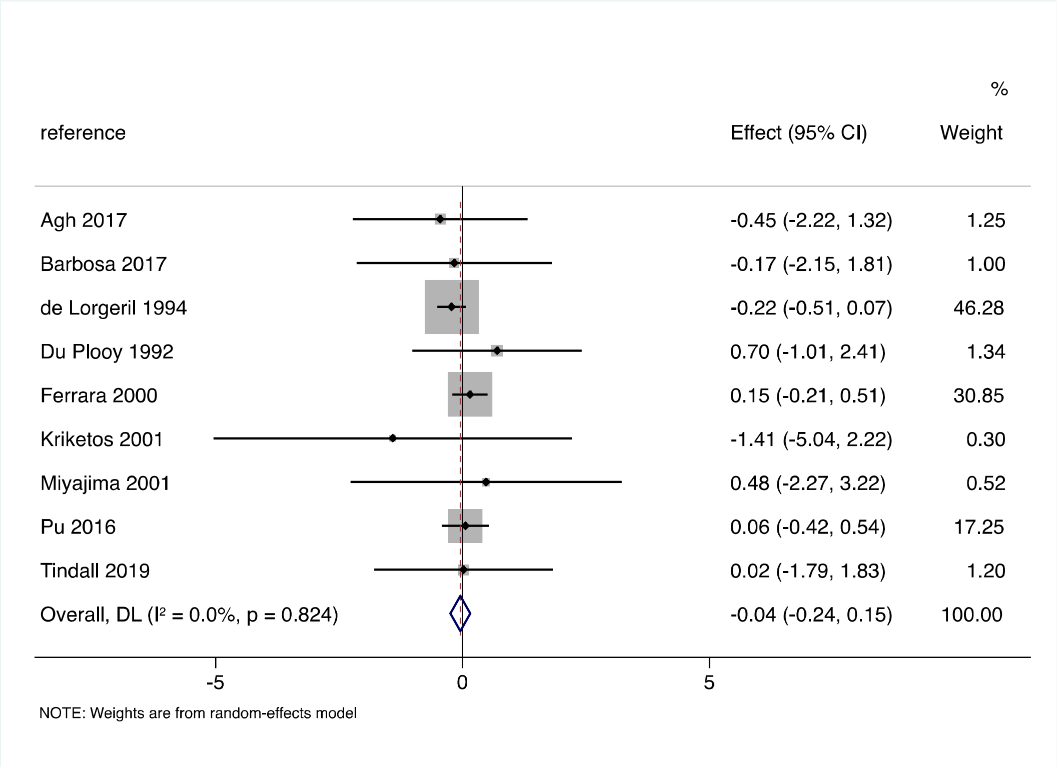

Figure S1-9-5 Forest plot of RCTs investigating the effect of antioxidant fatty acids supplementation on TG in population with hypertension.

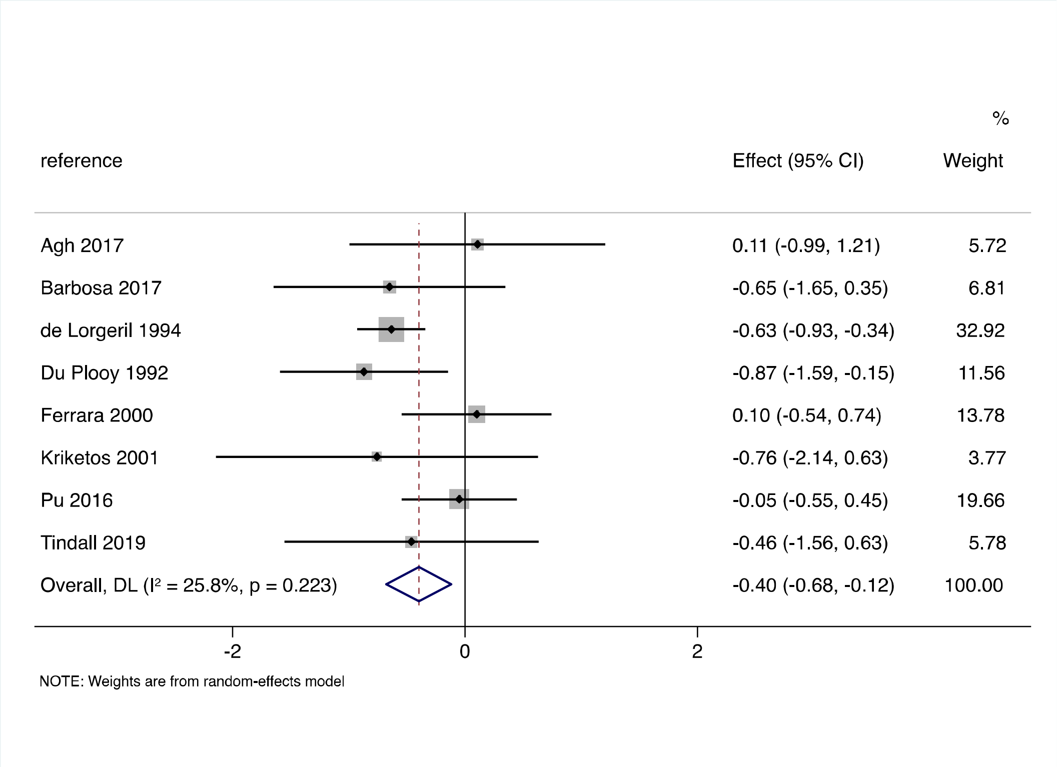

Figure S1-9-6 Forest plot of RCTs investigating the effect of antioxidant fatty acids supplementation on TC in population with hypertension.

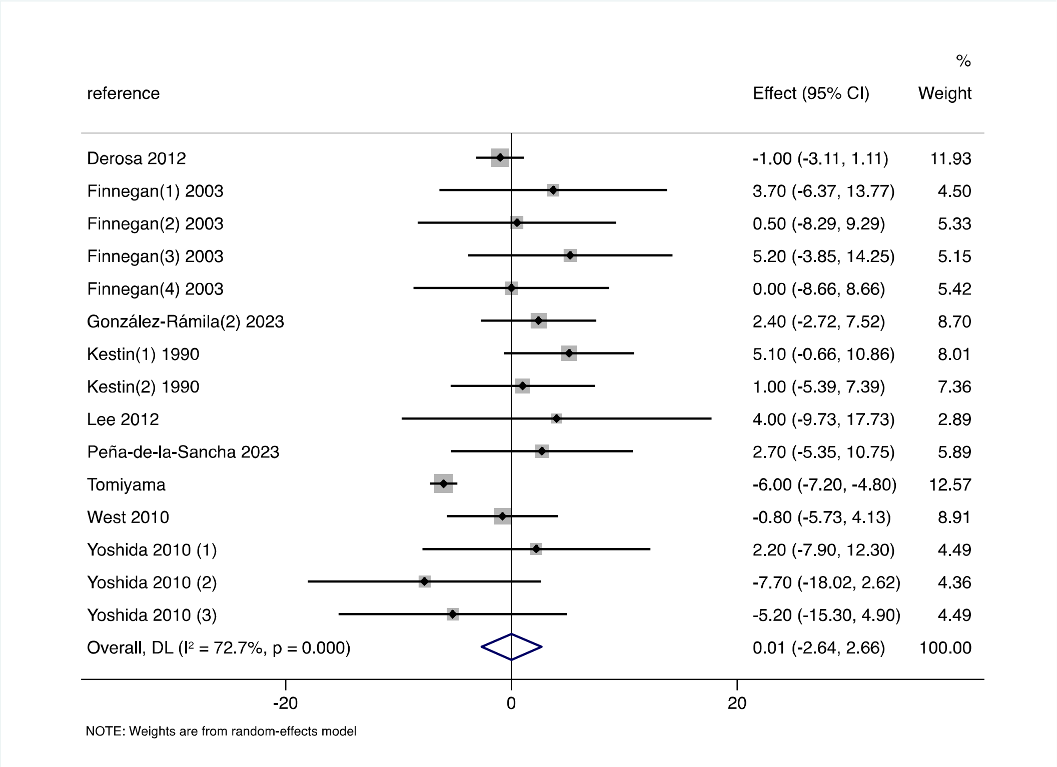

Figure S1-9-7 Forest plot of RCTs investigating the effect of antioxidant fatty acids supplementation on FBG in population with hypertension.

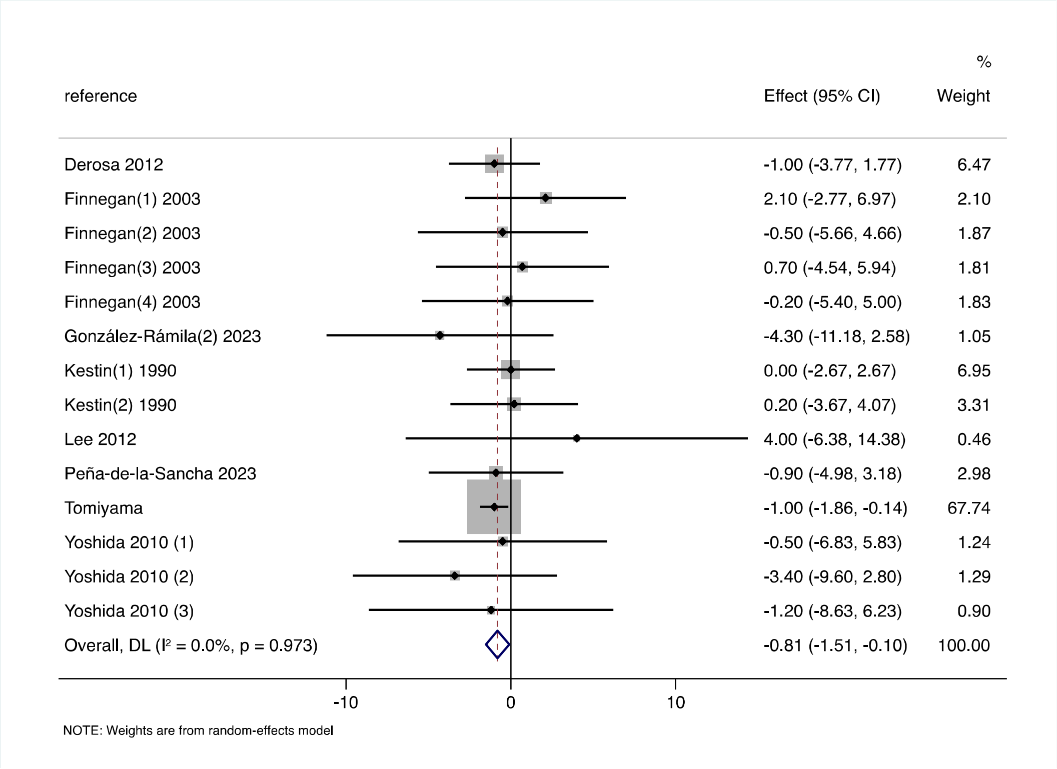

Figure S1-10-1 Forest plot of RCTs investigating the effect of antioxidant fatty acids supplementation on SBP in population with dyslipidemia.

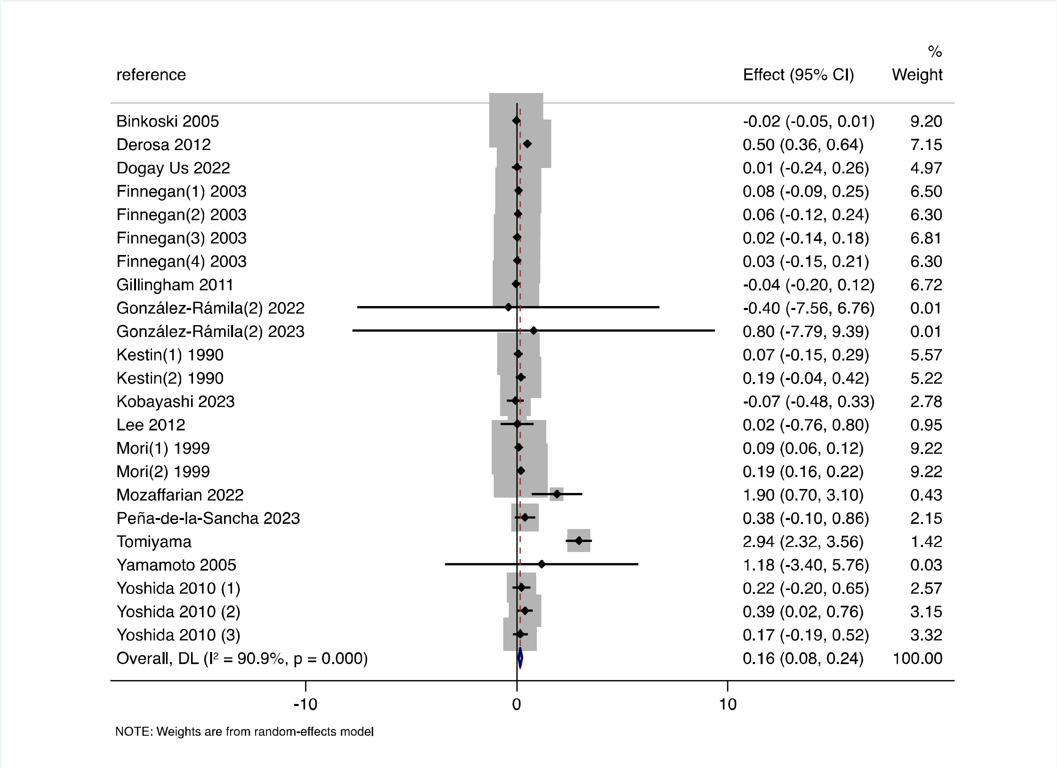

Figure S1-10-2 Forest plot of RCTs investigating the effect of antioxidant fatty acids supplementation on DBP in population with dyslipidemia.

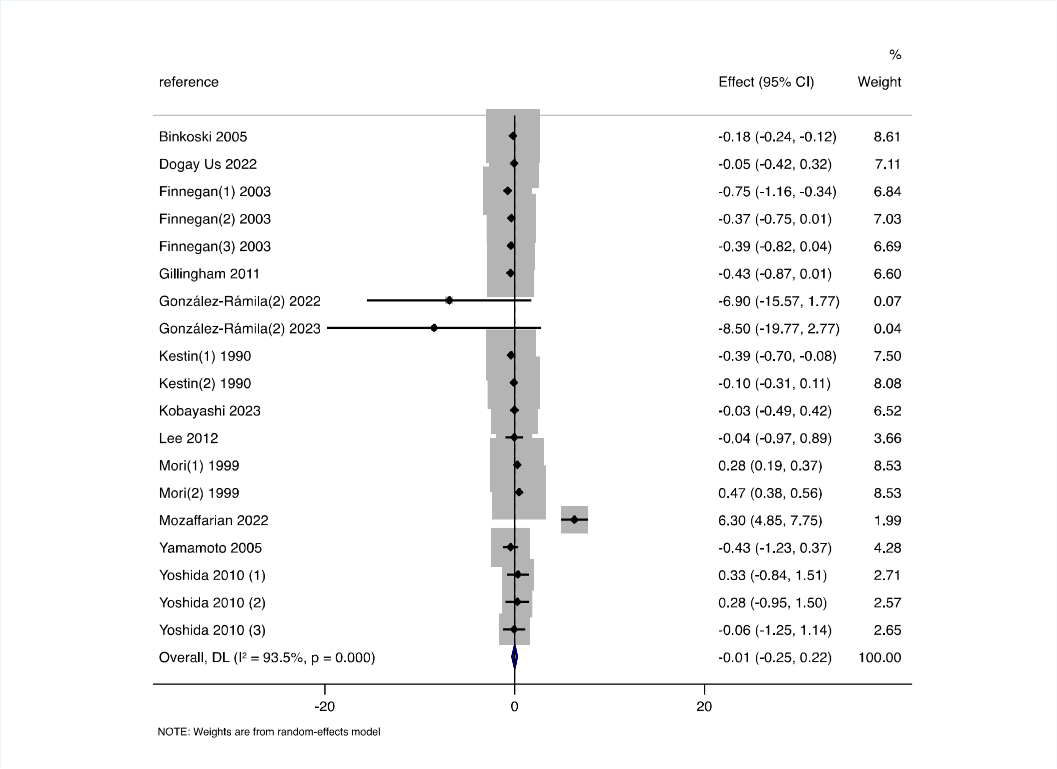

Figure S1-10-3 Forest plot of RCTs investigating the effect of antioxidant fatty acids supplementation on HDL in population with dyslipidemia.

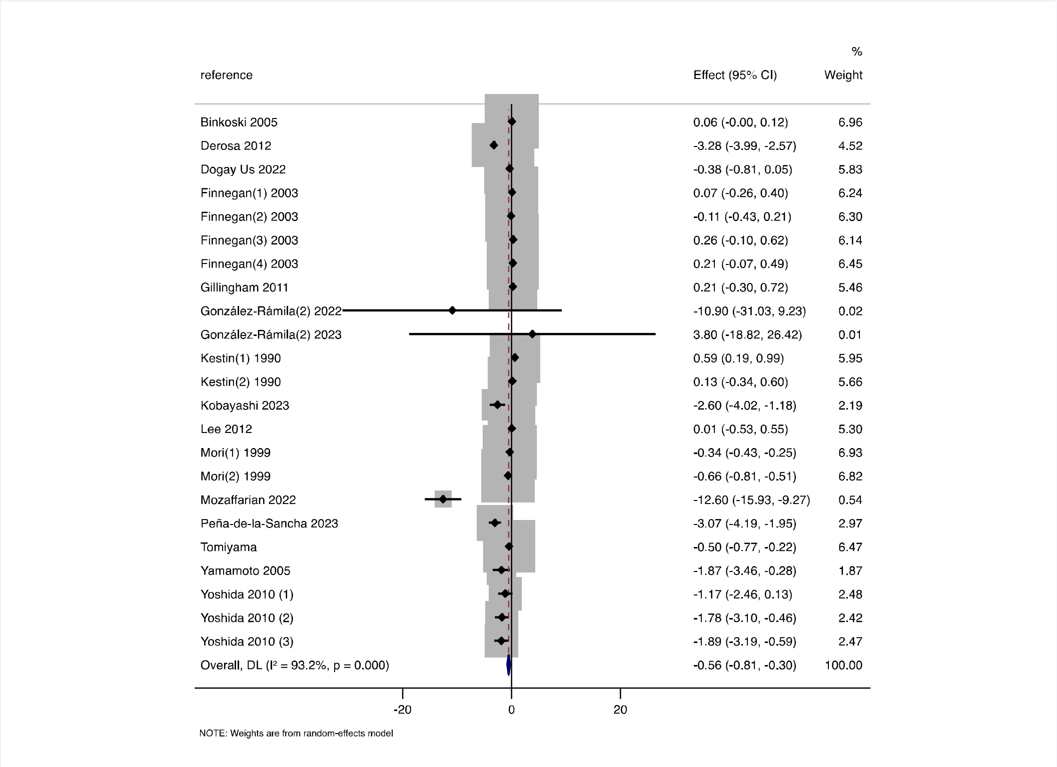

Figure S1-10-4 Forest plot of RCTs investigating the effect of antioxidant fatty acids supplementation on LDL in population with dyslipidemia.

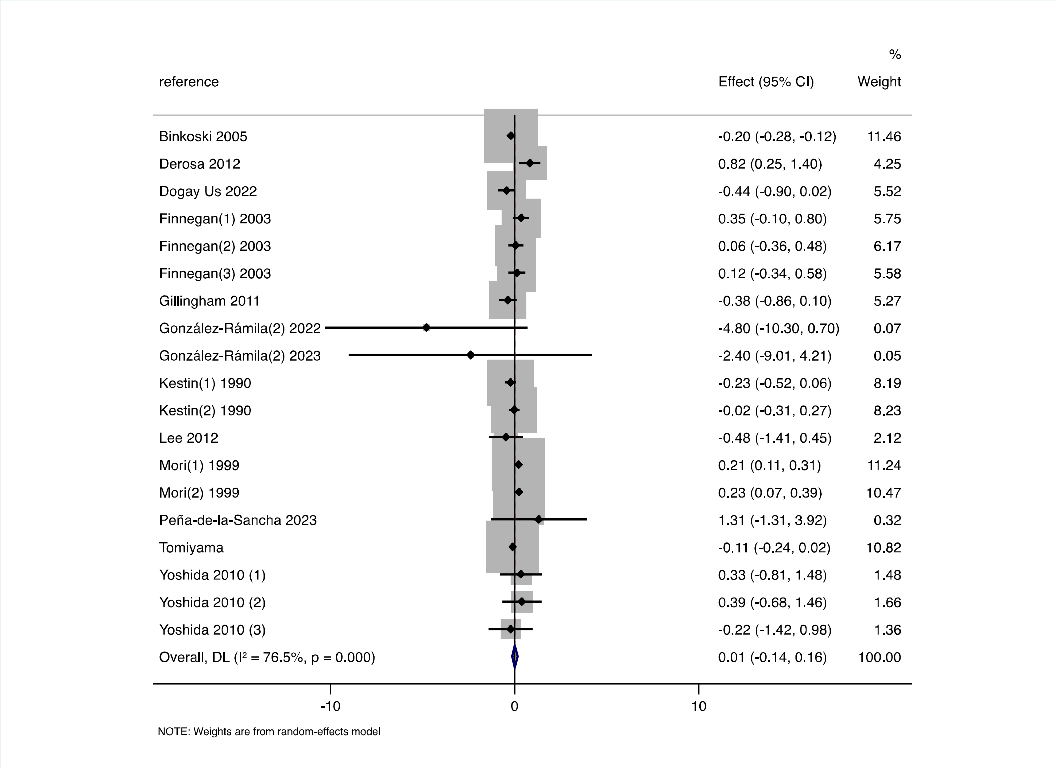

Figure S1-10-5 Forest plot of RCTs investigating the effect of antioxidant fatty acids supplementation on TG in population with dyslipidemia.

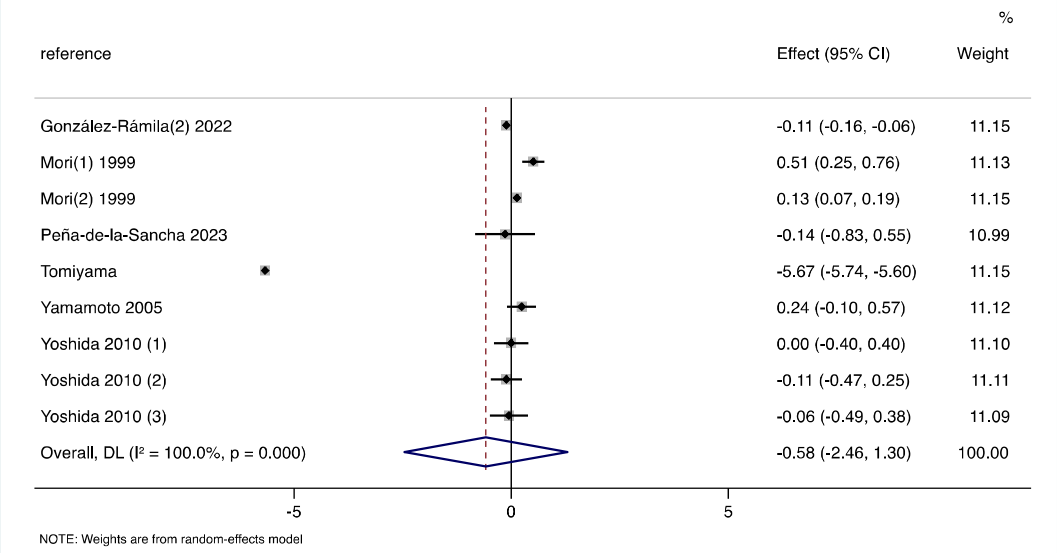

Figure S1-10-6 Forest plot of RCTs investigating the effect of antioxidant fatty acids supplementation on TC in population with dyslipidemia.

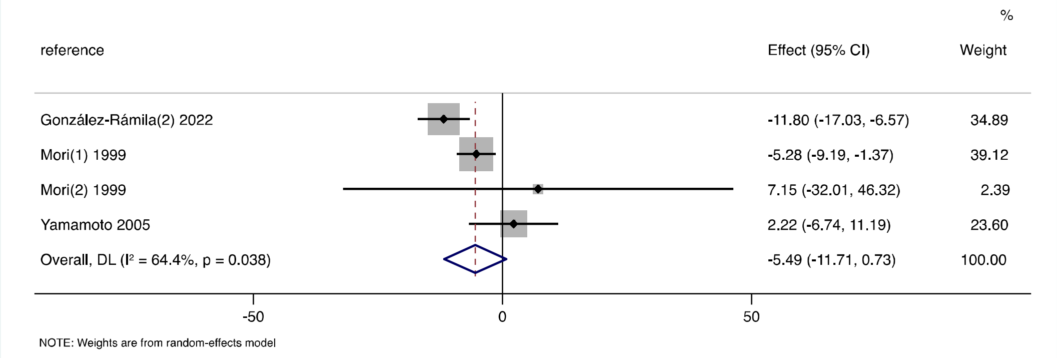

Figure S1-10-7 Forest plot of RCTs investigating the effect of antioxidant fatty acids supplementation on FBG in population with dyslipidemia.

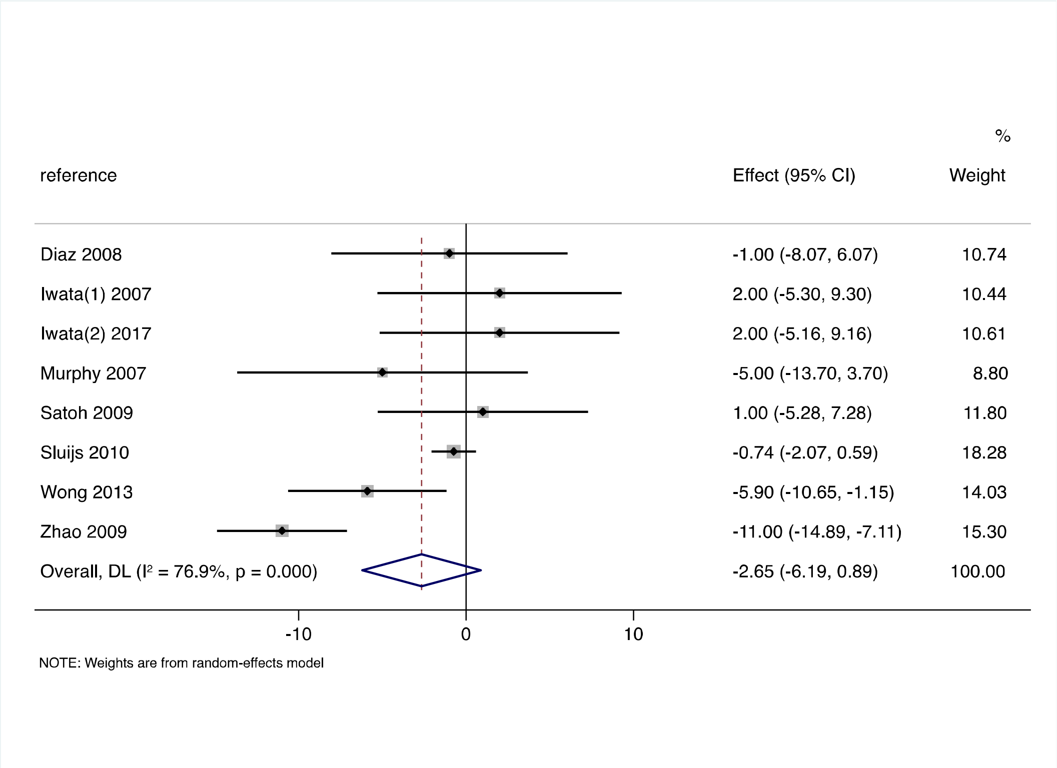

Figure S1-11-1 Forest plot of RCTs investigating the effect of antioxidant fatty acids supplementation on SBP in population with obesity.

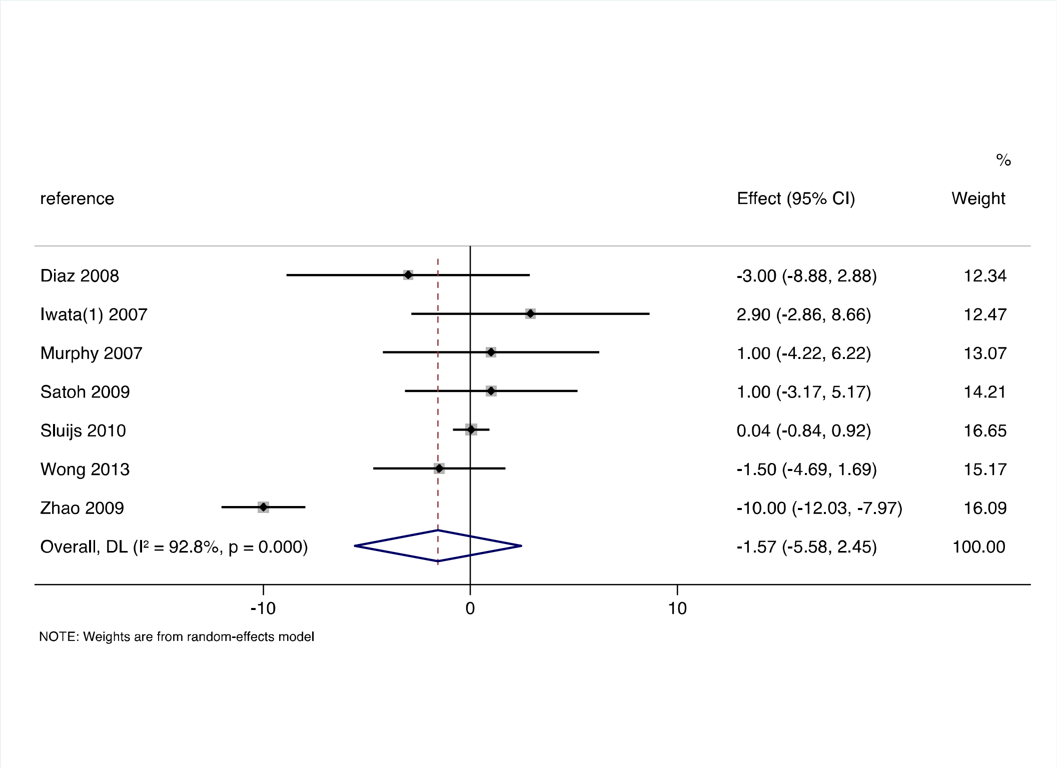

Figure S1-11-2 Forest plot of RCTs investigating the effect of antioxidant fatty acids supplementation on DBP in population with obesity.

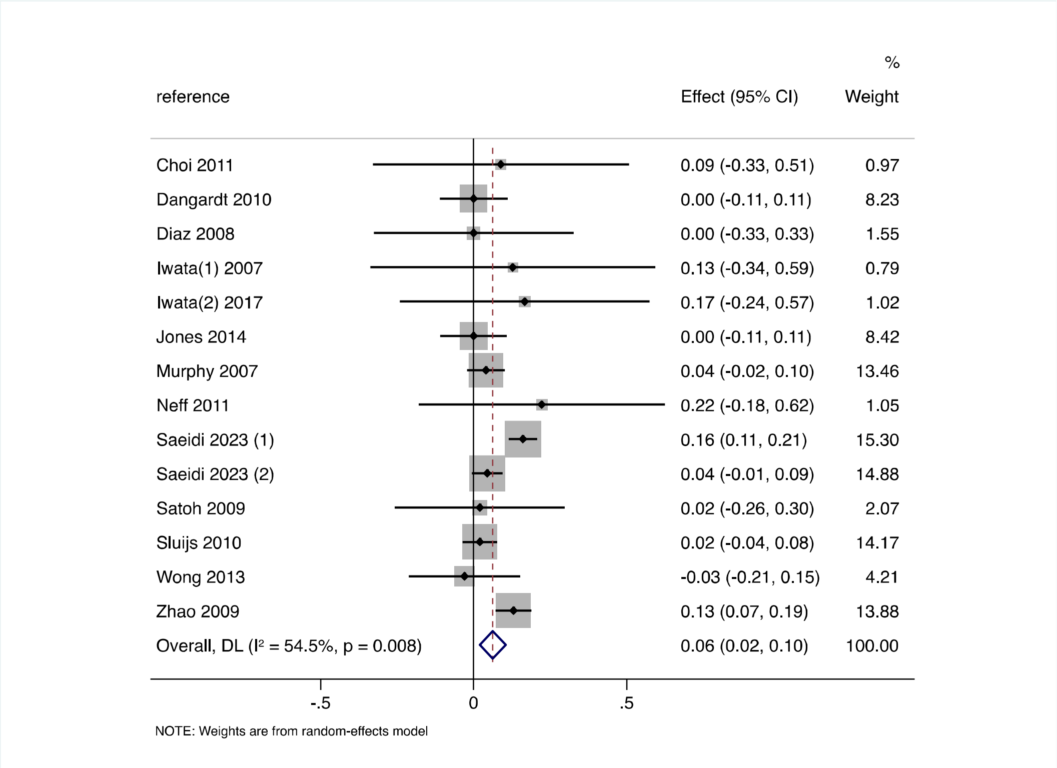

Figure S1-11-3 Forest plot of RCTs investigating the effect of antioxidant fatty acids supplementation on HDL in population with obesity.

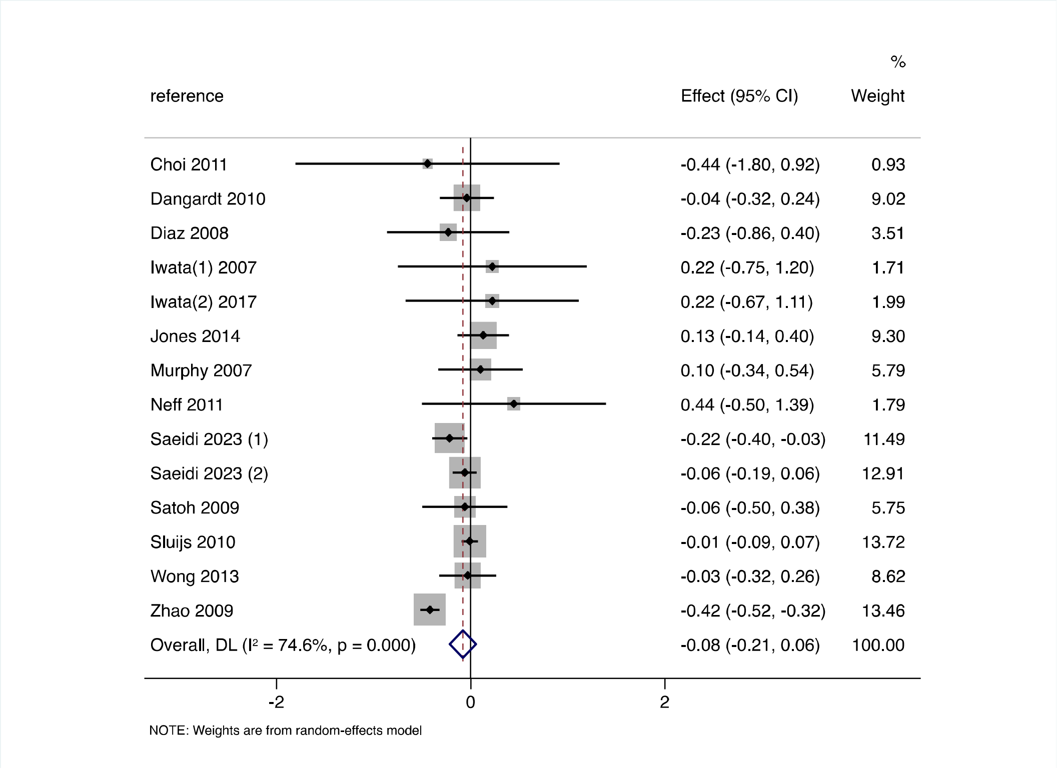

Figure S1-11-4 Forest plot of RCTs investigating the effect of antioxidant fatty acids supplementation on LDL in population with obesity.

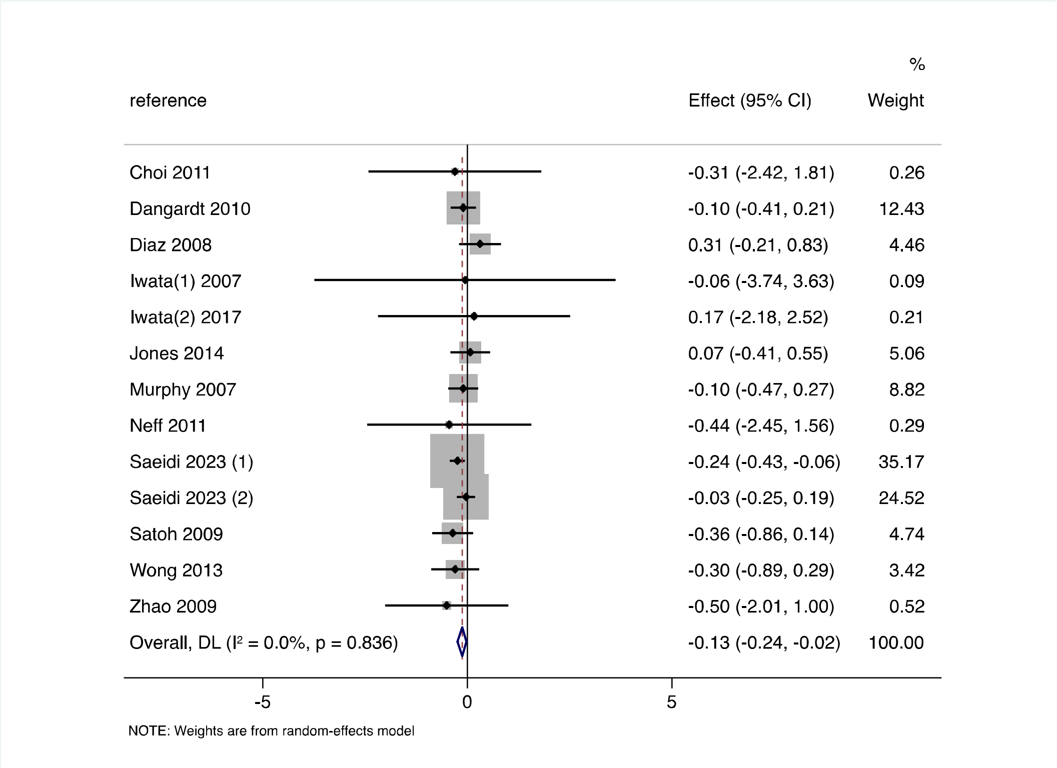

Figure S1-11-5 Forest plot of RCTs investigating the effect of antioxidant fatty acids supplementation on TG in population with obesity.

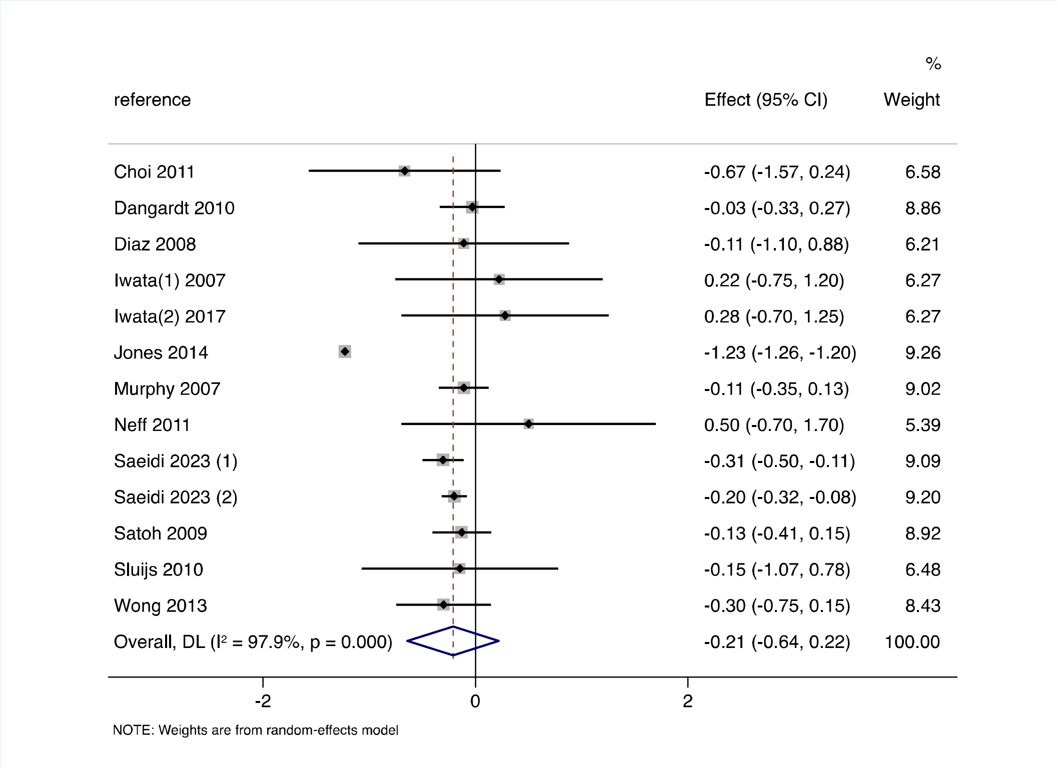

Figure S1-11-6 Forest plot of RCTs investigating the effect of antioxidant fatty acids supplementation on TC in population with obesity.

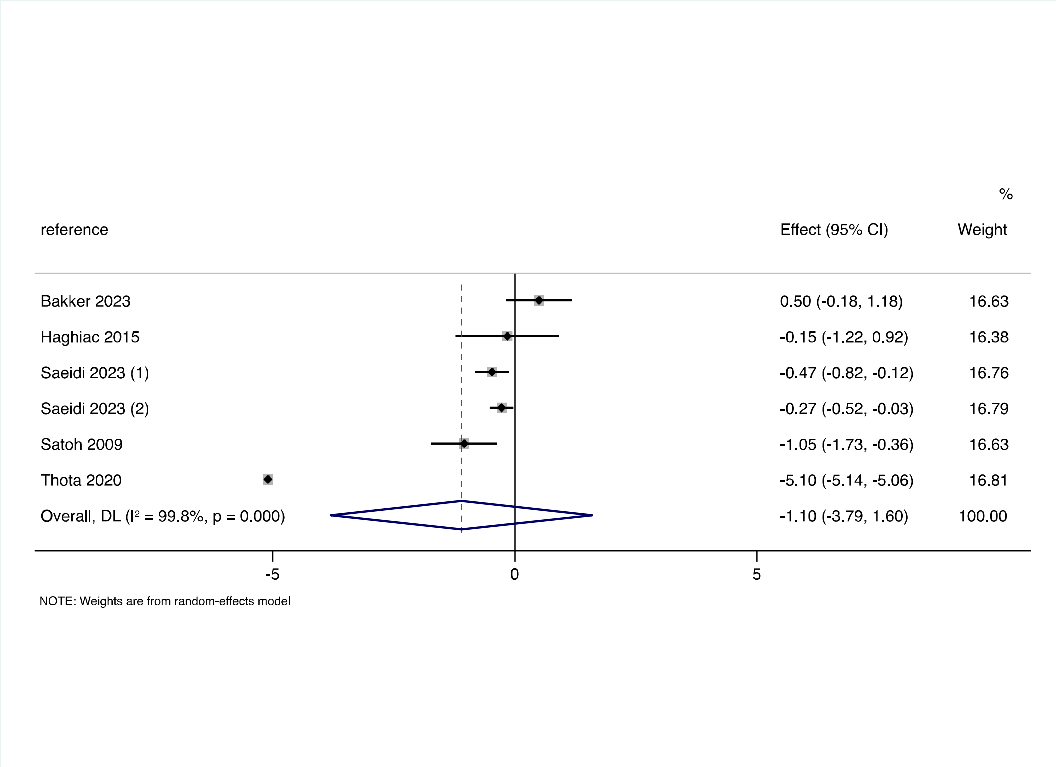

Figure S1-11-7 Forest plot of RCTs investigating the effect of antioxidant fatty acids supplementation on FBG in population with obesity.

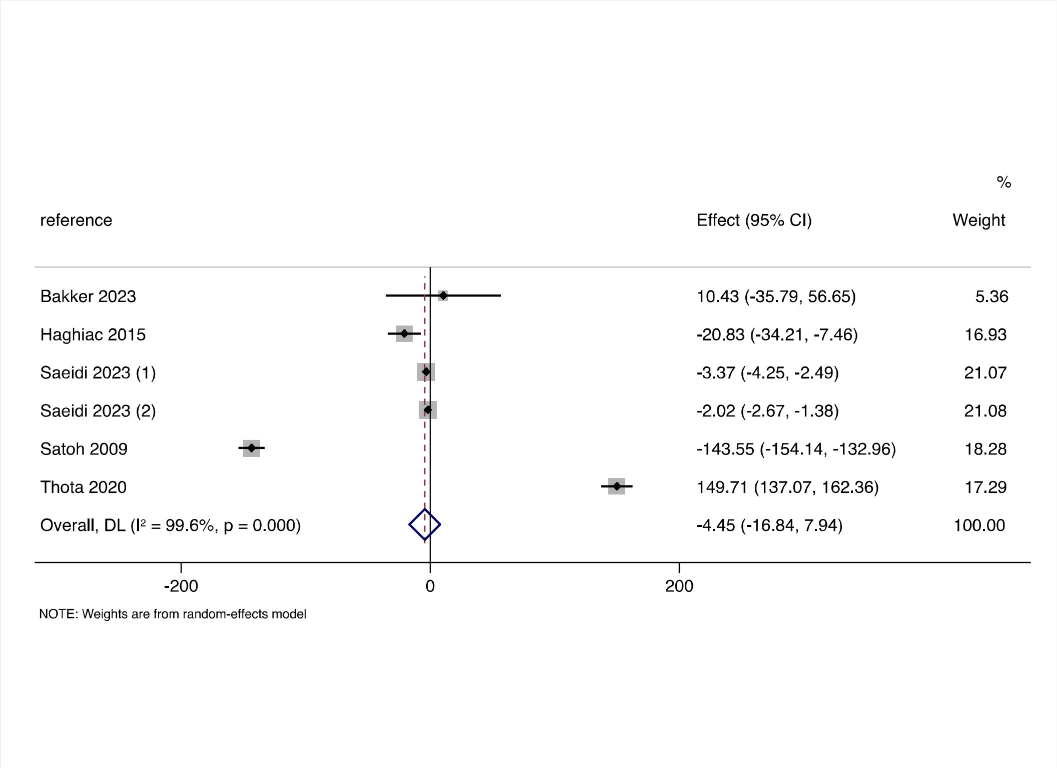

Figure S1-11-8 Forest plot of RCTs investigating the effect of antioxidant fatty acids supplementation on FBI in population with obesity.

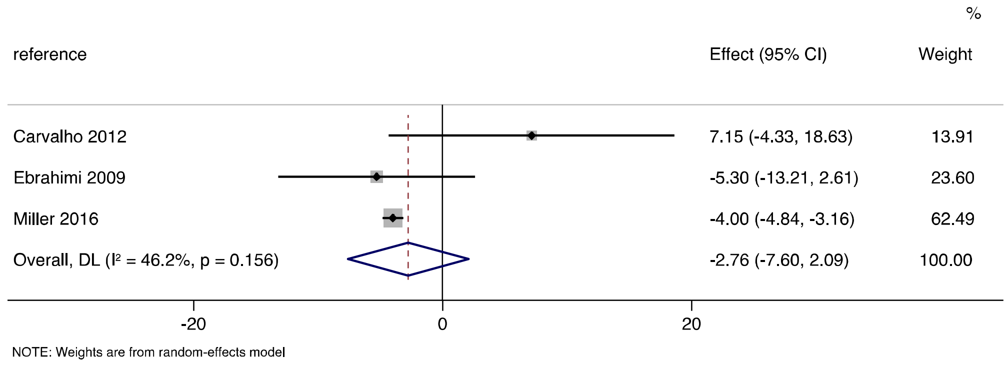

Figure S1-12-1 Forest plot of RCTs investigating the effect of antioxidant fatty acids supplementation on SBP in population with metabolic syndrome.

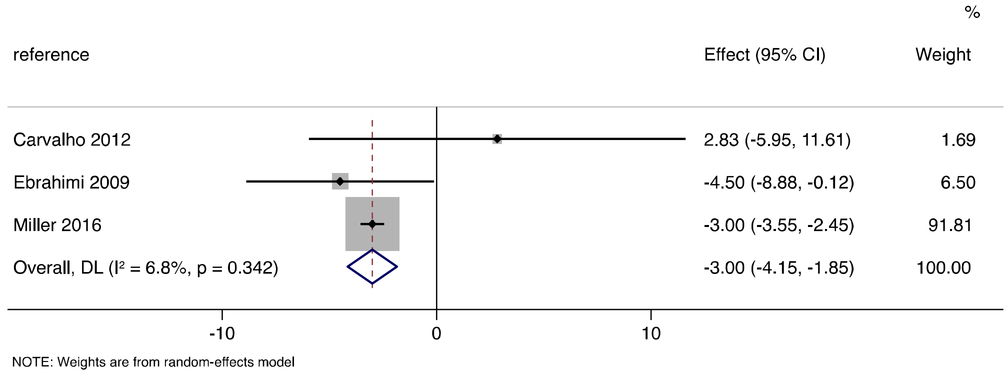

Figure S1-12-2 Forest plot of RCTs investigating the effect of antioxidant fatty acids supplementation on DBP in population with metabolic syndrome.

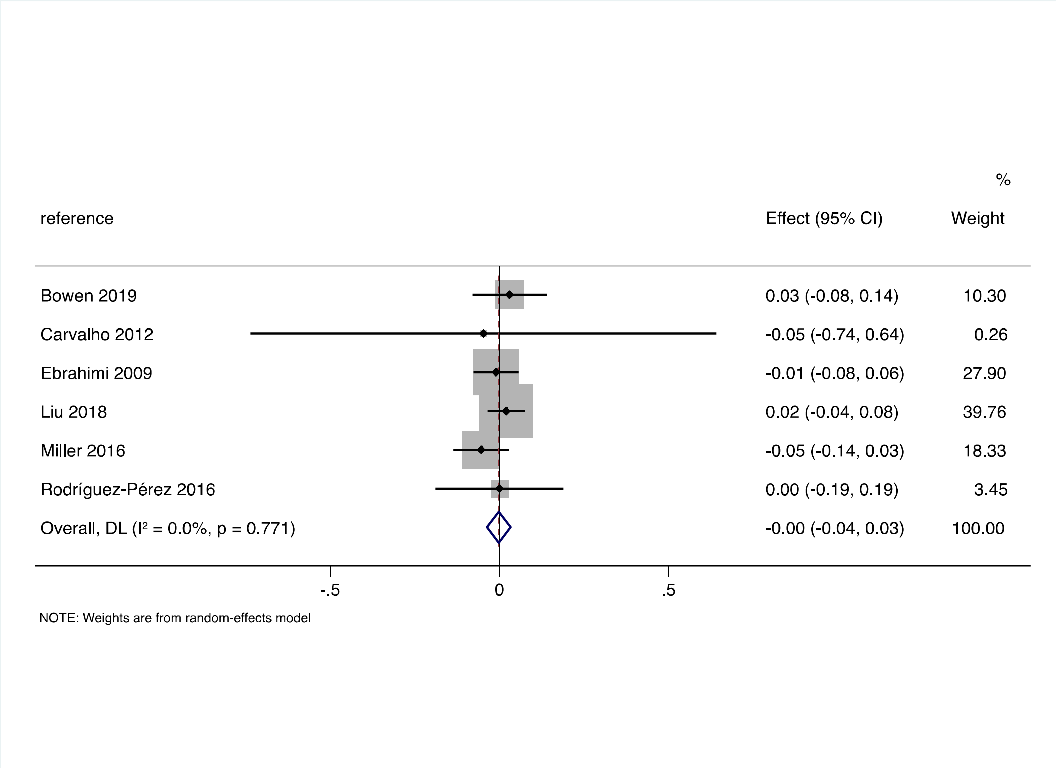

Figure S1-12-3 Forest plot of RCTs investigating the effect of antioxidant fatty acids supplementation on HDL in population with metabolic syndrome.

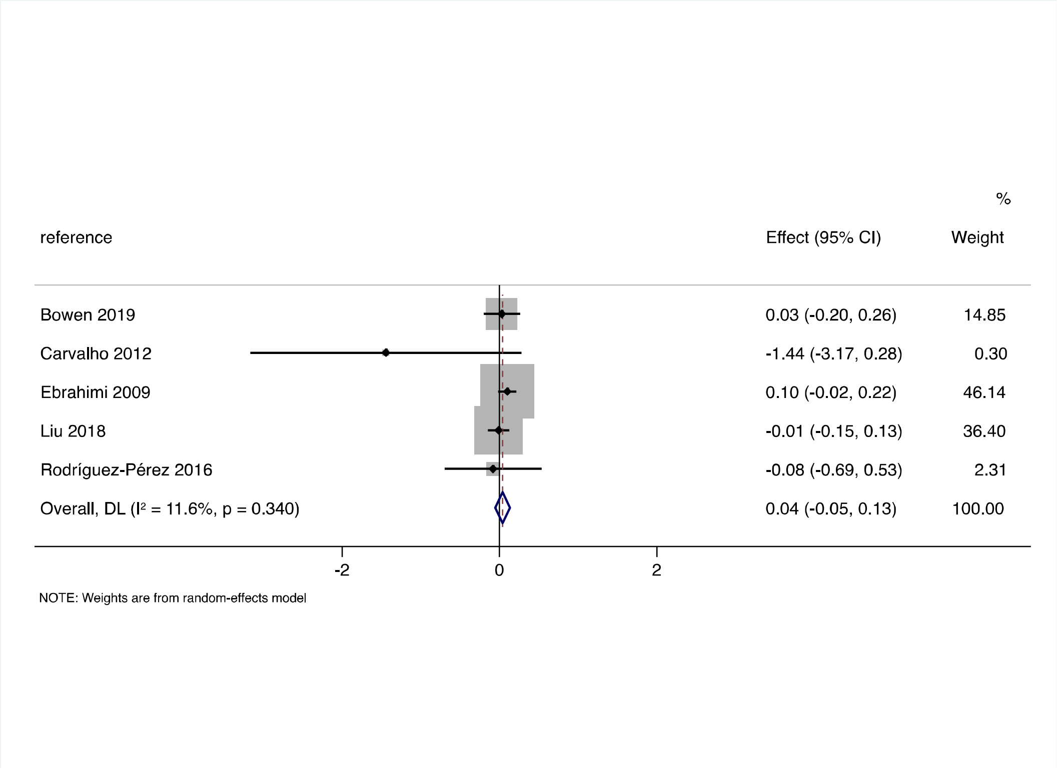

Figure S1-12-4 Forest plot of RCTs investigating the effect of antioxidant fatty acids supplementation on LDL in population with metabolic syndrome.

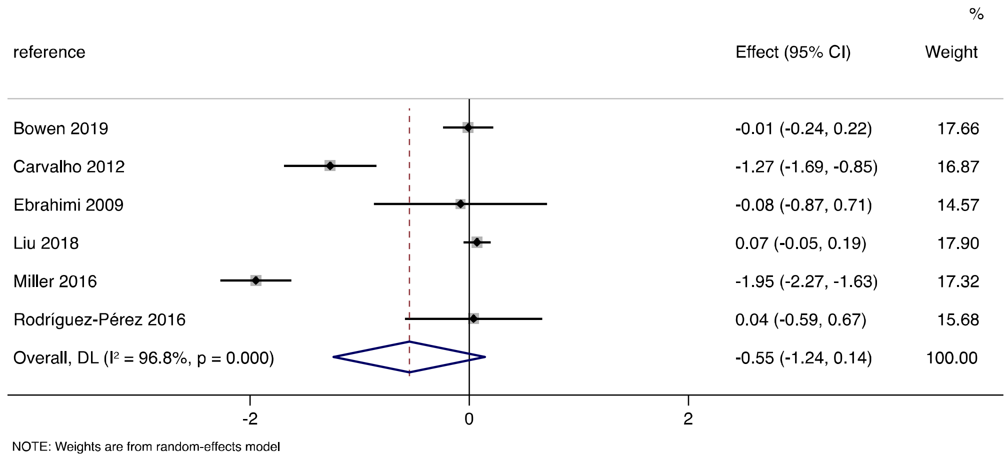

Figure S1-12-5 Forest plot of RCTs investigating the effect of antioxidant fatty acids supplementation on TG in population with metabolic syndrome.

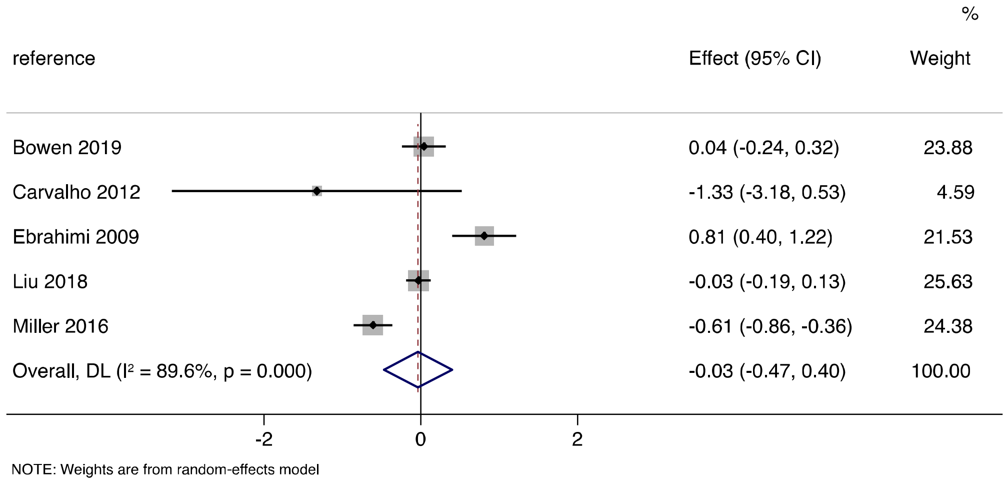

Figure S1-12-6 Forest plot of RCTs investigating the effect of antioxidant fatty acids supplementation on TC in population with metabolic syndrome.

Supplemental 3- Risk of bias

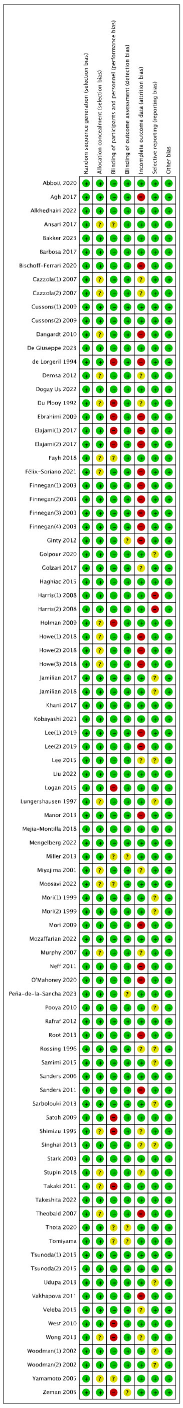

Figure S2-1 Risk of bias graph: the distribution of risk of bias judgments (Low risk of bias, Green; High risk of bias, Red; Unclear, Yellow) for each study (n3 fatty acid).

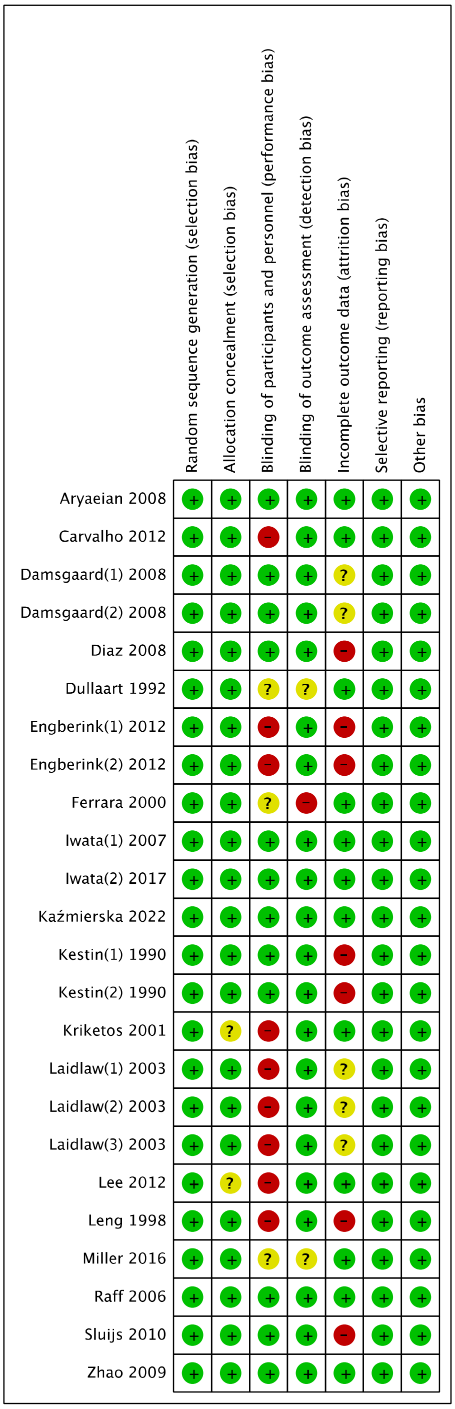

Figure S2-2 Risk of bias graph: the distribution of risk of bias judgments (Low risk of bias, Green; High risk of bias, Red; Unclear, Yellow) for each study (n6 fatty acid).

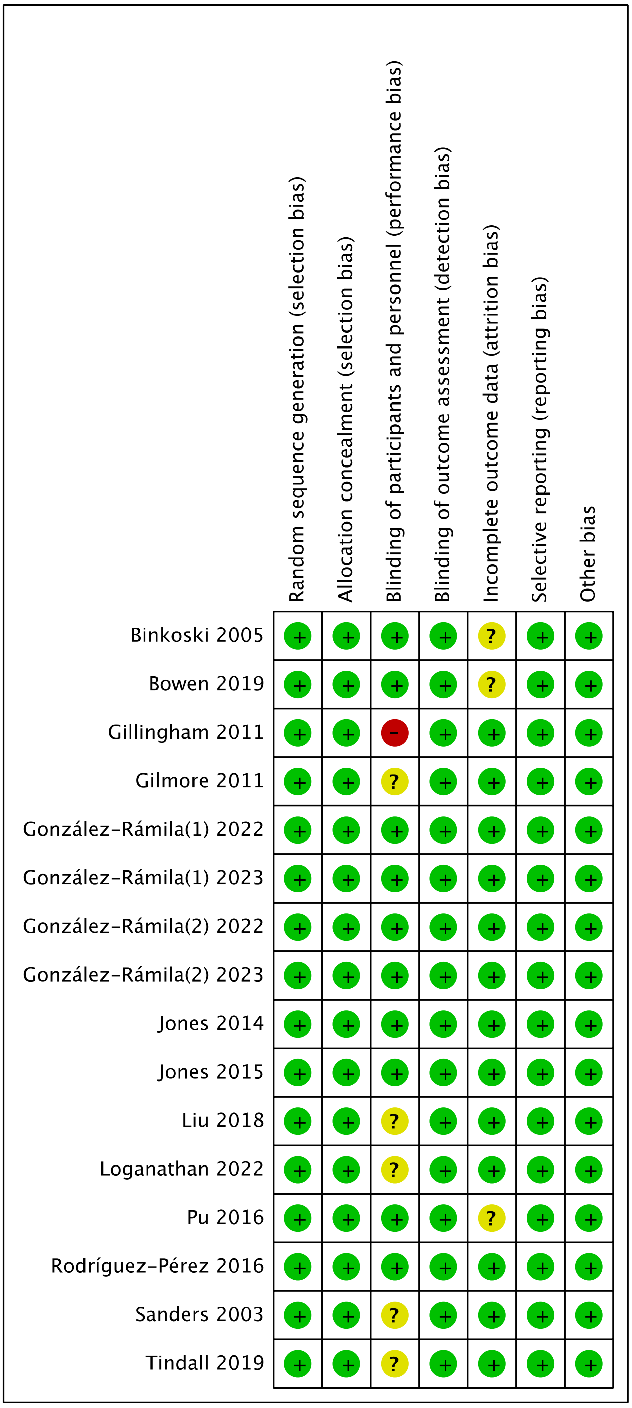

Figure S2-3 Risk of bias graph: the distribution of risk of bias judgments (Low risk of bias, Green; High risk of bias, Red; Unclear, Yellow) for each study (n9 fatty acid).

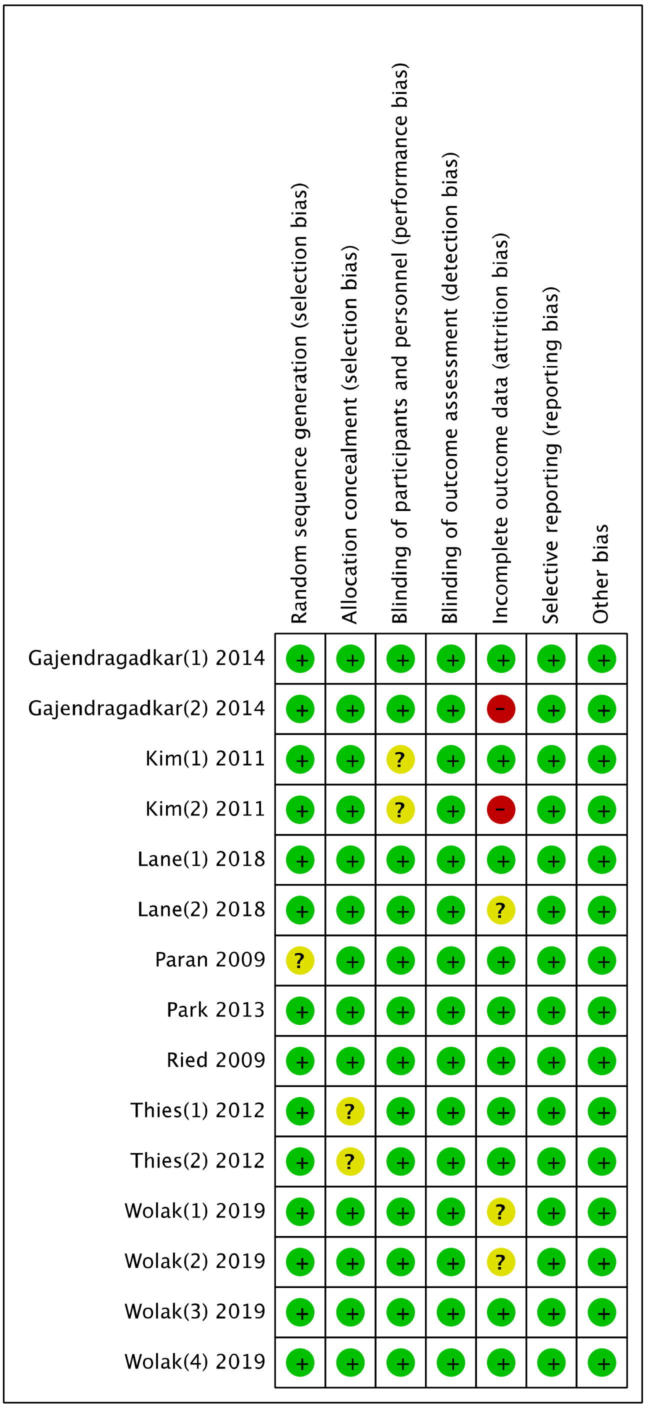

Figure S2-4 Risk of bias graph: the distribution of risk of bias judgments (Low risk of bias, Green; High risk of bias, Red; Unclear, Yellow) for each study (lycopene).

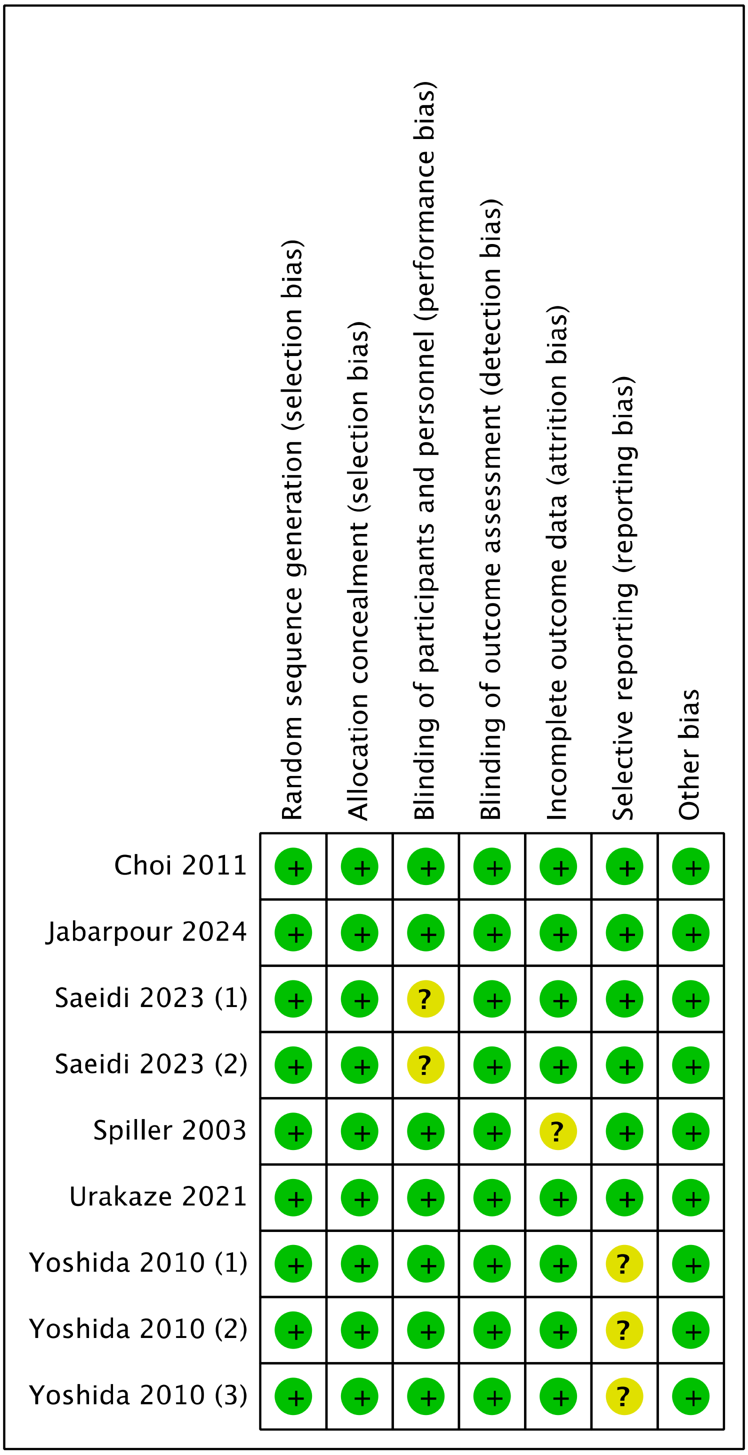

Figure S2-5 Risk of bias graph: the distribution of risk of bias judgments (Low risk of bias, Green; High risk of bias, Red; Unclear, Yellow) for each study (astaxanthin).

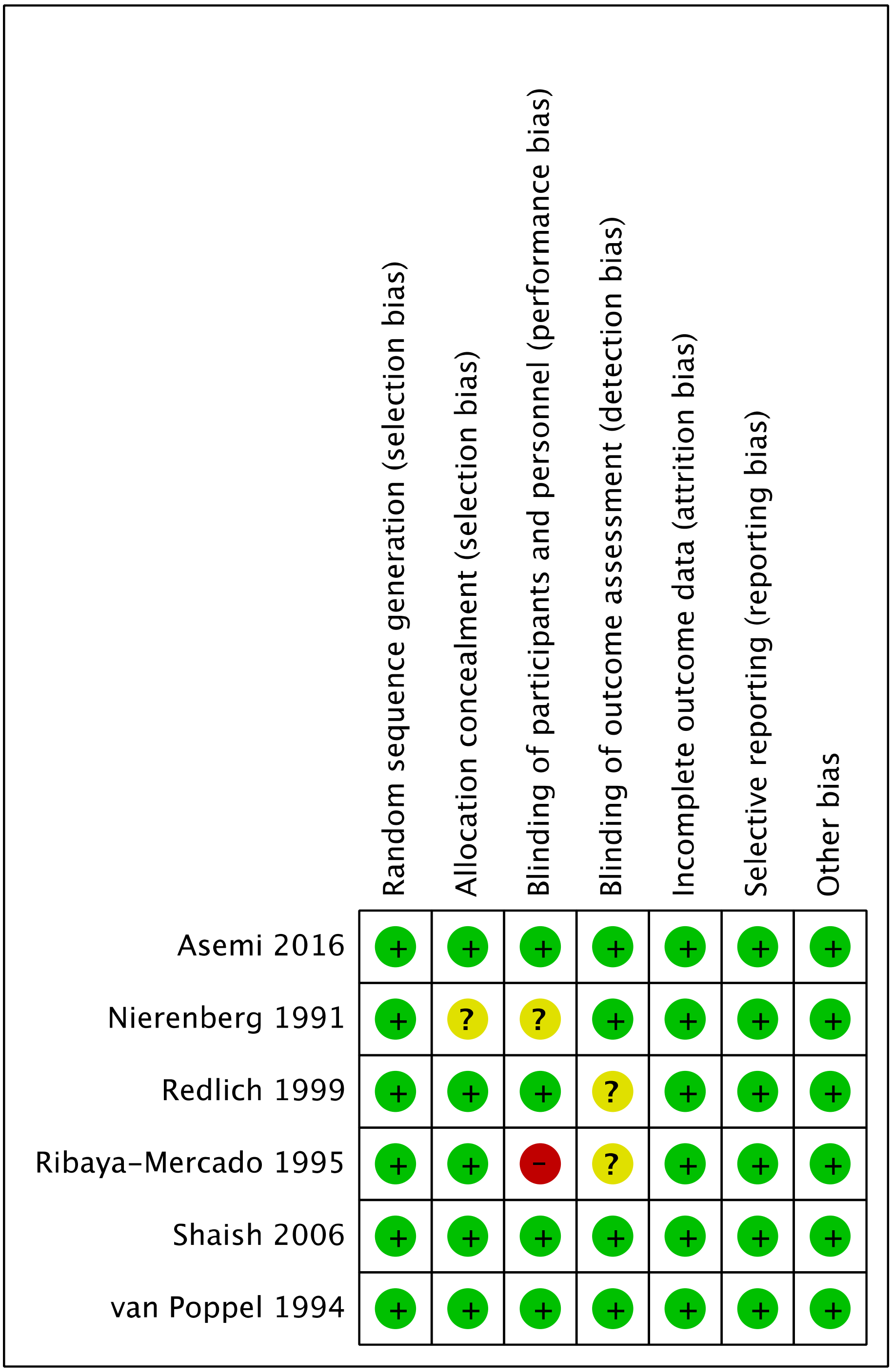

Figure S2-6 Risk of bias graph: the distribution of risk of bias judgments (Low risk of bias, Green; High risk of bias, Red; Unclear, Yellow) for each study (beta-carotene).

Supplemental 4 - Funnel plot

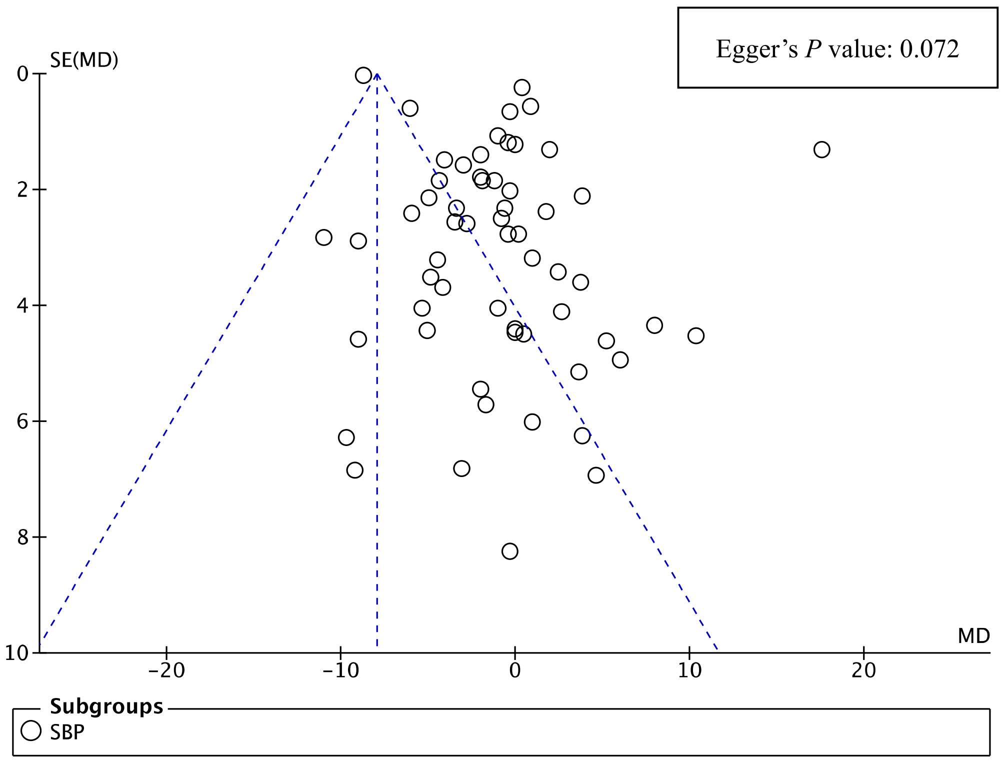

Figure S3-1-1 Funnel plot of RCTs investigating the effect of n3 fatty acids supplementation on SBP.

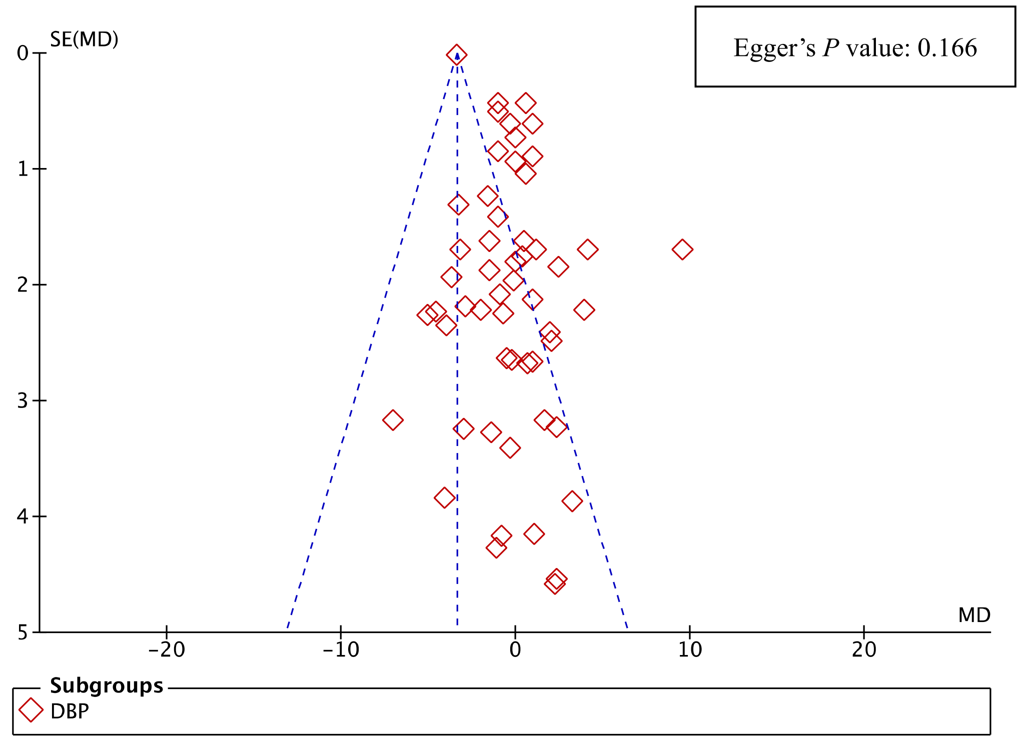

Figure S3-1-2 Funnel plot of RCTs investigating the effect of n3 fatty acids supplementation on DBP.

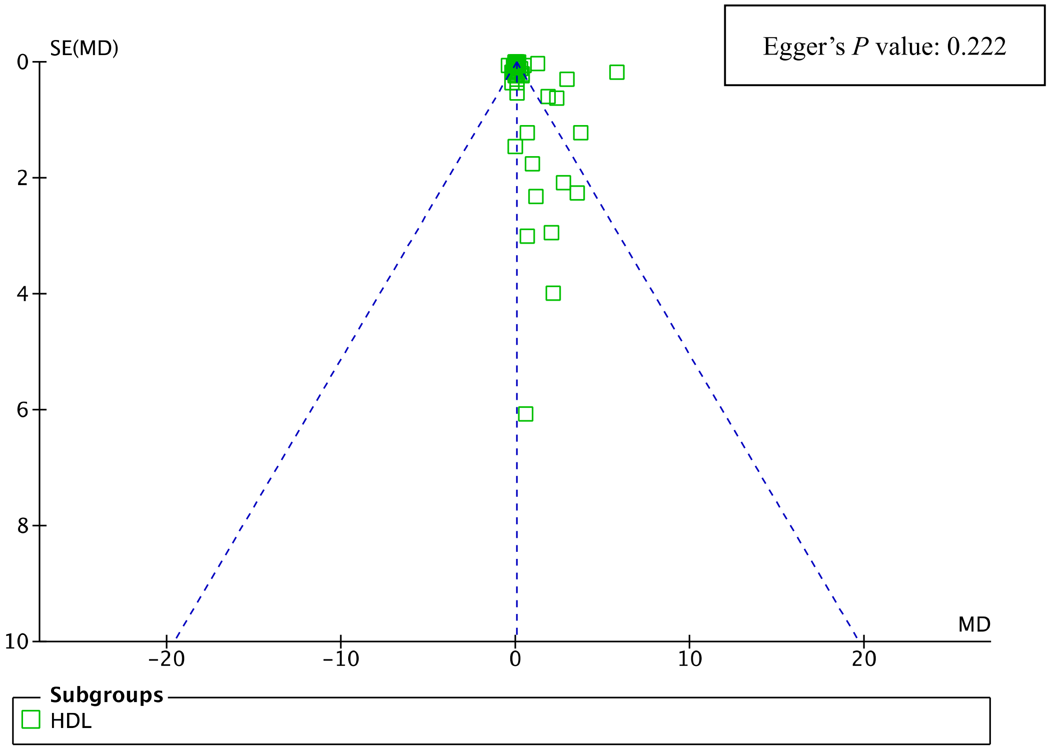

Figure S3-1-3 Funnel plot of RCTs investigating the effect of n3 fatty acids supplementation on HDL-C.

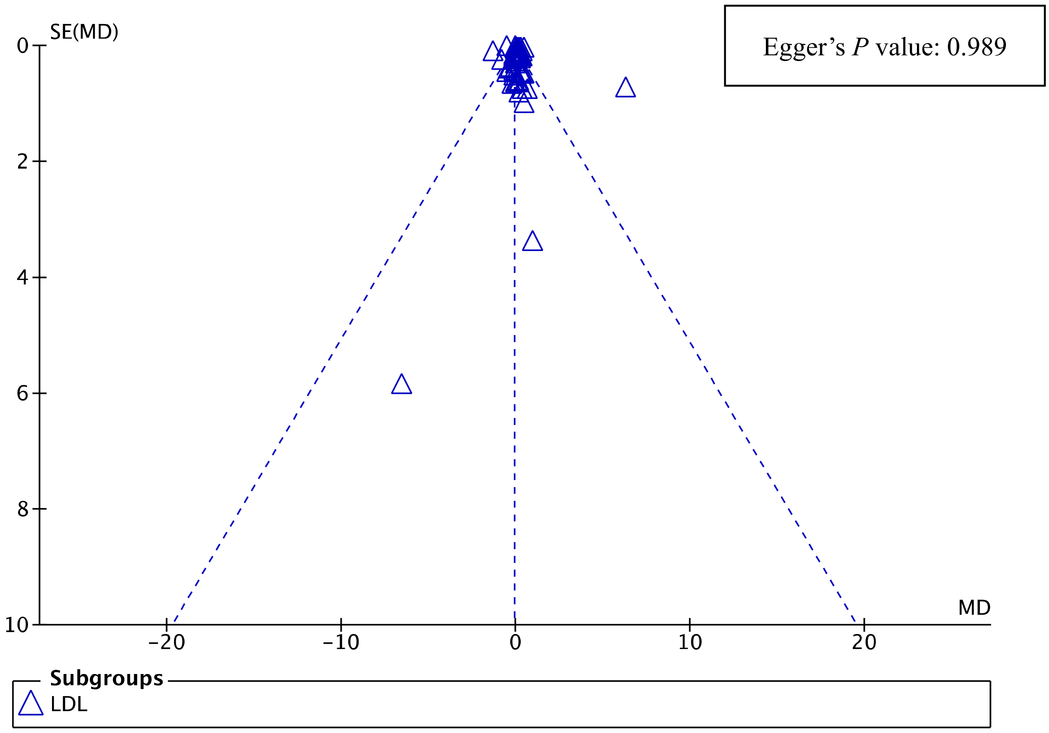

Figure S3-1-4 Funnel plot of RCTs investigating the effect of n3 fatty acids supplementation on LDL-C.

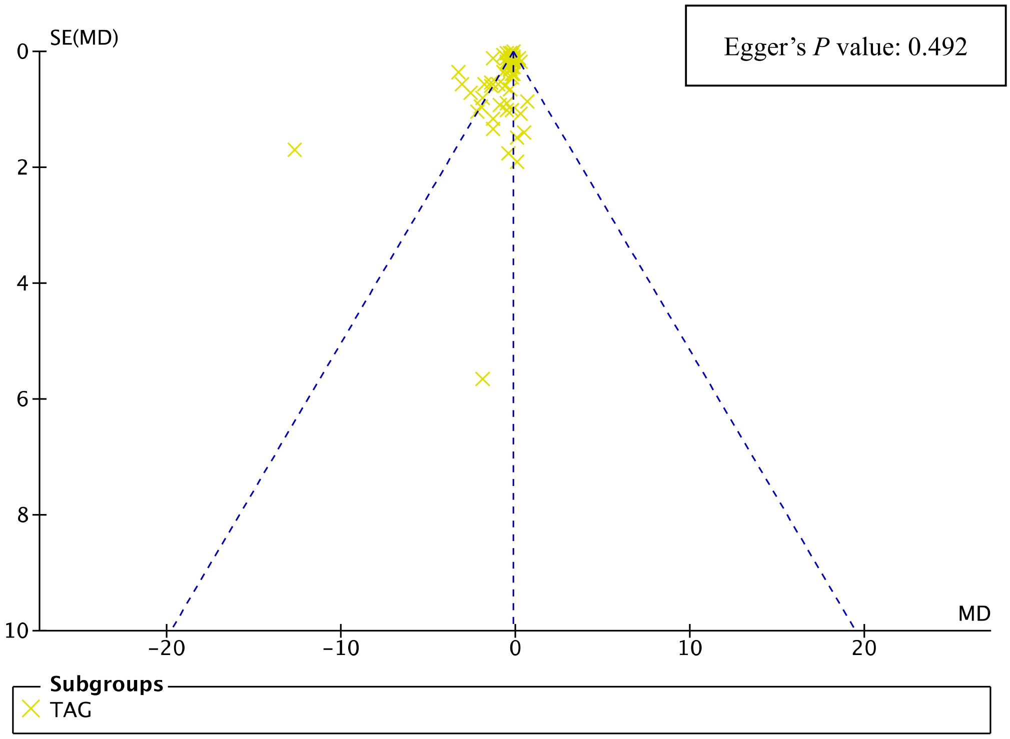

Figure S3-1-5 Funnel plot of RCTs investigating the effect of n3 fatty acids supplementation on TG

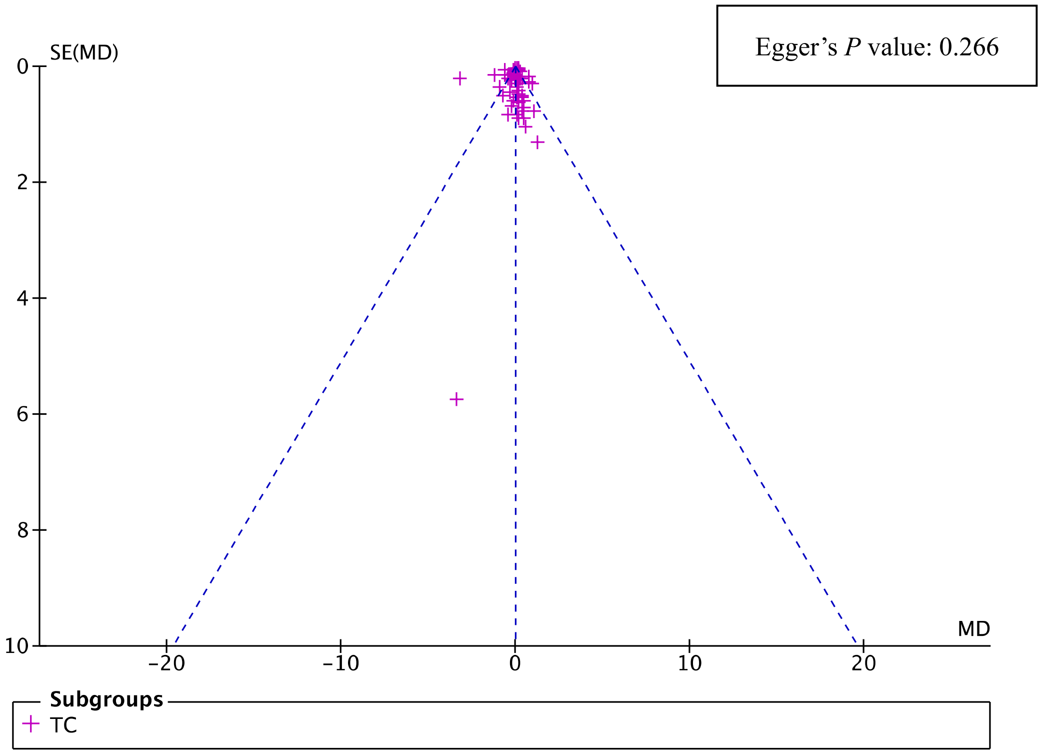

Figure S3-1-6 Funnel plot of RCTs investigating the effect of n3 fatty acids supplementation on TC.

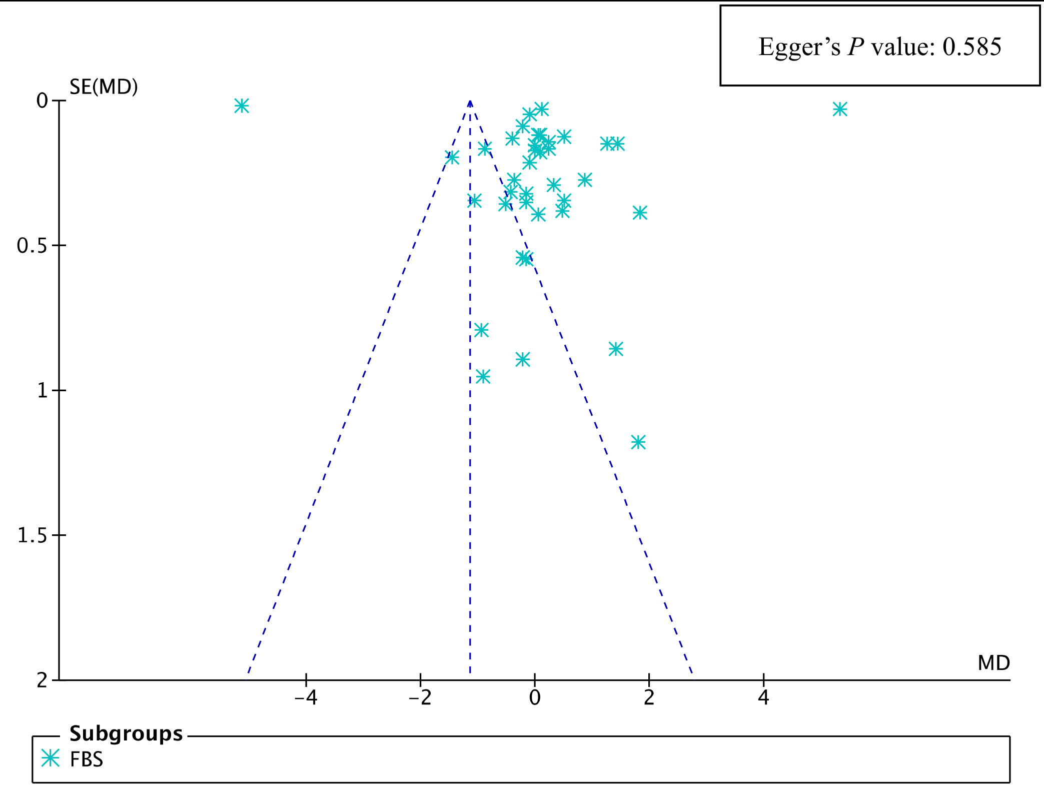

Figure S3-1-7 Funnel plot of RCTs investigating the effect of n3 fatty acids supplementation on FBG.

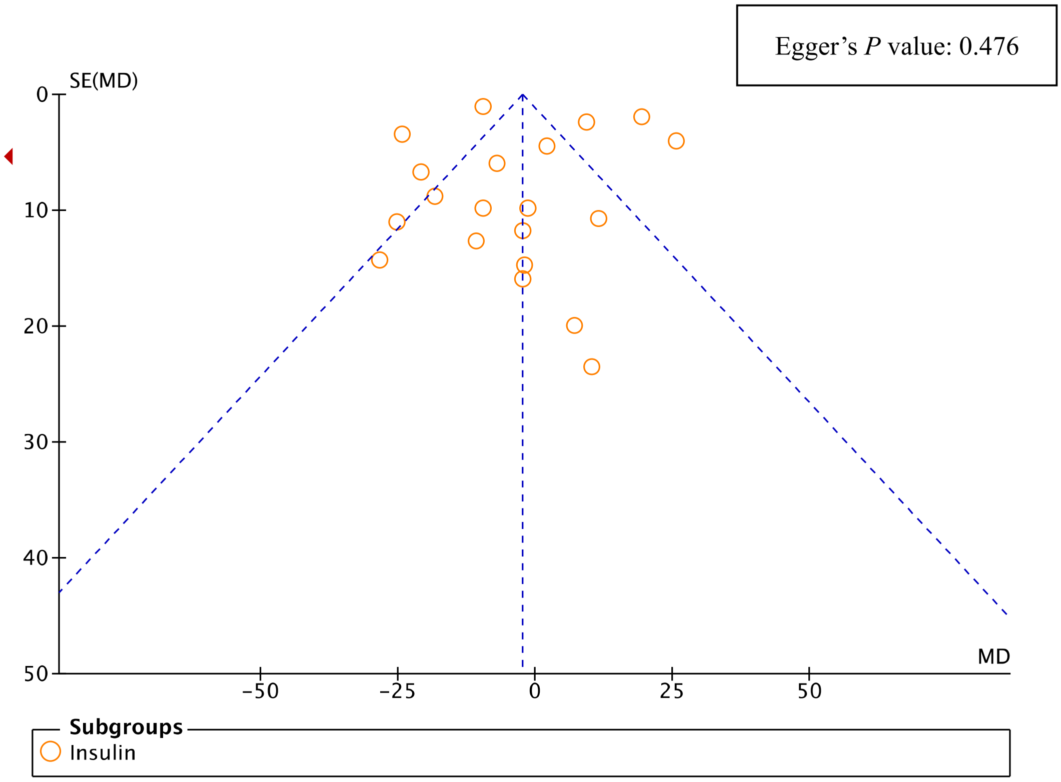

Figure S3-1-8 Funnel plot of RCTs investigating the effect of n3 fatty acids supplementation on FBI.

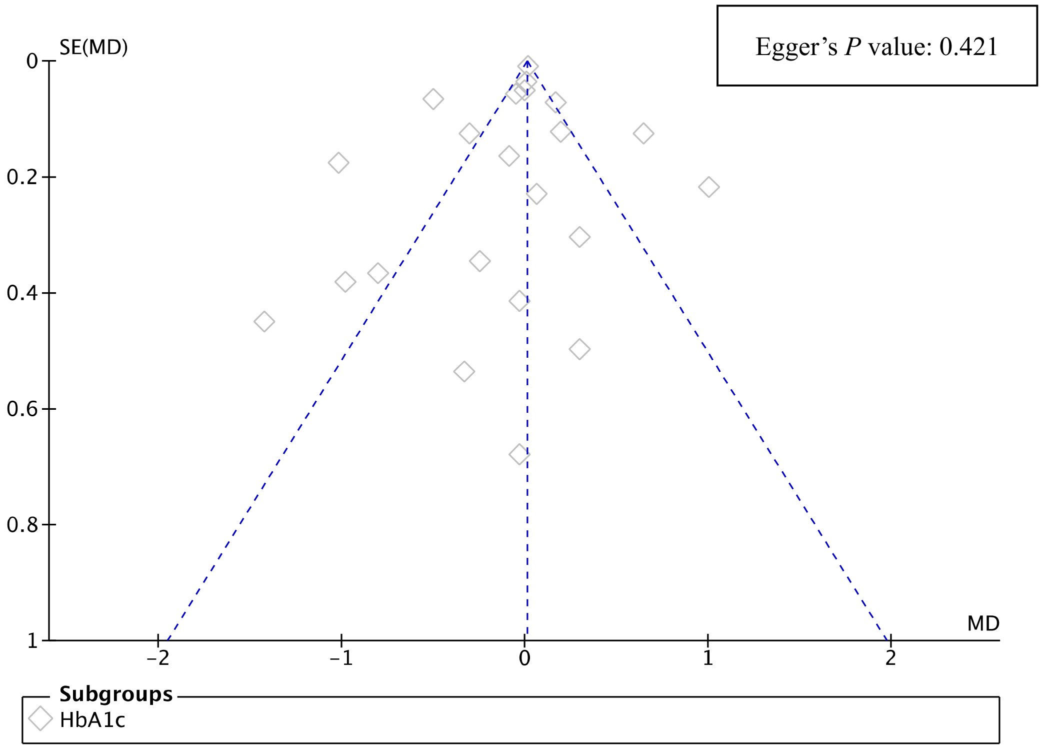

Figure S3-1-9 Funnel plot of RCTs investigating the effect of n3 fatty acids supplementation on A1C.

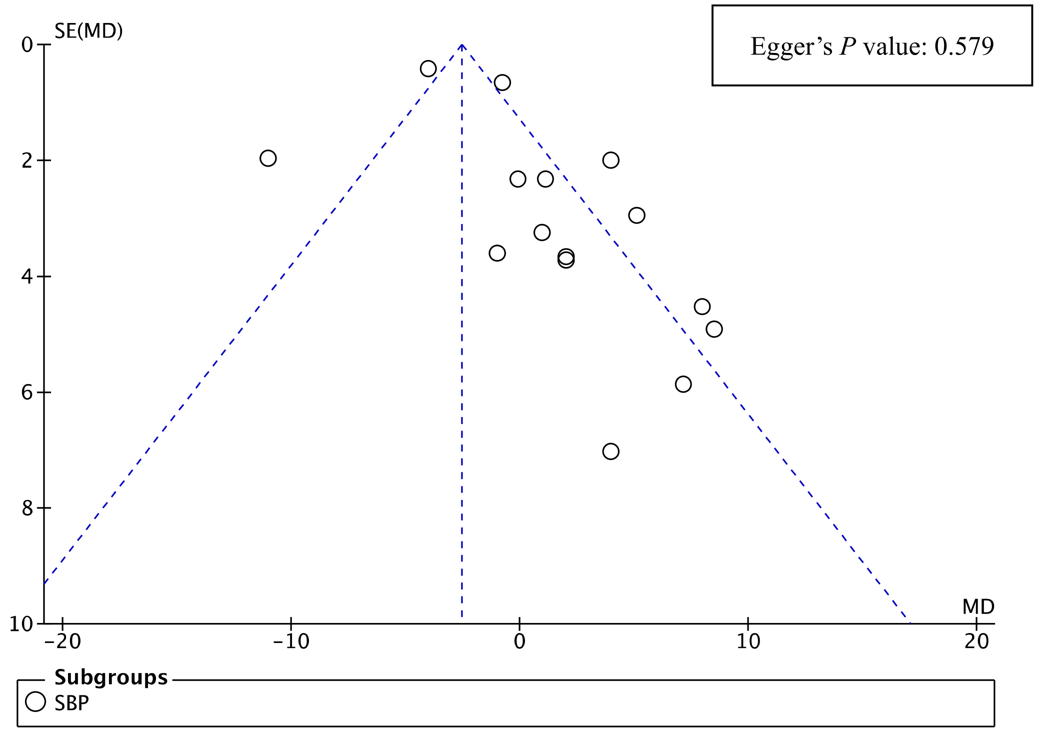

Figure S3-2-1 Funnel plot of RCTs investigating the effect of n6 fatty acids supplementation on SBP.

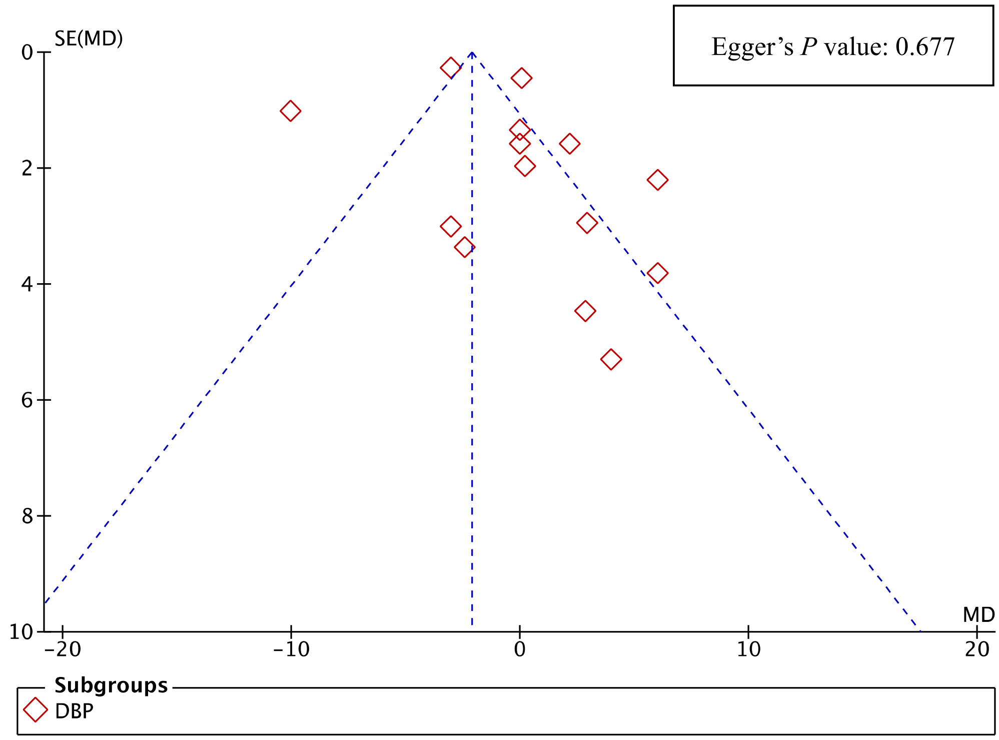

Figure S3-2-2 Funnel plot of RCTs investigating the effect of n6 fatty acids supplementation on DBP.

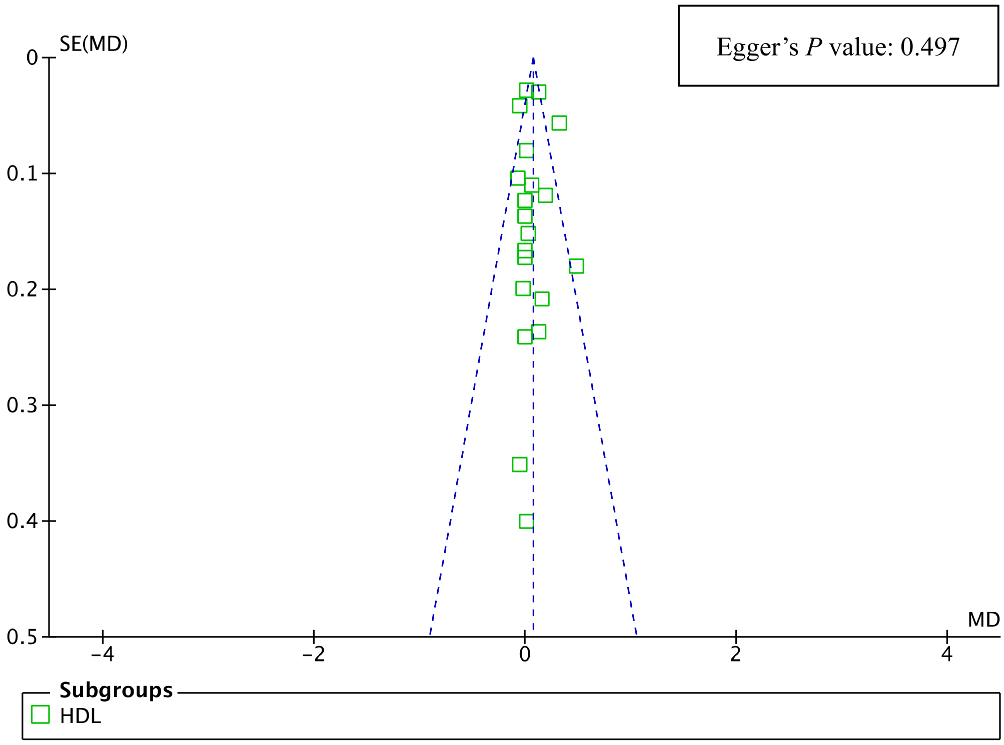

Figure S3-2-3 Funnel plot of RCTs investigating the effect of n6 fatty acids supplementation on HDL-C.

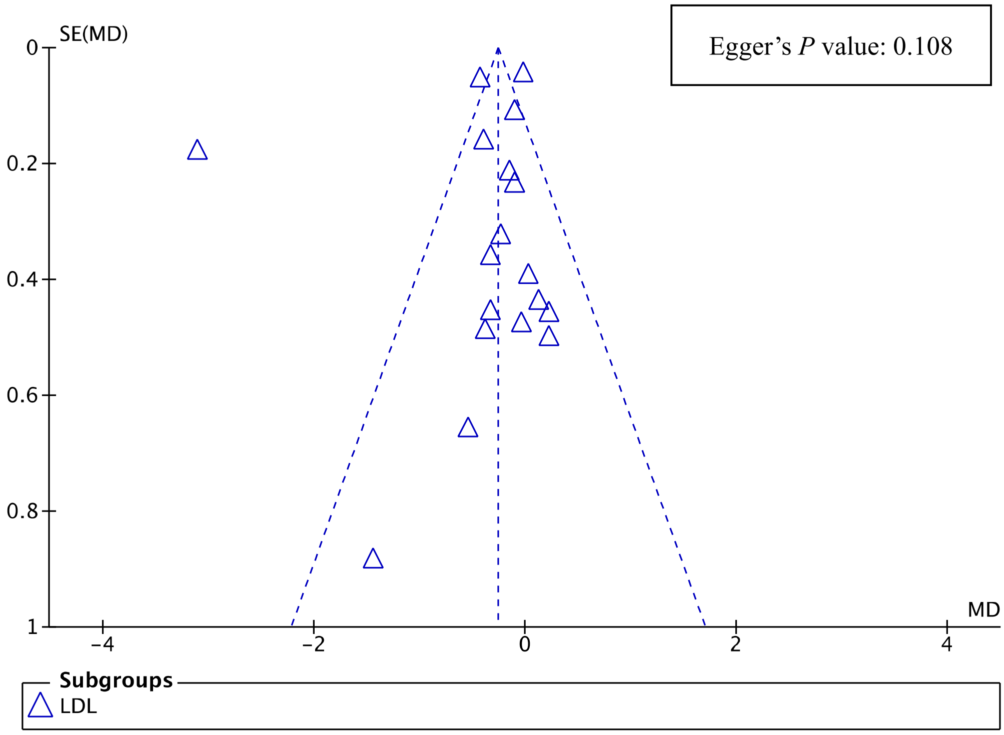

Figure S3-2-4 Funnel plot of RCTs investigating the effect of n6 fatty acids supplementation on LDL-C.

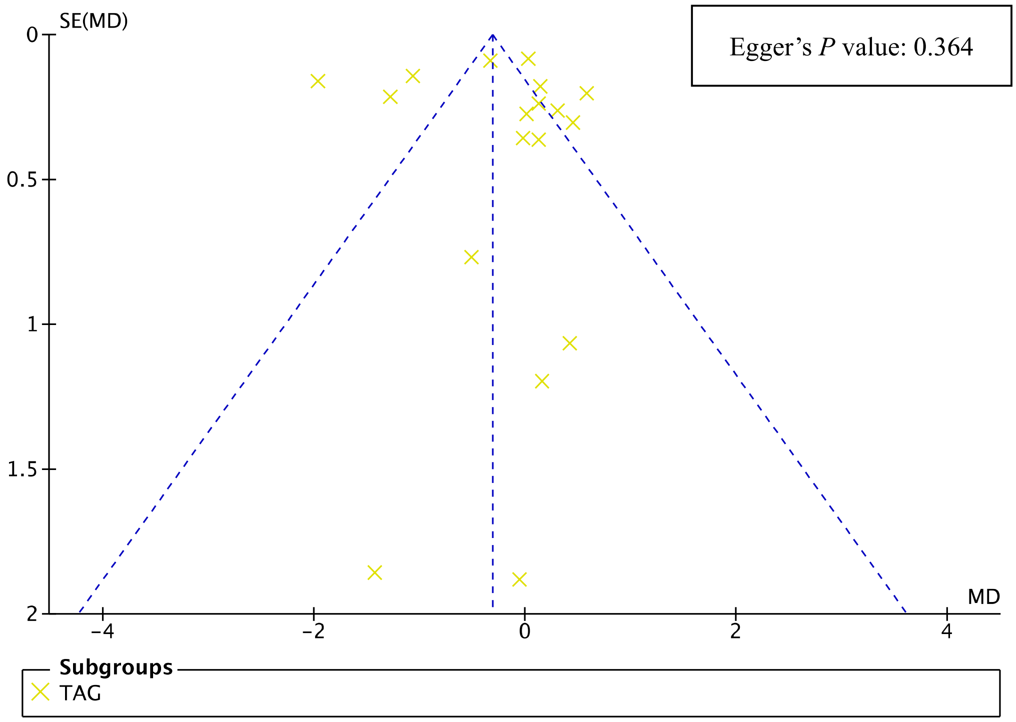

Figure S3-2-5 Funnel plot of RCTs investigating the effect of n6 fatty acids supplementation on TG

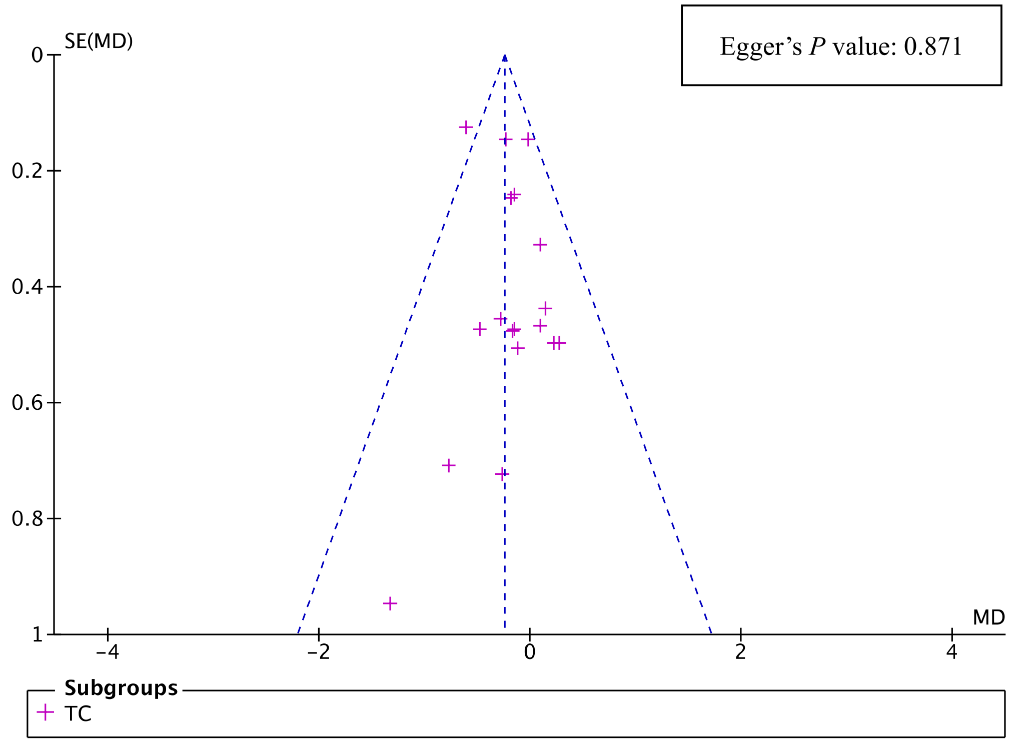

Figure S3-2-6 Funnel plot of RCTs investigating the effect of n6 fatty acids supplementation on TC.

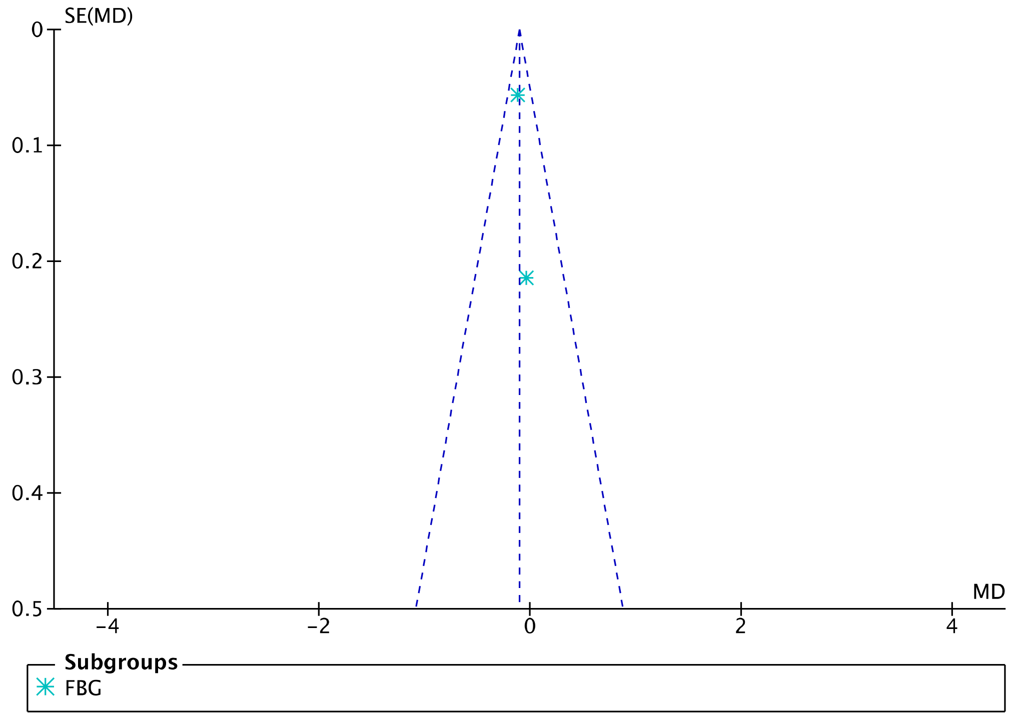

Figure S3-2-7 Funnel plot of RCTs investigating the effect of n6 fatty acids supplementation on FBG.

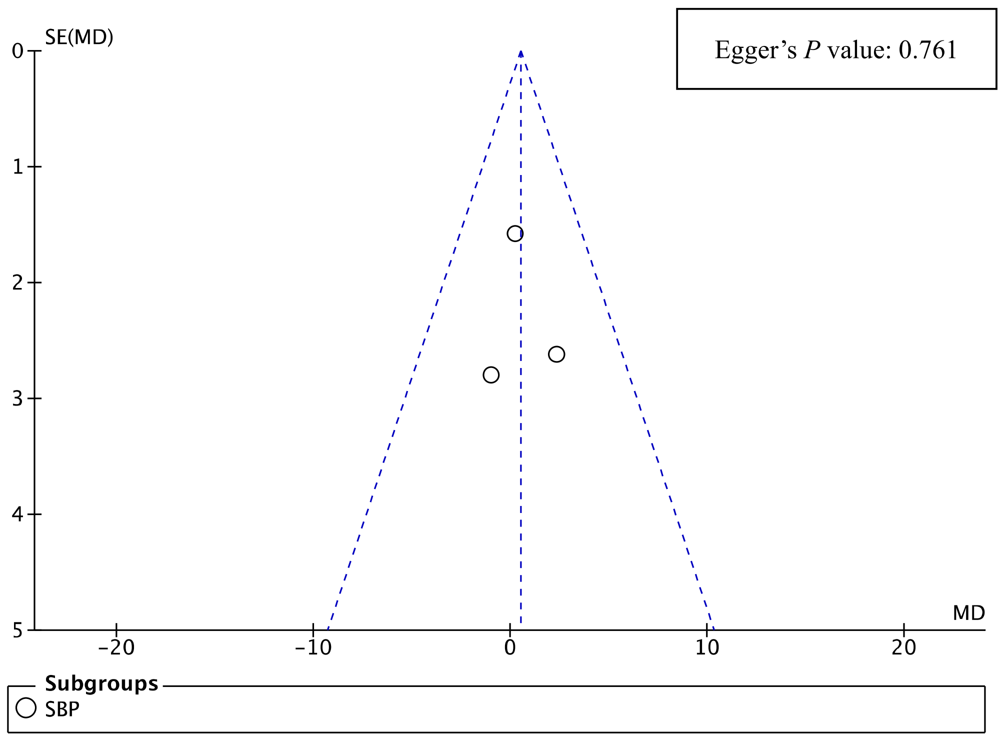

Figure S3-3-1 Funnel plot of RCTs investigating the effect of n9 fatty acids supplementation on SBP.

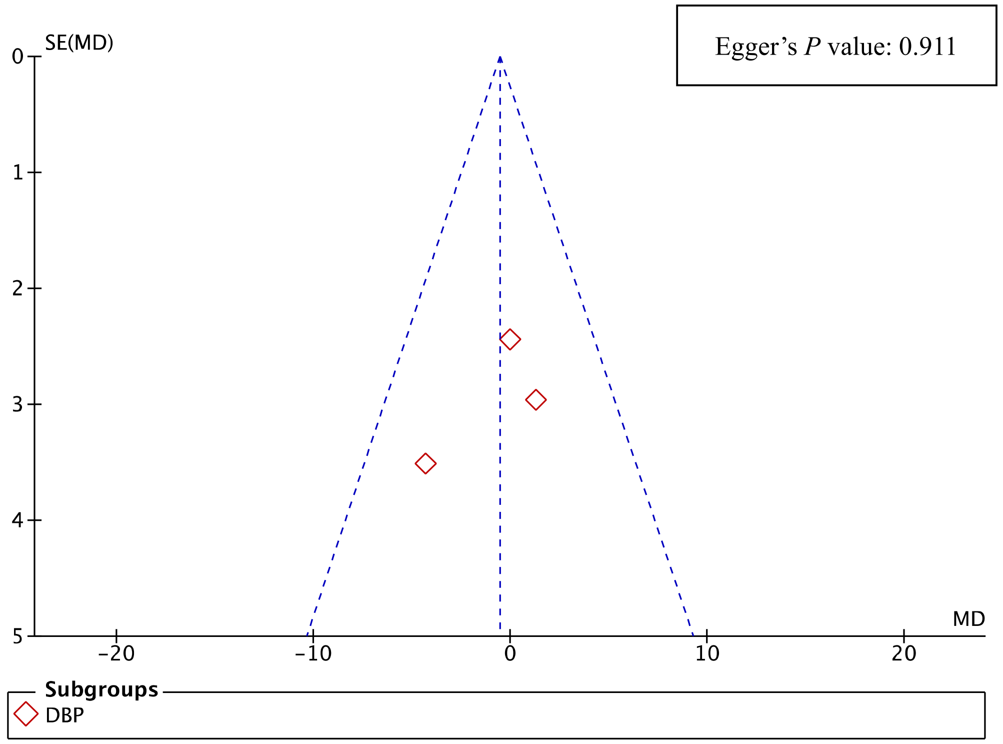

Figure S3-3-2 Funnel plot of RCTs investigating the effect of n9 fatty acids supplementation on DBP.

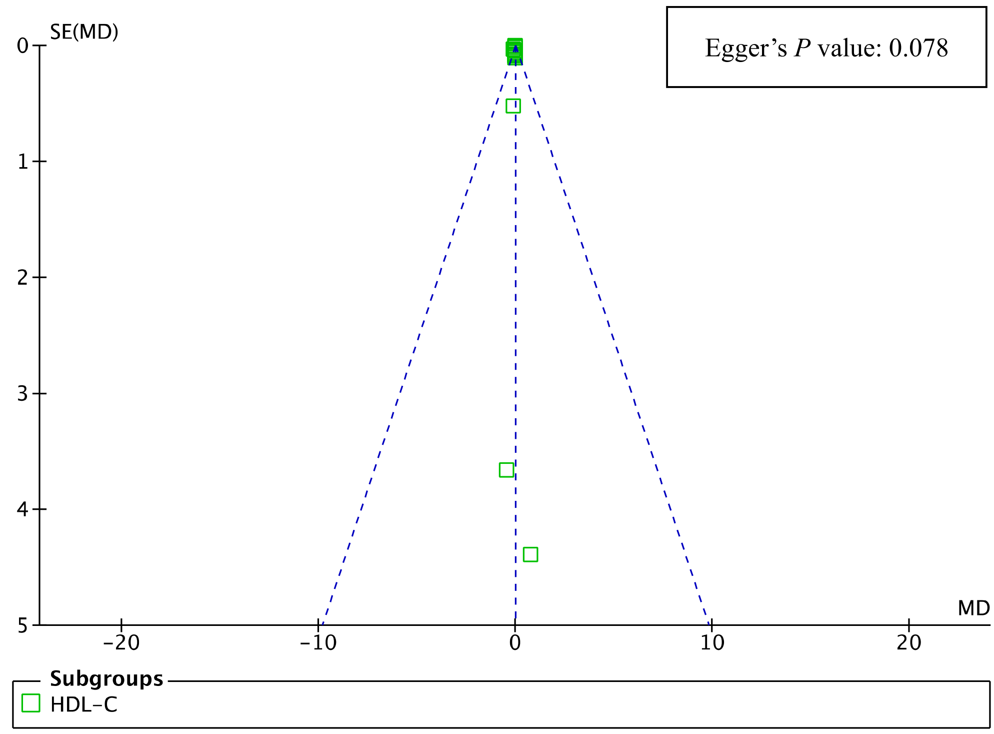

Figure S3-3-3 Funnel plot of RCTs investigating the effect of n9 fatty acids supplementation on HDL-C.

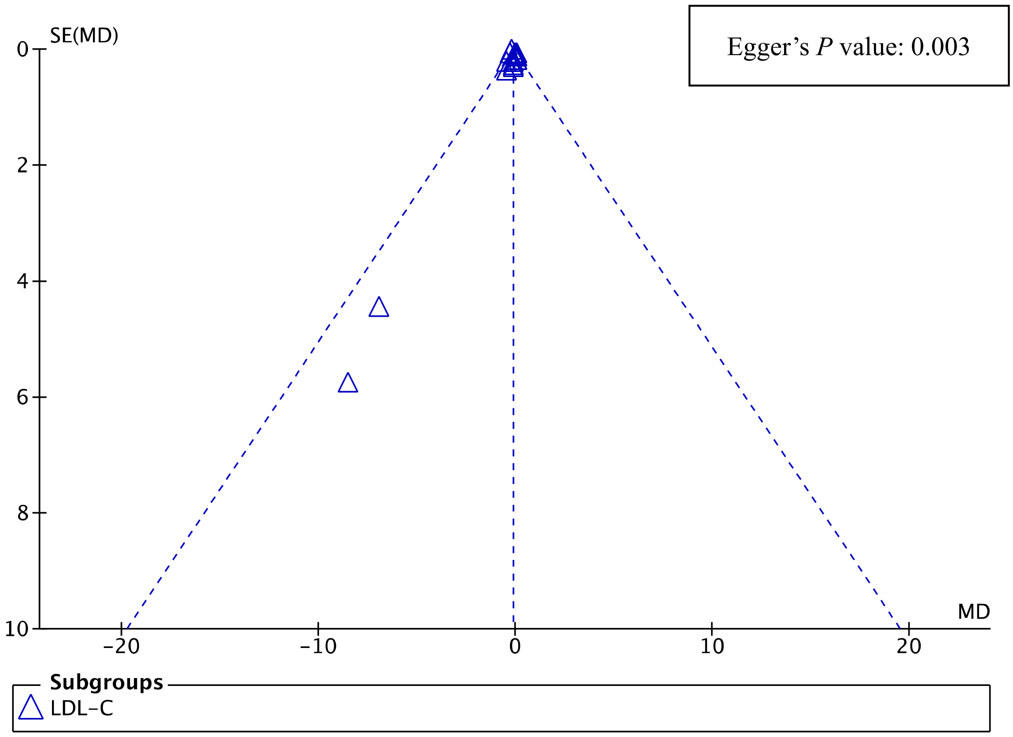

Figure S3-3-4 Funnel plot of RCTs investigating the effect of n9 fatty acids supplementation on LDL-C.

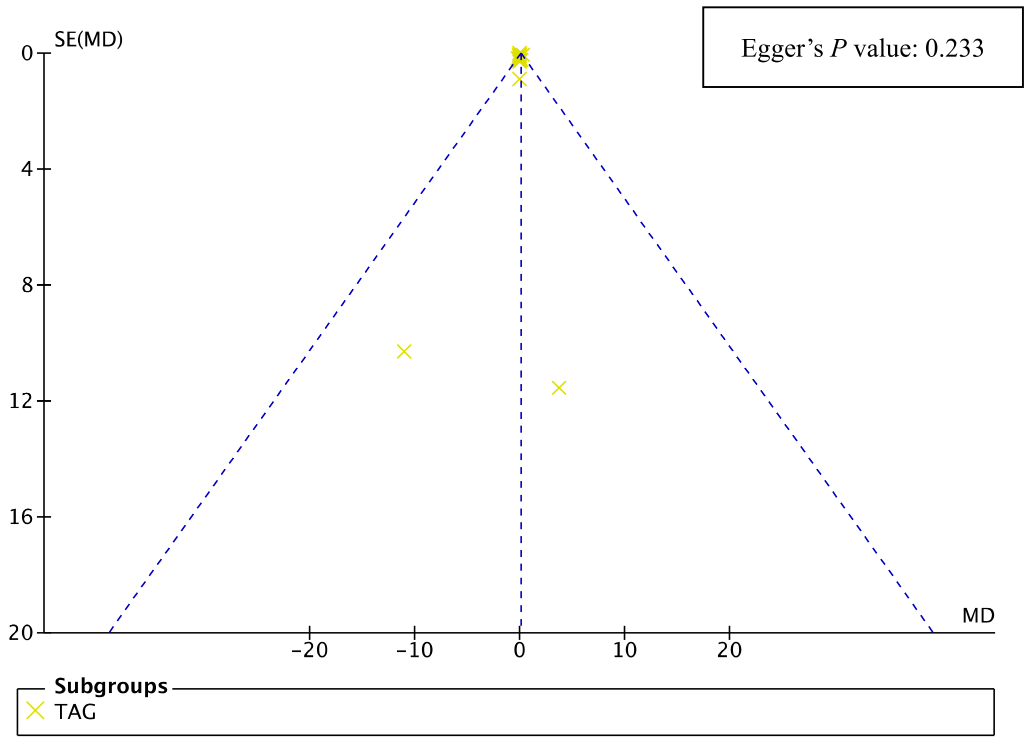

Figure S3-3-5 Funnel plot of RCTs investigating the effect of n9 fatty acids supplementation on TG

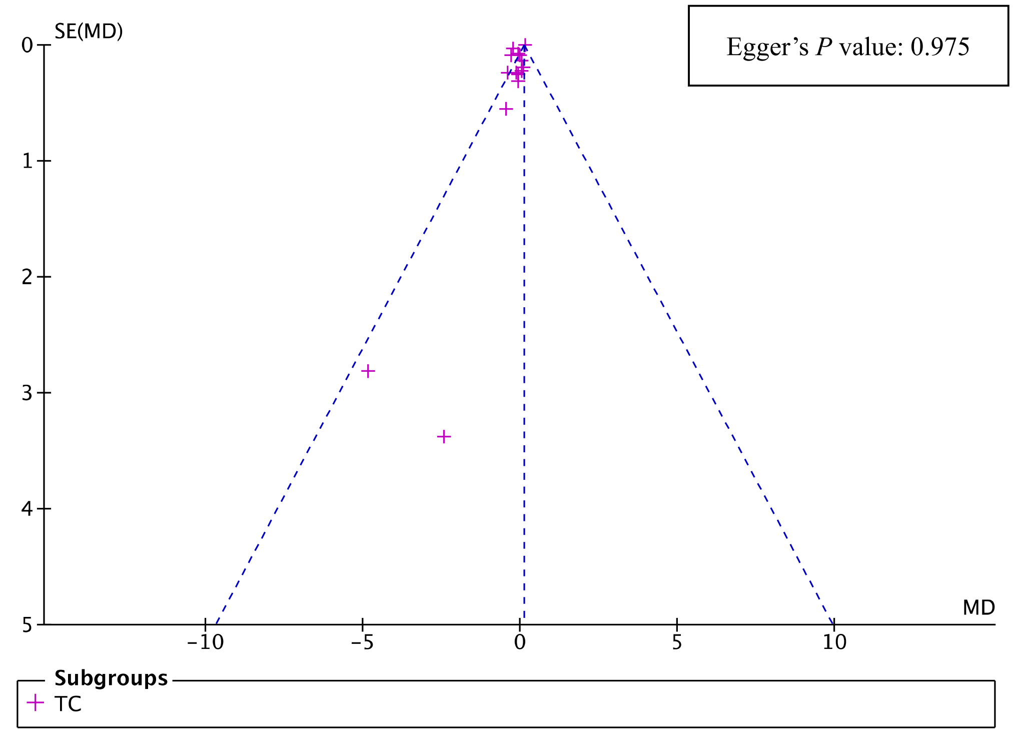

Figure S3-3-6 Funnel plot of RCTs investigating the effect of n9 fatty acids supplementation on TC.

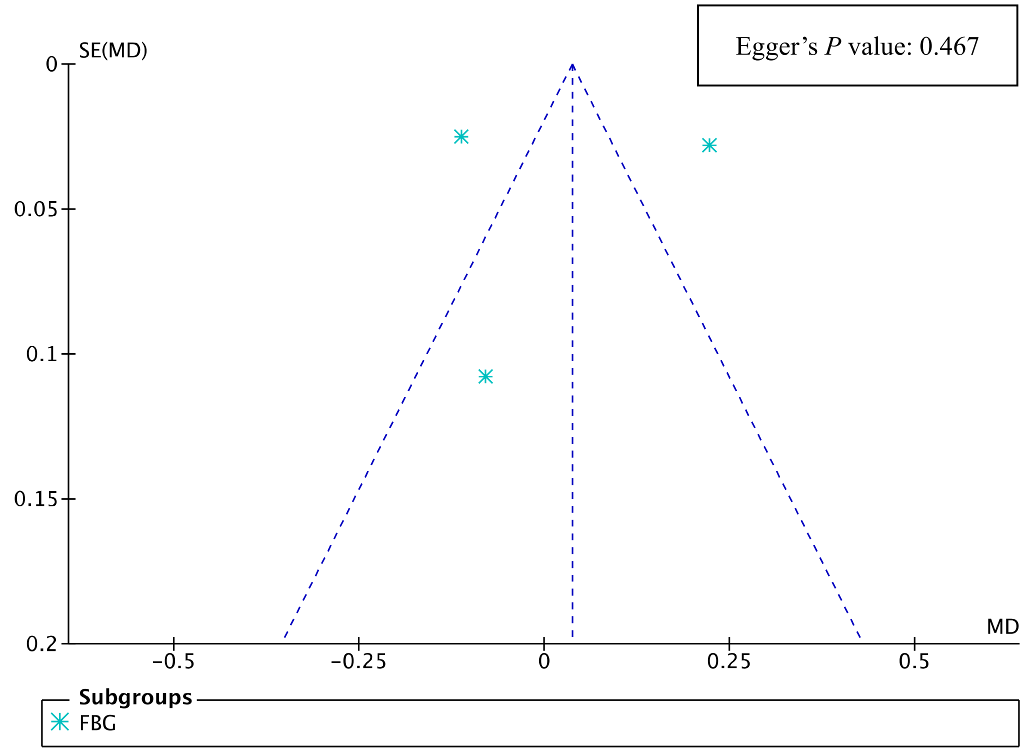

Figure S3-3-7 Funnel plot of RCTs investigating the effect of n9 fatty acids supplementation on FBG.

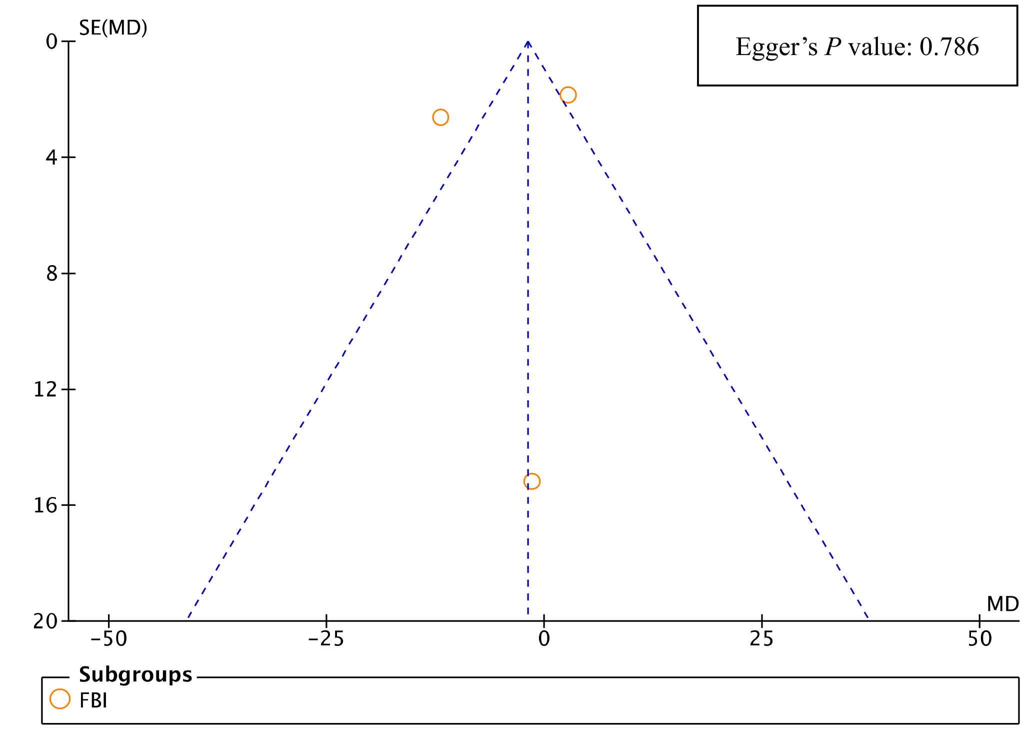

Figure S3-3-8 Funnel plot of RCTs investigating the effect of n9 fatty acids supplementation on FBI.

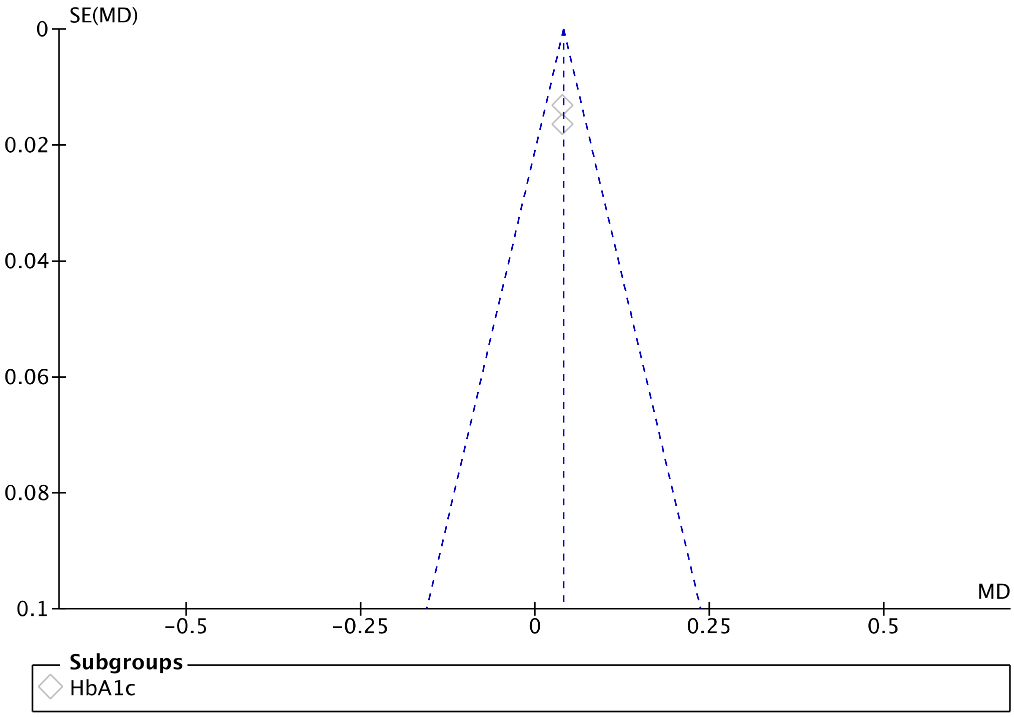

Figure S3-3-9 Funnel plot of RCTs investigating the effect of n9 fatty acids supplementation on A1C.

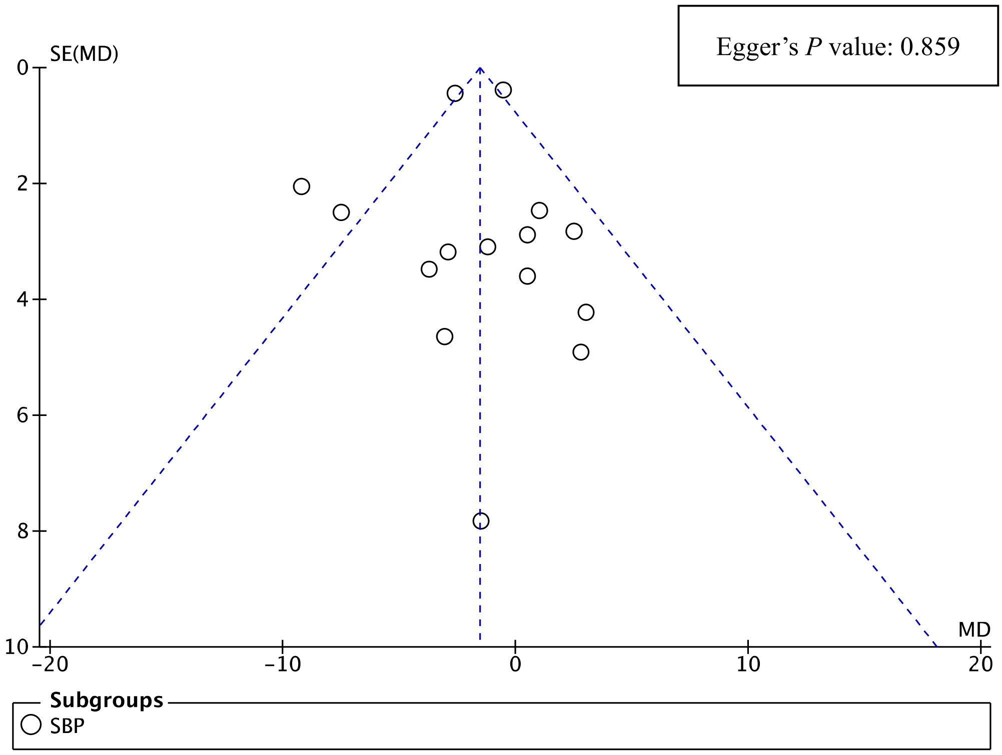

Figure S3-4-1 Funnel plot of RCTs investigating the effect of lycopene supplementation on SBP.

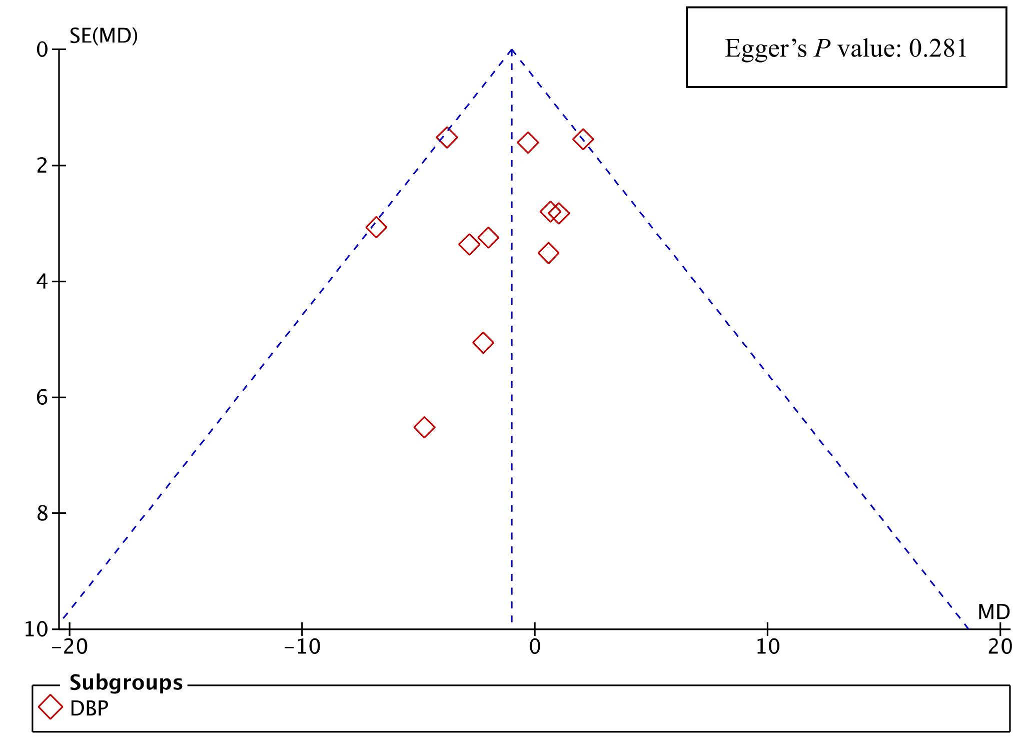

Figure S3-4-2 Funnel plot of RCTs investigating the effect of lycopene supplementation on DBP.

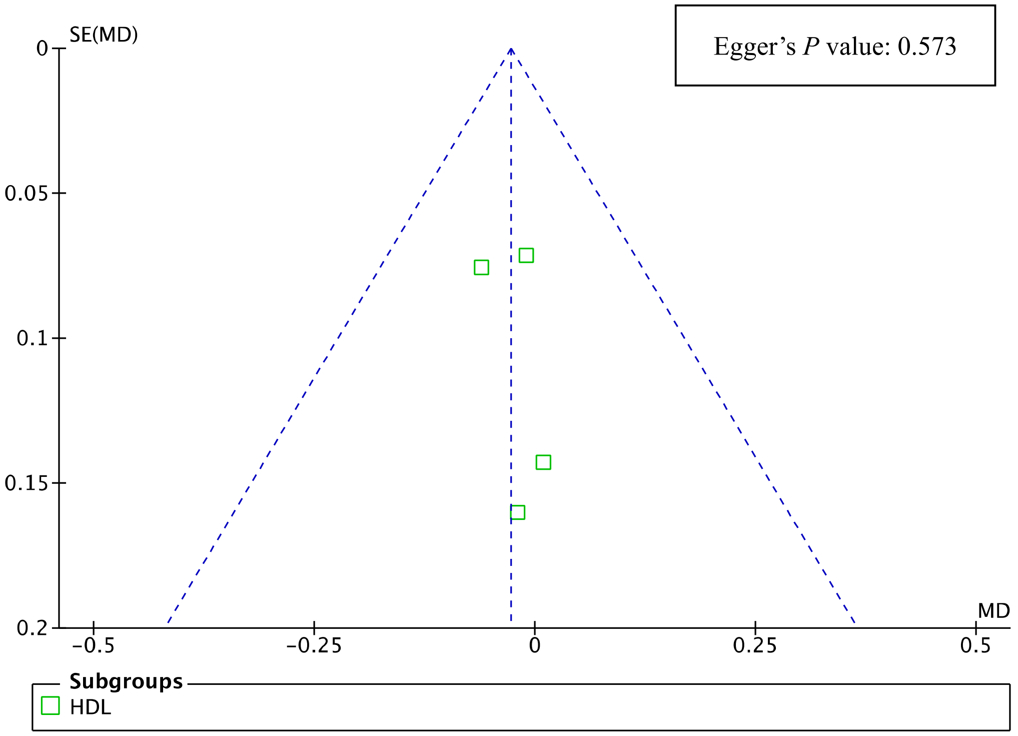

Figure S3-4-3 Funnel plot of RCTs investigating the effect of lycopene supplementation on HDL-C.

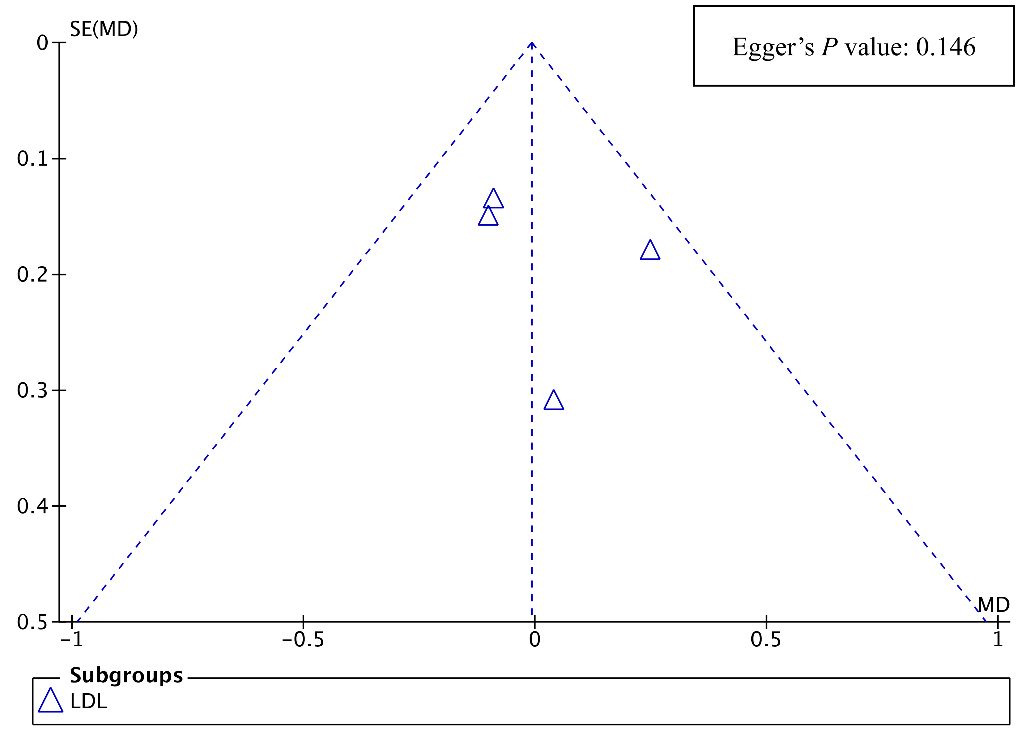

Figure S3-4-4 Funnel plot of RCTs investigating the effect of lycopene supplementation on LDL-C.

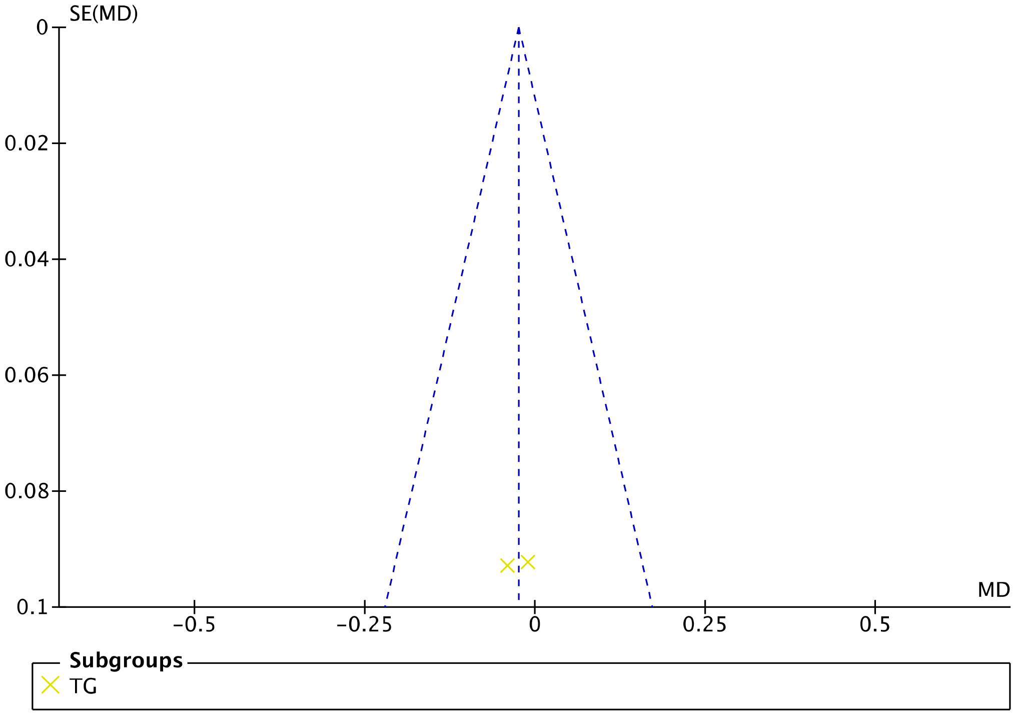

Figure S3-4-5 Funnel plot of RCTs investigating the effect of lycopene supplementation on TG

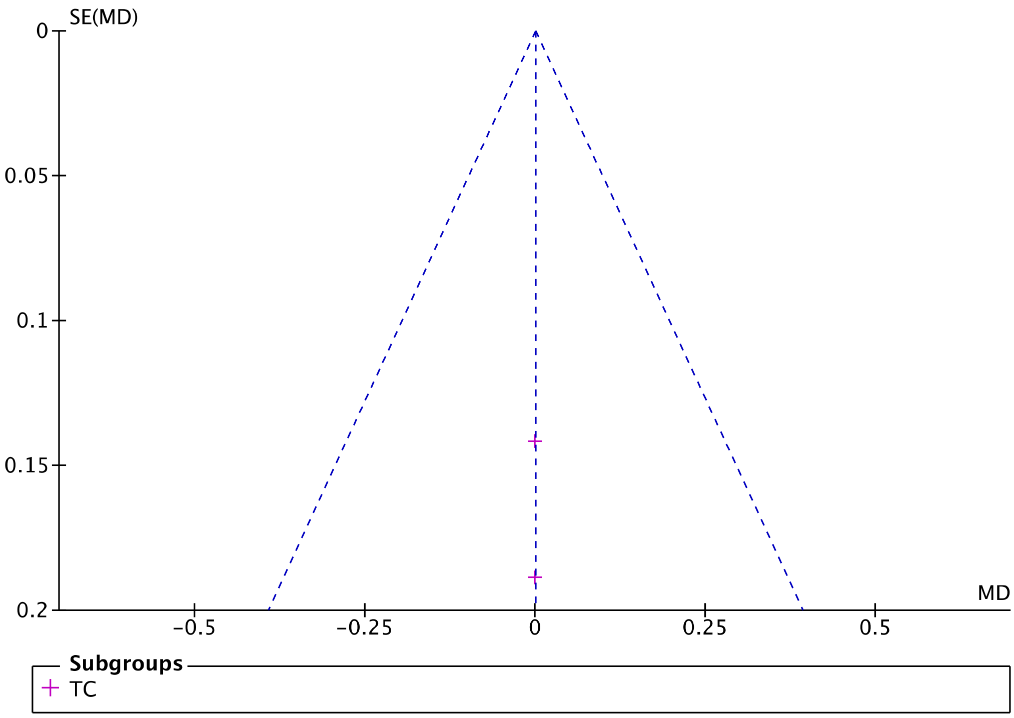

Figure S3-4-6 Funnel plot of RCTs investigating the effect of lycopene supplementation on TC.

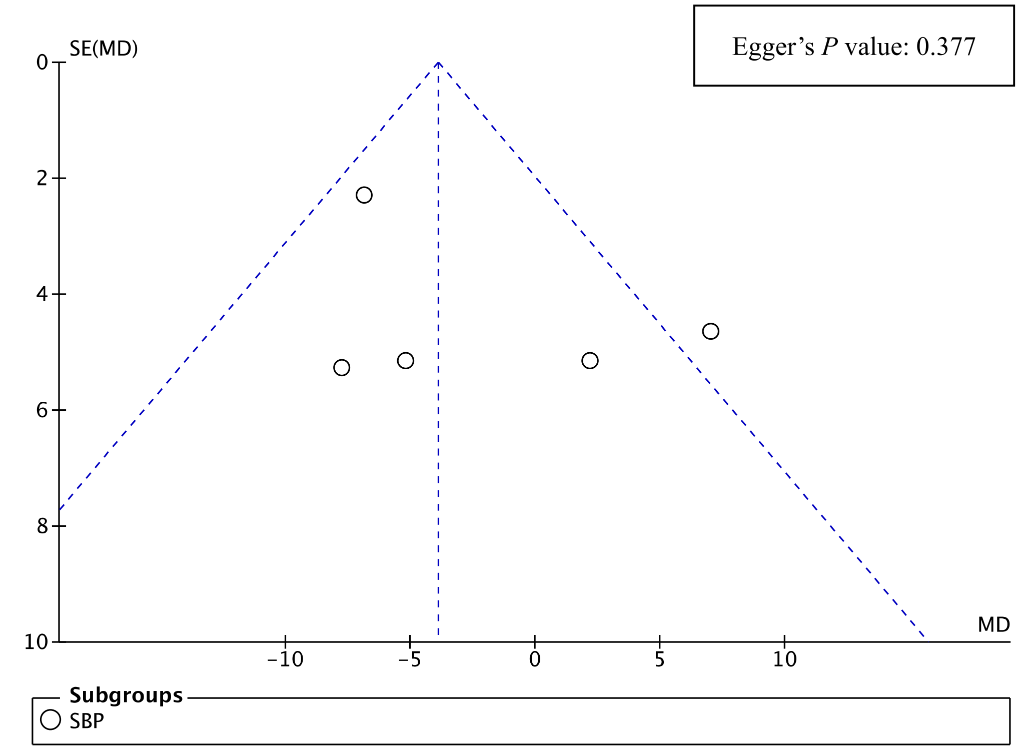

Figure S3-5-1 Funnel plot of RCTs investigating the effect of astaxanthin supplementation on SBP.

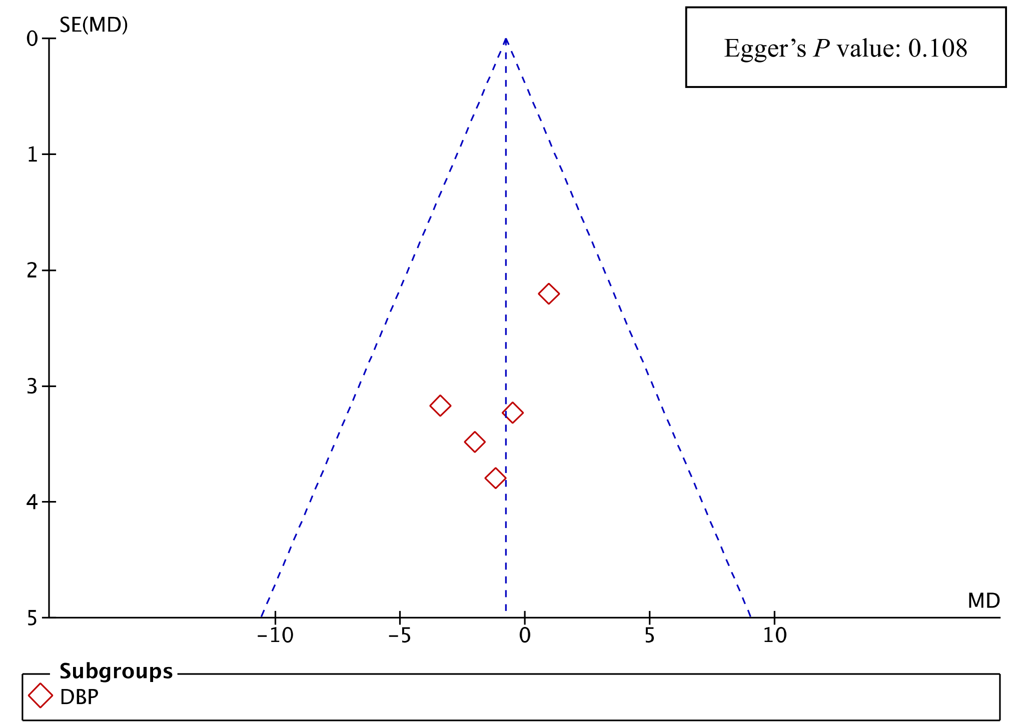

Figure S3-5-2 Funnel plot of RCTs investigating the effect of astaxanthin supplementation on DBP.

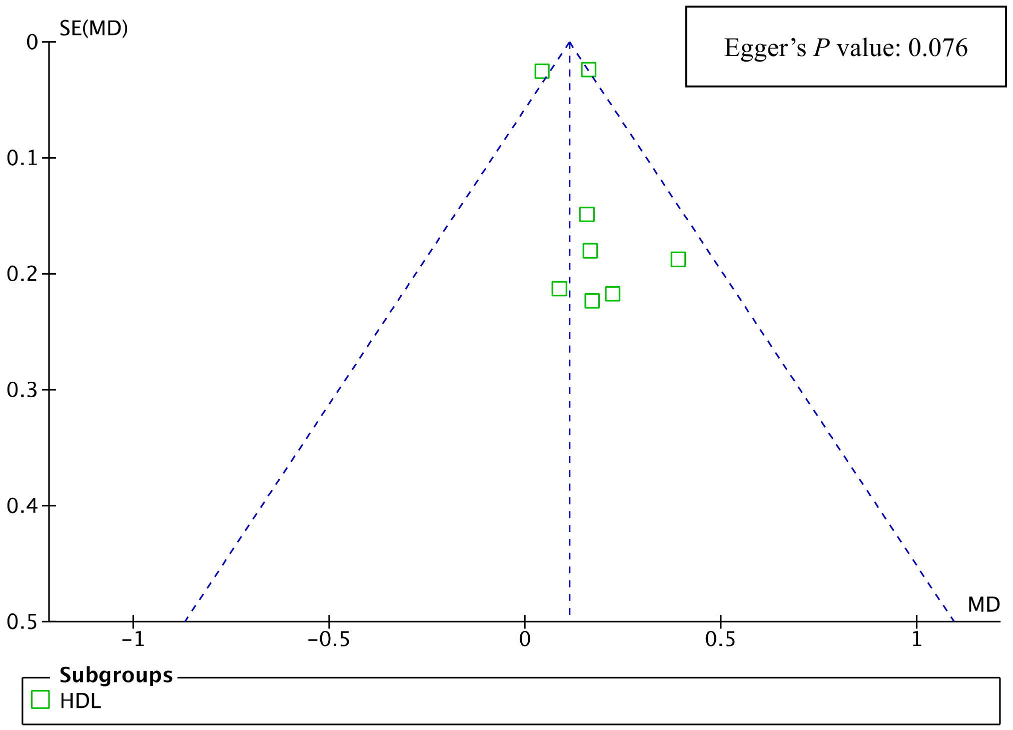

Figure S3-5-3 Funnel plot of RCTs investigating the effect of astaxanthin supplementation on HDL-C.

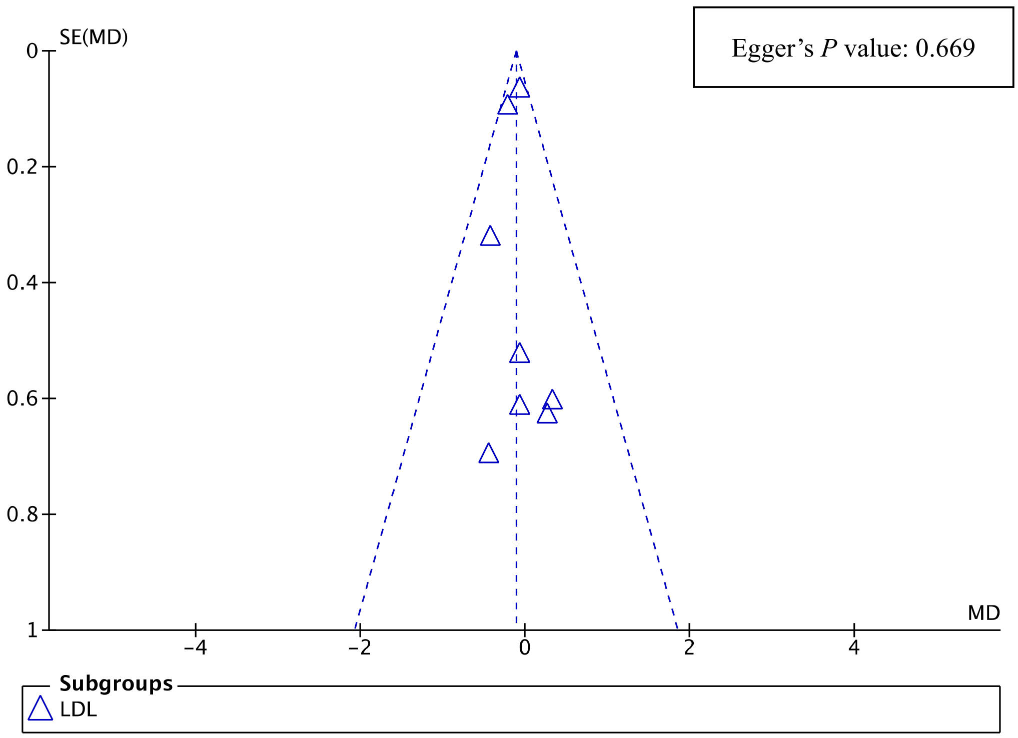

Figure S3-5-4 Funnel plot of RCTs investigating the effect of astaxanthin supplementation on LDL-C.

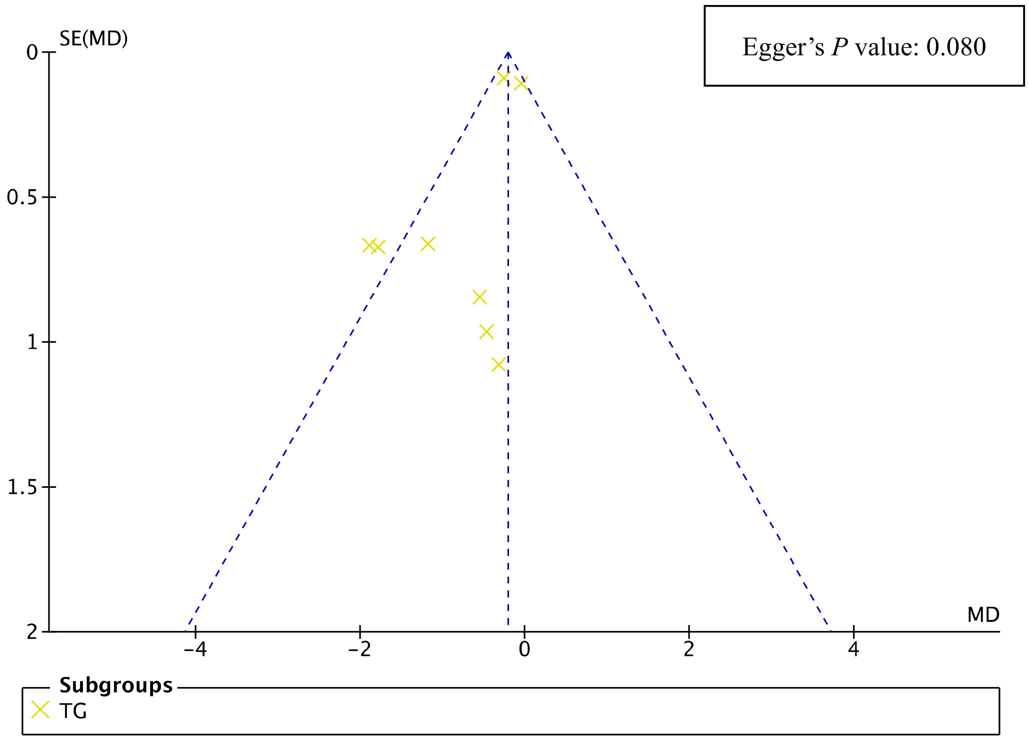

Figure S3-5-5 Funnel plot of RCTs investigating the effect of astaxanthin supplementation on TG

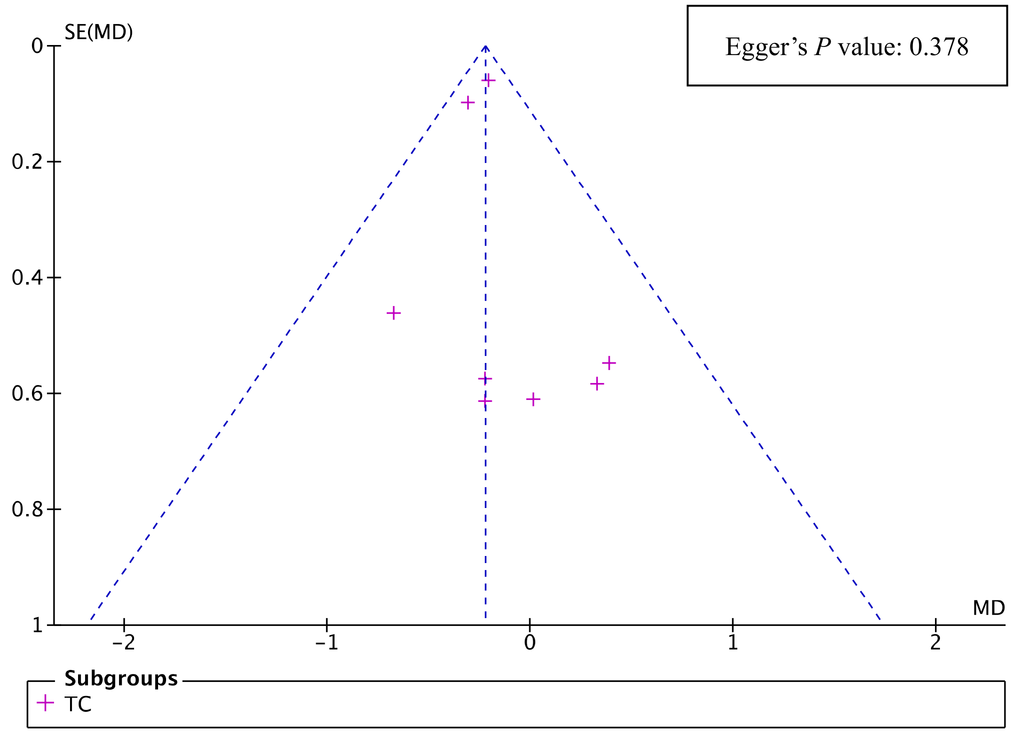

Figure S3-5-6 Funnel plot of RCTs investigating the effect of astaxanthin supplementation on TC.

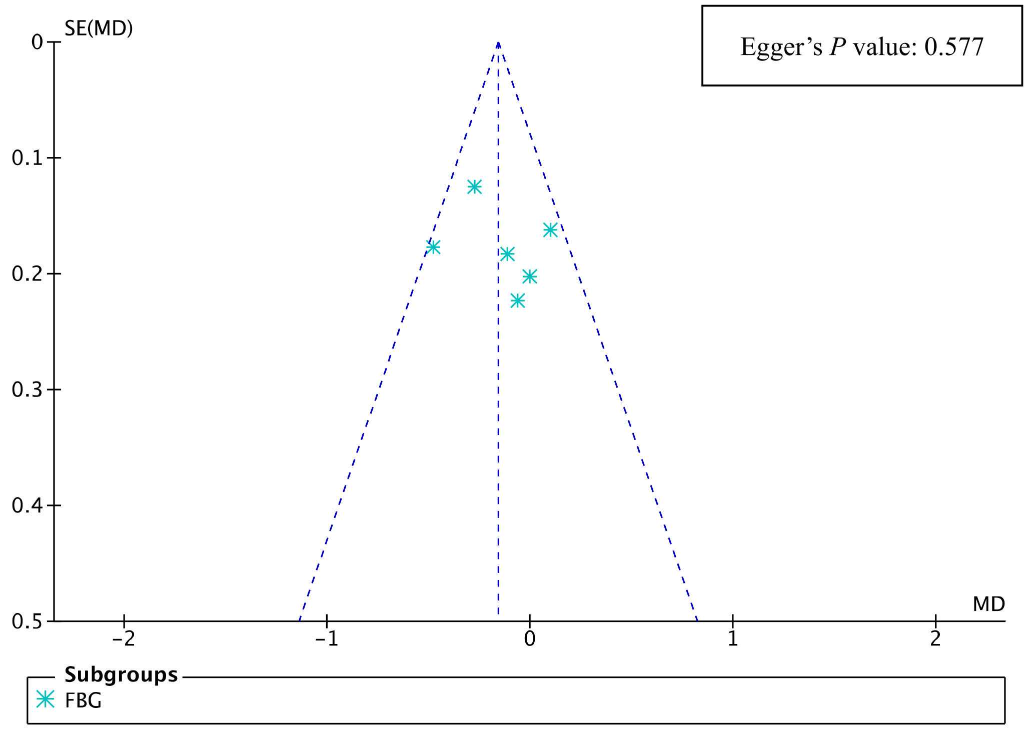

Figure S3-5-7 Funnel plot of RCTs investigating the effect of astaxanthin supplementation on FBG.

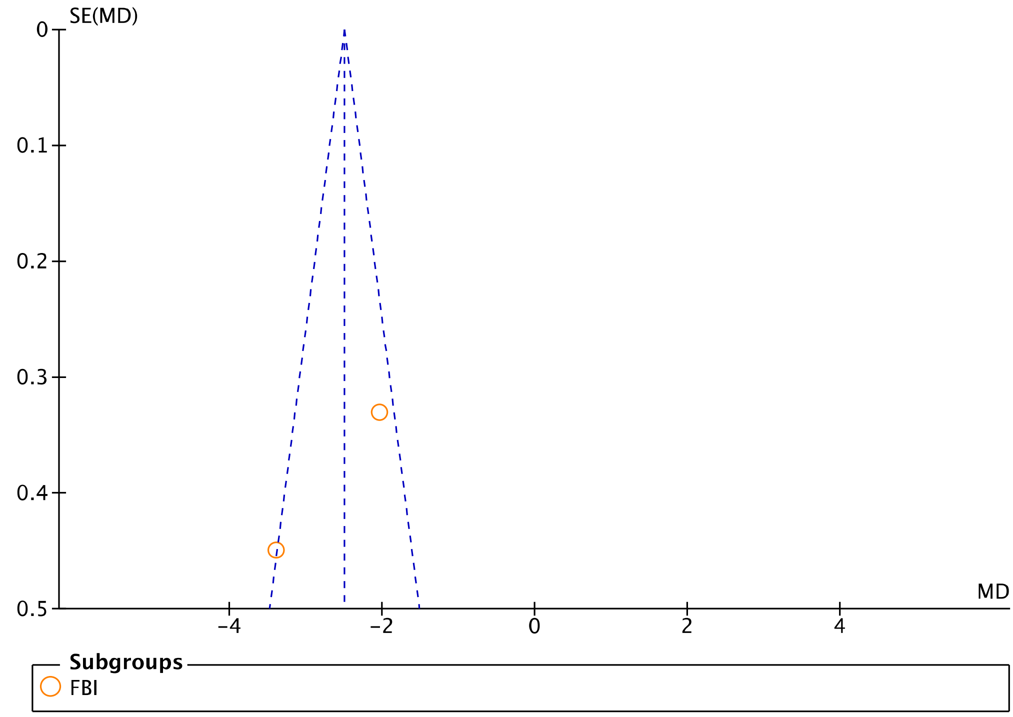

Figure S3-5-8 Funnel plot of RCTs investigating the effect of astaxanthin supplementation on FBI.

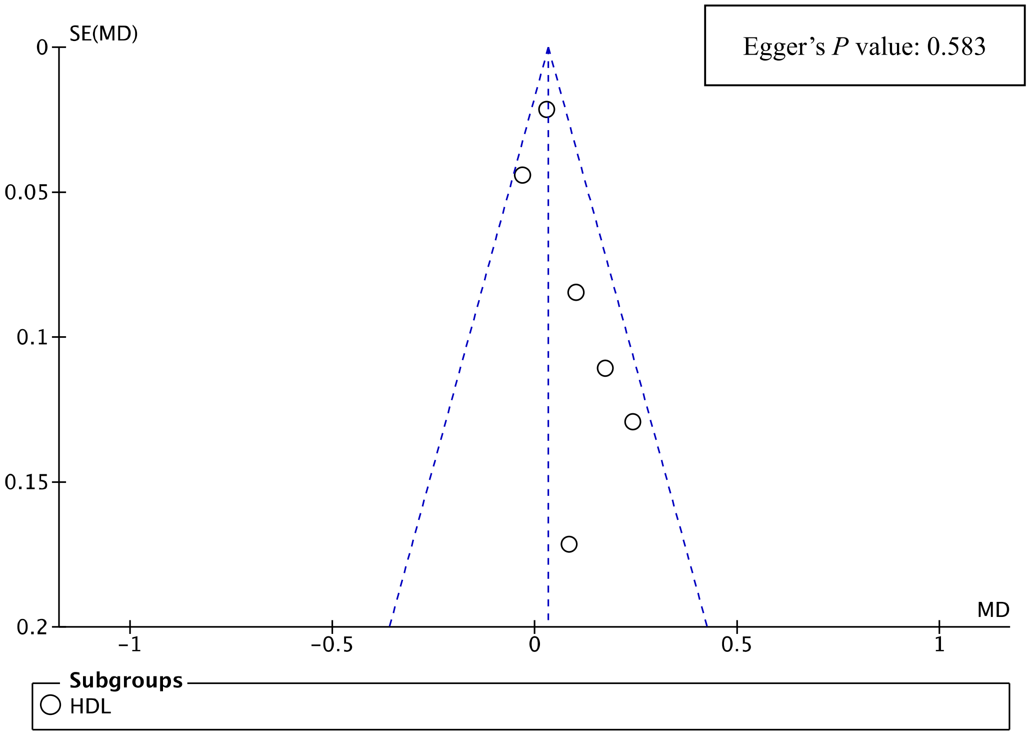

Figure S3-6-1 Funnel plot of RCTs investigating the effect of beta-carotene supplementation on HDL-C.

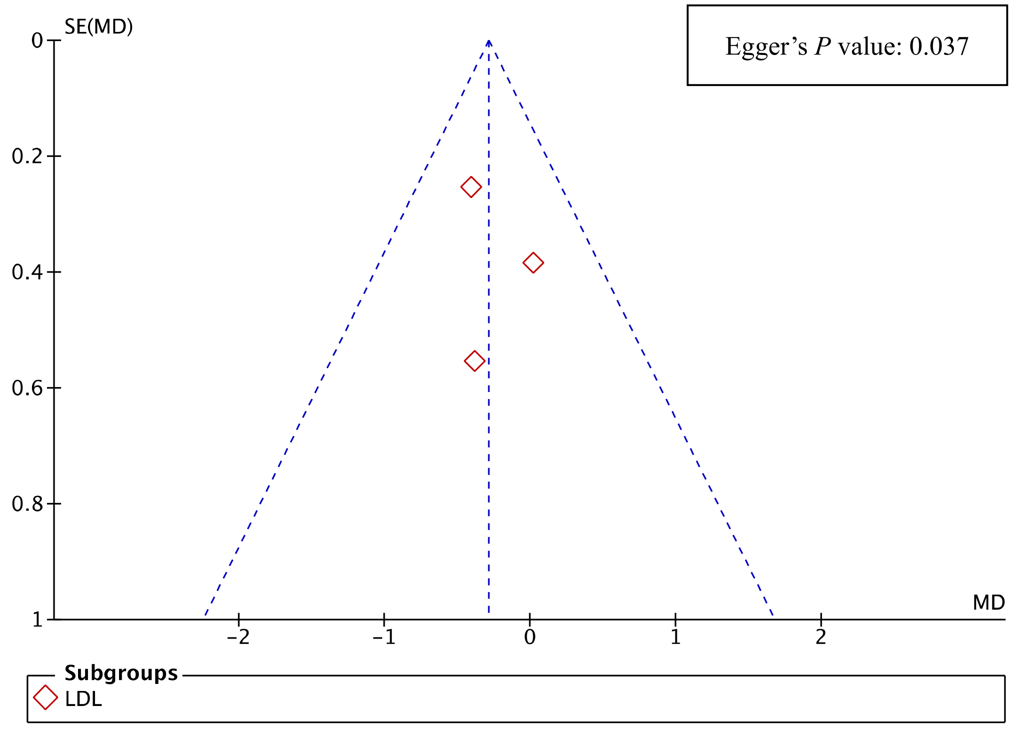

Figure S3-6-2 Funnel plot of RCTs investigating the effect of beta-carotene supplementation on LDL-C.

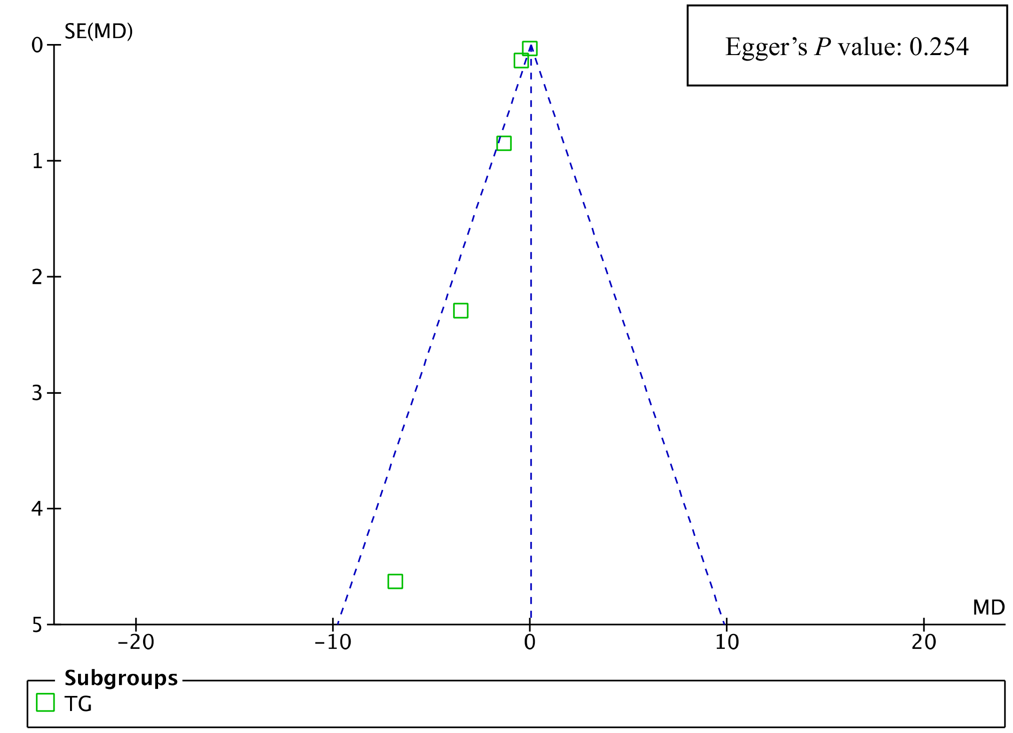

Figure S3-6-3 Funnel plot of RCTs investigating the effect of beta-carotene supplementation on TG

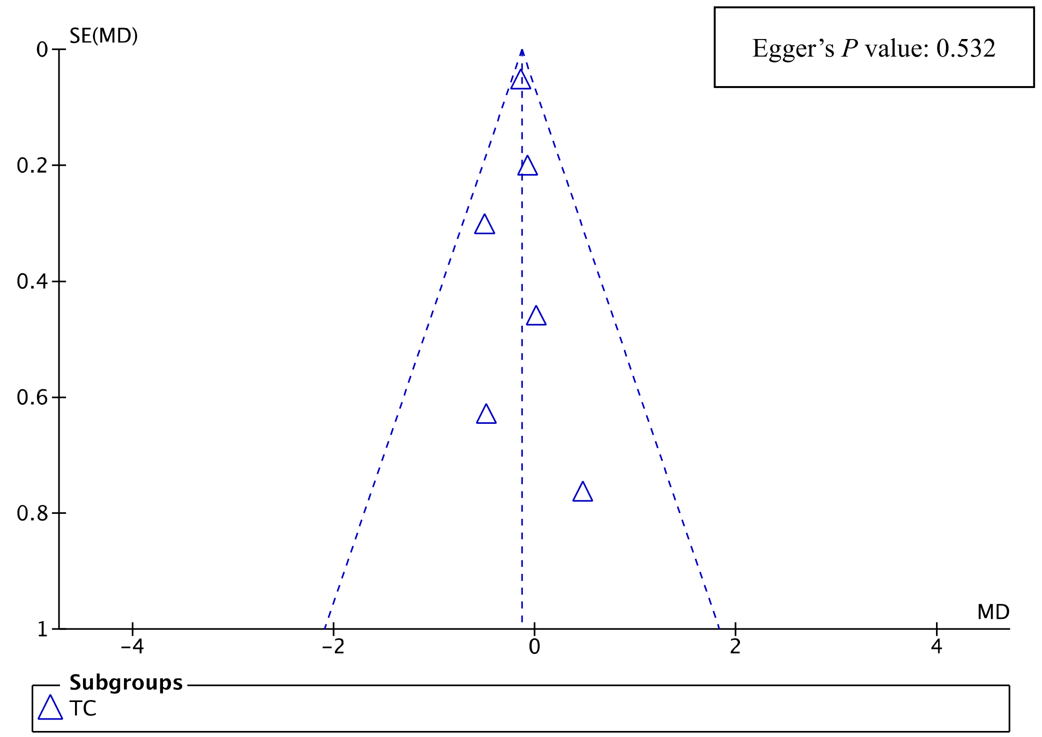

Figure S3-6-4 Funnel plot of RCTs investigating the effect of beta-carotene supplementation on TC.

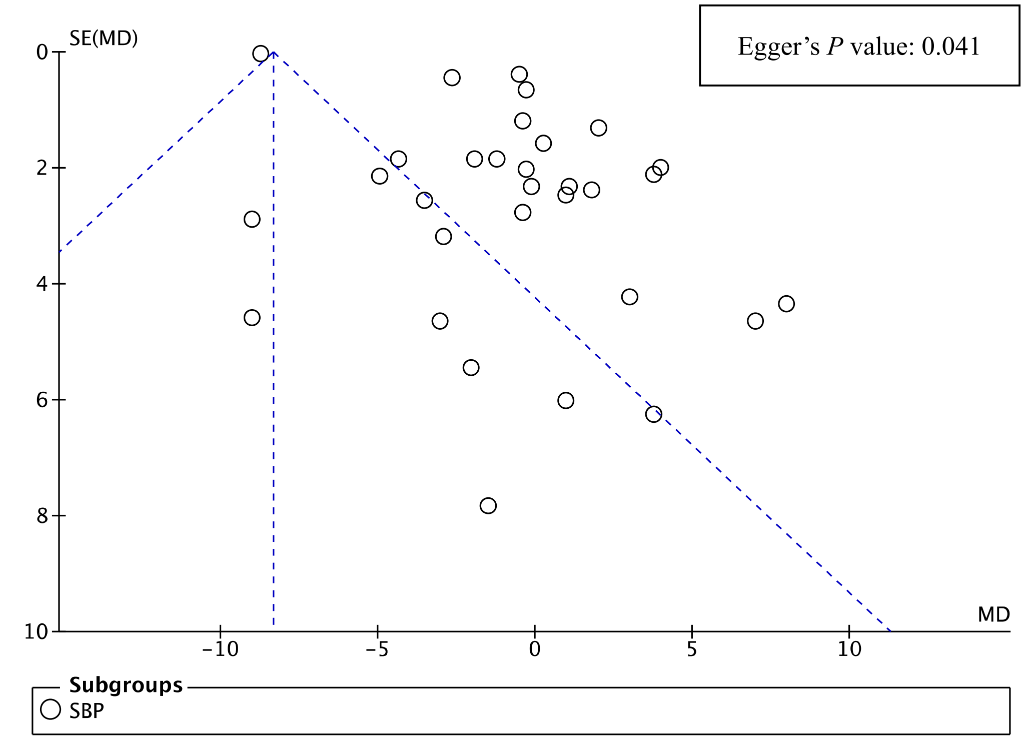

Figure S3-7-1 Funnel plot of RCTs investigating the effect of antioxidant fatty acids supplementation on SBP in healthy population.

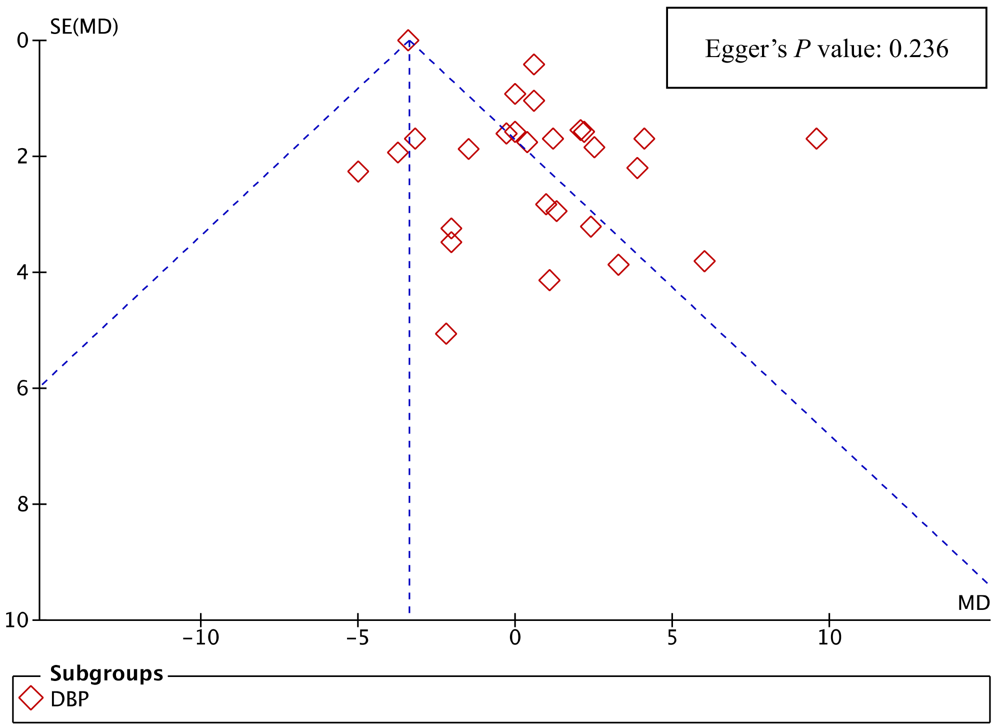

Figure S3-7-2 Funnel plot of RCTs investigating the effect of antioxidant fatty acids supplementation on DBP in healthy population.

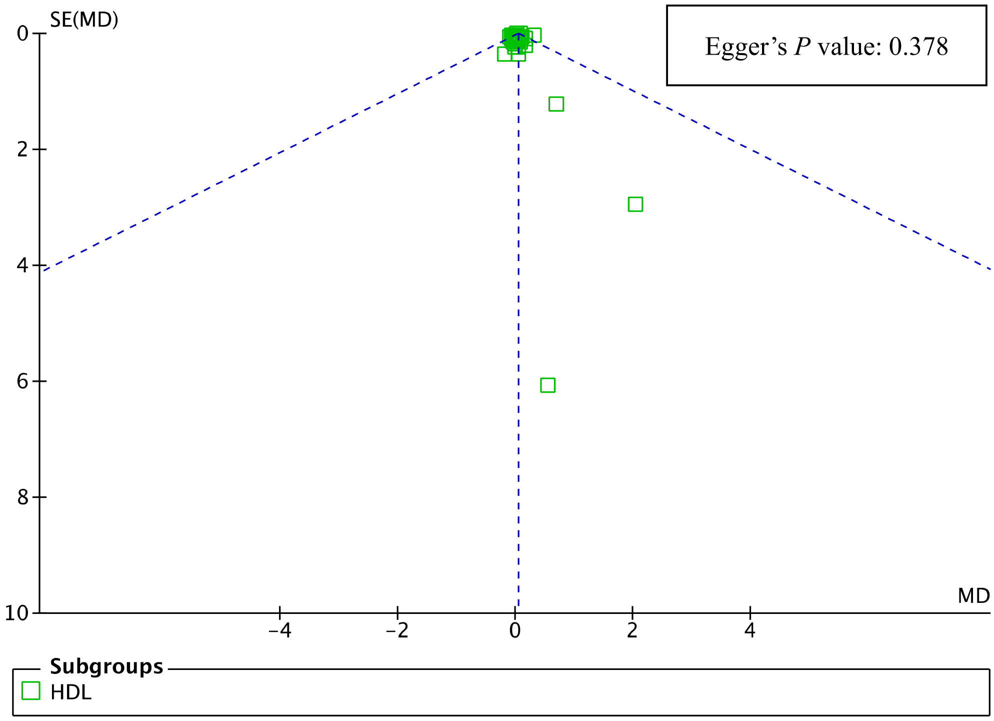

Figure S3-7-3 Funnel plot of RCTs investigating the effect of antioxidant fatty acids supplementation on HDL in healthy population.

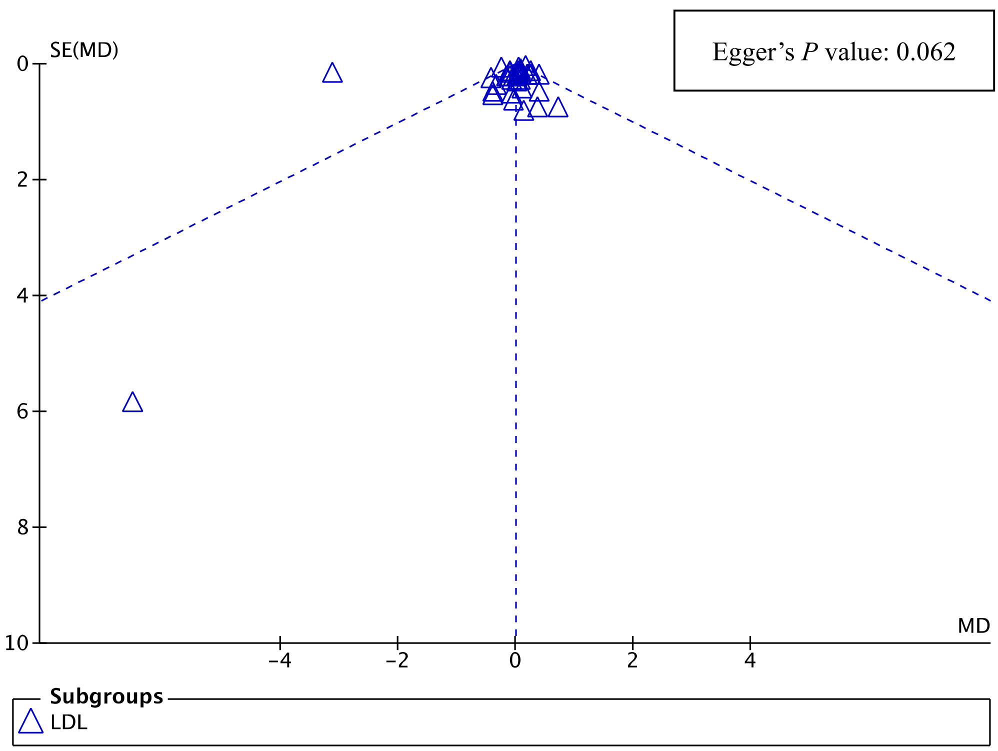

Figure S3-7-4 Funnel plot of RCTs investigating the effect of antioxidant fatty acids supplementation on LDL in healthy population.

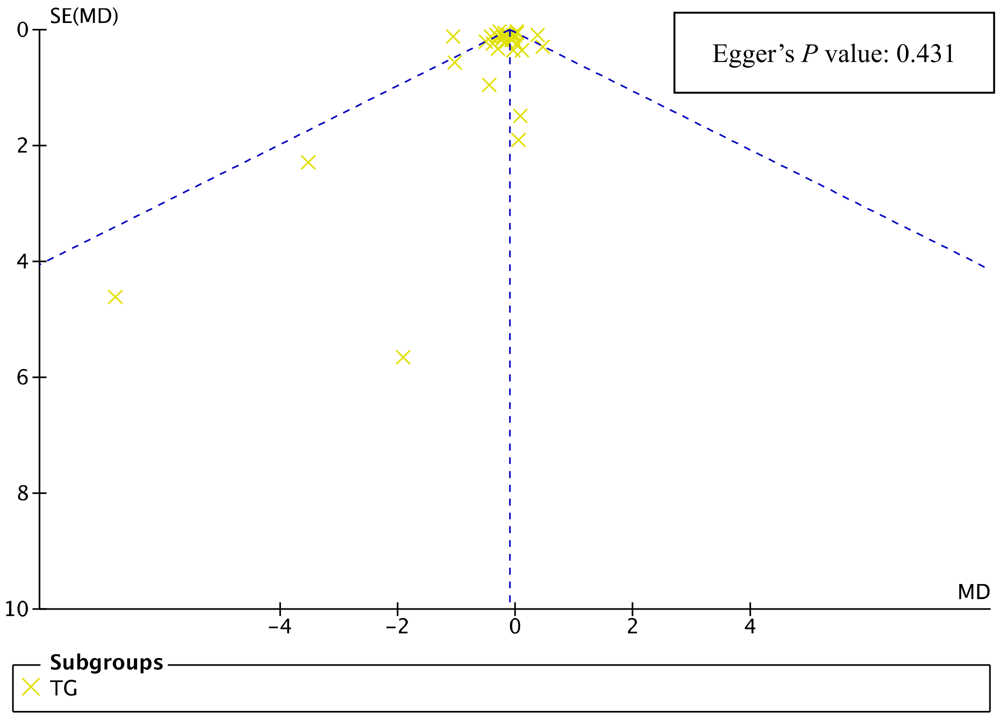

Figure S3-7-5 Funnel plot of RCTs investigating the effect of antioxidant fatty acids supplementation on TG in healthy population.

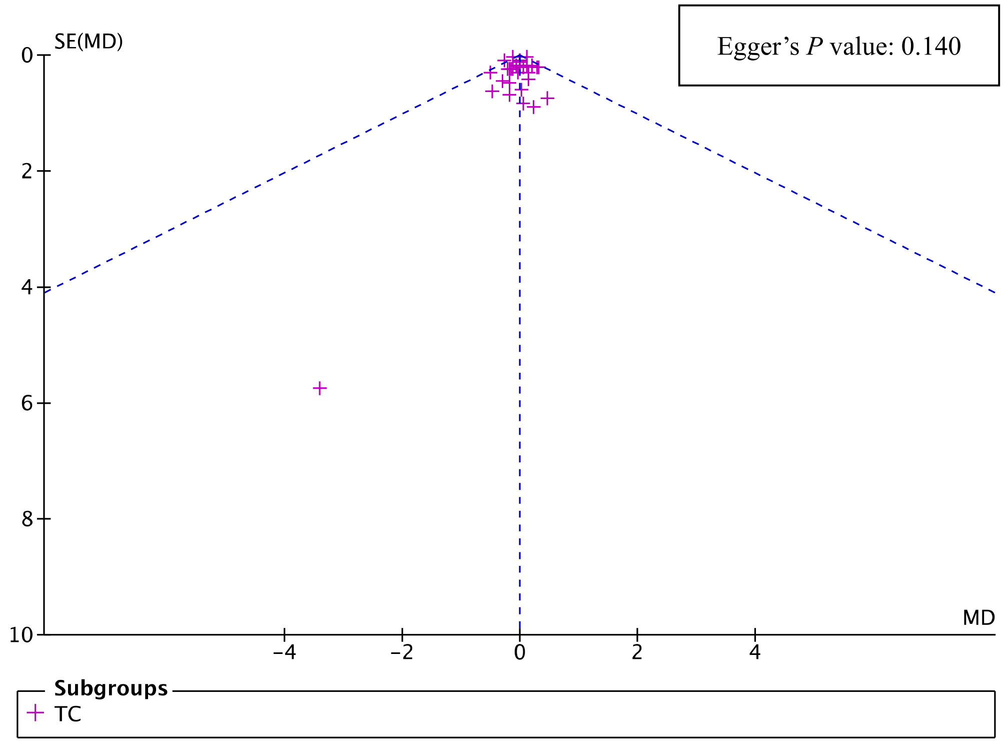

Figure S3-7-6 Funnel plot of RCTs investigating the effect of antioxidant fatty acids supplementation on TC in healthy population.

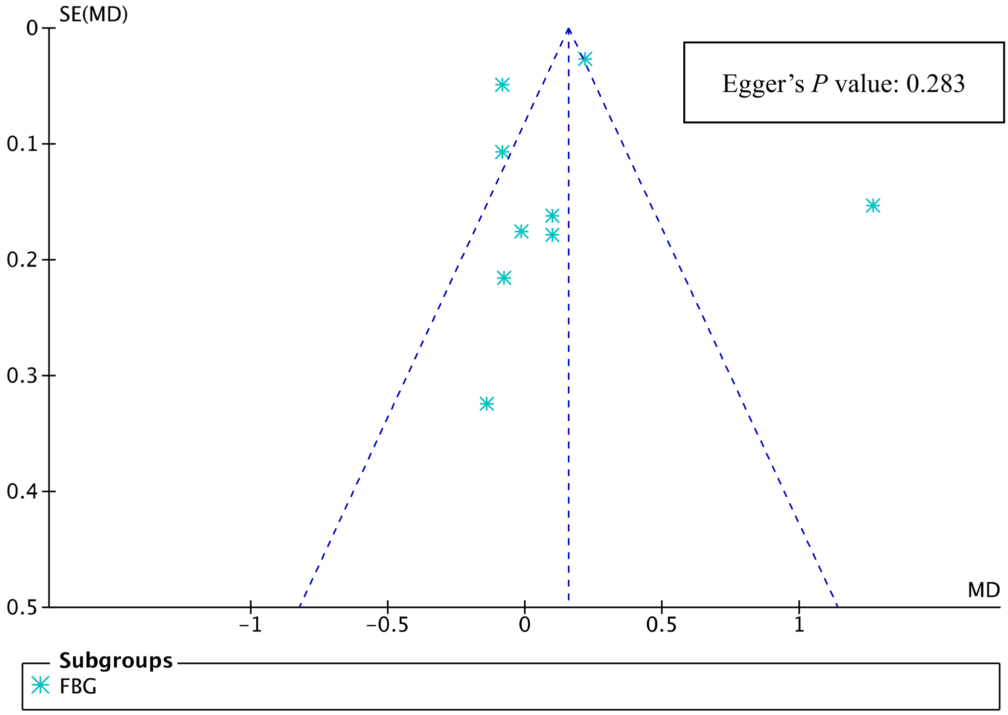

Figure S3-7-7 Funnel plot of RCTs investigating the effect of antioxidant fatty acids supplementation on FBG in healthy population.

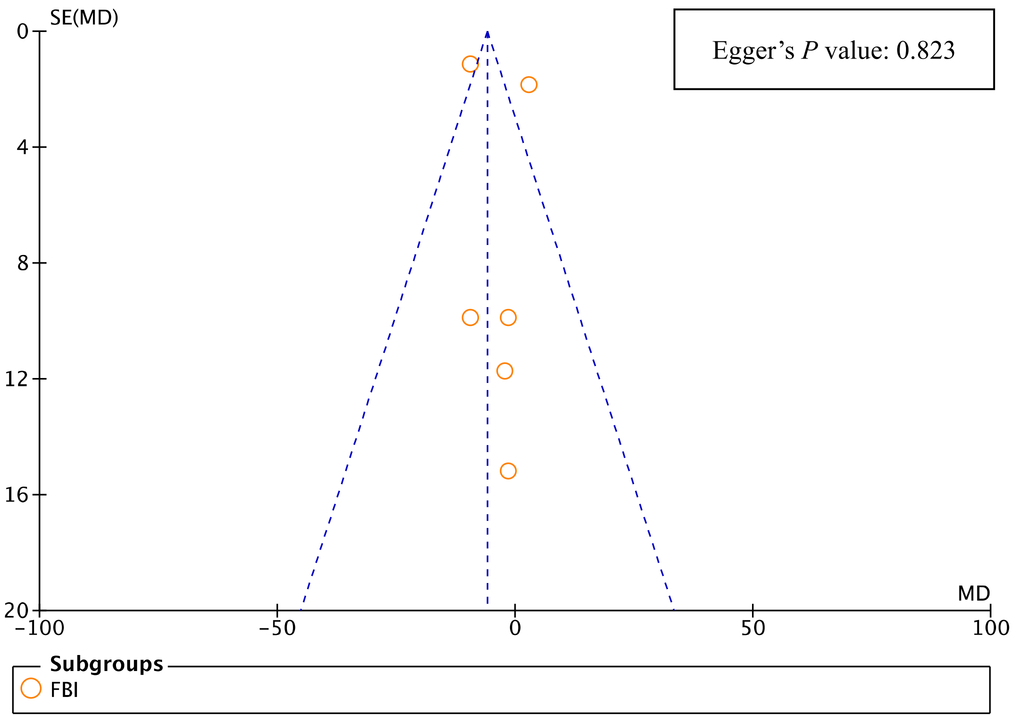

Figure S3-7-8 Funnel plot of RCTs investigating the effect of antioxidant fatty acids supplementation on FBI in healthy population.

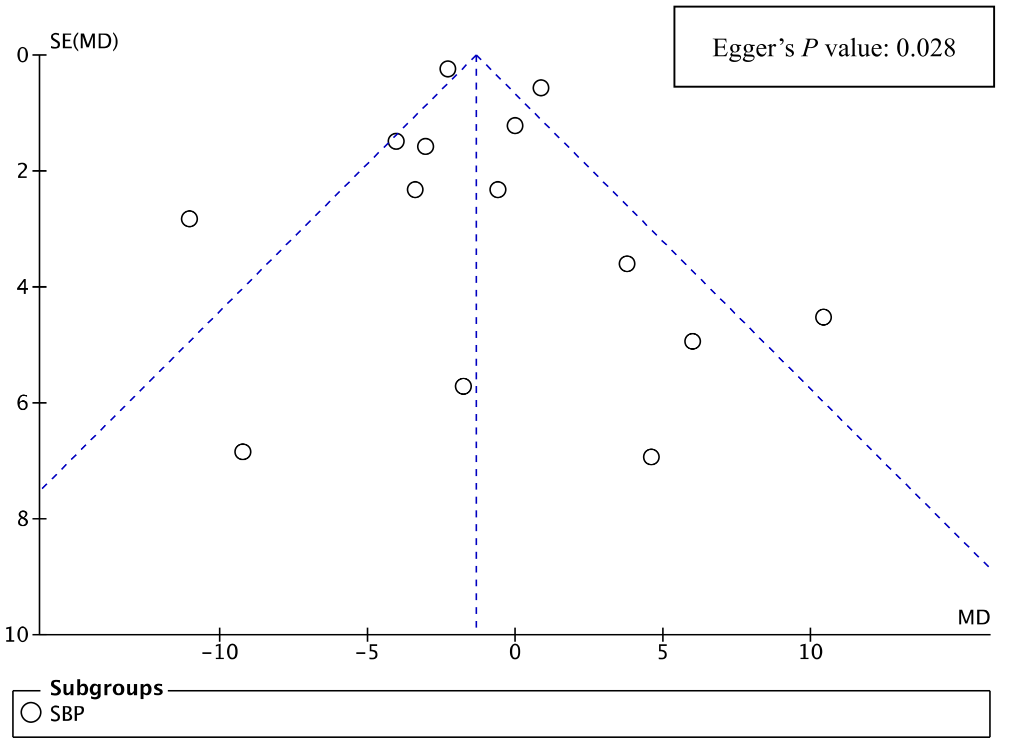

Figure S3-8-1 Funnel plot of RCTs investigating the effect of antioxidant fatty acids supplementation on SBP in population with diabetes.

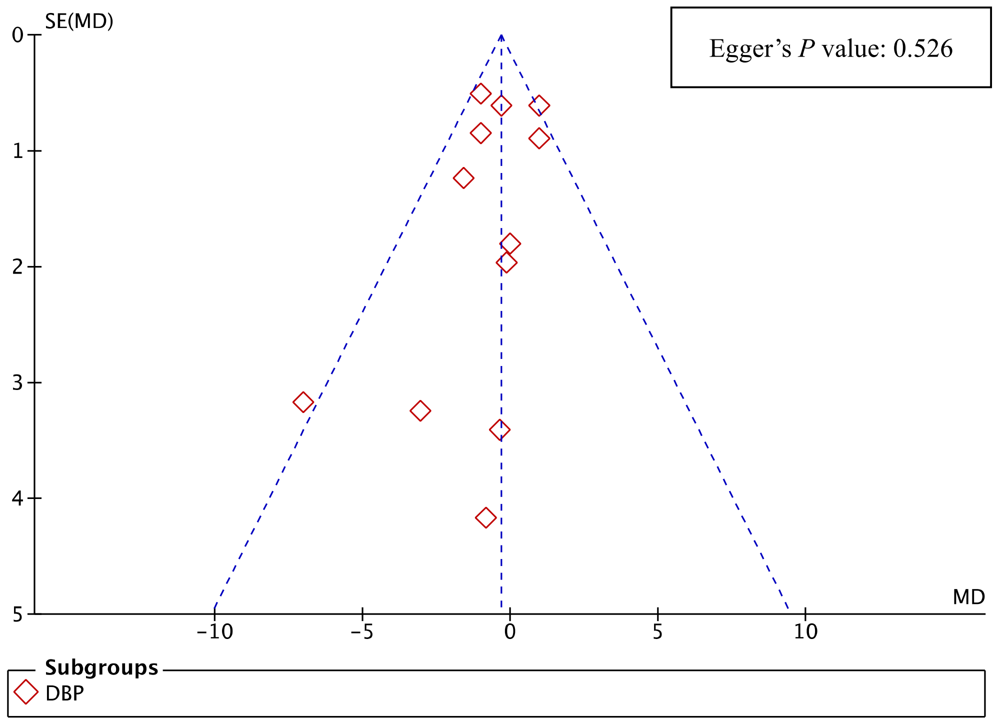

Figure S3-8-2 Funnel plot of RCTs investigating the effect of antioxidant fatty acids supplementation on DBP in population with diabetes.

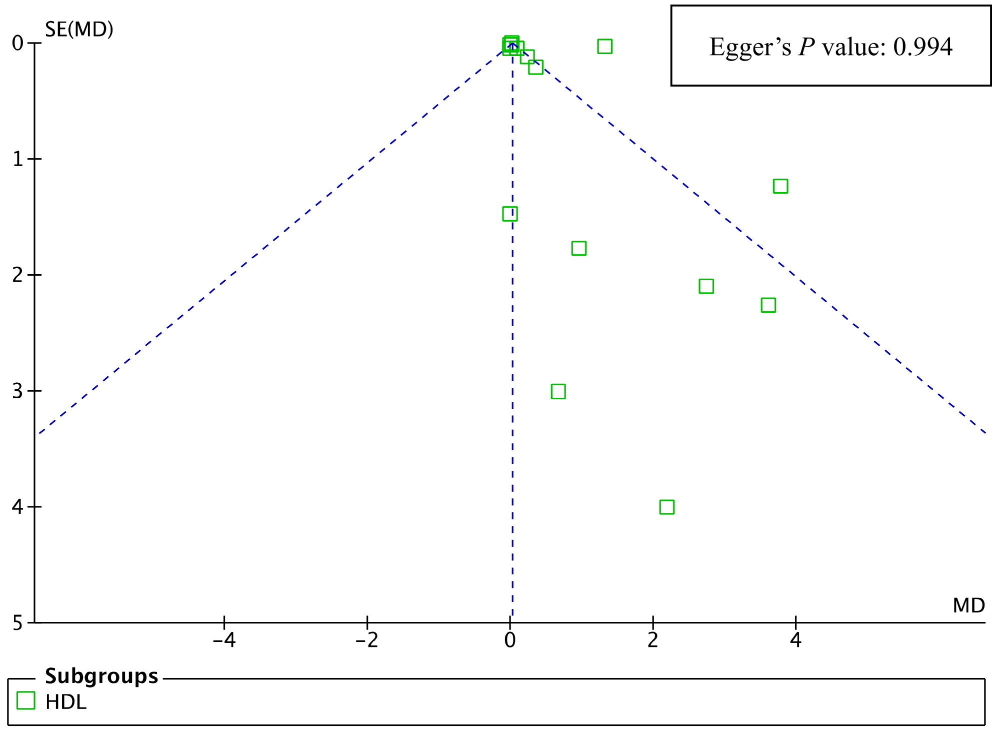

Figure S3-8-3 Funnel plot of RCTs investigating the effect of antioxidant fatty acids supplementation on HDL in population with diabetes.

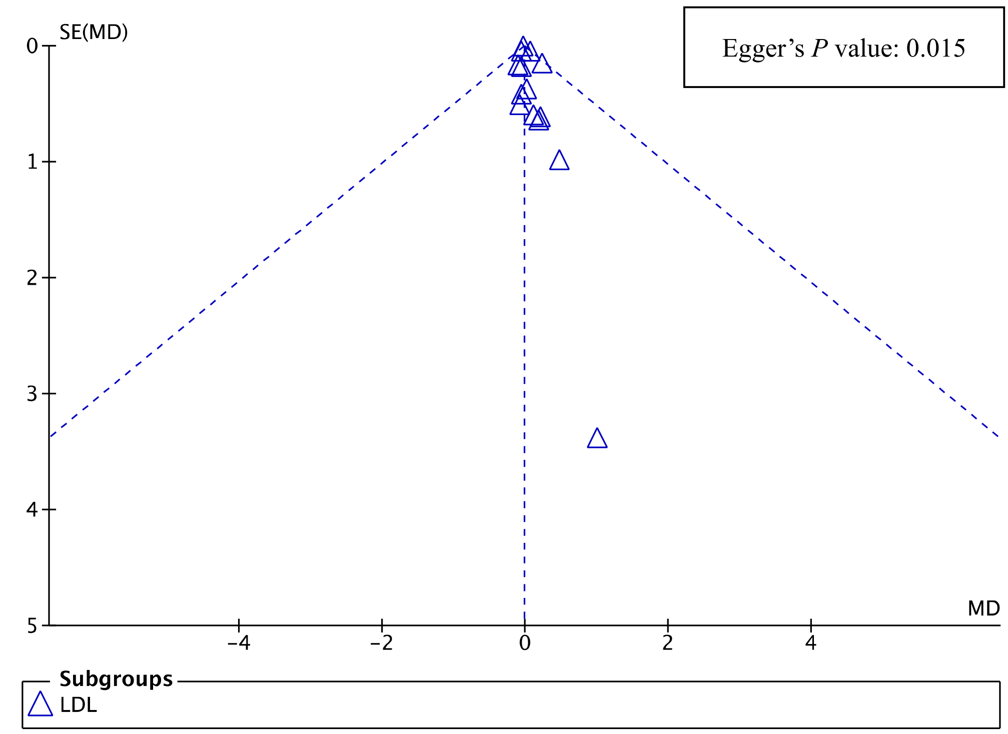

Figure S3-8-4 Funnel plot of RCTs investigating the effect of antioxidant fatty acids supplementation on LDL in population with diabetes.

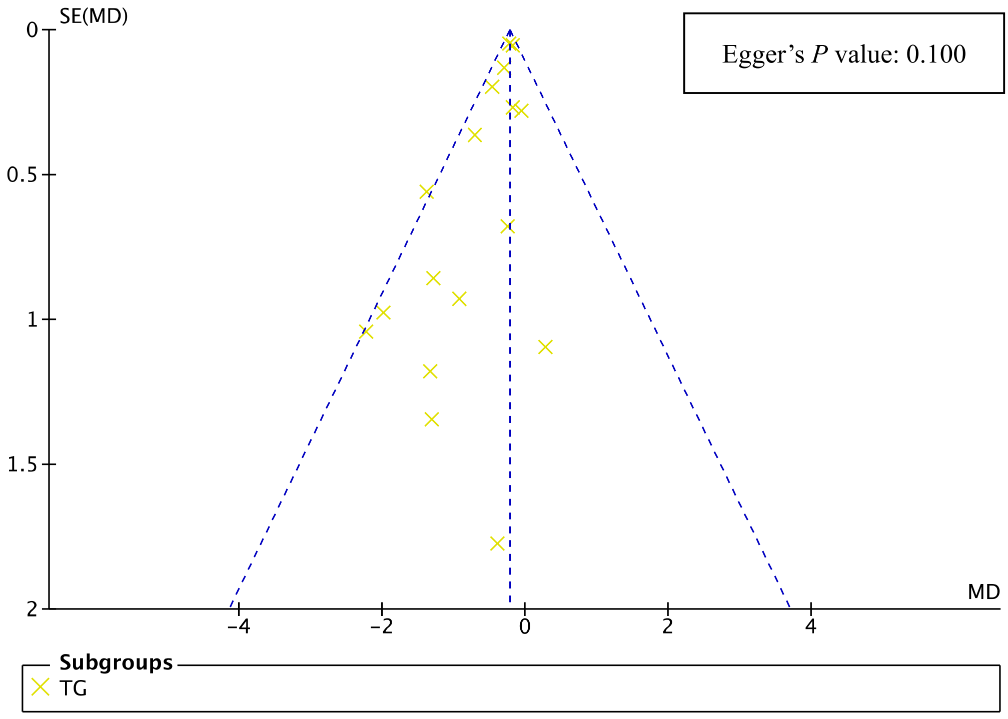

Figure S3-8-5 Funnel plot of RCTs investigating the effect of antioxidant fatty acids supplementation on TG in population with diabetes.

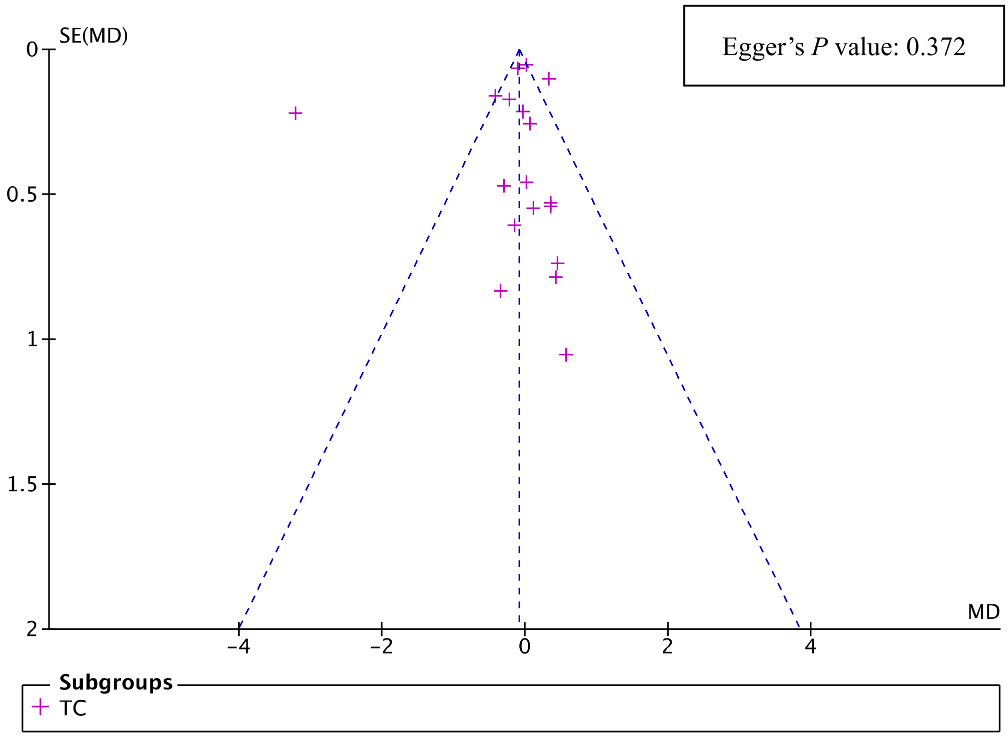

Figure S3-8-6 Funnel plot of RCTs investigating the effect of antioxidant fatty acids supplementation on TC in population with diabetes.

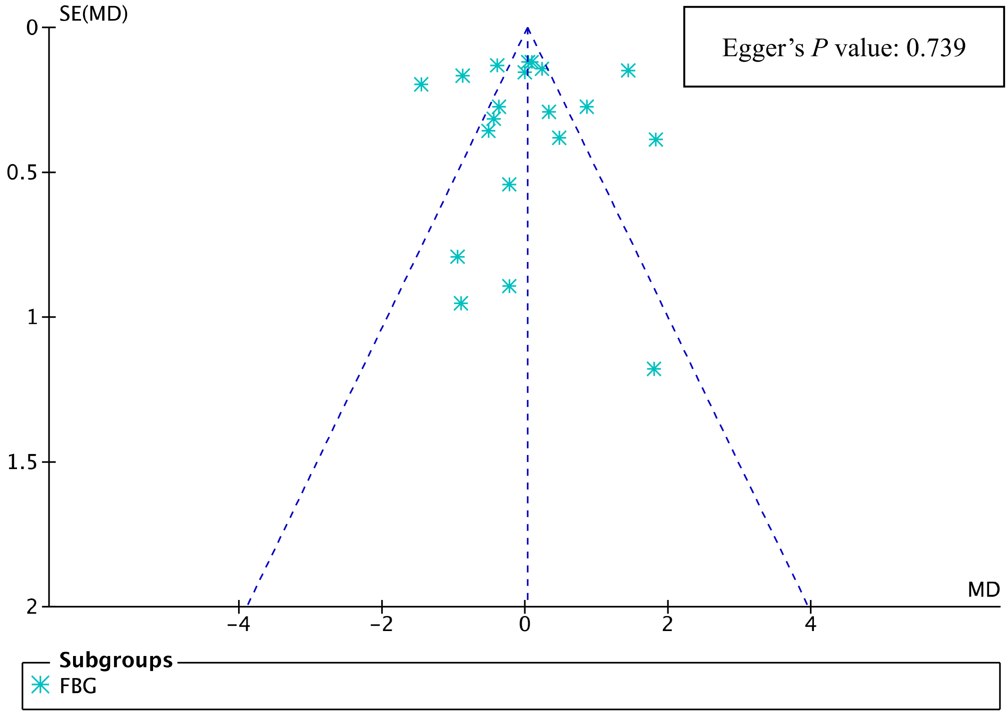

Figure S3-8-7 Funnel plot of RCTs investigating the effect of antioxidant fatty acids supplementation on FBG in population with diabetes.

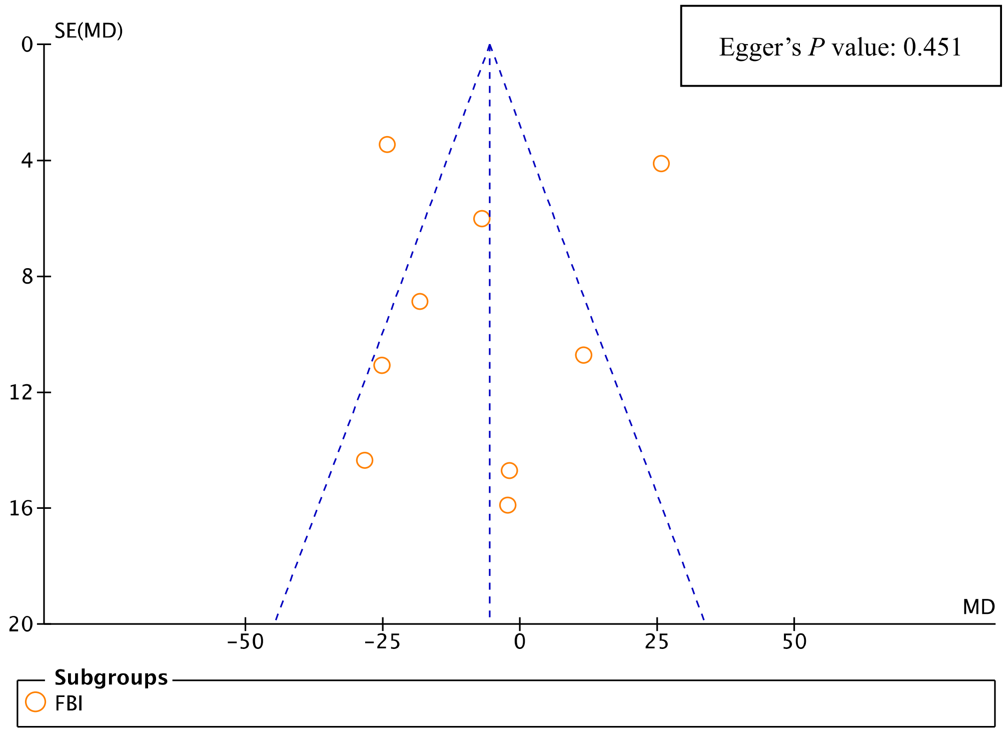

Figure S3-8-8 Funnel plot of RCTs investigating the effect of antioxidant fatty acids supplementation on FBI in population with diabetes.

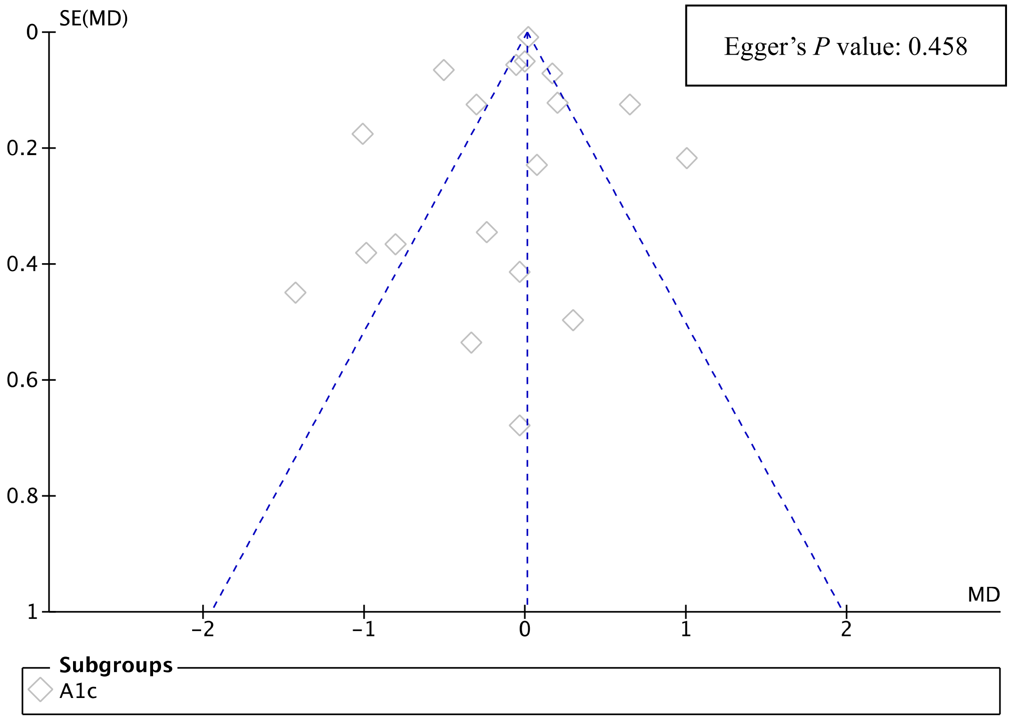

Figure S3-8-9 Funnel plot of RCTs investigating the effect of antioxidant fatty acids supplementation on A1C in population with diabetes.

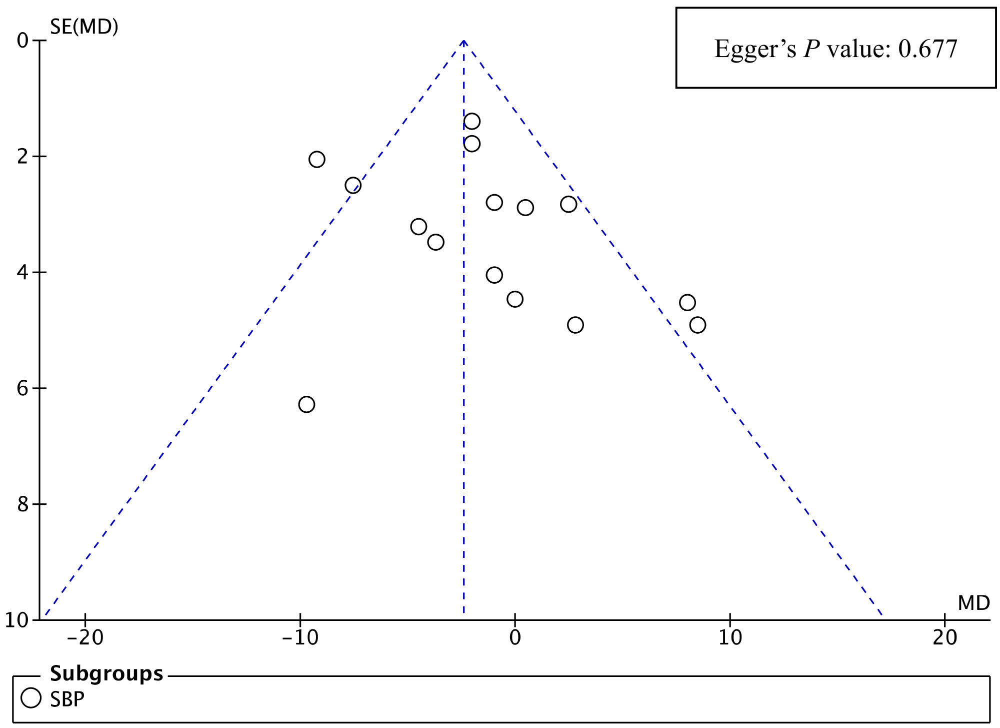

Figure S3-9-1 Funnel plot of RCTs investigating the effect of antioxidant fatty acids supplementation on SBP in population with hypertension.

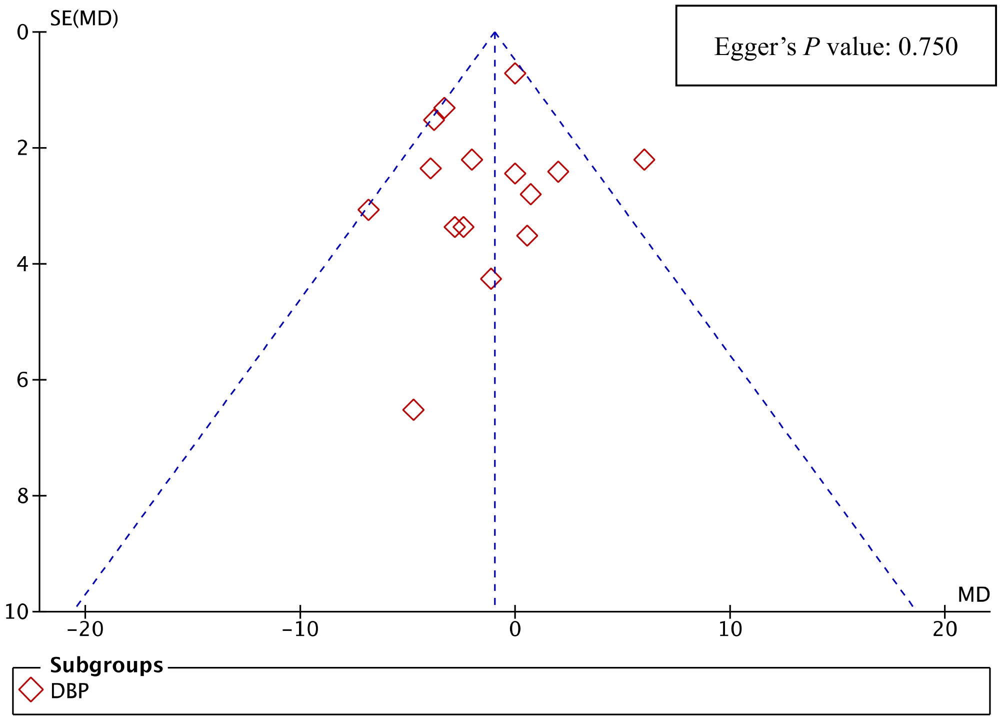

Figure S3-9-2 Funnel plot of RCTs investigating the effect of antioxidant fatty acids supplementation on DBP in population with hypertension.

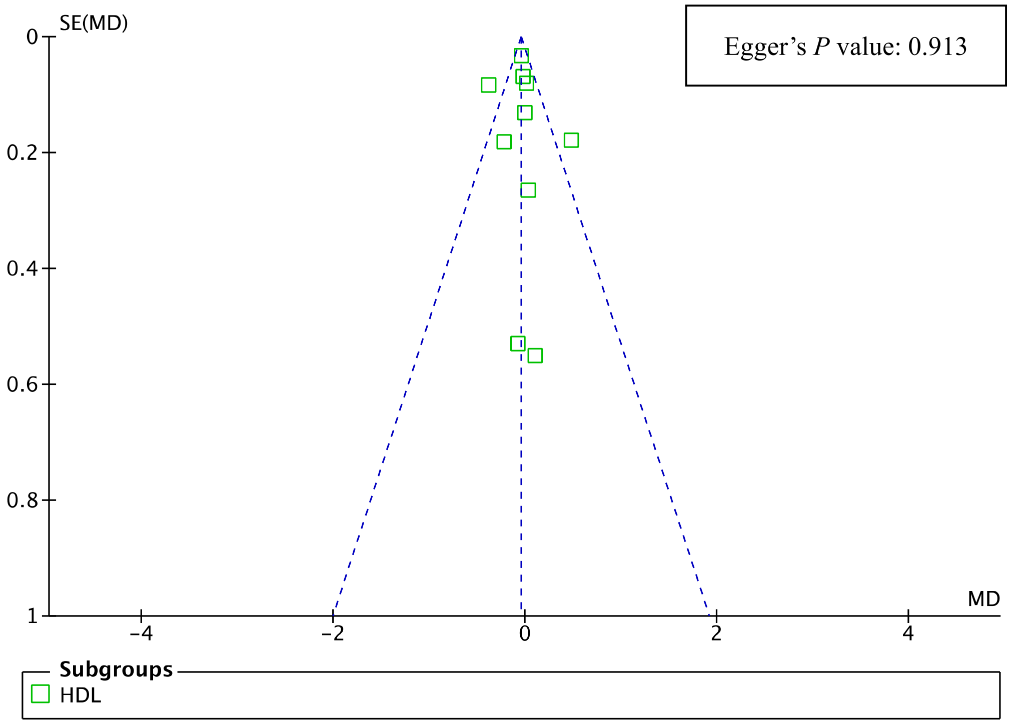

Figure S3-9-3 Funnel plot of RCTs investigating the effect of antioxidant fatty acids supplementation on HDL in population with hypertension.

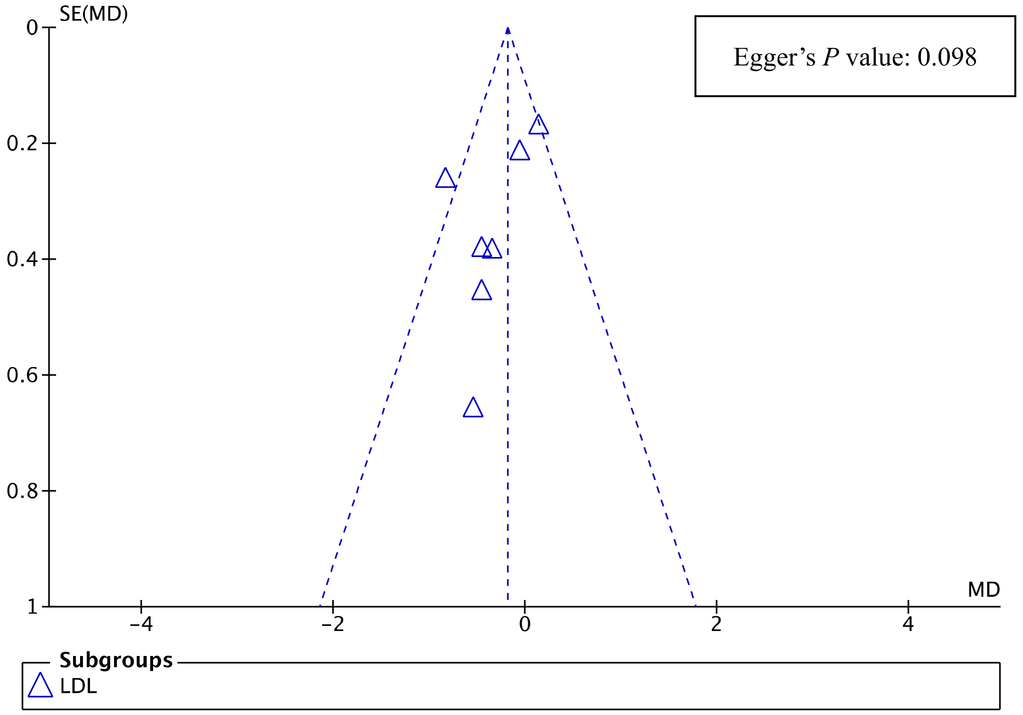

Figure S3-9-4 Funnel plot of RCTs investigating the effect of antioxidant fatty acids supplementation on LDL in population with hypertension.

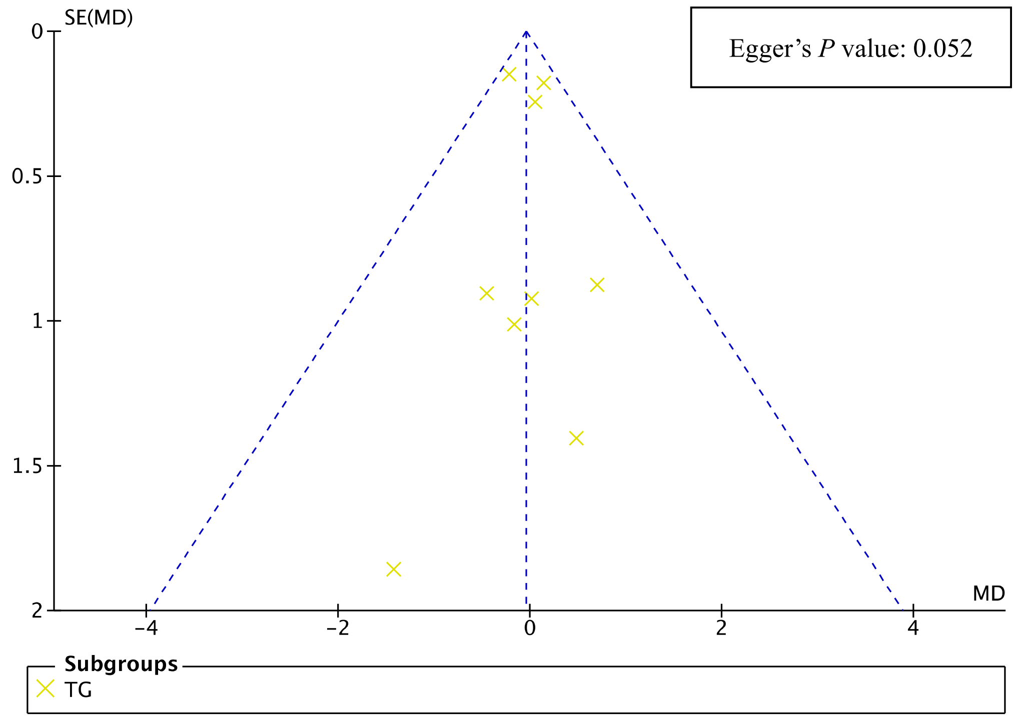

Figure S3-9-5 Funnel plot of RCTs investigating the effect of antioxidant fatty acids supplementation on TG in population with hypertension.

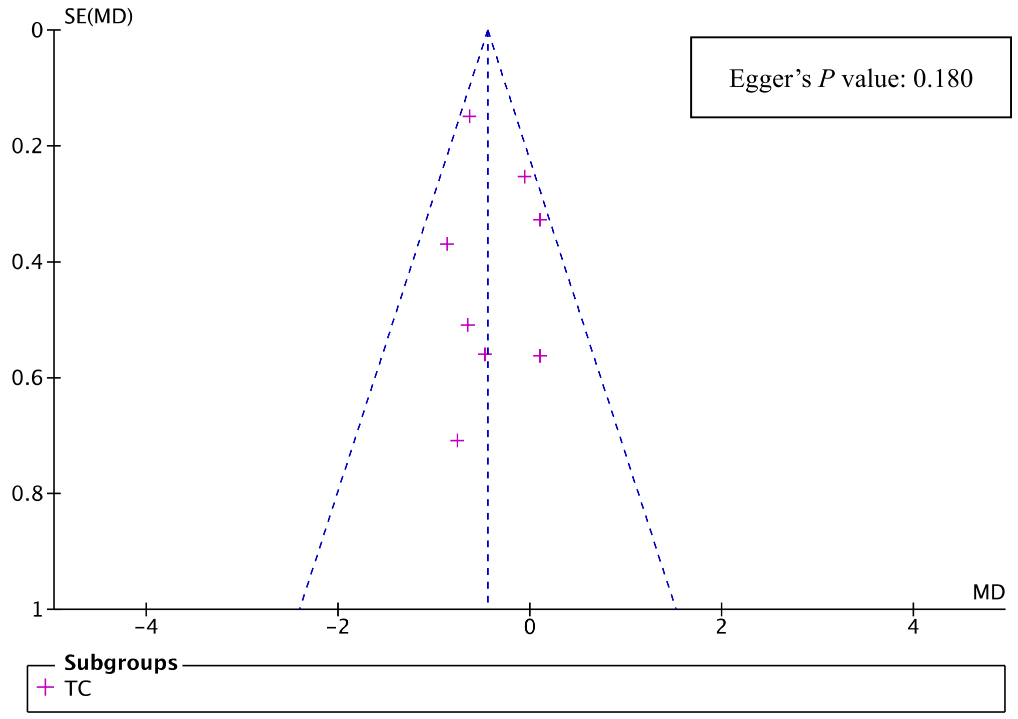

Figure S3-9-6 Funnel plot of RCTs investigating the effect of antioxidant fatty acids supplementation on TC in population with hypertension.

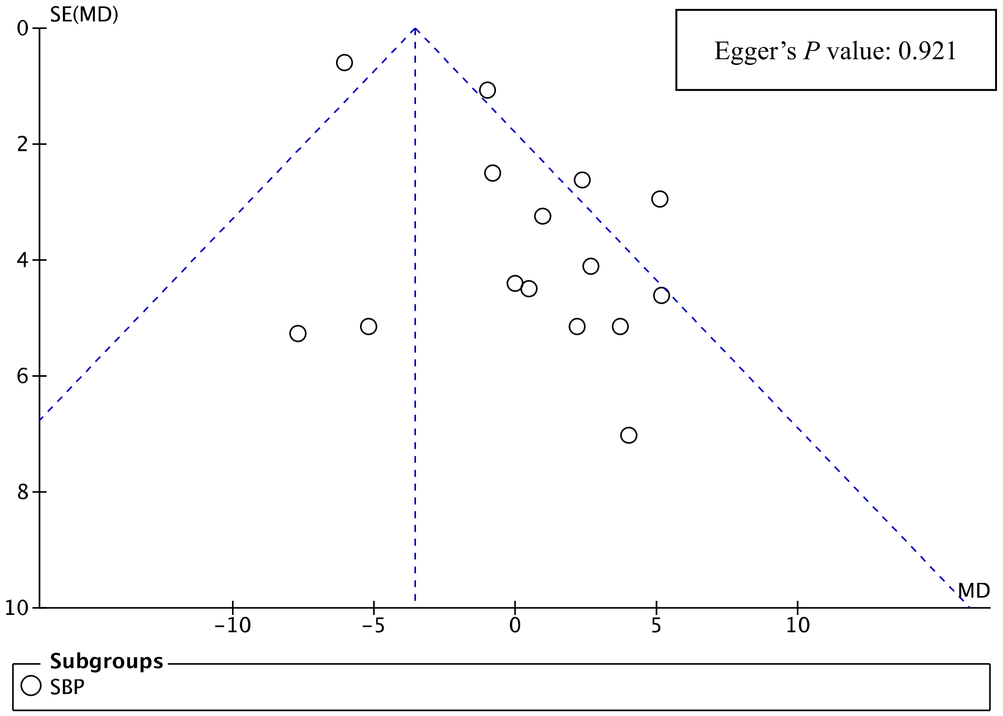

Figure S3-10-1 Funnel plot of RCTs investigating the effect of antioxidant fatty acids supplementation on SBP in population with dyslipidemia.

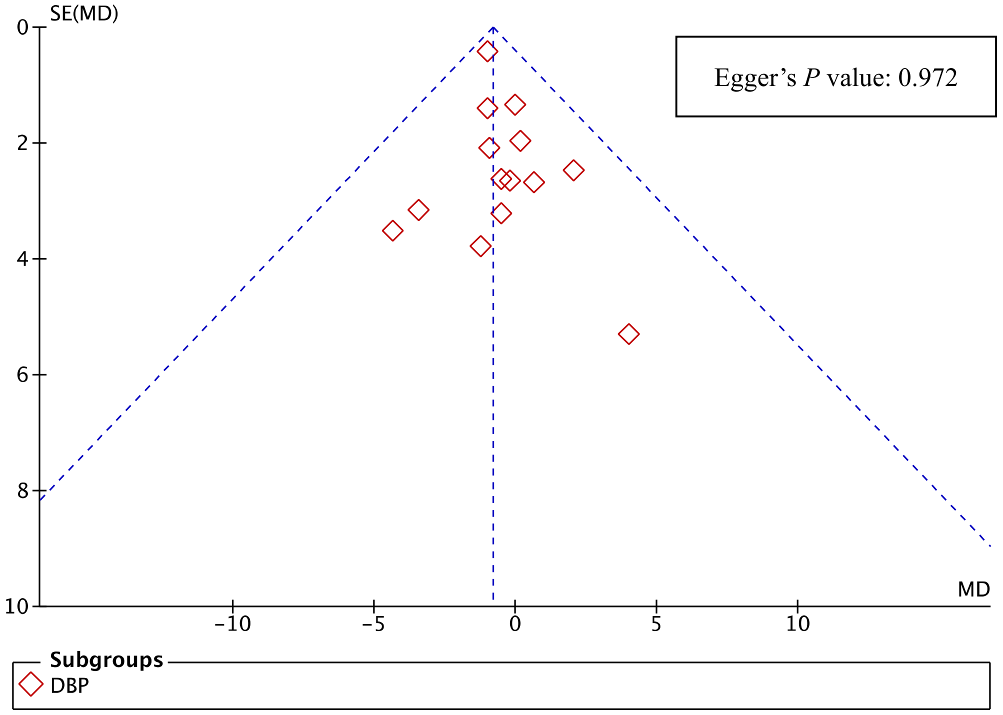

Figure S3-10-2 Funnel plot of RCTs investigating the effect of antioxidant fatty acids supplementation on DBP in population with dyslipidemia.

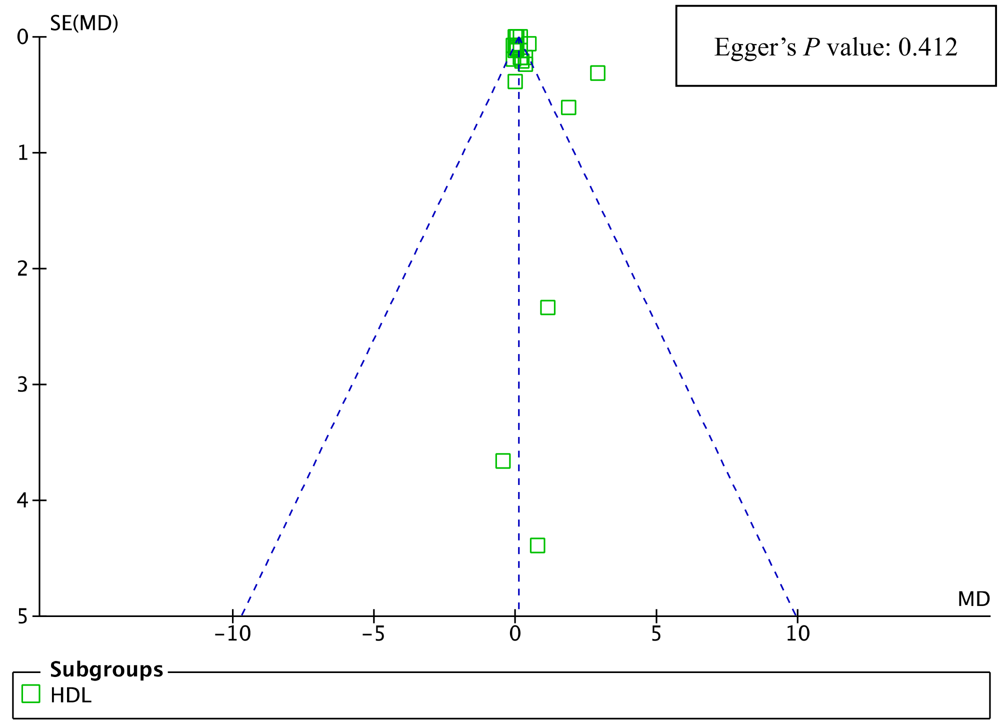

Figure S3-10-3 Funnel plot of RCTs investigating the effect of antioxidant fatty acids supplementation on HDL in population with dyslipidemia.

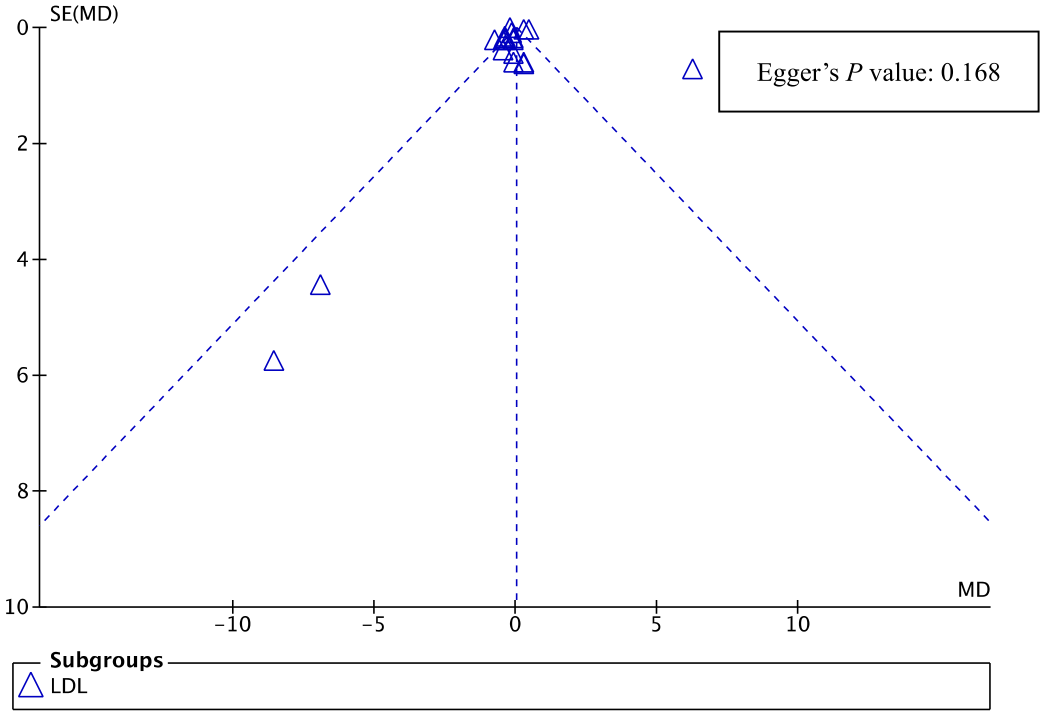

Figure S3-10-4 Funnel plot of RCTs investigating the effect of antioxidant fatty acids supplementation on LDL in population with dyslipidemia.

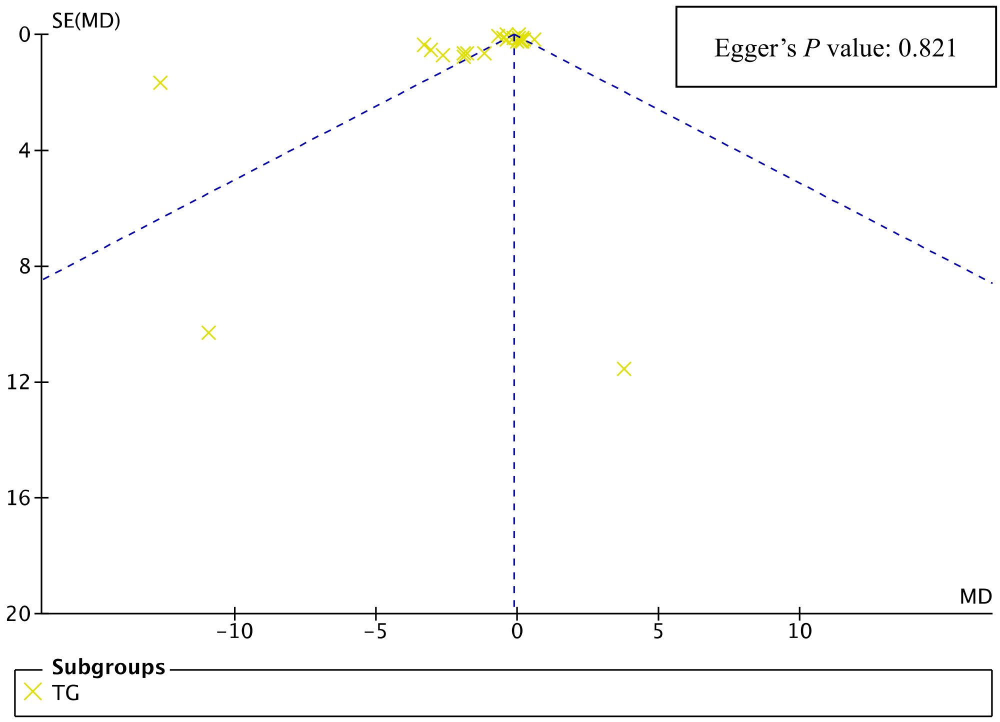

Figure S3-10-5 Funnel plot of RCTs investigating the effect of antioxidant fatty acids supplementation on TG in population with dyslipidemia.

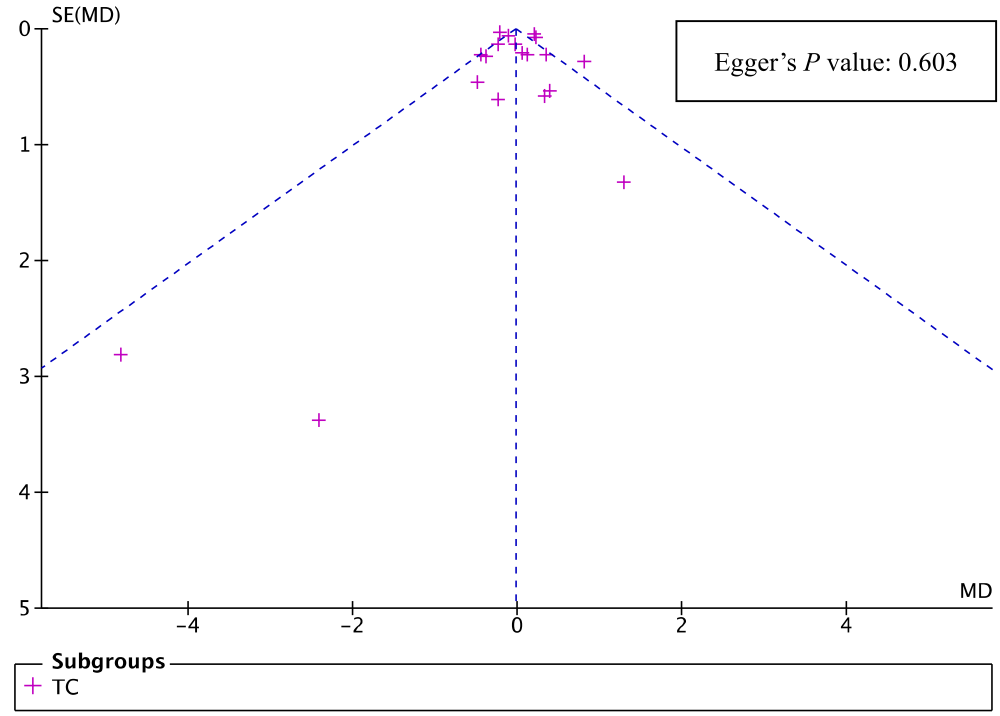

Figure S3-10-6 Funnel plot of RCTs investigating the effect of antioxidant fatty acids supplementation on TC in population with dyslipidemia.

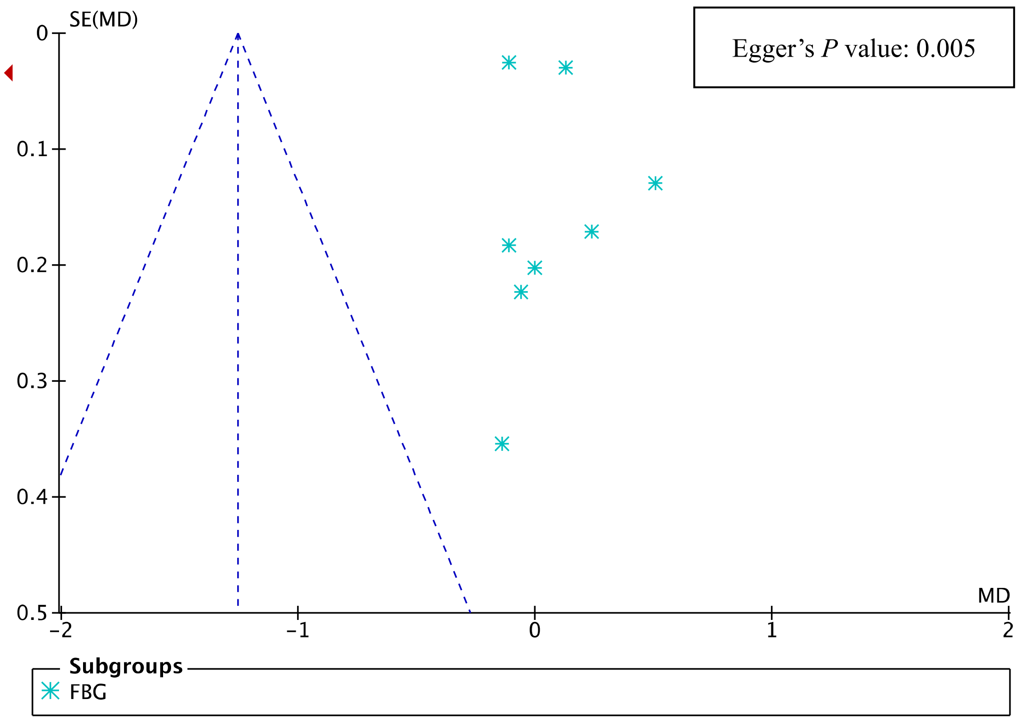

Figure S3-10-7 Funnel plot of RCTs investigating the effect of antioxidant fatty acids supplementation on FBG in population with dyslipidemia.

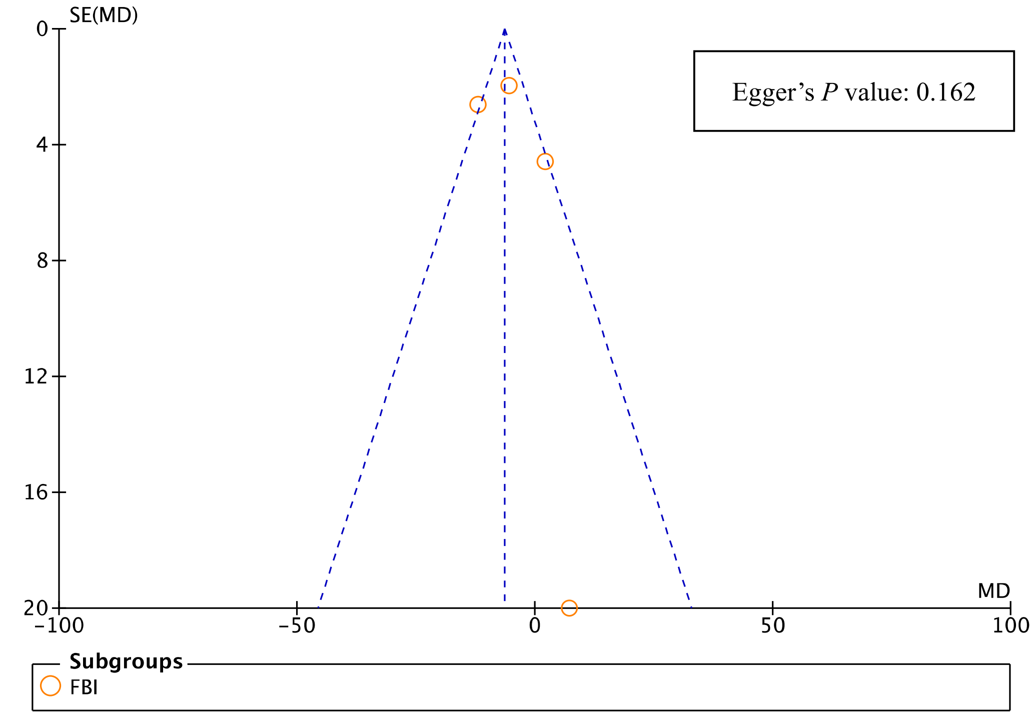

Figure S3-10-8 Funnel plot of RCTs investigating the effect of antioxidant fatty acids supplementation on FBI in population with dyslipidemia.

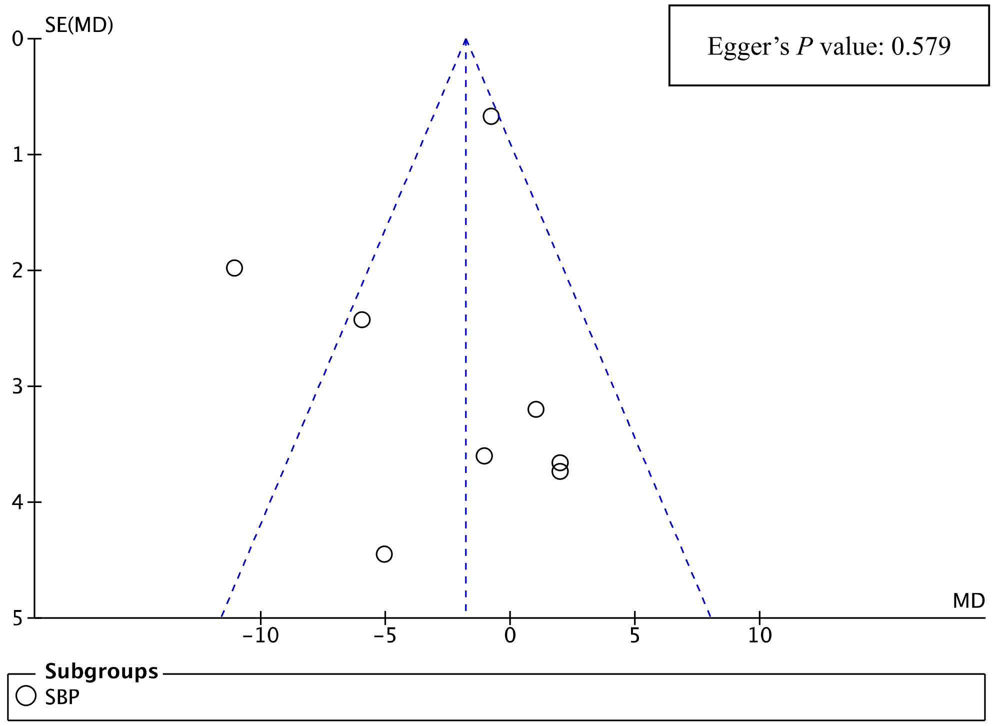

Figure S3-11-1 Funnel plot of RCTs investigating the effect of antioxidant fatty acids supplementation on SBP in population with obesity.

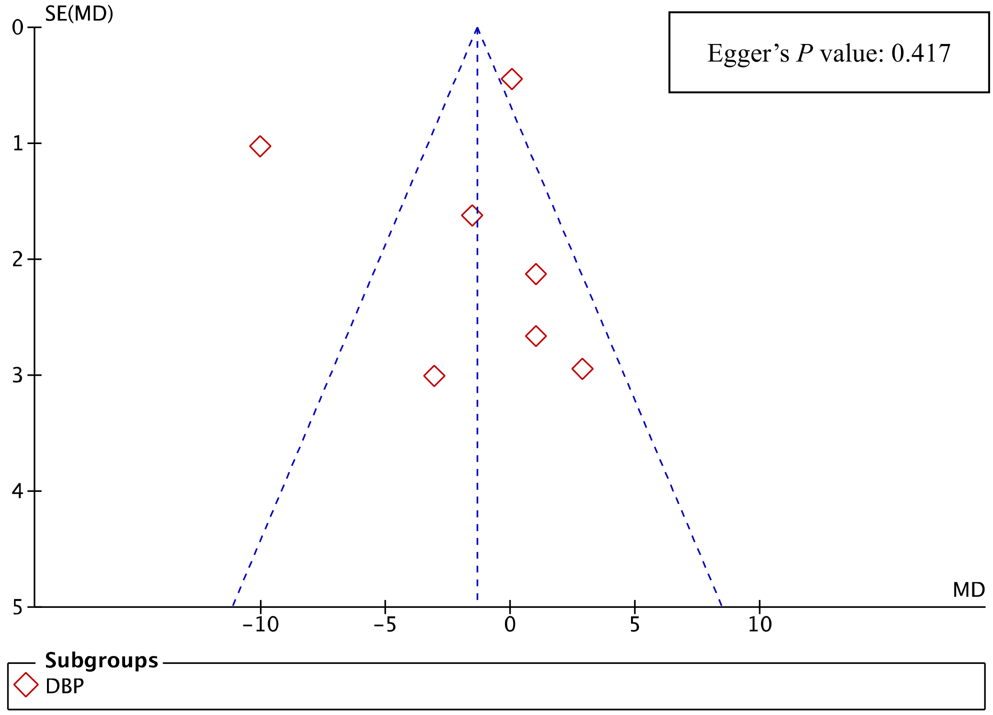

Figure S3-11-2 Funnel plot of RCTs investigating the effect of antioxidant fatty acids supplementation on DBP in population with obesity.

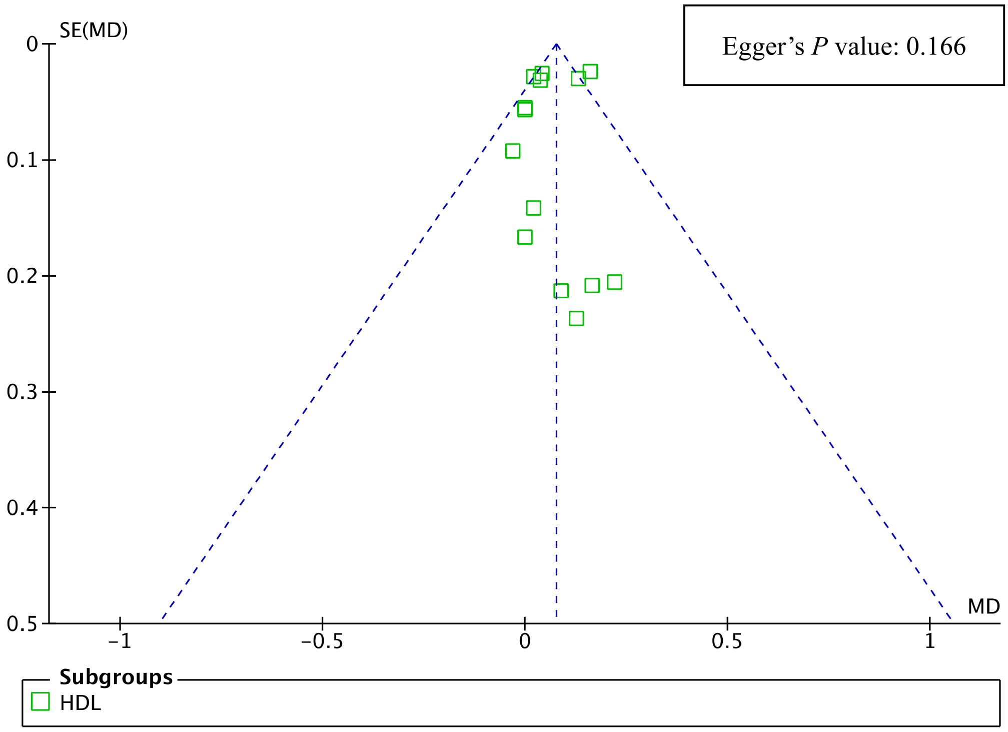

Figure S3-11-3 Funnel plot of RCTs investigating the effect of antioxidant fatty acids supplementation on HDL in population with obesity.

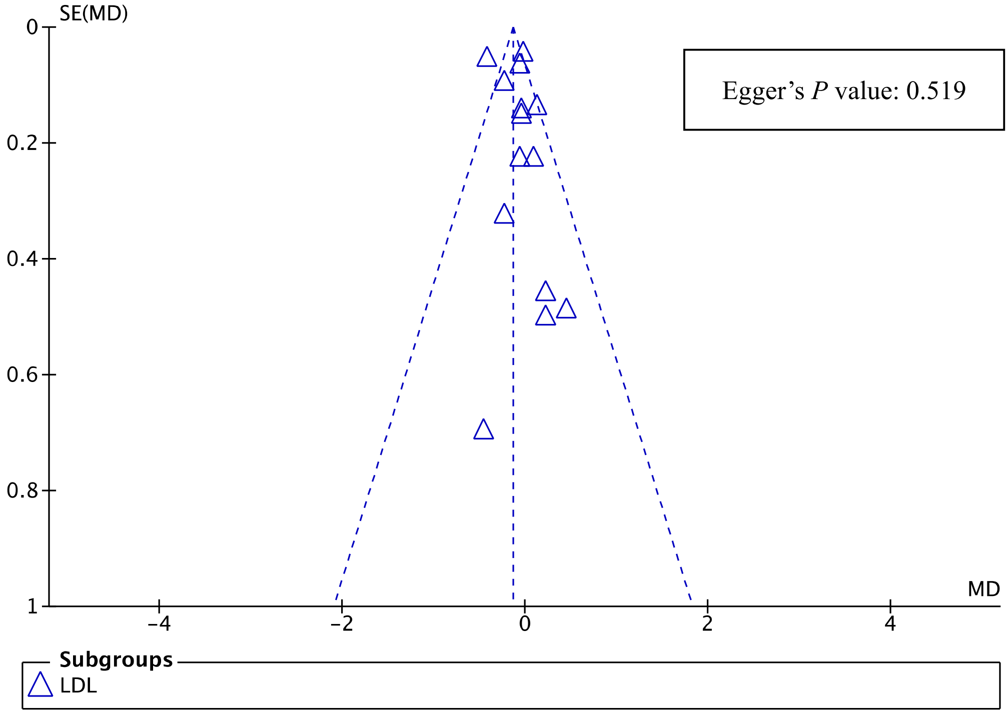

Figure S3-11-4 Funnel plot of RCTs investigating the effect of antioxidant fatty acids supplementation on LDL in population with obesity.

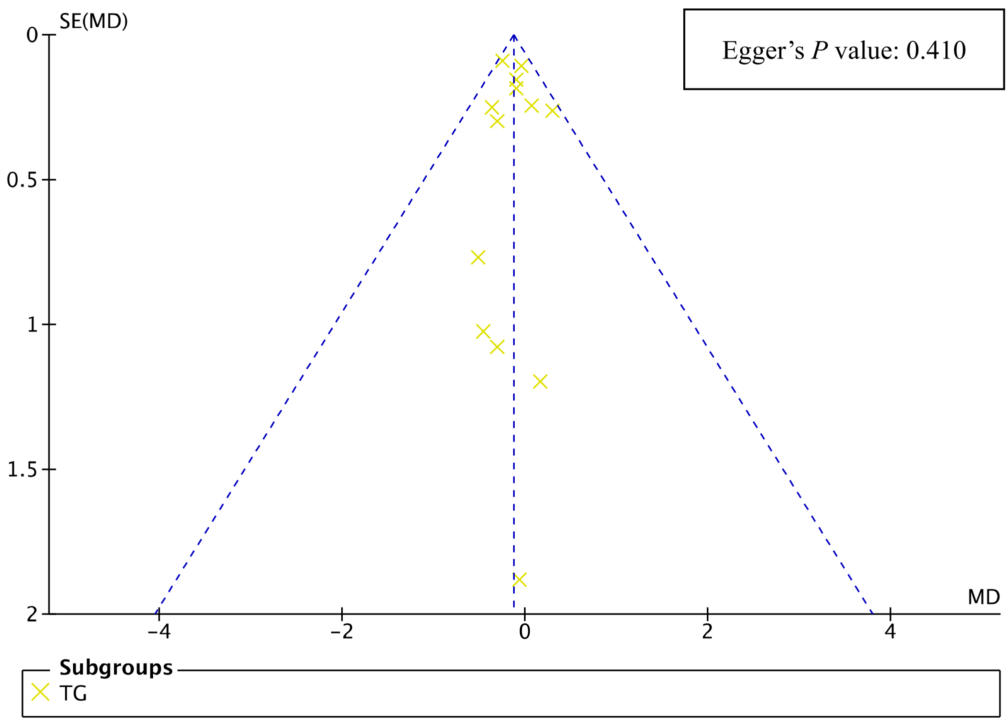

Figure S3-11-5 Funnel plot of RCTs investigating the effect of antioxidant fatty acids supplementation on TG in population with obesity.

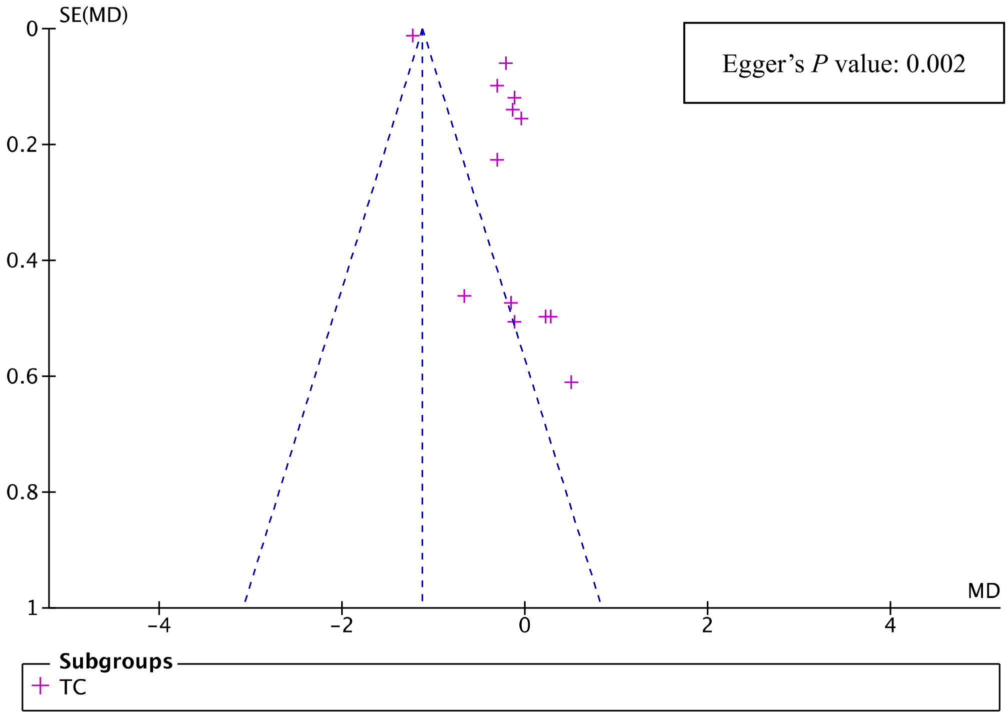

Figure S3-11-6 Funnel plot of RCTs investigating the effect of antioxidant fatty acids supplementation on TC in population with obesity.

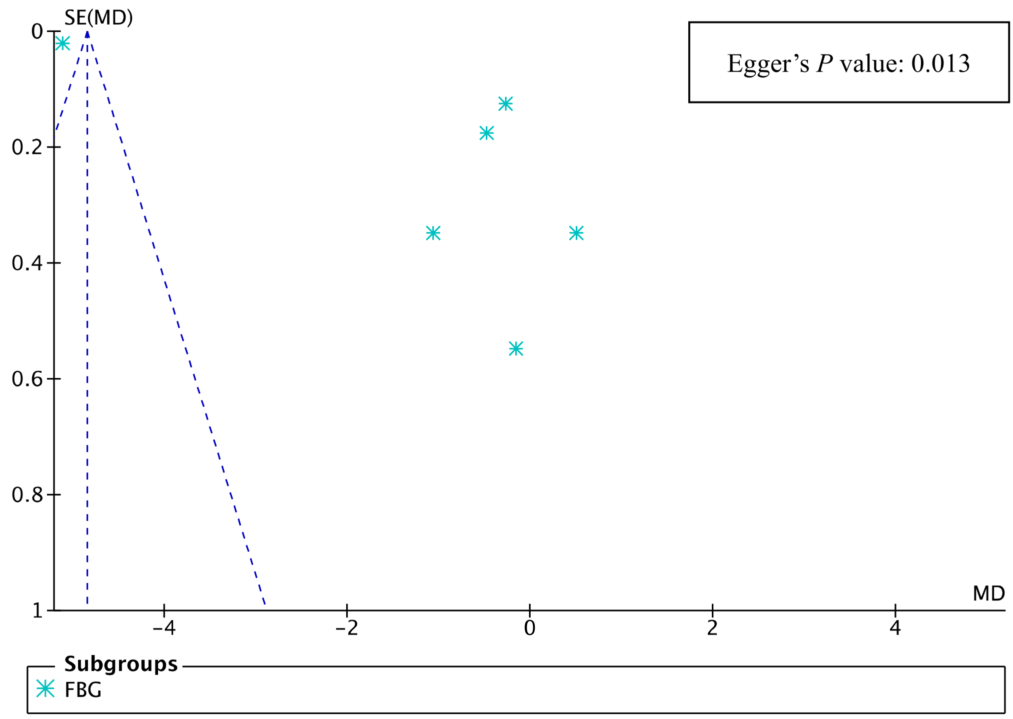

Figure S3-11-7 Funnel plot of RCTs investigating the effect of antioxidant fatty acids supplementation on FBG in population with obesity.

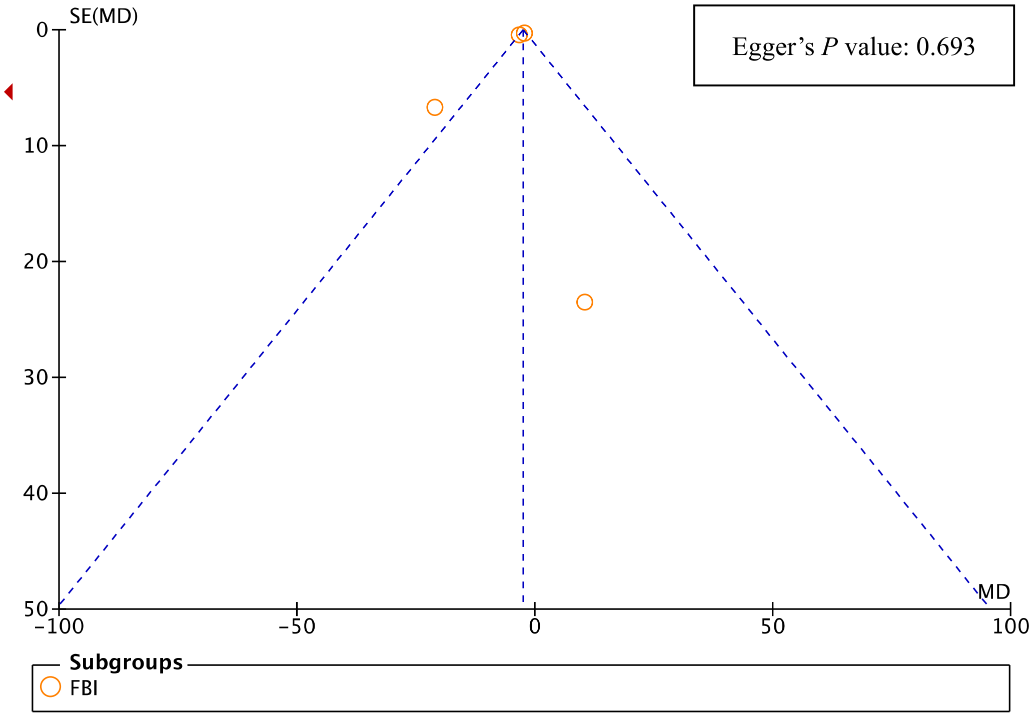

Figure S3-11-8 Funnel plot of RCTs investigating the effect of antioxidant fatty acids supplementation on FBI in population with obesity.

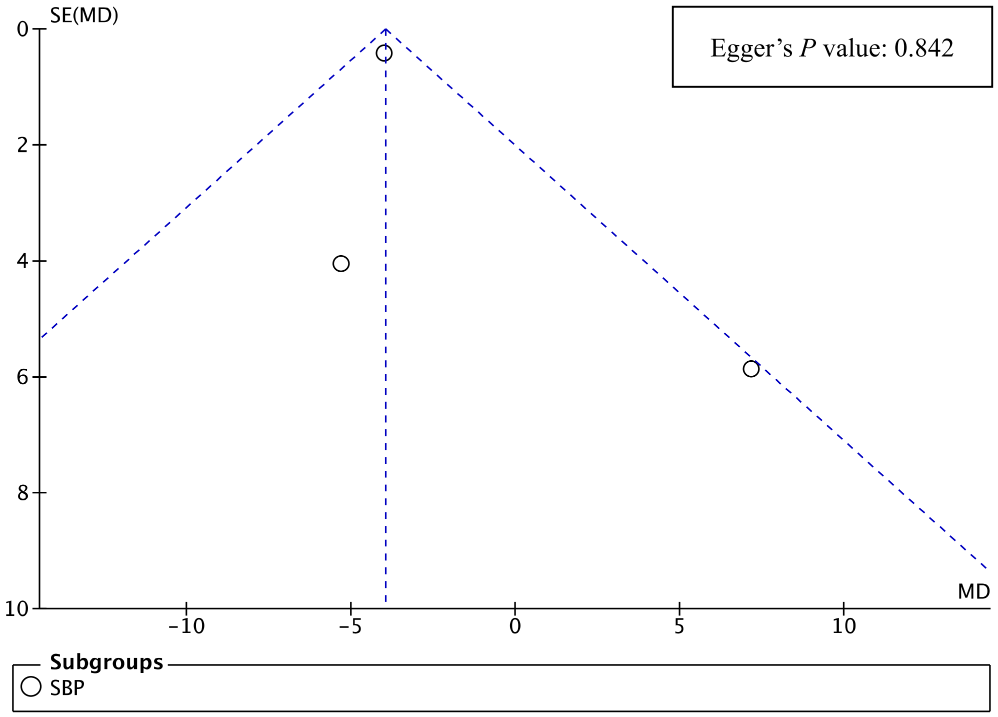

Figure S3-12-1 Funnel plot of RCTs investigating the effect of antioxidant fatty acids supplementation on SBP in population with metabolic syndrome.

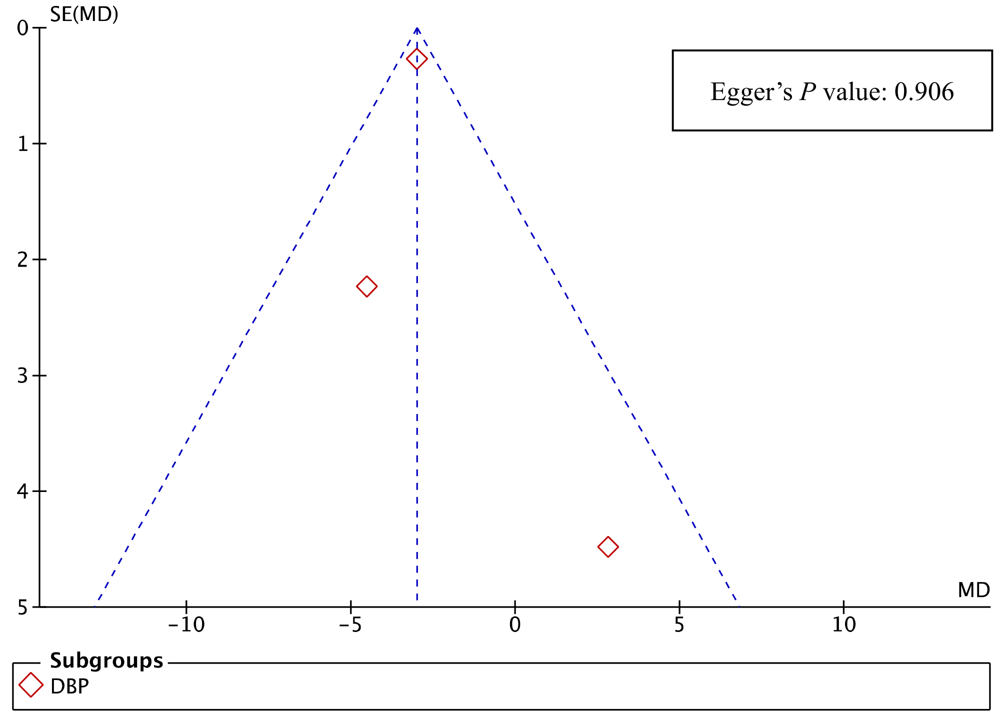

Figure S3-12-2 Funnel plot of RCTs investigating the effect of antioxidant fatty acids supplementation on DBP in population with metabolic syndrome.

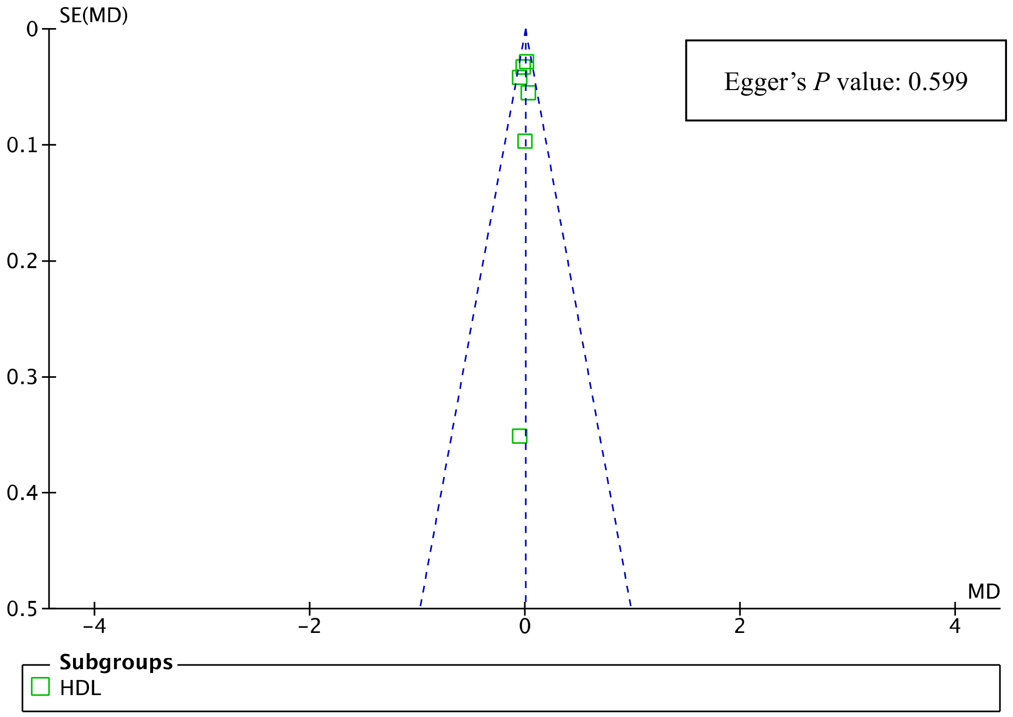

Figure S3-12-3 Funnel plot of RCTs investigating the effect of antioxidant fatty acids supplementation on HDL in population with metabolic syndrome.

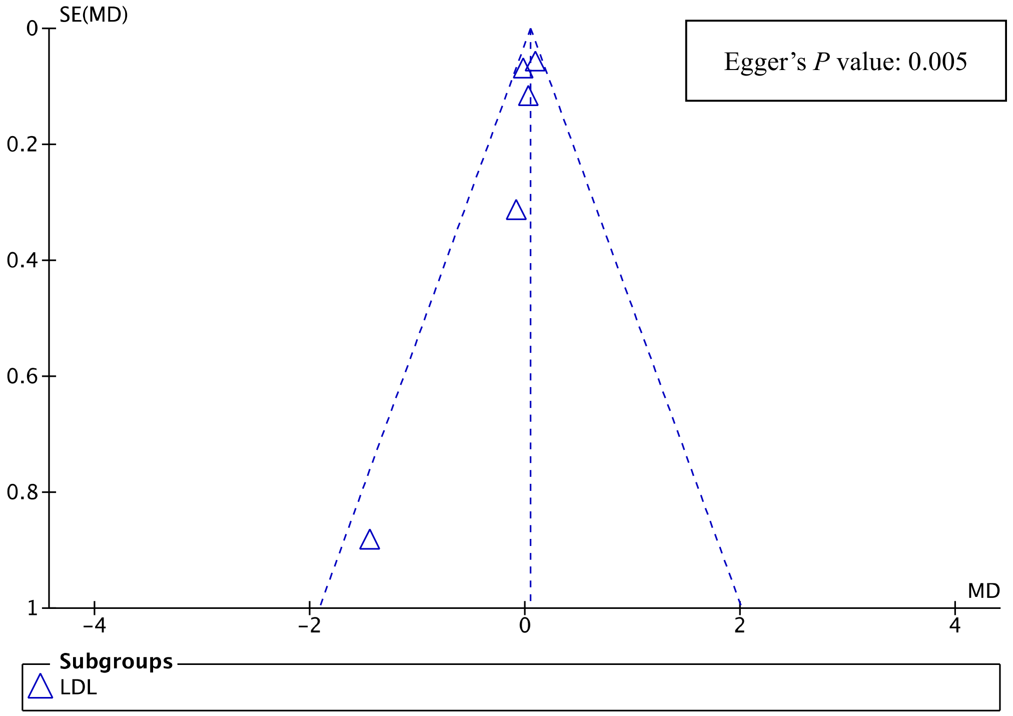

Figure S3-12-4 Funnel plot of RCTs investigating the effect of antioxidant fatty acids supplementation on LDL in population with metabolic syndrome.

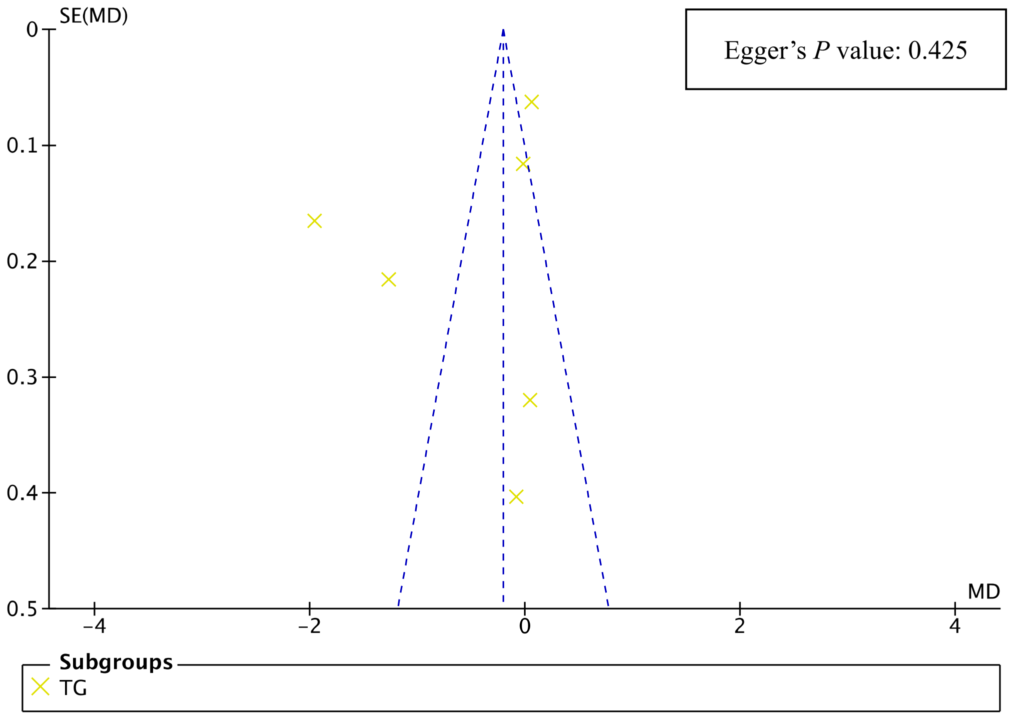

Figure S3-12-5 Funnel plot of RCTs investigating the effect of antioxidant fatty acids supplementation on TG in population with metabolic syndrome.

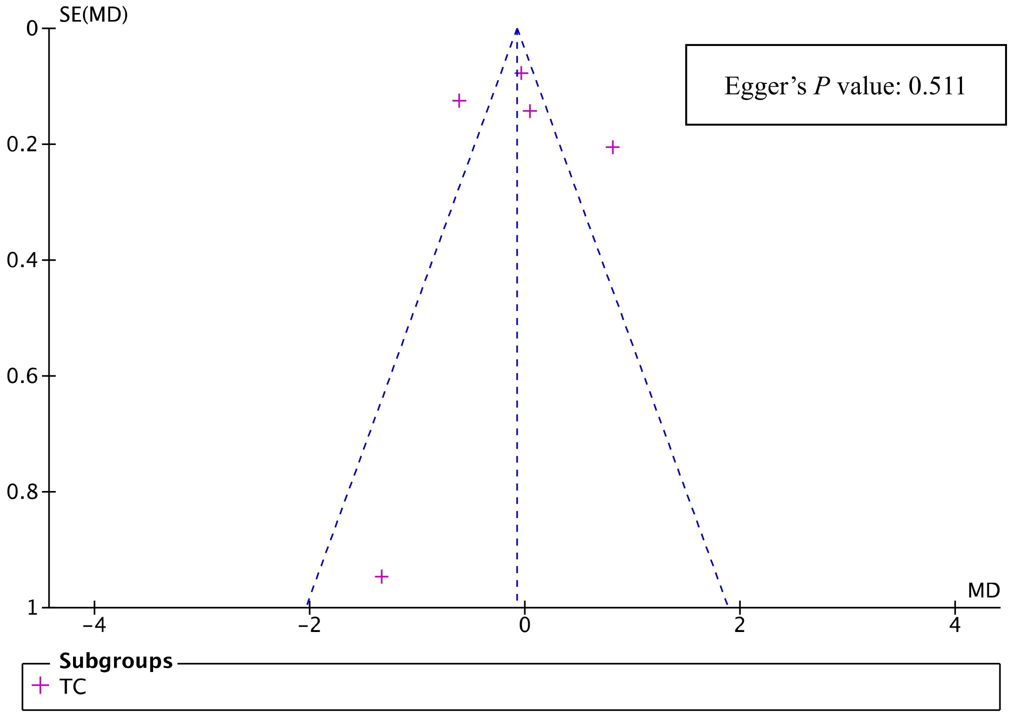

Figure S3-12-6 Funnel plot of RCTs investigating the effect of antioxidant fatty acids supplementation on TC in population with metabolic syndrome.

Supplemental 5 – GRADE

Table S2-1. GRADE profile for n3 fatty acids supplementation.

Certainty assessment							№ of patients		Effect		Certainty	Importance
№ of studies	Study design	Risk of bias	Inconsistency	Indirectness	Imprecision	Other considerations	[n3]	[comparison]	Relative (95% CI)	Absolute (95% CI)		
**Outcome - SBP**												
59	randomised trials	not serious	serious[a]	not serious	serious[b]	none	3086	3071	-	MD **1.03 lower** (2.84 lower to 0.78 higher)	⨁⨁◯◯ Low	
**Outcome - DBP**												
54	randomised trials	not serious	serious[a]	not serious	serious[b]	none	2820	2811	-	MD **0.32 lower** (1.15 lower to 0.52 higher)	⨁⨁◯◯ Low	
**Outcome - HDL**												

Certainty assessment							№ of patients		Effect		Certainty	Importance
№ of studies	Study design	Risk of bias	Inconsistency	Indirectness	Imprecision	Other considerations	[n3]	[comparison]	Relative (95% CI)	Absolute (95% CI)		
65	randomised trials	not serious	serious[a]	not serious	serious[b]	none	2722	2462	-	MD **0.2 higher** (0.13 higher to 0.27 higher)	⊕⊕◯◯ Low	
**Outcome - LDL**												
59	randomised trials	not serious	serious[a]	not serious	serious[b]	none	2617	2303	-	MD **0.07 higher** (0.04 lower to 0.18 higher)	⊕⊕◯◯ Low	
**Outcome - TAG**												
64	randomised trials	not serious	serious[a]	not serious	not serious	none	2655	2406	-	MD **0.37 lower** (0.47 lower to 0.28 lower)	⊕⊕⊕◯ Moderate	
**Outcome - TC**												

Certainty assessment							№ of patients		Effect		Certainty	Importance
№ of studies	Study design	Risk of bias	Inconsistency	Indirectness	Imprecision	Other considerations	[n3]	[comparison]	Relative (95% CI)	Absolute (95% CI)		
59	randomised trials	not serious	serious[a]	not serious	serious[b]	none	1995	1941	-	MD **0.05 lower** (0.18 lower to 0.08 higher)	⊕⊕◯◯ Low	

Outcome - FBG

38	randomised trials	not serious	serious[a]	not serious	serious[b]	none	1125	1171	-	MD **0.09 higher** (1.24 lower to 1.42 higher)	⊕⊕◯◯ Low	

Outcome - Insulin

22	randomised trials	not serious	serious[a]	not serious	serious[b]	none	803	812	-	MD **3.19 lower** (19.06 lower to 12.68 higher)	⊕⊕◯◯ Low	

Outcome - HbA1c

Certainty assessment							№ of patients		Effect		Certainty	Importance
№ of studies	Study design	Risk of bias	Inconsistency	Indirectness	Imprecision	Other considerations	[n3]	[comparison]	Relative (95% CI)	Absolute (95% CI)		
22	randomised trials	not serious	serious[a]	not serious	serious[b]	none	803	788	-	MD **0.06 lower** (0.17 lower to 0.06 higher)	⨁⨁◯◯ Low	

CI: confidence interval; **MD:** mean difference

Explanations

a. Rated down for inconsistency.

b. Rated down for imprecision.

Table S2-2. GRADE profile for n6 fatty acids supplementation.

Certainty assessment							№ of patients		Effect		Certainty	Importance
№ of studies	Study design	Risk of bias	Inconsistency	Indirectness	Imprecision	Other considerations	[n6]	[comparison]	Relative (95% CI)	Absolute (95% CI)		
**Outcome - SBP**												

Certainty assessment							№ of patients		Effect		Certainty	Importance
№ of studies	Study design	Risk of bias	Inconsistency	Indirectness	Imprecision	Other considerations	[n6]	[comparison]	Relative (95% CI)	Absolute (95% CI)		
15	randomised trials	not serious	serious[a]	not serious	serious[b]	none	593	596	-	MD **0.47 higher** (1.85 lower to 2.78 higher)	⊕⊕◯◯ Low	

Outcome - DBP

№ of studies	Study design	Risk of bias	Inconsistency	Indirectness	Imprecision	Other considerations	[n6]	[comparison]	Relative (95% CI)	Absolute (95% CI)	Certainty	Importance
14	randomised trials	not serious	serious[a]	not serious	serious[b]	none	573	576	-	MD **0.31 lower** (2.32 lower to 1.7 higher)	⊕⊕◯◯ Low	

Outcome - HDL

№ of studies	Study design	Risk of bias	Inconsistency	Indirectness	Imprecision	Other considerations	[n6]	[comparison]	Relative (95% CI)	Absolute (95% CI)	Certainty	Importance
20	randomised trials	not serious	serious[a]	not serious	serious[b]	none	612	618	-	MD **0.08 higher** (0.01 higher to 0.14 higher)	⊕⊕◯◯ Low	

Outcome - LDL

Certainty assessment							№ of patients		Effect		Certainty	Importance
№ of studies	Study design	Risk of bias	Inconsistency	Indirectness	Imprecision	Other considerations	[n6]	[comparison]	Relative (95% CI)	Absolute (95% CI)		
18	randomised trials	not serious	serious[a]	not serious	serious[b]	none	545	551	-	MD **0.39 lower** (0.71 lower to 0.06 lower)	⨁⨁◯◯ Low	
**Outcome - TAG**												
18	randomised trials	not serious	serious[a]	not serious	serious[b]	none	379	368	-	MD **0.23 lower** (0.61 lower to 0.14 higher)	⨁⨁◯◯ Low	
**Outcome - TC**												
18	randomised trials	not serious	not serious	not serious	not serious	none	547	403	-	MD **0.24 lower** (0.37 lower to 0.11 lower)	⨁⨁⨁⨁ High	
**Outcome - FBG**												

Certainty assessment							№ of patients		Effect		Certainty	Importance
№ of studies	Study design	Risk of bias	Inconsistency	Indirectness	Imprecision	Other considerations	[n6]	[comparison]	Relative (95% CI)	Absolute (95% CI)		
2	randomised trials	not serious	serious[a]	not serious	serious[b]	none	67	67	-	MD **0.22 higher** (0.19 lower to 0.63 higher)	⨁⨁◯◯ Low	

CI: confidence interval; **MD:** mean difference

Explanations

a. Rated down for inconsistency.

b. Rated down for imprecision.

Table S2-3. GRADE profile for n9 fatty acids supplementation.

Certainty assessment							№ of patients		Effect		Certainty	Importance
№ of studies	Study design	Risk of bias	Inconsistency	Indirectness	Imprecision	Other considerations	[n9]	[comparison]	Relative (95% CI)	Absolute (95% CI)		
**Outcome - SBP**												

Certainty assessment							№ of patients		Effect		Certainty	Importance
№ of studies	Study design	Risk of bias	Inconsistency	Indirectness	Imprecision	Other considerations	[n9]	[comparison]	Relative (95% CI)	Absolute (95% CI)		
3	randomised trials	not serious	not serious	not serious	serious[a]	none	48	43	-	MD **0.51 higher** (1.88 lower to 2.89 higher)	⊕⊕⊕◯ Moderate	
**Outcome - DBP**												
3	randomised trials	not serious	not serious	not serious	serious[a]	none	43	43	-	MD **0.55 lower** (3.8 lower to 2.7 higher)	⊕⊕⊕◯ Moderate	
**Outcome - HDL-C**												
16	randomised trials	not serious	not serious	not serious	serious[a]	none	303	303	-	MD **0.01 lower** (0.03 lower to 0.01 higher)	⊕⊕⊕◯ Moderate	
**Outcome - LDL-C**												

Certainty assessment							№ of patients		Effect		Certainty	Importance
№ of studies	Study design	Risk of bias	Inconsistency	Indirectness	Imprecision	Other considerations	[n9]	[comparison]	Relative (95% CI)	Absolute (95% CI)		
16	randomised trials	not serious	not serious	not serious	serious[a]	none	332	303	-	MD **0.06 lower** (0.15 lower to 0.03 higher)	⊕⊕⊕◯ Moderate	
**Outcome - TAG**												
16	randomised trials	not serious	not serious	not serious	serious[a]	none	303	303	-	MD **0.06 higher** (0.01 higher to 0.11 higher)	⊕⊕⊕◯ Moderate	
**Outcome - TC**												
15	randomised trials	not serious	serious[b]	not serious	serious[a]	none	285	285	-	MD **0.06 lower** (0.21 lower to 0.09 higher)	⊕⊕◯◯ Low	
**Outcome - FBG**												

Certainty assessment							№ of patients		Effect		Certainty	Importance
№ of studies	Study design	Risk of bias	Inconsistency	Indirectness	Imprecision	Other considerations	[n9]	[comparison]	Relative (95% CI)	Absolute (95% CI)		
3	randomised trials	not serious	serious[b]	not serious	serious[a]	none	53	53	-	MD **0.02 higher** (0.25 lower to 0.28 higher)	⊕⊕◯◯ Low	
**Outcome - FBI**												
3	randomised trials	not serious	serious[b]	not serious	serious[a]	none	53	53	-	MD **3.98 lower** (16.75 lower to 8.8 higher)	⊕⊕◯◯ Low	
**Outcome - HbA1c**												
2	randomised trials	not serious	not serious	not serious	serious[a]	none	33	33	-	MD **0.04 higher** (0.02 higher to 0.06 higher)	⊕⊕⊕◯ Moderate	

CI: confidence interval; **MD:** mean difference

Explanations

a. Rated down for imprecision.

b. Rated down for inconsistency.

Table S2-4. GRADE profile for lycopene supplementation.

Certainty assessment							№ of patients		Effect		Certainty	Importance
№ of studies	Study design	Risk of bias	Inconsistency	Indirectness	Imprecision	Other considerations	[lycopene]	[comparison]	Relative (95% CI)	Absolute (95% CI)		
**Outcome - SBP**												
15	randomised trials	not serious	serious[a]	not serious	serious[b]	none	499	462	-	MD **1.95 lower** (3.54 lower to 0.36 lower)	⨁⨁◯◯ Low	
**Outcome - DBP**												
11	randomised trials	not serious	not serious	not serious	serious[b]	none	333	296	-	MD **1.12 lower** (2.84 lower to 0.61 higher)	⨁⨁⨁◯ Moderate	

Certainty assessment							№ of patients		Effect		Certainty	Importance
№ of studies	Study design	Risk of bias	Inconsistency	Indirectness	Imprecision	Other considerations	[lycopene]	[comparison]	Relative (95% CI)	Absolute (95% CI)		
**Outcome - HDL**												
4	randomised trials	not serious	not serious	not serious	serious[b]	none	197	176	-	MD **0.03 lower** (0.12 lower to 0.06 higher)	⨁⨁⨁◯ Moderate	
**Outcome - LDL**												
4	randomised trials	not serious	not serious	not serious	serious[b]	none	197	176	-	MD **0.01 lower** (0.17 lower to 0.16 higher)	⨁⨁⨁◯ Moderate	
**Outcome - TG**												
2	randomised trials	not serious	not serious	not serious	serious[b]	none	149	152	-	MD **0.02 lower** (0.15 lower to 0.1 higher)	⨁⨁⨁◯ Moderate	

Certainty assessment							№ of patients		Effect		Certainty	Importance
№ of studies	Study design	Risk of bias	Inconsistency	Indirectness	Imprecision	Other considerations	[lycopene]	[comparison]	Relative (95% CI)	Absolute (95% CI)		
**Outcome - TC**												
2	randomised trials	not serious	not serious	not serious	serious[b]	none	149	149	-	MD **0** (0.22 lower to 0.22 higher)	⨁⨁⨁◯ Moderate	

CI: confidence interval; **MD:** mean difference

Explanations

a. Rated down for inconsistency.

b. Rated down for imprecision.

Table S2-5. GRADE profile for astaxanthin supplementation.

Certainty assessment							№ of patients		Effect		Certainty	Importance
№ of studies	Study design	Risk of bias	Inconsistency	Indirectness	Imprecision	Other considerations	[astaxanthin]	[comparison]	Relative (95% CI)	Absolute (95% CI)		
**outcome - SBP**												
5	randomised trials	not serious	serious[a]	not serious	serious[b]	none	91	87	-	MD **2.51 lower** (8.19 lower to 3.17 higher)	⨁⨁◯◯ Low	
**outcome - DBP**												
5	randomised trials	not serious	not serious	not serious	serious[b]	none	91	87	-	MD **0.79 lower** (3.42 lower to 1.84 higher)	⨁⨁⨁◯ Moderate	
**outcome - HDL**												
8	randomised trials	not serious	not serious	not serious	serious[b]	none	143	139	-	MD **0.13 higher** (0.05 higher to 0.21 higher)	⨁⨁⨁◯ Moderate	

Certainty assessment							№ of patients		Effect		Certainty	Importance
№ of studies	Study design	Risk of bias	Inconsistency	Indirectness	Imprecision	Other considerations	[astaxanthin]	[comparison]	Relative (95% CI)	Absolute (95% CI)		
**outcome - LDL**												
8	randomised trials	not serious	not serious	not serious	serious[b]	none	143	139	-	MD **0.11 lower** (0.21 lower to 0.01 lower)	⨁⨁⨁◯ Moderate	
**outcome - TG**												
8	randomised trials	not serious	serious[a]	not serious	not serious	none	143	139	-	MD **0.46 lower** (0.83 lower to 0.1 lower)	⨁⨁⨁◯ Moderate	
**outcome - TC**												
8	randomised trials	not serious	not serious	not serious	not serious	none	143	139	-	MD **0.22 lower** (0.32 lower to 0.12 lower)	⨁⨁⨁⨁ High	

Certainty assessment							№ of patients		Effect		Certainty	Importance
№ of studies	Study design	Risk of bias	Inconsistency	Indirectness	Imprecision	Other considerations	[astaxanthin]	[comparison]	Relative (95% CI)	Absolute (95% CI)		
**outcome - FBG**												
6	randomised trials	not serious	not serious	not serious	serious[b]	none	98	95	-	MD **0.15 lower** (0.32 lower to 0.02 higher)	⨁⨁⨁◯ Moderate	
**outcome - FBI**												
2	randomised trials	not serious	serious[a]	not serious	not serious	none	34	34	-	MD **2.66 lower** (3.98 lower to 1.34 lower)	⨁⨁⨁◯ Moderate	

CI: confidence interval; **MD:** mean difference

Explanations

a. Rated down for inconsistency.

b. Rated down for imprecision.

Table S2-6. GRADE profile for beta-carotene supplementation.

Certainty assessment							№ of patients		Effect		Certainty	Importance
№ of studies	Study design	Risk of bias	Inconsistency	Indirectness	Imprecision	Other considerations	[beta-carotene]	[comparison]	Relative (95% CI)	Absolute (95% CI)		
**outcome - HDL**												
6	randomised trials	not serious	not serious	not serious	serious[a]	none	152	145	-	MD **0.04 higher** (0.02 lower to 0.1 higher)	⨁⨁⨁◯ Moderate	
**outcome - LDL**												
3	randomised trials	not serious	not serious	not serious	serious[a]	none	85	79	-	MD **0.29 lower** (0.67 lower to 0.1 higher)	⨁⨁⨁◯ Moderate	
**outcome - TG**												

Certainty assessment							№ of patients		Effect		Certainty	Importance
№ of studies	Study design	Risk of bias	Inconsistency	Indirectness	Imprecision	Other considerations	[beta-carotene]	[comparison]	Relative (95% CI)	Absolute (95% CI)		
5	randomised trials	not serious	serious[b]	not serious	serious[a]	none	127	120	-	MD **0.3 lower** (0.78 lower to 0.18 higher)	⨁⨁◯◯ Low	
**outcome - TC**												
6	randomised trials	not serious	not serious	not serious	serious[a]	none	152	145	-	MD **0.13 lower** (0.23 lower to 0.04 lower)	⨁⨁⨁◯ Moderate	

CI: confidence interval; **MD:** mean difference

Explanations

a. Rated down for imprecision.

b. Rated down for inconsistency.

Table S2-7. GRADE profile for antioxidant lipids in the healthy population.

Certainty assessment							№ of patients		Effect		Certainty	Importance
№ of studies	Study design	Risk of bias	Inconsistency	Indirectness	Imprecision	Other considerations	[lipids]	[comparison]	Relative (95% CI)	Absolute (95% CI)		
**healthy - SBP**												
31	randomised trials	not serious	serious[a]	not serious	serious[b]	none	2132	2098	-	MD **0.8 lower** (2.99 lower to 1.38 higher)	⨁⨁◯◯ Low	
**healthy - DBP**												
27	randomised trials	not serious	serious[a]	not serious	serious[b]	none	2012	1983	-	MD **0.64 higher** (0.8 lower to 2.08 higher)	⨁⨁◯◯ Low	
**healthy - HDL**												
39	randomised trials	not serious	not serious	not serious	serious[b]	none	1141	1094	-	MD **0.03 higher** (0 to 0.06 higher)	⨁⨁⨁◯ Moderate	

Certainty assessment							№ of patients		Effect		Certainty	Importance
№ of studies	Study design	Risk of bias	Inconsistency	Indirectness	Imprecision	Other considerations	[lipids]	[comparison]	Relative (95% CI)	Absolute (95% CI)		
**healthy - LDL**												
36	randomised trials	not serious	serious[a]	not serious	serious[b]	none	1084	1028	-	MD **0.06 lower** (0.25 lower to 0.12 higher)	⨁⨁◯◯ Low	
**healthy - TG**												
34	randomised trials	not serious	serious[a]	not serious	serious[b]	none	1014	992	-	MD **0.15 lower** (0.25 lower to 0.06 lower)	⨁⨁◯◯ Low	
**healthy - TC**												
32	randomised trials	not serious	not serious	not serious	serious[b]	none	912	895	-	MD **0.02 lower** (0.07 lower to 0.04 higher)	⨁⨁⨁◯ Moderate	

Certainty assessment							№ of patients		Effect		Certainty	Importance
№ of studies	Study design	Risk of bias	Inconsistency	Indirectness	Imprecision	Other considerations	[lipids]	[comparison]	Relative (95% CI)	Absolute (95% CI)		
**healthy - FBG**												
9	randomised trials	not serious	serious[a]	not serious	serious[b]	none	347	330	-	MD **0.16 higher** (0.05 lower to 0.37 higher)	⨁⨁◯◯ Low	
**healthy - FBI**												
6	randomised trials	not serious	serious[a]	not serious	serious[b]	none	163	173	-	MD **3.6 lower** (11.91 lower to 4.71 higher)	⨁⨁◯◯ Low	

CI: confidence interval; **MD:** mean difference

Explanations

a. Rated down for inconsistency.

b. Rated down for imprecision.

Table S2-8. GRADE profile for antioxidant lipids in population with diabetes.

Certainty assessment							№ of patients		Effect		Certainty	Importance
№ of studies	Study design	Risk of bias	Inconsistency	Indirectness	Imprecision	Other considerations	[lipids]	[comparison]	Relative (95% CI)	Absolute (95% CI)		
**diabetes - SBP**												
15	randomised trials	not serious	serious[a]	not serious	serious[b]	none	564	543	-	MD **0.54 higher** (2.45 lower to 3.53 higher)	⊕⊕◯◯ Low	
**diabetes - DBP**												
12	randomised trials	not serious	not serious	not serious	serious[b]	none	332	314	-	MD **0.36 lower** (1.1 lower to 0.37 higher)	⊕⊕⊕◯ Moderate	
**diabetes - HDL**												

Certainty assessment							№ of patients		Effect		Certainty	Importance
№ of studies	Study design	Risk of bias	Inconsistency	Indirectness	Imprecision	Other considerations	[lipids]	[comparison]	Relative (95% CI)	Absolute (95% CI)		
16	randomised trials	not serious	serious[a]	not serious	serious[b]	none	647	626	-	MD **0.28 higher** (0.07 higher to 0.5 higher)	⨁⨁◯◯ Low	
**diabetes - LDL**												
14	randomised trials	not serious	not serious	not serious	serious[b]	none	567	571	-	MD **0.02 lower** (0.04 lower to 0)	⨁⨁⨁◯ Moderate	
**diabetes - TG**												
17	randomised trials	not serious	not serious	not serious	not serious	none	460	448	-	MD **0.26 lower** (0.37 lower to 0.15 lower)	⨁⨁⨁⨁ High	
**diabetes - TC**												

Certainty assessment							№ of patients		Effect		Certainty	Importance
№ of studies	Study design	Risk of bias	Inconsistency	Indirectness	Imprecision	Other considerations	[lipids]	[comparison]	Relative (95% CI)	Absolute (95% CI)		
18	randomised trials	not serious	serious[a]	not serious	serious[b]	none	445	488	-	MD **0.21 lower** (0.54 lower to 0.13 higher)	⊕⊕◯◯ Low	

diabetes - FBG

20	randomised trials	not serious	serious[a]	not serious	serious[b]	none	554	528	-	MD **0.03 higher** (0.32 lower to 0.38 higher)	⊕⊕◯◯ Low	

diabetes - FBI

9	randomised trials	not serious	serious[a]	not serious	serious[b]	none	341	252	-	MD **7.37 lower** (24.11 lower to 9.38 higher)	⊕⊕◯◯ Low	

diabetes - A1c

Certainty assessment							№ of patients		Effect		Certainty	Importance
№ of studies	Study design	Risk of bias	Inconsistency	Indirectness	Imprecision	Other considerations	[lipids]	[comparison]	Relative (95% CI)	Absolute (95% CI)		
19	randomised trials	not serious	serious[a]	not serious	serious[b]	none	687	673	-	MD **0.09 lower** (0.23 lower to 0.06 higher)	⨁⨁◯◯ Low	

CI: confidence interval; **MD:** mean difference

Explanations

a. Rated down for inconsistency.

b. Rated down for imprecision.

Table S2-9. GRADE profile for antioxidant lipids in population with hypertension.

Certainty assessment							№ of patients		Effect		Certainty	Importance
№ of studies	Study design	Risk of bias	Inconsistency	Indirectness	Imprecision	Other considerations	[lipids]	[comparison]	Relative (95% CI)	Absolute (95% CI)		
**hypertension - SBP**												

Certainty assessment							№ of patients		Effect		Certainty	Importance
№ of studies	Study design	Risk of bias	Inconsistency	Indirectness	Imprecision	Other considerations	[lipids]	[comparison]	Relative (95% CI)	Absolute (95% CI)		
15	randomised trials	not serious	serious[a]	not serious	serious[b]	none	418	411	-	MD **1.81 lower** (4.14 lower to 0.53 higher)	⊕⊕◯◯ Low	
**hypertension - DBP**												
15	randomised trials	not serious	not serious	not serious	serious[b]	none	371	363	-	MD **1.22 lower** (2.79 lower to 0.35 higher)	⊕⊕⊕◯ Moderate	
**hypertension - HDL**												
10	randomised trials	not serious	serious[a]	not serious	serious[b]	none	314	316	-	MD **0.04 lower** (0.16 lower to 0.08 higher)	⊕⊕◯◯ Low	
**hypertension - LDL**												

Certainty assessment							№ of patients		Effect		Certainty	Importance
№ of studies	Study design	Risk of bias	Inconsistency	Indirectness	Imprecision	Other considerations	[lipids]	[comparison]	Relative (95% CI)	Absolute (95% CI)		
7	randomised trials	not serious	not serious	not serious	serious[b]	none	214	178	-	MD **0.28 lower** (0.6 lower to 0.03 higher)	⊕⊕⊕◯ Moderate	
**hypertension - TG**												
9	randomised trials	not serious	not serious	not serious	serious[b]	none	289	291	-	MD **0.04 lower** (0.24 lower to 0.15 higher)	⊕⊕⊕◯ Moderate	
**hypertension - TC**												
8	randomised trials	not serious	not serious	not serious	not serious	none	263	274	-	MD **0.4 lower** (0.68 lower to 0.12 lower)	⊕⊕⊕⊕ High	

CI: confidence interval; **MD:** mean difference

Explanations

a. Rated down for inconsistency.

b. Rated down for imprecision.

Table S2-10. GRADE profile for antioxidant lipids in population with dyslipidemia.

Certainty assessment							№ of patients		Effect		Certainty	Importance
№ of studies	Study design	Risk of bias	Inconsistency	Indirectness	Imprecision	Other considerations	[lipids]	[comparison]	Relative (95% CI)	Absolute (95% CI)		
**dyslipidemia - SBP**												
15	randomised trials	not serious	serious[a]	not serious	serious[b]	none	386	390	-	MD **0.01 higher** (2.64 lower to 2.66 higher)	⨁⨁◯◯ Low	
**dyslipidemia - DBP**												
14	randomised trials	not serious	not serious	not serious	serious[b]	none	424	434	-	MD **0.81 lower** (1.51 lower to 0.1 lower)	⨁⨁⨁◯ Moderate	

Certainty assessment							№ of patients		Effect		Certainty	Importance
№ of studies	Study design	Risk of bias	Inconsistency	Indirectness	Imprecision	Other considerations	[lipids]	[comparison]	Relative (95% CI)	Absolute (95% CI)		
**dyslipidemia - HDL**												
23	randomised trials	not serious	serious[a]	not serious	serious[b]	none	910	693	-	MD **0.16 higher** (0.08 higher to 0.24 higher)	⨁⨁◯◯ Low	
**dyslipidemia - LDL**												
19	randomised trials	not serious	serious[a]	not serious	serious[b]	none	751	530	-	MD **0.01 lower** (0.25 lower to 0.22 higher)	⨁⨁◯◯ Low	
**dyslipidemia - TG**												
23	randomised trials	not serious	serious[a]	not serious	not serious	none	909	693	-	MD **0.56 lower** (0.81 lower to 0.3 lower)	⨁⨁⨁◯ Moderate	

Certainty assessment							№ of patients		Effect		Certainty	Importance
№ of studies	Study design	Risk of bias	Inconsistency	Indirectness	Imprecision	Other considerations	[lipids]	[comparison]	Relative (95% CI)	Absolute (95% CI)		
**dyslipidemia - TC**												
19	randomised trials	not serious	serious[a]	not serious	serious[b]	none	422	467	-	MD **0.01 higher** (0.14 lower to 0.16 higher)	⨁⨁◯◯ Low	
**dyslipidemia - FBG**												
9	randomised trials	not serious	serious[a]	not serious	serious[b]	none	154	185	-	MD **0.58 lower** (2.46 lower to 1.3 higher)	⨁⨁◯◯ Low	
**dyslipidemia - FBI**												
4	randomised trials	not serious	serious[a]	not serious	serious[b]	none	72	74	-	MD **5.49 lower** (11.71 lower to 0.73 higher)	⨁⨁◯◯ Low	

CI: confidence interval; **MD:** mean difference

Explanations

a. Rated down for inconsistency.

b. Rated down for imprecision.

Table S2-11. GRADE profile for antioxidant lipids in population with obesity.

Certainty assessment							№ of patients		Effect		Certainty	Importance
№ of studies	Study design	Risk of bias	Inconsistency	Indirectness	Imprecision	Other considerations	[lipids]	[comparison]	Relative (95% CI)	Absolute (95% CI)		
**obesity - SBP**												
8	randomised trials	not serious	serious[a]	not serious	serious[b]	none	394	393	-	MD **2.65 lower** (6.19 lower to 0.89 higher)	⨁⨁◯◯ Low	
**obesity - DBP**												

Certainty assessment							№ of patients		Effect		Certainty	Importance
№ of studies	Study design	Risk of bias	Inconsistency	Indirectness	Imprecision	Other considerations	[lipids]	[comparison]	Relative (95% CI)	Absolute (95% CI)		
7	randomised trials	not serious	serious[a]	not serious	serious[b]	none	374	373	-	MD **1.57 lower** (5.58 lower to 2.45 higher)	⨁⨁◯◯ Low	
**obesity - HDL**												
14	randomised trials	not serious	serious[a]	not serious	serious[b]	none	512	508	-	MD **0.06 higher** (0.02 higher to 0.1 higher)	⨁⨁◯◯ Low	
**obesity - LDL**												
14	randomised trials	not serious	serious[a]	not serious	serious[b]	none	609	508	-	MD **0.08 lower** (0.21 lower to 0.06 higher)	⨁⨁◯◯ Low	
**obesity - TG**												

Certainty assessment							№ of patients		Effect		Certainty	Importance
№ of studies	Study design	Risk of bias	Inconsistency	Indirectness	Imprecision	Other considerations	[lipids]	[comparison]	Relative (95% CI)	Absolute (95% CI)		
13	randomised trials	not serious	not serious	not serious	serious[b]	none	339	318	-	MD **0.13 lower** (0.24 lower to 0.02 lower)	⊕⊕⊕◯ Moderate	
**obesity - TC**												
13	randomised trials	not serious	serious[a]	not serious	serious[b]	none	569	318	-	MD **0.21 lower** (0.64 lower to 0.22 higher)	⊕⊕◯◯ Low	
**obesity - FBG**												
6	randomised trials	not serious	serious[a]	not serious	serious[b]	none	146	161	-	MD **1.1 lower** (3.79 lower to 1.6 higher)	⊕⊕◯◯ Low	
**obesity - FBI**												

Certainty assessment							№ of patients		Effect		Certainty	Importance
№ of studies	Study design	Risk of bias	Inconsistency	Indirectness	Imprecision	Other considerations	[lipids]	[comparison]	Relative (95% CI)	Absolute (95% CI)		
6	randomised trials	not serious	serious[a]	not serious	serious[b]	none	267	272	-	MD **4.45 lower** (16.84 lower to 7.94 higher)	⨁⨁◯◯ Low	

CI: confidence interval; **MD:** mean difference

Explanations

a. Rated down for inconsistency.

b. Rated down for imprecision.

Table S2-12. GRADE profile for antioxidant lipids in population with metabolic syndrome.

Certainty assessment							№ of patients		Effect		Certainty	Importance
№ of studies	Study design	Risk of bias	Inconsistency	Indirectness	Imprecision	Other considerations	[lipids]	[comparison]	Relative (95% CI)	Absolute (95% CI)		
**MetS - SBP**												

Certainty assessment							№ of patients		Effect		Certainty	Importance
№ of studies	Study design	Risk of bias	Inconsistency	Indirectness	Imprecision	Other considerations	[lipids]	[comparison]	Relative (95% CI)	Absolute (95% CI)		
3	randomised trials	not serious	not serious	not serious	serious[a]	none	98	93	-	MD **2.76 lower** (7.6 lower to 2.09 higher)	⊕⊕⊕◯ Moderate	
**MetS - DBP**												
3	randomised trials	not serious	not serious	not serious	not serious	none	98	93	-	MD **3 lower** (4.15 lower to 1.85 lower)	⊕⊕⊕⊕ High	
**MetS - HDL**												
6	randomised trials	not serious	not serious	not serious	serious[a]	none	176	171	-	MD **0** (0.04 lower to 0.03 higher)	⊕⊕⊕◯ Moderate	
**MetS - LDL**												

Certainty assessment							№ of patients		Effect		Certainty	Importance
№ of studies	Study design	Risk of bias	Inconsistency	Indirectness	Imprecision	Other considerations	[lipids]	[comparison]	Relative (95% CI)	Absolute (95% CI)		
5	randomised trials	not serious	not serious	not serious	serious[a]	none	180	173	-	MD **0.04 higher** (0.05 lower to 0.13 higher)	⨁⨁⨁◯ Moderate	
**MetS - TG**												
6	randomised trials	not serious	serious[b]	not serious	serious[a]	none	176	171	-	MD **0.55 lower** (1.24 lower to 0.14 higher)	⨁⨁◯◯ Low	
**MetS - TC**												
5	randomised trials	not serious	serious[b]	not serious	serious[a]	none	206	153	-	MD **0.03 lower** (0.47 lower to 0.4 higher)	⨁⨁◯◯ Low	

CI: confidence interval; **MD:** mean difference

Explanations

a. Rated down for imprecision.

b. Rated down for inconsistency.

Supplemental 6 - Results of sensitivity analysis of the systematic removal of each study

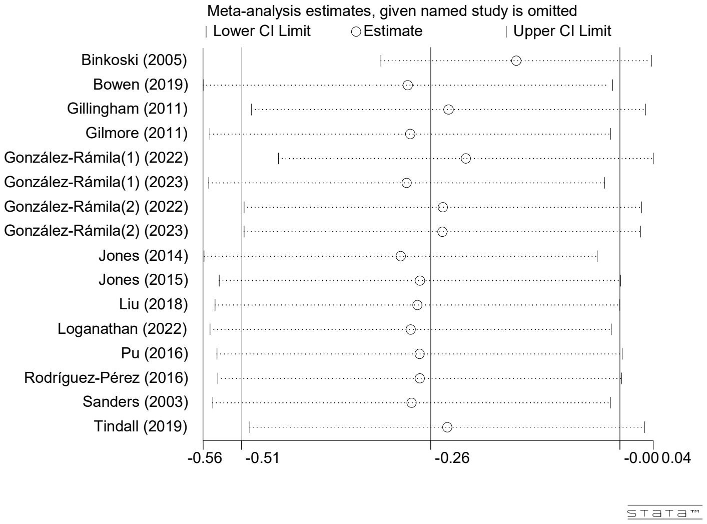

Figure S4-1 Sensitivity analysis of the systematic removal of each study for LDL-C in participants supplemented with n9 fatty acid.

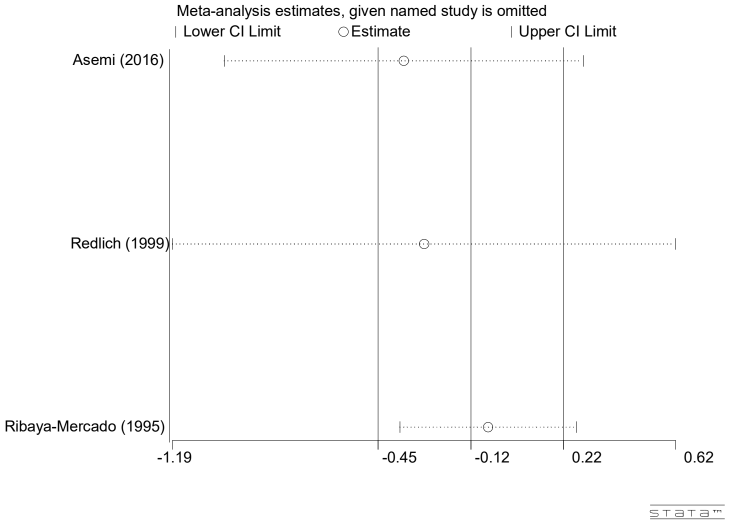

Figure S4-2 Sensitivity analysis of the systematic removal of each study for LDL-C in participants supplemented with beta-carotene.

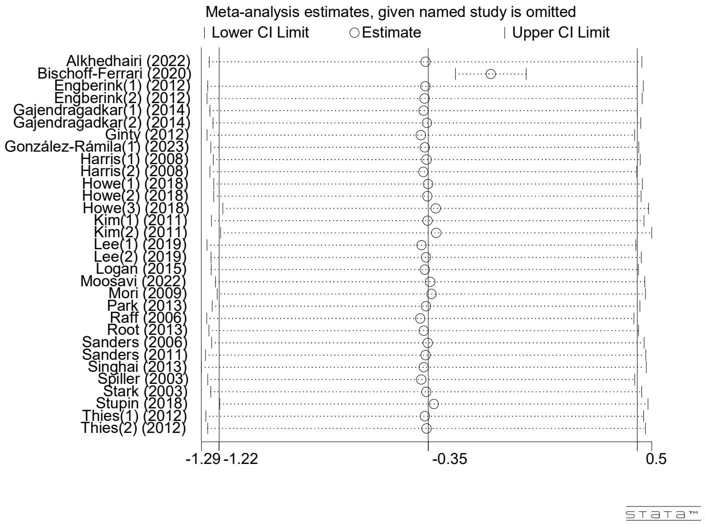

Figure S4-3 Sensitivity analysis of the systematic removal of each study for SBP in the healthy population.

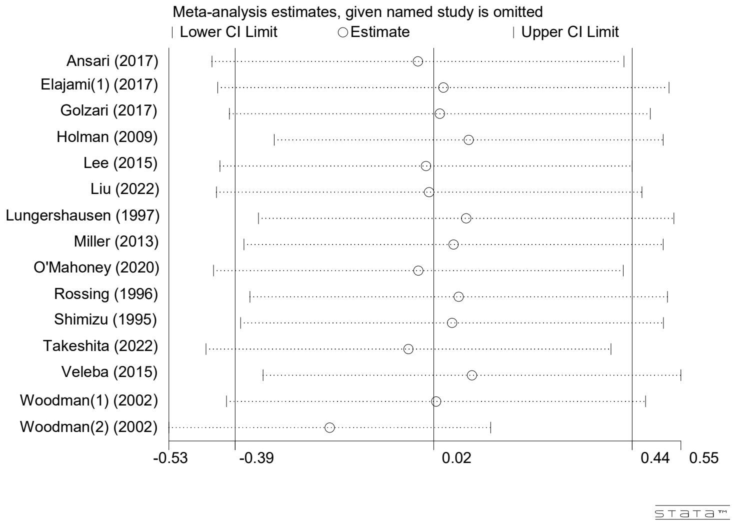

Figure S4-4 Sensitivity analysis of the systematic removal of each study for SBP in the population with diabetes.

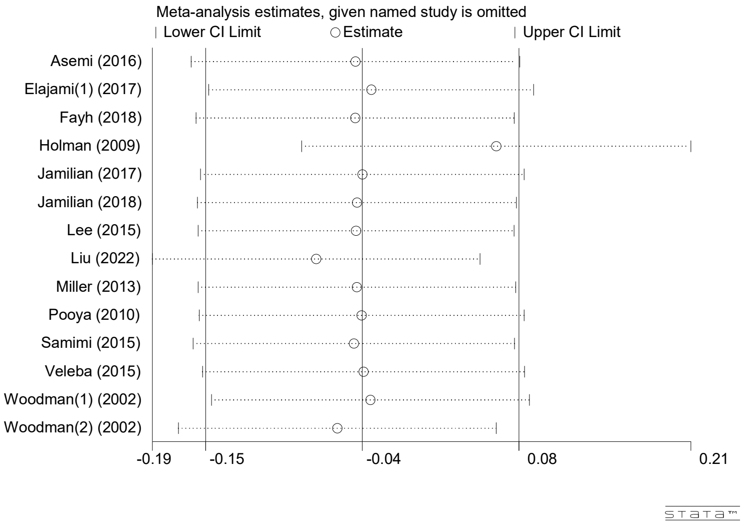

Figure S4-5 Sensitivity analysis of the systematic removal of each study for LDL-C in the population with diabetes.

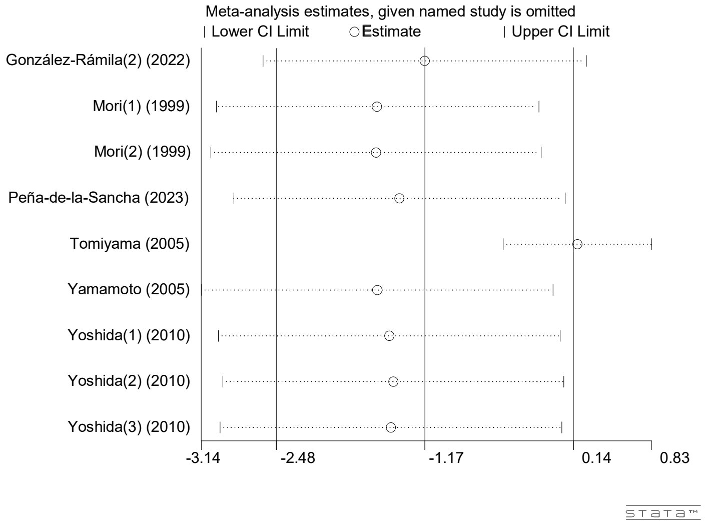

Figure S4-6 Sensitivity analysis of the systematic removal of each study for FBG in the population with dyslipidemia.

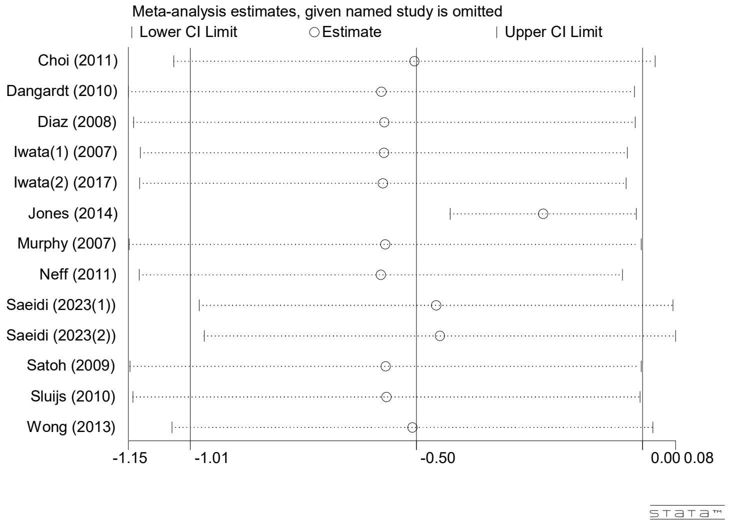

Figure S4-7 Sensitivity analysis of the systematic removal of each study for TC in the population with obesity.

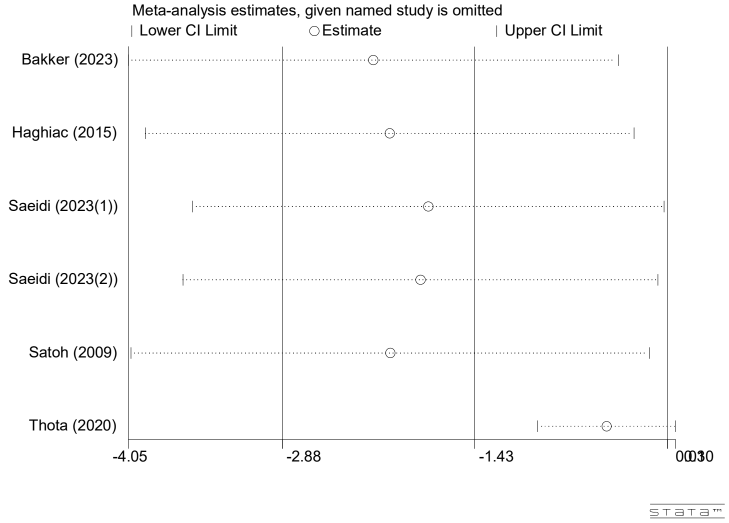

Figure S4-8 Sensitivity analysis of the systematic removal of each study for FBG in the population with obesity.

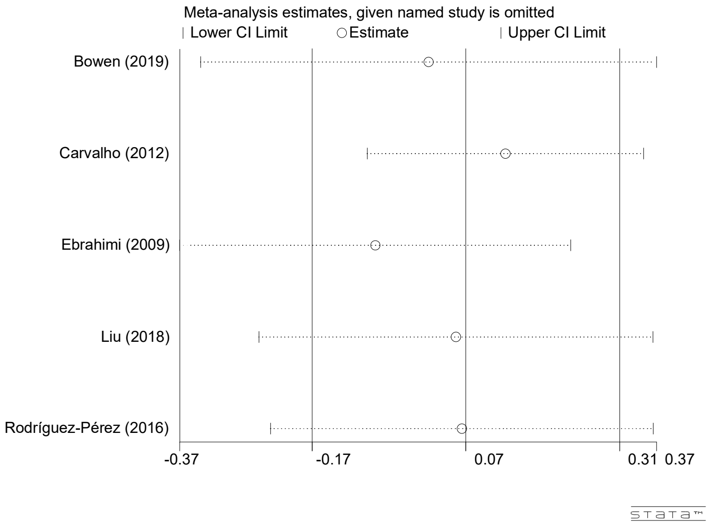

Figure S4-9 Sensitivity analysis of the systematic removal of each study for LDL-C in the population with metabolic syndrome.

Reference

1. Abbott KA, Burrows TL, Acharya S, Thota RN, Garg ML. DHA-enriched fish oil reduces insulin resistance in overweight and obese adults. Prostaglandins Leukot Essent Fatty Acids 2020;159:102154.
2. Agh F, Mohammadzadeh Honarvar N, Djalali M et al. Omega-3 Fatty Acid Could Increase One of Myokines in Male Patients with Coronary Artery Disease: A Randomized, Double-Blind, Placebo-Controlled Trial. Arch Iran Med 2017;20:28-33.
3. Alkhedhairi SA, Aba Alkhayl FF, Ismail AD et al. The effect of krill oil supplementation on skeletal muscle function and size in older adults: A randomised controlled trial. Clin Nutr 2022;41:1228-1235.
4. Ansari S, Djalali M, Mohammadzadeh Honarvar N et al. The Effect of n-3 Polyunsaturated Fatty Acids Supplementation on Serum Irisin in Patients with Type 2 Diabetes: A Randomized, Double-Blind, Placebo-Controlled Trial. Int J Endocrinol Metab 2017;15:e40614.
5. Bakker N, Hickey M, Shams R et al. Oral ω-3 PUFA supplementation modulates inflammation in adipose tissue depots in morbidly obese women: A randomized trial. Nutrition 2023;111:112055.
6. Barbosa MM, Melo AL, Damasceno NR. The benefits of ω-3 supplementation depend on adiponectin basal level and adiponectin increase after the supplementation: A randomized clinical trial. Nutrition 2017;34:7-13.
7. Bischoff-Ferrari HA, Vellas B, Rizzoli R et al. Effect of Vitamin D Supplementation, Omega-3 Fatty Acid Supplementation, or a Strength-Training Exercise Program on Clinical Outcomes in Older Adults: The DO-HEALTH Randomized Clinical Trial. Jama 2020;324:1855-1868.
8. Cazzola R, Russo-Volpe S, Miles EA et al. Age- and dose-dependent effects of an eicosapentaenoic acid-rich oil on cardiovascular risk factors in healthy male subjects. Atherosclerosis 2007;193:159-67.
9. Cussons AJ, Watts GF, Mori TA, Stuckey BG. Omega-3 fatty acid supplementation decreases liver fat content in polycystic ovary syndrome: a randomized controlled trial employing proton magnetic resonance spectroscopy. J Clin Endocrinol Metab 2009;94:3842-8.
10. Dangardt F, Osika W, Chen Y et al. Omega-3 fatty acid supplementation improves vascular function and reduces inflammation in obese adolescents. Atherosclerosis 2010;212:580-5.
11. De Giuseppe R, Di Napoli I, Tomasinelli CE et al. The Effect of Crackers Enriched with Camelina Sativa Oil on Omega-3 Serum Fatty Acid Composition in Older Adults: A Randomized Placebo-Controlled Pilot Trial. J Nutr Health Aging 2023;27:463-471.
12. de Lorgeril M, Renaud S, Mamelle N et al. Mediterranean alpha-linolenic acid-rich diet in secondary prevention of coronary heart disease. Lancet 1994;343:1454-9.
13. Derosa G, Cicero AF, Fogari E et al. Effects of n-3 PUFAs on postprandial

variation of metalloproteinases, and inflammatory and insulin resistance parameters in dyslipidemic patients: evaluation with euglycemic clamp and oral fat load. J Clin Lipidol 2012;6:553-64.
14. Dogay Us G, Mushtaq S. N-3 fatty acid supplementation mediates lipid profile, including small dense LDL, when combined with statins: a randomized double blind placebo controlled trial. Lipids Health Dis 2022;21:84.
15. Du Plooy WJ, Venter CP, Muntingh GM, Venter HL, Glatthaar, II, Smith KA. The cumulative dose response effect of eicosapentaenoic and docosahexaenoic acid on blood pressure, plasma lipid profile and diet pattern in mild to moderate essential hypertensive black patients. Prostaglandins Leukot Essent Fatty Acids 1992;46:315-21.
16. Ebrahimi M, Ghayour-Mobarhan M, Rezaiean S et al. Omega-3 fatty acid supplements improve the cardiovascular risk profile of subjects with metabolic syndrome, including markers of inflammation and auto-immunity. Acta Cardiol 2009;64:321-7.
17. Elajami TK, Alfaddagh A, Lakshminarayan D, Soliman M, Chandnani M, Welty FK. Eicosapentaenoic and Docosahexaenoic Acids Attenuate Progression of Albuminuria in Patients With Type 2 Diabetes Mellitus and Coronary Artery Disease. J Am Heart Assoc 2017;6.
18. Fayh APT, Borges K, Cunha GS et al. Effects of n-3 fatty acids and exercise on oxidative stress parameters in type 2 diabetic: a randomized clinical trial. J Int Soc Sports Nutr 2018;15:18.
19. Félix-Soriano E, Martínez-Gayo A, Cobo MJ et al. Effects of DHA-Rich n-3 Fatty Acid Supplementation and/or Resistance Training on Body Composition and Cardiometabolic Biomarkers in Overweight and Obese Post-Menopausal Women. Nutrients 2021;13.
20. Finnegan YE, Minihane AM, Leigh-Firbank EC et al. Plant- and marine-derived n-3 polyunsaturated fatty acids have differential effects on fasting and postprandial blood lipid concentrations and on the susceptibility of LDL to oxidative modification in moderately hyperlipidemic subjects. Am J Clin Nutr 2003;77:783-95.
21. Ginty AT, Conklin SM. Preliminary evidence that acute long-chain omega-3 supplementation reduces cardiovascular reactivity to mental stress: a randomized and placebo controlled trial. Biol Psychol 2012;89:269-72.
22. Golpour P, Nourbakhsh M, Mazaherioun M, Janani L, Nourbakhsh M, Yaghmaei P. Improvement of NRF2 gene expression and antioxidant status in patients with type 2 diabetes mellitus after supplementation with omega-3 polyunsaturated fatty acids: A double-blind randomised placebo-controlled clinical trial. Diabetes Res Clin Pract 2020;162:108120.
23. Golzari MH, Hosseini S, Koohdani F et al. The Effect of Eicosapentaenoic Acid on the Serum Levels and Enzymatic Activity of Paraoxonase 1 in the Patients With Type 2 Diabetes Mellitus. Acta Med Iran 2017;55:486-495.
24. Haghiac M, Yang XH, Presley L et al. Dietary Omega-3 Fatty Acid Supplementation Reduces Inflammation in Obese Pregnant Women: A

Randomized Double-Blind Controlled Clinical Trial. PLoS One 2015;10:e0137309.
25. Harris WS, Lemke SL, Hansen SN et al. Stearidonic acid-enriched soybean oil increased the omega-3 index, an emerging cardiovascular risk marker. Lipids 2008;43:805-11.
26. Howe PRC, Evans HM, Kuszewski JC, Wong RHX. Effects of Long Chain Omega-3 Polyunsaturated Fatty Acids on Brain Function in Mildly Hypertensive Older Adults. Nutrients 2018;10.
27. Jamilian M, Samimi M, Ebrahimi FA et al. The effects of vitamin D and omega-3 fatty acid co-supplementation on glycemic control and lipid concentrations in patients with gestational diabetes. J Clin Lipidol 2017;11:459-468.
28. Jamilian M, Samimi M, Mirhosseini N et al. A Randomized Double-Blinded, Placebo-Controlled Trial Investigating the Effect of Fish Oil Supplementation on Gene Expression Related to Insulin Action, Blood Lipids, and Inflammation in Gestational Diabetes Mellitus-Fish Oil Supplementation and Gestational Diabetes. Nutrients 2018;10.
29. Khani B, Mardanian F, Fesharaki SJ. Omega-3 supplementation effects on polycystic ovary syndrome symptoms and metabolic syndrome. J Res Med Sci 2017;22:64.
30. Kobayashi Y, Fujikawa T, Haruna A et al. Omega-3 Fatty Acids Reduce Remnant-like Lipoprotein Cholesterol and Improve the Ankle-Brachial Index of Hemodialysis Patients with Dyslipidemia: A Pilot Study. Medicina (Kaunas) 2023;60.
31. Lee SM, Chung SH, Park Y et al. Effect of Omega-3 Fatty Acid on the Fatty Acid Content of the Erythrocyte Membrane and Proteinuria in Patients with Diabetic Nephropathy. Int J Endocrinol 2015;2015:208121.
32. Lee JB, Notay K, Klingel SL, Chabowski A, Mutch DM, Millar PJ. Docosahexaenoic acid reduces resting blood pressure but increases muscle sympathetic outflow compared with eicosapentaenoic acid in healthy men and women. Am J Physiol Heart Circ Physiol 2019;316:H873-h881.
33. Liu H, Wang F, Liu X et al. Effects of marine-derived and plant-derived omega-3 polyunsaturated fatty acids on erythrocyte fatty acid composition in type 2 diabetic patients. Lipids Health Dis 2022;21:20.
34. Logan SL, Spriet LL. Omega-3 Fatty Acid Supplementation for 12 Weeks Increases Resting and Exercise Metabolic Rate in Healthy Community-Dwelling Older Females. PLoS One 2015;10:e0144828.
35. Lungershausen YK, Howe PR, Clifton PM et al. Evaluation of an omega-3 fatty acid supplement in diabetics with microalbuminuria. Ann N Y Acad Sci 1997;827:369-81.
36. Manor I, Magen A, Keidar D et al. Safety of phosphatidylserine containing omega3 fatty acids in ADHD children: a double-blind placebo-controlled trial followed by an open-label extension. Eur Psychiatry 2013;28:386-91.
37. Mejia-Montilla J, Reyna-Villasmil E, Domínguez-Brito L et al. Supplementation with omega-3 fatty acids and plasma adiponectin in women

with polycystic ovary syndrome. Endocrinol Diabetes Nutr (Engl Ed) 2018;65:192-199.

38. Mengelberg A, Leathem J, Podd J, Hill S, Conlon C. The effects of docosahexaenoic acid supplementation on cognition and well-being in mild cognitive impairment: A 12-month randomised controlled trial. Int J Geriatr Psychiatry 2022;37.

39. Miller ER, 3rd, Juraschek SP, Anderson CA et al. The effects of n-3 long-chain polyunsaturated fatty acid supplementation on biomarkers of kidney injury in adults with diabetes: results of the GO-FISH trial. Diabetes Care 2013;36:1462-9.

40. Miyajima T, Tsujino T, Saito K, Yokoyama M. Effects of eicosapentaenoic acid on blood pressure, cell membrane fatty acids, and intracellular sodium concentration in essential hypertension. Hypertens Res 2001;24:537-42.

41. Moosavi D, Vuckovic I, Kunz HE, Lanza IR. A randomized trial of omega-3 fatty acid supplementation and circulating lipoprotein subclasses in healthy older adults. J Nutr 2022.

42. Mori TA, Burke V, Puddey I et al. The effects of [omega]3 fatty acids and coenzyme Q10 on blood pressure and heart rate in chronic kidney disease: a randomized controlled trial. J Hypertens 2009;27:1863-72.

43. Mori TA, Burke V, Puddey IB et al. Purified eicosapentaenoic and docosahexaenoic acids have differential effects on serum lipids and lipoproteins, LDL particle size, glucose, and insulin in mildly hyperlipidemic men. Am J Clin Nutr 2000;71:1085-94.

44. Mozaffarian D, Maki KC, Bays HE et al. Effectiveness of a Novel ω-3 Krill Oil Agent in Patients With Severe Hypertriglyceridemia: A Randomized Clinical Trial. JAMA Netw Open 2022;5:e2141898.

45. Murphy KJ, Meyer BJ, Mori TA et al. Impact of foods enriched with n-3 long-chain polyunsaturated fatty acids on erythrocyte n-3 levels and cardiovascular risk factors. Br J Nutr 2007;97:749-57.

46. Neff LM, Culiner J, Cunningham-Rundles S et al. Algal docosahexaenoic acid affects plasma lipoprotein particle size distribution in overweight and obese adults. J Nutr 2011;141:207-13.

47. O'Mahoney LL, Dunseath G, Churm R et al. Omega-3 polyunsaturated fatty acid supplementation versus placebo on vascular health, glycaemic control, and metabolic parameters in people with type 1 diabetes: a randomised controlled preliminary trial. Cardiovasc Diabetol 2020;19:127.

48. Peña-de-la-Sancha P, Muñoz-García A, Espínola-Zavaleta N et al. Eicosapentaenoic and Docosahexaenoic Acid Supplementation Increases HDL Content in n-3 Fatty Acids and Improves Endothelial Function in Hypertriglyceridemic Patients. Int J Mol Sci 2023;24.

49. Pooya S, Jalali MD, Jazayery AD, Saedisomeolia A, Eshraghian MR, Toorang F. The efficacy of omega-3 fatty acid supplementation on plasma homocysteine and malondialdehyde levels of type 2 diabetic patients. Nutr Metab Cardiovasc Dis 2010;20:326-31.

50. Rafraf M, Mohammadi E, Asghari-Jafarabadi M, Farzadi L. Omega-3 fatty acids improve glucose metabolism without effects on obesity values and serum visfatin levels in women with polycystic ovary syndrome. J Am Coll Nutr 2012;31:361-8.
51. Root M, Collier SR, Zwetsloot KA, West KL, McGinn MC. A randomized trial of fish oil omega-3 fatty acids on arterial health, inflammation, and metabolic syndrome in a young healthy population. Nutr J 2013;12:40.
52. Rossing P, Hansen BV, Nielsen FS, Myrup B, Hølmer G, Parving HH. Fish oil in diabetic nephropathy. Diabetes Care 1996;19:1214-9.
53. Samimi M, Jamilian M, Asemi Z, Esmaillzadeh A. Effects of omega-3 fatty acid supplementation on insulin metabolism and lipid profiles in gestational diabetes: Randomized, double-blind, placebo-controlled trial. Clin Nutr 2015;34:388-93.
54. Sanders TA, Gleason K, Griffin B, Miller GJ. Influence of an algal triacylglycerol containing docosahexaenoic acid (22 : 6n-3) and docosapentaenoic acid (22 : 5n-6) on cardiovascular risk factors in healthy men and women. Br J Nutr 2006;95:525-31.
55. Sanders TA, Hall WL, Maniou Z, Lewis F, Seed PT, Chowienczyk PJ. Effect of low doses of long-chain n-3 PUFAs on endothelial function and arterial stiffness: a randomized controlled trial. Am J Clin Nutr 2011;94:973-80.
56. Sarbolouki S, Javanbakht MH, Derakhshanian H et al. Eicosapentaenoic acid improves insulin sensitivity and blood sugar in overweight type 2 diabetes mellitus patients: a double-blind randomised clinical trial. Singapore Med J 2013;54:387-90.
57. Satoh N, Shimatsu A, Kotani K et al. Highly purified eicosapentaenoic acid reduces cardio-ankle vascular index in association with decreased serum amyloid A-LDL in metabolic syndrome. Hypertens Res 2009;32:1004-8.
58. Shimizu H, Ohtani K, Tanaka Y, Sato N, Mori M, Shimomura Y. Long-term effect of eicosapentaenoic acid ethyl (EPA-E) on albuminuria of non-insulin dependent diabetic patients. Diabetes Res Clin Pract 1995;28:35-40.
59. Singhal A, Lanigan J, Storry C et al. Docosahexaenoic acid supplementation, vascular function and risk factors for cardiovascular disease: a randomized controlled trial in young adults. J Am Heart Assoc 2013;2:e000283.
60. Stark KD, Holub BJ. Differential eicosapentaenoic acid elevations and altered cardiovascular disease risk factor responses after supplementation with docosahexaenoic acid in postmenopausal women receiving and not receiving hormone replacement therapy. Am J Clin Nutr 2004;79:765-73.
61. Stupin A, Rasic L, Matic A et al. Omega-3 polyunsaturated fatty acids-enriched hen eggs consumption enhances microvascular reactivity in young healthy individuals. Appl Physiol Nutr Metab 2018;43:988-995.
62. Takaki A, Umemoto S, Ono K et al. Add-on therapy of EPA reduces oxidative stress and inhibits the progression of aortic stiffness in patients with coronary artery disease and statin therapy: a randomized controlled study. J Atheroscler Thromb 2011;18:857-66.
63. Takeshita Y, Teramura C, Kamoshita K et al. Effects of eicosapentaenoic acid

on serum levels of selenoprotein P and organ-specific insulin sensitivity in humans with dyslipidemia and type 2 diabetes. J Diabetes Investig 2022;13:532-542.

64. Theobald HE, Goodall AH, Sattar N, Talbot DC, Chowienczyk PJ, Sanders TA. Low-dose docosahexaenoic acid lowers diastolic blood pressure in middle-aged men and women. J Nutr 2007;137:973-8.
65. Thota RN, Rosato JI, Burrows TL et al. Docosahexaenoic Acid-Rich Fish Oil Supplementation Reduces Kinase Associated with Insulin Resistance in Overweight and Obese Midlife Adults. Nutrients 2020;12.
66. Tomiyama H, Takazawa K, Osa S et al. Do eicosapentaenoic acid supplements attenuate age-related increases in arterial stiffness in patients with dyslipidemia?: A preliminary study. Hypertens Res 2005;28:651-5.
67. Tsunoda F, Lamon-Fava S, Asztalos BF, Iyer LK, Richardson K, Schaefer EJ. Effects of oral eicosapentaenoic acid versus docosahexaenoic acid on human peripheral blood mononuclear cell gene expression. Atherosclerosis 2015;241:400-8.
68. Udupa A, Nahar P, Shah S, Kshirsagar M, Ghongane B. A comparative study of effects of omega-3 Fatty acids, alpha lipoic Acid and vitamin e in type 2 diabetes mellitus. Ann Med Health Sci Res 2013;3:442-6.
69. Vakhapova V, Richter Y, Cohen T, Herzog Y, Korczyn AD. Safety of phosphatidylserine containing omega-3 fatty acids in non-demented elderly: a double-blind placebo-controlled trial followed by an open-label extension. BMC Neurol 2011;11:79.
70. Veleba J, Kopecky J, Jr., Janovska P et al. Combined intervention with pioglitazone and n-3 fatty acids in metformin-treated type 2 diabetic patients: improvement of lipid metabolism. Nutr Metab (Lond) 2015;12:52.
71. West SG, Krick AL, Klein LC et al. Effects of diets high in walnuts and flax oil on hemodynamic responses to stress and vascular endothelial function. J Am Coll Nutr 2010;29:595-603.
72. Wong AT, Chan DC, Barrett PH, Adams LA, Watts GF. Supplementation with n3 fatty acid ethyl esters increases large and small artery elasticity in obese adults on a weight loss diet. J Nutr 2013;143:437-41.
73. Woodman RJ, Mori TA, Burke V, Puddey IB, Watts GF, Beilin LJ. Effects of purified eicosapentaenoic and docosahexaenoic acids on glycemic control, blood pressure, and serum lipids in type 2 diabetic patients with treated hypertension. Am J Clin Nutr 2002;76:1007-15.
74. Yamamoto T, Kajikawa Y, Otani S et al. Protective effect of eicosapentaenoic acid on insulin resistance in hyperlipidemic patients and on the postoperative course of cardiac surgery patients: the possible involvement of adiponectin. Acta Med Okayama 2014;68:349-61.
75. Zeman M, Zák A, Vecka M, Tvrzická E, Písaríková A, Stanková B. N-3 fatty acid supplementation decreases plasma homocysteine in diabetic dyslipidemia treated with statin-fibrate combination. J Nutr Biochem 2006;17:379-84.
76. Aryaeian N, Shahram F, Djalali M et al. Effect of conjugated linoleic acid,

vitamin E and their combination on lipid profiles and blood pressure of Iranian adults with active rheumatoid arthritis. Vasc Health Risk Manag 2008;4:1423-32.

77. Carvalho RF, Uehara SK, Rosa G. Microencapsulated conjugated linoleic acid associated with hypocaloric diet reduces body fat in sedentary women with metabolic syndrome. Vasc Health Risk Manag 2012;8:661-7.
78. Damsgaard CT, Frøkiaer H, Andersen AD, Lauritzen L. Fish oil in combination with high or low intakes of linoleic acid lowers plasma triacylglycerols but does not affect other cardiovascular risk markers in healthy men. J Nutr 2008;138:1061-6.
79. Diaz ML, Watkins BA, Li Y, Anderson RA, Campbell WW. Chromium picolinate and conjugated linoleic acid do not synergistically influence diet- and exercise-induced changes in body composition and health indexes in overweight women. J Nutr Biochem 2008;19:61-8.
80. Dullaart RP, Beusekamp BJ, Meijer S, Hoogenberg K, van Doormaal JJ, Sluiter WJ. Long-term effects of linoleic-acid-enriched diet on albuminuria and lipid levels in type 1 (insulin-dependent) diabetic patients with elevated urinary albumin excretion. Diabetologia 1992;35:165-72.
81. Engberink MF, Geleijnse JM, Wanders AJ, Brouwer IA. The effect of conjugated linoleic acid, a natural trans fat from milk and meat, on human blood pressure: results from a randomized crossover feeding study. J Hum Hypertens 2012;26:127-32.
82. Ferrara LA, Raimondi AS, d'Episcopo L, Guida L, Dello Russo A, Marotta T. Olive oil and reduced need for antihypertensive medications. Arch Intern Med 2000;160:837-42.
83. Iwata T, Kamegai T, Yamauchi-Sato Y et al. Safety of dietary conjugated linoleic acid (CLA) in a 12-weeks trial in healthy overweight Japanese male volunteers. J Oleo Sci 2007;56:517-25.
84. Kaźmierska A, Bolesławska I, Jagielski P et al. Effect of Evening Primrose Oil Supplementation on Biochemical Parameters and Nutrition of Patients Treated with Isotretinoin for Acne Vulgaris: A Randomized Double-Blind Trial. Nutrients 2022;14.
85. Kestin M, Clifton P, Belling GB, Nestel PJ. n-3 fatty acids of marine origin lower systolic blood pressure and triglycerides but raise LDL cholesterol compared with n-3 and n-6 fatty acids from plants. Am J Clin Nutr 1990;51:1028-34.
86. Kriketos AD, Robertson RM, Sharp TA et al. Role of weight loss and polyunsaturated fatty acids in improving metabolic fitness in moderately obese, moderately hypertensive subjects. J Hypertens 2001;19:1745-54.
87. Laidlaw M, Holub BJ. Effects of supplementation with fish oil-derived n-3 fatty acids and gamma-linolenic acid on circulating plasma lipids and fatty acid profiles in women. Am J Clin Nutr 2003;77:37-42.
88. Lee SP, Dart AM, Walker KZ, O'Dea K, Chin-Dusting JP, Skilton MR. Effect of altering dietary n-6:n-3 PUFA ratio on cardiovascular risk measures in

patients treated with statins: a pilot study. Br J Nutr 2012;108:1280-5.
89. Leng GC, Lee AJ, Fowkes FG et al. Randomized controlled trial of gamma-linolenic acid and eicosapentaenoic acid in peripheral arterial disease. Clin Nutr 1998;17:265-71.
90. Miller M, Sorkin JD, Mastella L et al. Poly is more effective than monounsaturated fat for dietary management in the metabolic syndrome: The muffin study. J Clin Lipidol 2016;10:996-1003.
91. Raff M, Tholstrup T, Sejrsen K, Straarup EM, Wiinberg N. Diets rich in conjugated linoleic acid and vaccenic acid have no effect on blood pressure and isobaric arterial elasticity in healthy young men. J Nutr 2006;136:992-7.
92. Binkoski AE, Kris-Etherton PM, Wilson TA, Mountain ML, Nicolosi RJ. Balance of unsaturated fatty acids is important to a cholesterol-lowering diet: comparison of mid-oleic sunflower oil and olive oil on cardiovascular disease risk factors. J Am Diet Assoc 2005;105:1080-6.
93. Bowen KJ, Kris-Etherton PM, West SG et al. Diets Enriched with Conventional or High-Oleic Acid Canola Oils Lower Atherogenic Lipids and Lipoproteins Compared to a Diet with a Western Fatty Acid Profile in Adults with Central Adiposity. J Nutr 2019;149:471-478.
94. Gillingham LG, Gustafson JA, Han SY, Jassal DS, Jones PJ. High-oleic rapeseed (canola) and flaxseed oils modulate serum lipids and inflammatory biomarkers in hypercholesterolaemic subjects. Br J Nutr 2011;105:417-27.
95. Gilmore LA, Walzem RL, Crouse SF et al. Consumption of high-oleic acid ground beef increases HDL-cholesterol concentration but both high- and low-oleic acid ground beef decrease HDL particle diameter in normocholesterolemic men. J Nutr 2011;141:1188-94.
96. González-Rámila S, Sarriá B, Seguido M, García-Cordero J, Bravo-Clemente L, Mateos R. Effect of Olive Pomace Oil on Cardiovascular Health and Associated Pathologies. Nutrients 2022;14.
97. González-Rámila S, Sarriá B, Seguido MA, García-Cordero J, Mateos R, Bravo L. Olive pomace oil can improve blood lipid profile: a randomized, blind, crossover, controlled clinical trial in healthy and at-risk volunteers. Eur J Nutr 2023;62:589-603.
98. Jones PJ, Senanayake VK, Pu S et al. DHA-enriched high-oleic acid canola oil improves lipid profile and lowers predicted cardiovascular disease risk in the canola oil multicenter randomized controlled trial. Am J Clin Nutr 2014;100:88-97.
99. Jones PJ, MacKay DS, Senanayake VK et al. High-oleic canola oil consumption enriches LDL particle cholesteryl oleate content and reduces LDL proteoglycan binding in humans. Atherosclerosis 2015;238:231-8.
100. Liu X, Garban J, Jones PJ et al. Diets Low in Saturated Fat with Different Unsaturated Fatty Acid Profiles Similarly Increase Serum-Mediated Cholesterol Efflux from THP-1 Macrophages in a Population with or at Risk for Metabolic Syndrome: The Canola Oil Multicenter Intervention Trial. J Nutr 2018;148:721-728.

101. Loganathan R, Nagapan G, Teng KT et al. Diets enriched with palm olein, cocoa butter, and extra virgin olive oil exhibited similar lipid response: a randomized controlled study in young healthy adults. Nutr Res 2022;105:113-125.
102. Pu S, Rodríguez-Pérez C, Ramprasath VR, Segura-Carretero A, Jones PJ. Dietary high oleic canola oil supplemented with docosahexaenoic acid attenuates plasma proprotein convertase subtilisin kexin type 9 (PCSK9) levels in participants with cardiovascular disease risk: A randomized control trial. Vascul Pharmacol 2016;87:60-65.
103. Rodríguez-Pérez C, Ramprasath VR, Pu S et al. Docosahexaenoic Acid Attenuates Cardiovascular Risk Factors via a Decline in Proprotein Convertase Subtilisin/Kexin Type 9 (PCSK9) Plasma Levels. Lipids 2016;51:75-83.
104. Sanders TA, Oakley FR, Crook D, Cooper JA, Miller GJ. High intakes of trans monounsaturated fatty acids taken for 2 weeks do not influence procoagulant and fibrinolytic risk markers for CHD in young healthy men. Br J Nutr 2003;89:767-76.
105. Tindall AM, Petersen KS, Skulas-Ray AC, Richter CK, Proctor DN, Kris-Etherton PM. Replacing Saturated Fat With Walnuts or Vegetable Oils Improves Central Blood Pressure and Serum Lipids in Adults at Risk for Cardiovascular Disease: A Randomized Controlled-Feeding Trial. J Am Heart Assoc 2019;8:e011512.
106. Gajendragadkar PR, Hubsch A, Mäki-Petäjä KM, Serg M, Wilkinson IB, Cheriyan J. Effects of oral lycopene supplementation on vascular function in patients with cardiovascular disease and healthy volunteers: a randomised controlled trial. PLoS One 2014;9:e99070.
107. Kim JY, Paik JK, Kim OY et al. Effects of lycopene supplementation on oxidative stress and markers of endothelial function in healthy men. Atherosclerosis 2011;215:189-95.
108. Lane JA, Er V, Avery KNL et al. ProDiet: A Phase II Randomized Placebo-controlled Trial of Green Tea Catechins and Lycopene in Men at Increased Risk of Prostate Cancer. Cancer Prev Res (Phila) 2018;11:687-696.
109. Paran E, Novack V, Engelhard YN, Hazan-Halevy I. The effects of natural antioxidants from tomato extract in treated but uncontrolled hypertensive patients. Cardiovasc Drugs Ther 2009;23:145-51.
110. Park E, Stacewicz-Sapuntzakis M, Sharifi R, Wu Z, Freeman VL, Bowen PE. Diet adherence dynamics and physiological responses to a tomato product whole-food intervention in African-American men. Br J Nutr 2013;109:2219-30.
111. Ried K, Frank OR, Stocks NP. Dark chocolate or tomato extract for prehypertension: a randomised controlled trial. BMC Complement Altern Med 2009;9:22.
112. Thies F, Masson LF, Rudd A et al. Effect of a tomato-rich diet on markers of cardiovascular disease risk in moderately overweight, disease-free, middle-aged adults: a randomized controlled trial. Am J Clin Nutr 2012;95:1013-22.
113. Wolak T, Sharoni Y, Levy J, Linnewiel-Hermoni K, Stepensky D, Paran E.

Effect of Tomato Nutrient Complex on Blood Pressure: A Double Blind, Randomized Dose⁻Response Study. Nutrients 2019;11.

114. Choi HD, Youn YK, Shin WG. Positive effects of astaxanthin on lipid profiles and oxidative stress in overweight subjects. Plant Foods Hum Nutr 2011;66:363-9.

115. Jabarpour M, Aleyasin A, Shabani Nashtaei M, Amidi F. Astaxanthin supplementation impact on insulin resistance, lipid profile, blood pressure, and oxidative stress in polycystic ovary syndrome patients: A triple-blind randomized clinical trial. Phytother Res 2024;38:321-330.

116. Saeidi A, Nouri-Habashi A, Razi O et al. Astaxanthin Supplemented with High-Intensity Functional Training Decreases Adipokines Levels and Cardiovascular Risk Factors in Men with Obesity. Nutrients 2023;15.

117. Spiller GA, Dewell A. Safety of an astaxanthin-rich Haematococcus pluvialis algal extract: a randomized clinical trial. J Med Food 2003;6:51-6.

118. Urakaze M, Kobashi C, Satou Y et al. The Beneficial Effects of Astaxanthin on Glucose Metabolism and Modified Low-Density Lipoprotein in Healthy Volunteers and Subjects with Prediabetes. Nutrients 2021;13.

119. Yoshida H, Yanai H, Ito K et al. Administration of natural astaxanthin increases serum HDL-cholesterol and adiponectin in subjects with mild hyperlipidemia. Atherosclerosis 2010;209:520-3.

120. Asemi Z, Alizadeh SA, Ahmad K, Goli M, Esmaillzadeh A. Effects of beta-carotene fortified synbiotic food on metabolic control of patients with type 2 diabetes mellitus: A double-blind randomized cross-over controlled clinical trial. Clin Nutr 2016;35:819-25.

121. Nierenberg DW, Bayrd GT, Stukel TA. Lack of effect of chronic administration of oral beta-carotene on serum cholesterol and triglyceride concentrations. Am J Clin Nutr 1991;53:652-4.

122. Redlich CA, Chung JS, Cullen MR, Blaner WS, Van Bennekum AM, Berglund L. Effect of long-term beta-carotene and vitamin A on serum cholesterol and triglyceride levels among participants in the Carotene and Retinol Efficacy Trial (CARET). Atherosclerosis 1999;143:427-34.

123. Ribaya-Mercado JD, Ordovas JM, Russell RM. Effect of beta-carotene supplementation on the concentrations and distribution of carotenoids, vitamin E, vitamin A, and cholesterol in plasma lipoprotein and non-lipoprotein fractions in healthy older women. J Am Coll Nutr 1995;14:614-20.

124. Shaish A, Harari A, Hananshvili L et al. 9-cis beta-carotene-rich powder of the alga Dunaliella bardawil increases plasma HDL-cholesterol in fibrate-treated patients. Atherosclerosis 2006;189:215-21.

125. van Poppel G, Hospers J, Buytenhek R, Princen HM. No effect of beta-carotene supplementation on plasma lipoproteins in healthy smokers. Am J Clin Nutr 1994;60:730-4.
